# Valency‐Controlled Multiphotochromism: Gated Switching and Photoswitchable Lewis Superacidity at Silicon

**DOI:** 10.1002/anie.2997357

**Published:** 2026-06-09

**Authors:** Lennart Stoess, Valentin D. Hannibal, Lutz Greb

**Affiliations:** ^1^ Anorganisch‐Chemisches Institut Ruprechts‐Karls Universität Heidelberg Heidelberg Germany

**Keywords:** chemistry, diarylethene, frustrated lewis pair, lewis acids and bases, metathesis, photochemistry, salt metathesis reaction, silicon, valency

## Abstract

Multiphotochromic systems promise multistate molecular information storage, yet strategies to control their switching‐state distributions and interchromophore communication remain scarce. Here, we demonstrate that silicon valency can serve as a molecular control element to gate multiphotochromism. Two silicon‐based diarylethene Lewis acids undergo stepwise twofold cyclization, with interconversion between tetra‐ and pentacoordinate states modulating electronic coupling between ligands. Donor coordination enables selective monocyclization, wavelength‐gated activation, or complete suppression of photoreactivity, depending on the bound base. In the donor‐free state, stepwise cyclization progressively enhances Lewis acidity and culminates in the first example of photoswitchable Lewis superacidity. In addition, dynamic Si─N/Si─O bond metathesis provides thermodynamic access to switching‐state distributions beyond statistical photochemical limits. These findings establish valency control as a design principle for multiphotochromic architectures.

## Graphical abstract

Multiphotochromic switching is controlled by silicon valency in a diarylethene‐based molecular architecture. Lewis base coordination gates electronic communication between the photochromic units, thereby enabling selective access to distinct switching states. This strategy reveals main‐group valency as a powerful design element for programmable and chemically gated photoresponse.

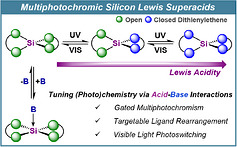

## 1 Introduction

**FIGURE 1:**
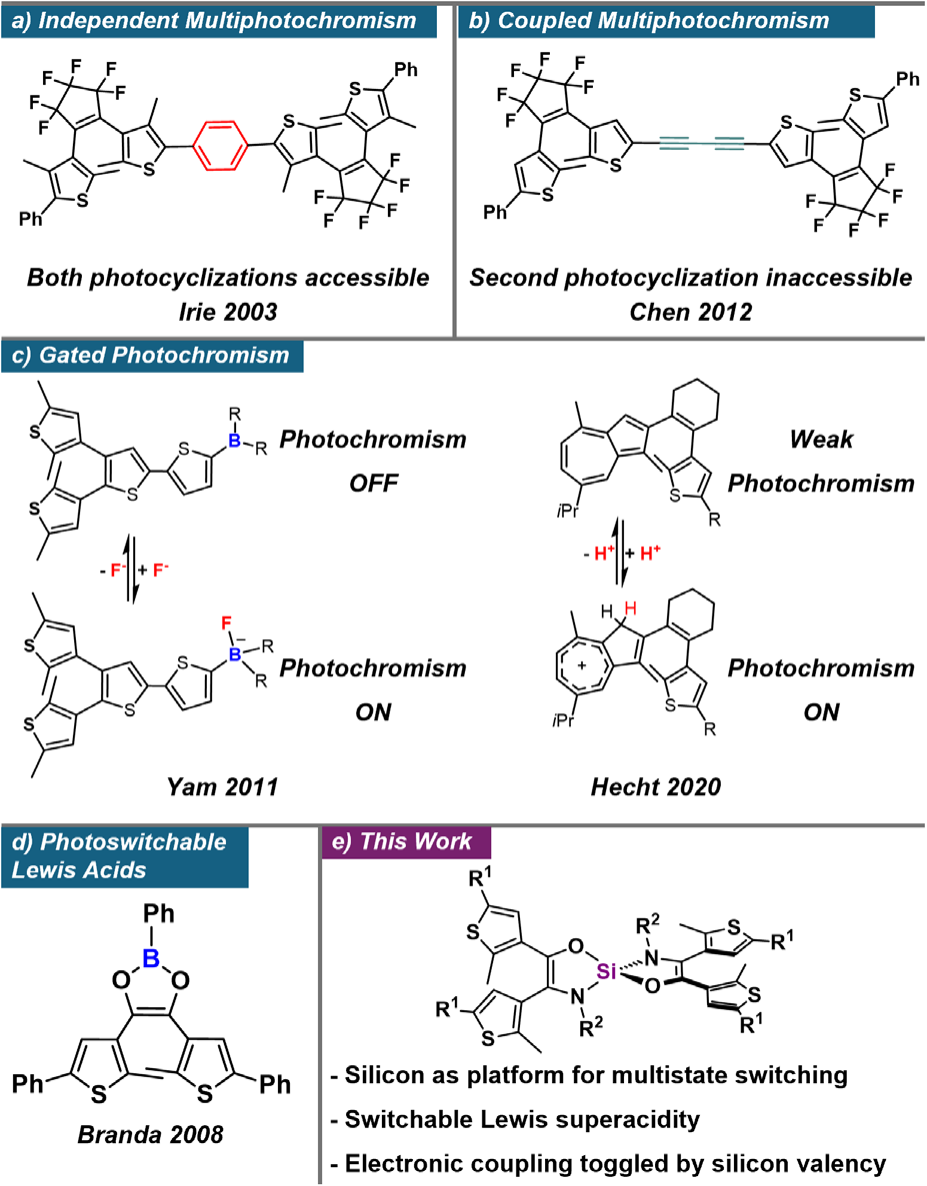
(a) Multiphotochromic system with accessible 2‐step photocyclization. (b) Multiphotochromic system with electronic coupling, preventing the second cyclization. (c) Examples for gated photochromism via fluoride ions and protons. (d) Photoswitchable Lewis acid, capable of reversible pyridine binding. (e) Multistate photoswitchable Lewis acid presented in this work.

Photochromism enables the reversible control of physicochemical properties by using light as an external stimulus [1]. While single photochromic units provide binary (“0‐1”) control, higher levels of complexity can be achieved by integrating multiple photochromic units within a single molecular framework, giving rise to multiple states (“0‐1‐2”) [2]. Multiphotochromic architectures can be divided into electronically coupled and decoupled systems [3]. Decoupled systems allow each unit to undergo photoswitching independently, as have been reported with phenylene (Figure 1a) [4, 5], sp^3^‐hybridized carbon [6], or metal [7, 8, 9, 10] spacers. In contrast, electronic coupling is leading to strongly interdependent switching behavior of the photochromic units, for example, by the direct connection or π‐conjugated alkyne spacers (Figure 1b) [7, 11, 12, 13]. However, in the reported systems, the degree of electronic coupling is predefined by molecular design, preventing control over coupled and decoupled behavior. Gated photochromism, in which the photochemical activity of a photoswitch is modulated by a second stimulus, represents a promising strategy to overcome this limitation [14]. Gated photochromic systems based on acid‐base chemistry (Figure 1c) [15, 16], redox processes [17], or host‐guest interactions [18, 19] have enabled control beyond simple photoisomerization. However, the application of gated photochromisim to multiphotochromic systems remains limited, particularly with respect to the regulation of electronic coupling between photoactive units. On the other hand, photoswitchable Lewis acids based on azobenzene or diarylethene (Figure 1d) [20, 21] scaffolds have been reported, enabling light‐induced modulation of acceptor strength. However, while multiphotochromic architectures have been explored for Lewis basic phosphines [22], similar concepts have not been extended to Lewis acids, leaving their potential for multistate control unexplored. In addition, their comparatively modest acceptor strength has so far precluded the realization of photoswitching into the Lewis superacidity regime.

Silicon Lewis acids offer an attractive platform to address these challenges: recent studies have shown that appropriately designed silicon centers can access Lewis superacidity [23, 24], and respond sensibly to changes in ligand electronic properties [25, 26]. The tetrahedral coordination environment of silicon allows incorporating two photochromic catechol‐type ligands without significant electronic coupling, due to a symmetry forbidden spiro conjugation [26]. At the same time, the ability of silicon for hypercoordination with pronounced geometrical changes [27] might unlock strategies for well‐defined control over interligand electronic coupling. Finally, the Lewis acidity of the silicon center provides a chemically readable output that might be influenced by the collective photochemical state. In contrast to previous gated photochromic systems that modulate individual switching events, here we present multiphotochromic silicon Lewis acids that integrate multistate photochemical switching, gated photochromism, and reversible modulation of Lewis superacidity within a single molecular framework (Figure 1e). Hence, while our previous work in this field [28] focused on the question: *Can the ligand switch the central element's (boron) properties?* we extend herein to: *Can the central element (silicon) also switch the ligand's properties?*

## 2 Results and Discussion

### 2.1 Synthesis

**FIGURE 2:**
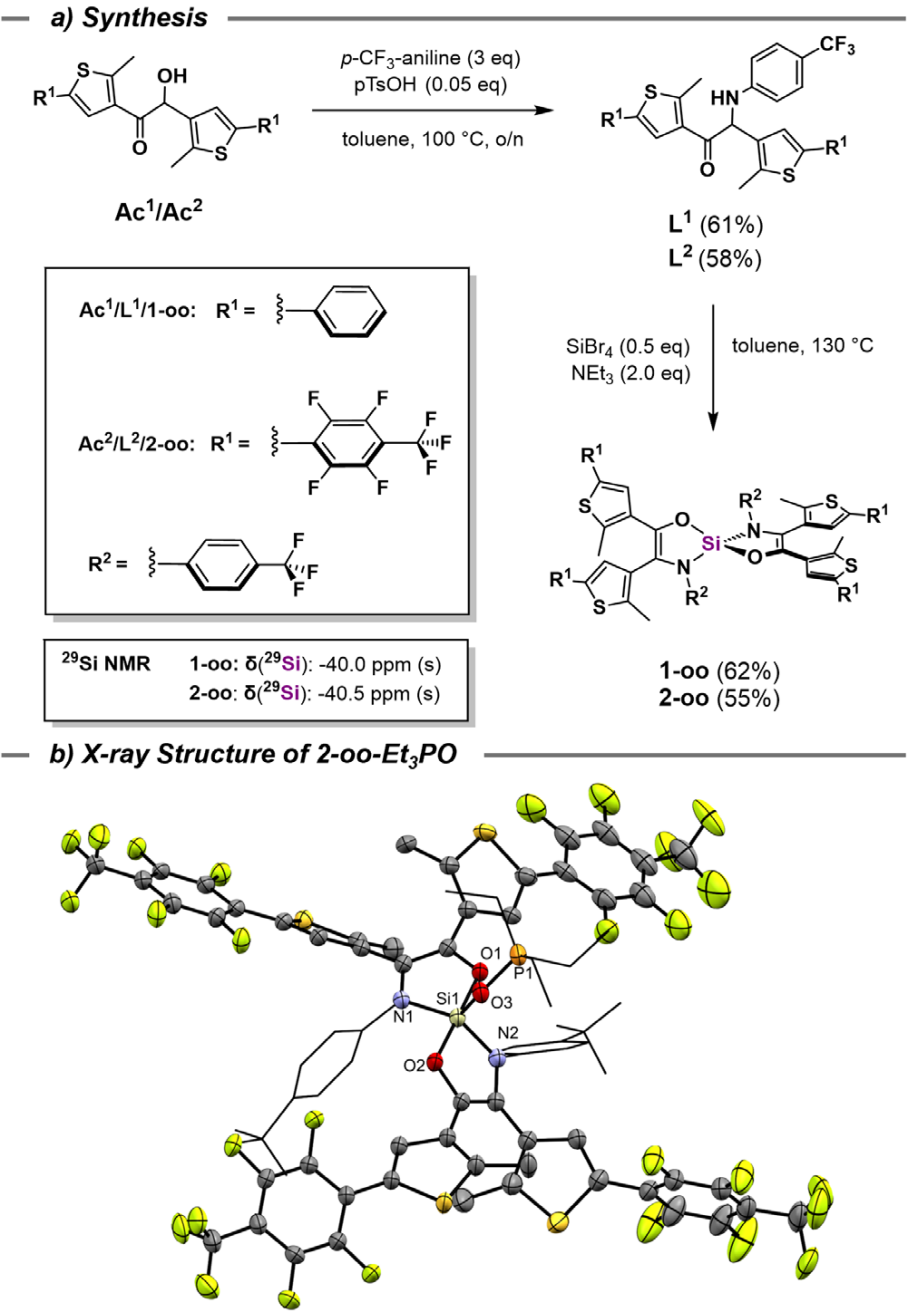
(a) Synthesis of photochromic silicon complexes. (b) Solid state structure of 2‐oo‐Et_3_PO. Hydrogen atoms, cocrystallized DCM molecules and disorder is omitted for clarity (Section S5 for details). Et_3_PO ethyl groups and nitrogen substituents are displayed as wireframe for clarity. Thermal ellipsoids at the 50% probability level. Selected bond length [pm] and angles [°]: Si1‐O2: 1.745(2), Si1‐N2: 1.762(2), Si1‐O3: 1.740(2), O1‐Si1‐O2: 178.72(11), N1‐Si1‐N2: 130.63(12), O2‐Si1‐N2: 88.57(10).

The use of NR/O substituents in the primary coordination sphere of silicon provides steric protection against oligomerization [29] to yield monomeric silicon species [30]. Hence, we initiated our investigation with the synthesis of suitable NR/O‐based dithienylethene ligands. Ligand synthesis was carried out by acid‐catalyzed condensation of acyloins **Ac^1^** and **Ac^2^** with 4‐trifluoromethyl‐aniline, giving aminoketone ligands **L^1^** and **L^2^**. Complexation of silicon was achieved by reaction with silicon tetrabromide and triethylamine at 130°C in toluene. Complexes **1‐oo** and **2‐oo** could be isolated as colorless solids after trituration with acetonitrile and recrystallization from DCM/*n*‐pentane at ‐40°C. While attempts to obtain crystals suitable for SCXRD failed, the identity of the complexes could be confirmed by ^29^Si NMR shifts of −40.0 ppm (**1‐oo**) and ‐40.5 ppm (**2‐oo**), in line with literature values for tetracoordinate silicon amidophenolates [29, 30], as well as by MALDI mass spectrometry. The ^1^H NMR spectra show line broadening due to hindered rotation of the thiophene residues. High‐temperature ^1^H NMR spectroscopy gives resolution to sharp signals, confirming the absence of hidden impurities (Section S1). For both **1‐oo** and **2‐oo**, the chemical equivalence of the ligands results in only one set of signals. The identity of **2** could be further confirmed by single crystals of its triethylphosphine oxide (Et_3_PO) adduct (Figure 2b), showing the expected trigonal bipyramidal coordination environment. Both ligands of **2‐oo‐Et_3_PO** adopt the photoactive antiparallel conformation.

### 2.2 Photoisomerization

**FIGURE 3:**
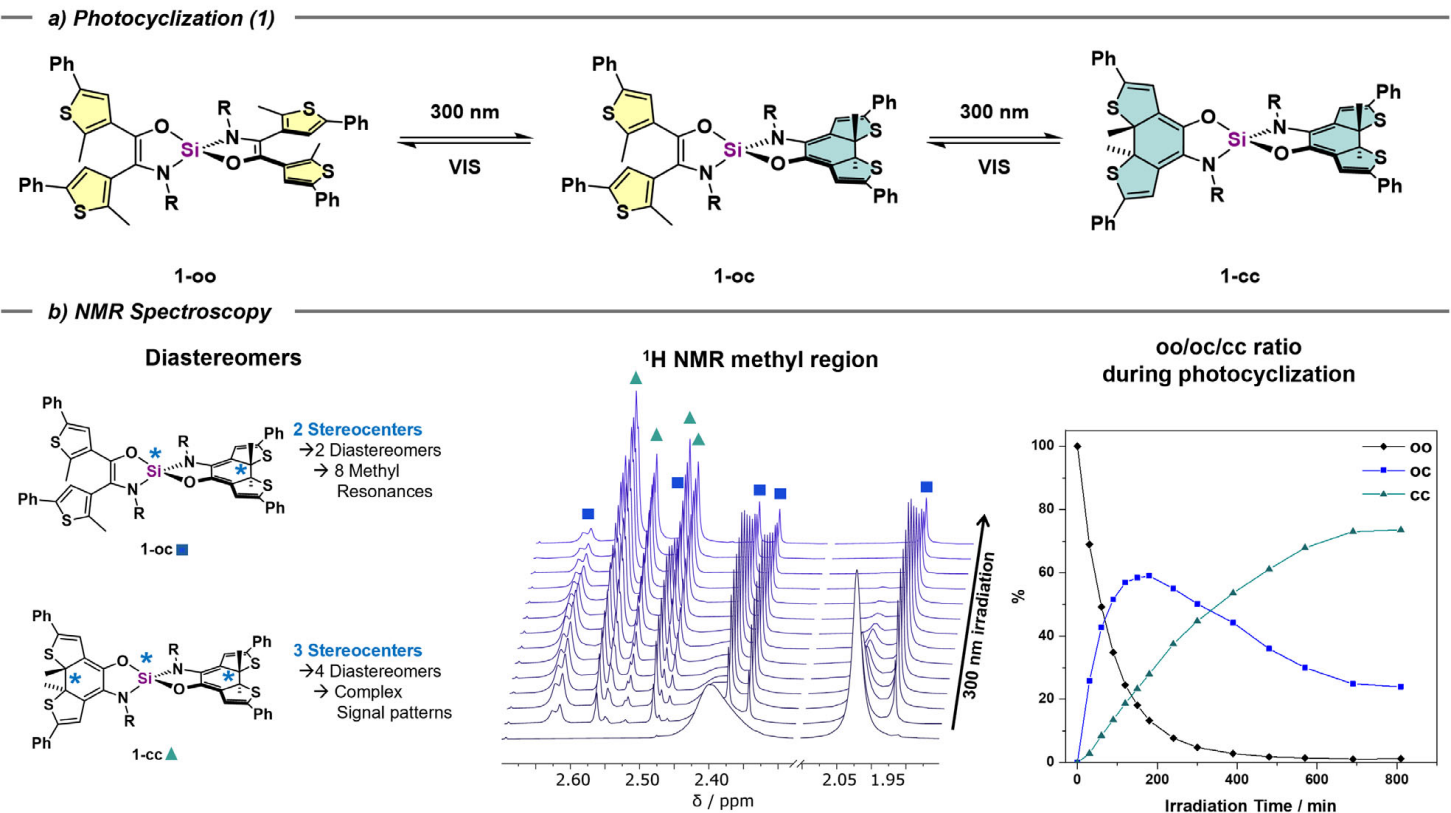
(a) Two‐step photoisomerization of 1. (b) Diastereomers of 1‐oc and 1‐cc. ^1^H NMR spectroscopic quantification of 1‐oo, 1‐oc and 1‐cc via methyl resonances. Ratio of 1‐oo, 1‐oc, and 1‐cc during irradiation at 300 nm in C_6_D_6_.

The photoisomerization of **1‐oo** and **2‐oo** occurs in two steps, going from both ligands open (**oo**) over one ligand closed (**oc**) to both ligands closed (**cc**) (Figure 3a). To determine whether the two photochromic ligands are electronically coupled or act independently, the photocyclization of compound **1‐oo** during irradiation at 300 nm was examined by NMR spectroscopy. Because of multiple possible diastereomers and the dynamics of the system, complex signal patterns emerge in the ^1^H NMR spectrum. For **1‐oc**, there are two stereocenters (helical chirality of the closed ligand and chirality of the silicon center), leading to 2 different diastereomers and 8 methyl resonances. The number of diastereomers is doubled by the additional stereocenter of the second closed ligand in **1‐cc**. Still, diagnostic signals in the methyl region (Figure 3b) allowed to quantify the amounts of **1‐oo**, **1‐oc**, and **1‐cc**. Upon irradiation, a close‐to‐statistical distribution of the three photoisomers was obtained (1% **1‐oo**, 24% **1‐oc**, 74% **1‐cc** after 800 min, corresponding to binomial distribution of overall 86% closed ligands, Section S4.1 for further details), indicating independent photoswitching of the two ligands at silicon.

We attribute this independence to the orthogonality of the ligands imparted by the central tetrahedral silicon atom, preventing electronic relaxation pathways from the excited open ligand to the energetically lower excited state of the closed ligand at the other side. Similarly, a statistical distribution (2/26/72 **2‐oo/2‐oc/2‐cc**) was obtained for compound **2** after UV irradiation for 480 min (Section S4.2). Treatment with broadband visible light for 30 min fully restored the open forms **1‐oo** and **2‐oo**.

### 2.3 UV–Vis Spectroscopy

**FIGURE 4:**
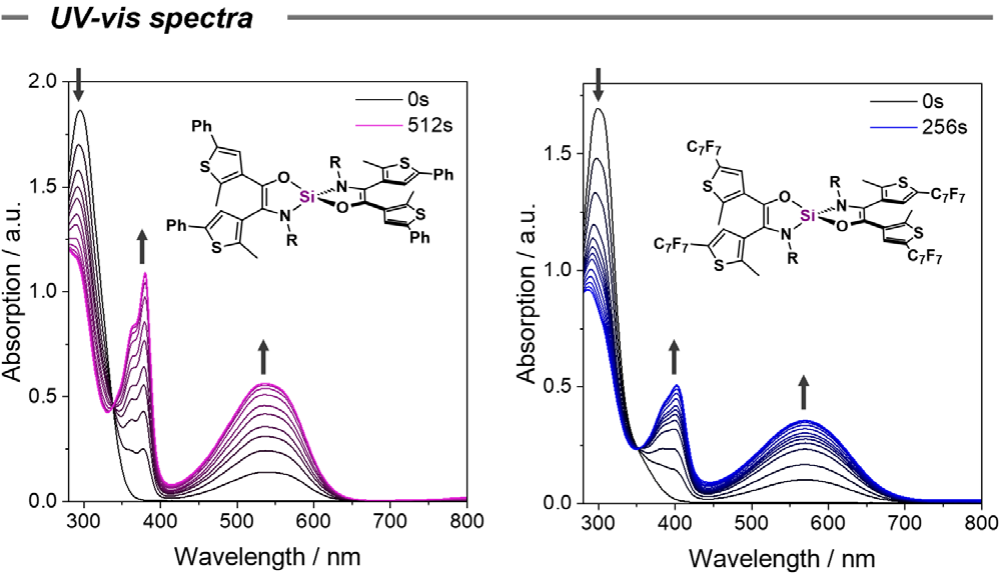
UV–vis absorption spectra of 1 (2.1 · 10^−5^ M, benzene) and 2 (1.6 · 10^−5^ M, benzene) during irradiation with 300 nm light.

The photoisomerization was additionally examined by UV–vis spectroscopy. For both **1‐oo** and **2‐oo**, colorless benzene solutions with no absorption in the visible region were obtained. The absorption maximum of **2‐oo** (301 nm) is redshifted compared to **1‐oo** (295 nm). Upon irradiation with UV light at 300 nm, absorption bands in the visible region appear, along with a color change to violet (**1**) and blue (**2**), in line with the formation of extended *π* systems in the closed forms (Figure 4). The photocyclization rate is higher for **2** compared to **1**, reaching saturation after 256 s, while more than 500 s are required for **1** under identical conditions. Remarkably, although the ratios of **oc** and **cc** vary during the course of irradiation, the absorption bands are not shifted with increasing irradiation time, further indicating that UV–vis spectral features of the closed form ligands are largely independent (decoupled) from the state of the second ligand at silicon for both **1** and **2**. This observation is corroborated by time‐dependent DFT calculations, which show that for the respective **cc** forms, visible excitation bands are only minimally shifted compared to **oc** (HOMO/HOMO‐1 spiro‐splitting: 0.12 eV for **1‐cc**, 0.11 eV for **2‐cc**, wB97X‐D3/def2‐TZVP/SMD(C_6_H_6_), Section S3.5). Irradiation for 60 s with visible light resulted in colorless solutions, and the initial UV–vis spectra were restored, albeit with reduced absorbance due to degradation. This degradation might be due to photochemical decomposition and the sensitivity to hydrolysis with trace water, which becomes significant at the low concentrations used for UV–vis spectroscopy. Therefore, photochemical fatigue resistance could not be measured by UV–vis spectroscopy.

### 2.4 Lewis Acidity

**FIGURE 5:**
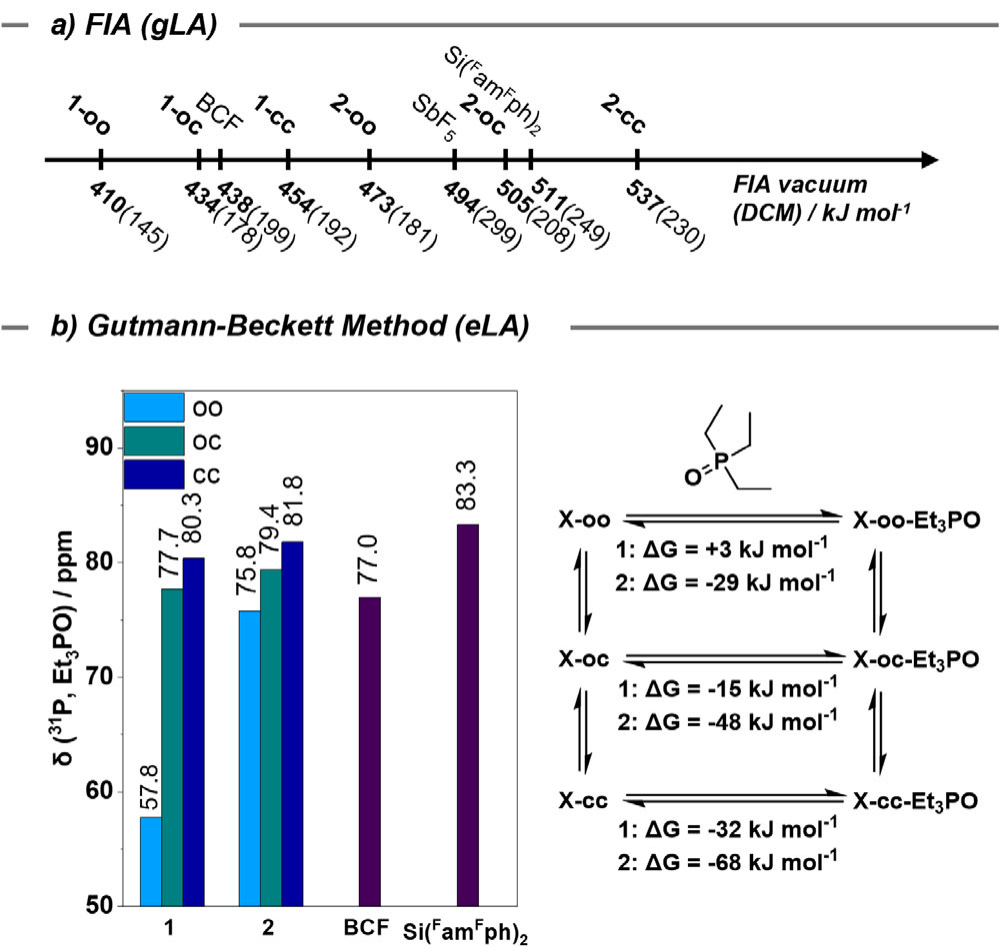
(a) Computed fluoride ion affinity of the photoisomers of **1** and **2**, and reference Lewis acids (against TMS/TMSF, DSD‐BLYP(D3BJ)/def2‐TZVPP//PBEh‐3c, for solvent corrected FIA values with SMD(DCM)). (b) Gutmann‐Beckett Method of compounds **1** and **2** in benzene with 0.5 equivalents of Et_3_PO. The weighted averages for the diastereomers of **1‐cc** and **2‐cc** is given. Computed free energy of Et_3_PO binding (DSD‐BLYP(D3BJ)/def2‐TZVPP/SMD(C_6_H_6_)//PBEh‐3c. Literature values for BCF [36] and Si(^F^am^F^ph)_2_ [30] in DCM.

To assess the global Lewis acidity of the different photoisomers of **1** and **2**, the fluoride ion affinities (FIA) were computed at the DSD‐BLYP(D3BJ)/def2‐TZVPP//PBEh‐3c level of theory. Photoswitching results in a marked, stepwise increase in gas phase FIA from **oo** to **oc** to **cc**, with substantially higher FIA for **2** compared to **1** due to the electron‐withdrawing nature of the thiophene substituents in **2** (Figure 5a). For compound **2‐cc**, the FIA is 537 kJ mol^−1^, exceeding that of SbF_5_ (494 kJ mol^−1^) in the gas phase. While limited to gas‐phase considerations, it represents the first reported case in which Lewis superacidity can be “switched on” by light as a trigger based on the definition of Lewis superacidity [31, 32]. It must be stressed that the large molecular size (i.e., relatively low solvation free energy) of **2** dampens the FIA in solution [33]. Consequently, the solvation corrected FIA_solv_ (DCM) are significantly attenuated and surpassed by SbF_5_, but still in the range of strong silicon Lewis acids, surpassing that of B(C_6_F_5_)_3_.

The effective Lewis acidity was evaluated by the Gutmann‐Beckett method [34, 35]. To solutions of **1‐oo** and **2‐oo** in C_6_D_6_, 0.5 equivalents of triethylphosphine oxide (Et_3_PO) were added. For **1‐oo**, a broad ^31^P NMR signal at 57.8 ppm (Figure 5b) was observed, indicating a weak association equilibrium resulting in an averaged minor shift. The weak binding is supported by DFT calculations, giving an association free enthalpy of + 3 kJ mol^−1^. Upon irradiation at 300 nm, separate signals in the ^31^P NMR spectrum appeared at 77.7 ppm and at 80.3 ppm, representing the GB shifts of **1‐co** and **1‐cc**, respectively (exchange dynamics do not influence these values, Section S4.3 for details). For **1‐cc‐Et_3_PO**, the structural rigidity of the closed ligand frameworks results in three sharp ^31^P resonances at 80.54, 80.24, and 79.94 ppm (80.3 ppm weighted average), indicating slight variations in effective Lewis acidity between diastereomers, but less significant compared to the differences between photoisomers. For **2‐oo**, association of Et_3_PO results in a shift of 75.8 ppm, which increased to 79.4 ppm (**2‐oc‐Et_3_PO**) and 81.8 ppm (81.77 and 81.81 ppm for two diastereomers of **2‐cc‐Et_3_PO**) upon irradiation. These values suggest a slightly lower effective Lewis acidity compared to the related perfluorinated Si(^F^am^F^ph)_2_ [30], but still surpassing B(C_6_F_5_)_3_ (BCF) [36]. The results confirm the modulation of both global (FIA) and effective (GB) Lewis acidity between three distinct states. While this feature might offer untapped opportunities for multistage control in Lewis acid catalysis, preliminary studies revealed the necessity of further optimizations for catalytic applications, which are therefore subject to future investigations. The following study focuses on a detailed investigation of the unique photochemical behaviors.

### 2.5 Gated Multiphotochromism

**FIGURE 6:**
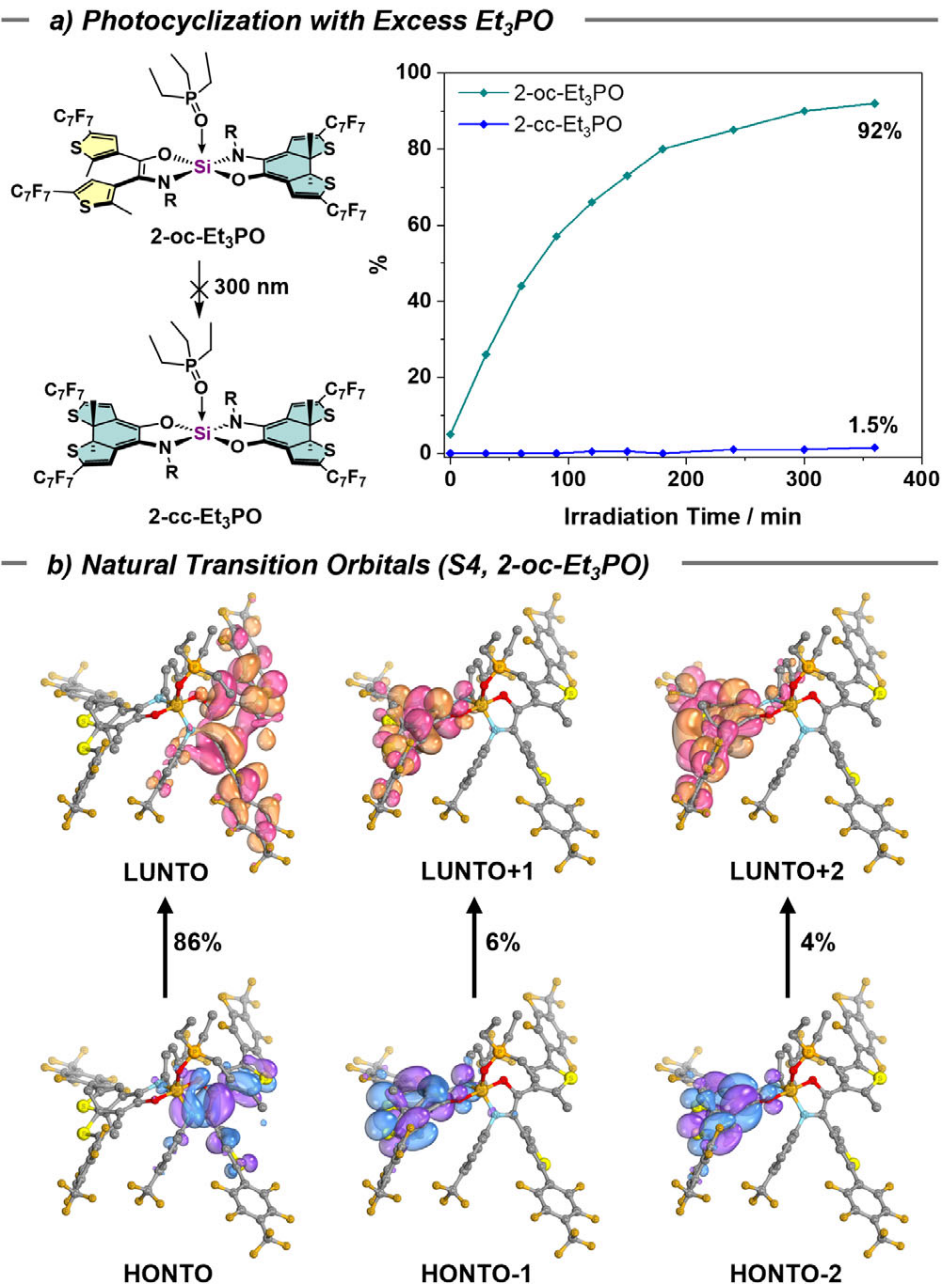
(a) Photocyclization of **2** with 2 equivalents of Et_3_PO in C_6_D_6_. (b) Natural transition orbitals for the S4 transition of **2‐oc‐Et_3_PO** (E = 4.05 eV, f = 0.149). Method: ωB97X‐D3/def2‐TZVP/SMD(C_6_H_6_).

Based on the loss of ligand orthogonality in the pentacoordinate adduct state (Figure 2b), we reasoned that the resulting coplanarity leads to electronic coupling across the silicon center, consequently inhibiting the second cyclization **oc**→**cc**. This would allow for the selective formation of only the **oc** form in the presence of a Lewis base, and thus a unique way of controlling multiphotochromic systems. Indeed, the second photocyclizations **oc**→**cc** are suppressed when an excess of Et_3_PO is present in solution, giving deviations from statistical switching‐state distribution. This effect is most pronounced for compound **2**, where the second photocyclization is almost completely inhibited in the presence of 2 equiv. Et_3_PO (< 2% **2‐cc** after 92% conversion to **2‐oc**, Figure 6a). For **1**, the conversion to **1‐cc** still occurs slowly in presence of excess Et_3_PO. This difference can be reasoned by the stronger Lewis acidity of all isomers of **2** compared to **1**, giving exclusively pentacoordinate species for **2‐oc** (ΔG = ‐48 kJ mol^−1^ for binding of Et_3_PO), while being in equilibrium for **1‐oc** (ΔG = ‐15 kJ mol^−1^, Figure 5b). The impact of the geometry on the second photocyclization **2‐oc**→**2‐cc** was investigated by TD‐DFT calculations. For both **2‐oc** and **2‐oc‐Et_3_PO**, the lowest‐energy excitations S1–S3 are localized on the *π* system of the closed ligand, while S4 is the *π*–π* transition of the open ligand responsible for the second ring‐closure. For tetrahedral **2‐oc**, this transition is entirely localized on the open ligand (Section S3.5). In contrast, the S4 transition for **2‐oc‐Et_3_PO** has significant (10%) *π*–π* contributions of the closed ligand (natural transition orbitals in Figure 6b). The mixing of open and closed ligand orbitals in this transition indicates electronic coupling between the photochromic units. This coupling may facilitate relaxation into lower‐energy excited states of the closed ligand and thereby reduce the efficiency of cyclization. With 0.5 equivalents of Et_3_PO, the **2‐oc→2‐cc** cyclization still occurs due to the presence of donor‐free species **2‐oc** in solution (Section S4.5). However, marked changes in the absorption features encouraged us to follow a second strategy to influence the switching state by silicon's valency, which is described in the next section.

### 2.6 Donor‐Induced Visible Light Photoswitching

**FIGURE 7:**
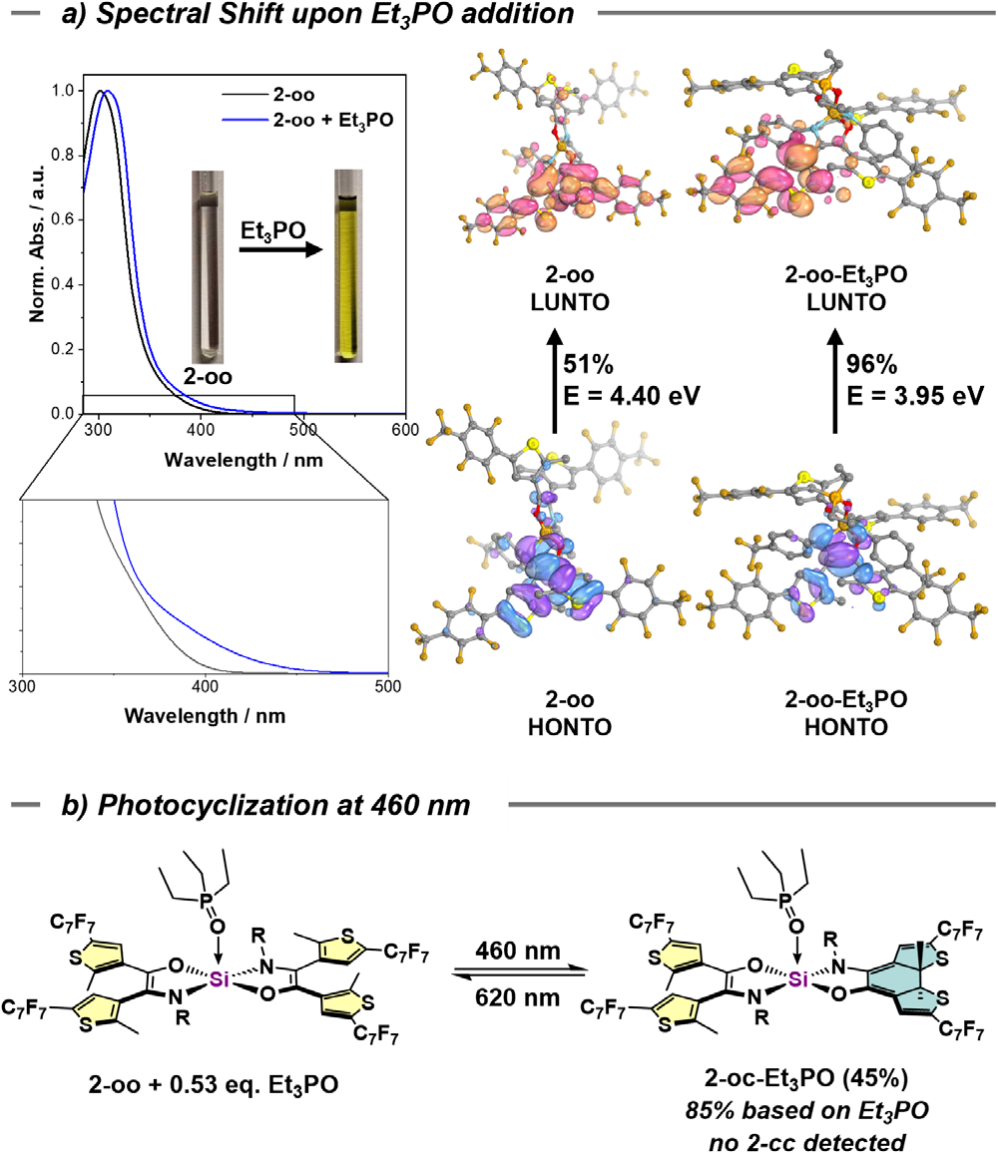
(a) Left: Spectral shift upon addition of Et_3_PO to **2‐oo** in benzene. Right: Natural transition orbitals for the S1 transition of **2‐oo** and **2‐oo‐Et_3_PO** (ωB97X‐D3/def2‐TZVP/SMD(C_6_H_6_)). Note: Due to the symmetry of **2‐oo**, excitations are not localized on one ligand, giving only 51% contribution for the shown orbital pair. (b) Selective photocyclization of **2‐oo‐Et_3_PO** in C_6_D_6_ at 460 nm, cycloreversion at 620 nm.

Addition of Et_3_PO to **2‐oo** leads to a color change from colorless to yellow, with the absorption maximum shifting from 301 nm (**2‐oo**) to 311 nm (**2‐oo‐Et_3_PO**). The low‐energy absorption band, which appears as a shoulder peak around 380 nm for **2‐oo‐Et_3_PO,** extends into the visible range (Figure 7a). This change can be reasoned by the charge‐transfer character of the low‐energy excitation (NTOs for the S1 transition of **2‐oo** and **2‐oo‐Et_3_PO** in Figure 7a). The binding of Et_3_PO exerts a destabilizing effect on the HOMO, lowering the HOMO–LUMO gap and reducing the excitation energy by 0.45 eV. As a result of the shifted absorption spectrum, photocyclization (**2‐oo‐Et_3_PO**→**2‐oc‐Et_3_PO**) becomes possible with visible light (460 nm blue LEDs). In addition to the advantage of avoiding UV light for cyclization, this gives another level of control over the two‐step photocyclization: at 460 nm, the cyclization of donor‐adducts can be induced selectively, while donor‐free complexes remain unchanged. Because irradiation at 460 nm selects for donor adducts which do not undergo a second photocyclization (cf. previous section), the mono‐cyclization (**2‐oo**→**2‐oc**) is induced selectively with visible light. In contrast to relying on the quantitative presence of pentacoordinate species to enable selectivity (cf. previous section), this approach enables selectivity even without an excess of donor added. Irradiating a solution of **2‐oo** with substoichiometric amounts of Et_3_PO (0.44 equivalents) at 300 nm yields a mixture of **2‐cc‐Et_3_PO** (25%), **2‐oc‐Et_3_PO** (19%) and **2‐oc** (37%), while irradiation at 460 nm selectively yields **2‐oc‐Et_3_PO** (45% conversion, 85% based on Et_3_PO, Figure 7b). The cycloreversion can be induced by irradiation at 620 nm, restoring the initial NMR spectra (Section S4.5).

**FIGURE 8:**
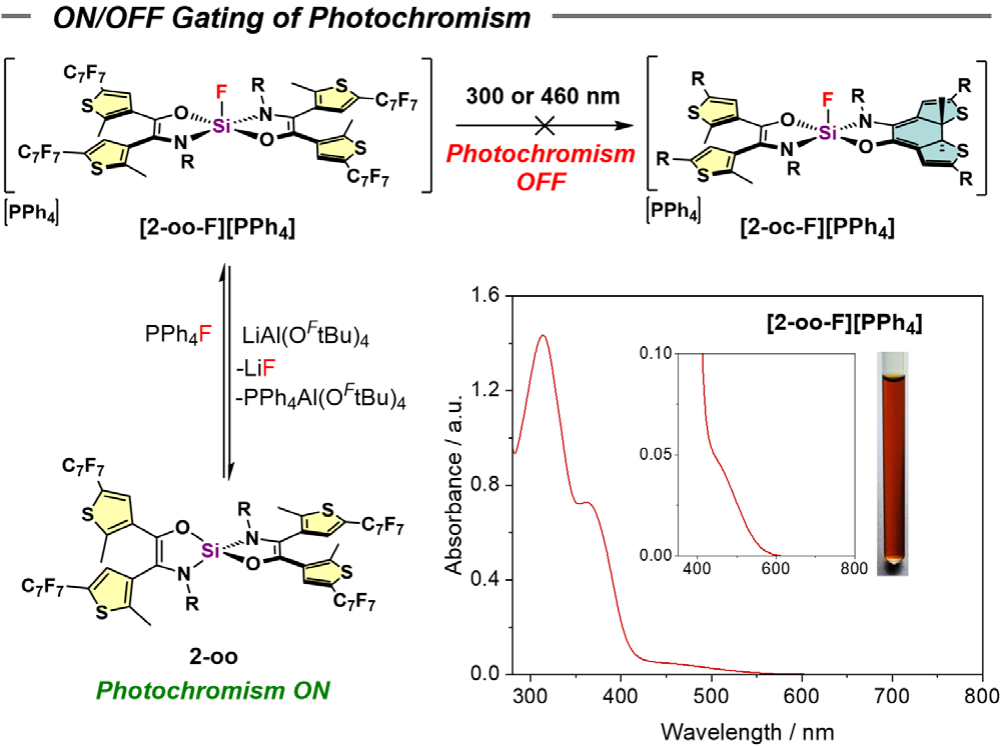
Charge‐transfer (CT) induced quenching of photochromism, and restoration of photochromic reactivity by fluoride abstraction. All Experiments were done with C_6_D_6_ as solvent. Bottom right: UV–vis absorption spectrum of **[2‐oo‐F][PPh_4_]** in benzene.

The ability of **2‐oo‐Et_3_PO** to undergo photocyclization despite the CT character of its low‐energy transition is notable, as strong donor‐acceptor interactions in diarylethene systems have been shown to suppress or fully inhibit photocyclization [37, 38]. In addition, inhibition has been reported in polar environments, where relaxation into twisted intramolecular charge‐transfer (TICT) states provides an efficient nonproductive decay pathway [39]. Aiming to fully inhibit the photoreactivity of **2**, a stronger Lewis base was employed. Adding a slight excess (1.05 equiv) of tetraphenylfluorophosphorane (PPh_4_F) results in an immediate color change to dark orange, the emergence of a red‐shifted absorption maximum at 362 nm and a broad CT band extending to 600 nm (Figure 8). ^1^H and ^19^F NMR spectroscopy support formation of the ion pair **[2‐oo‐F][PPh_4_]**. Irradiation at either 300 or 460 nm produced no detectable spectral changes, indicating complete suppression of photocyclization even with high‐energy excitation. This shows that the CT‐dominated excited states are photochemically nonproductive in this case, potentially reinforced by local polarity effects due to contact ion‐pair formation in benzene. Addition of LiAl(O*^F^* tBu)_4_ leads to the abstraction of fluoride, regenerating donor‐free **2‐oo** and its photoreactivity, allowing the generation of a 30/47/23 **oo/oc/cc** mixture (close‐to‐statistical distribution for 45% total ligand conversion) after 2 h of irradiation at 300 nm (Section S4.6).

These results demonstrate that the silicon center of **2** gives access for a three‐way gating of its multiphotochromic reactivity, with the photochromism fully switched off with bound fluoride, while Et_3_PO allows selective monocyclization, and the donor‐free complex allowing for cyclization towards both **2‐oc** and **2‐cc**.

### 2.7 Targetable Ligand Redistribution

**FIGURE 9:**
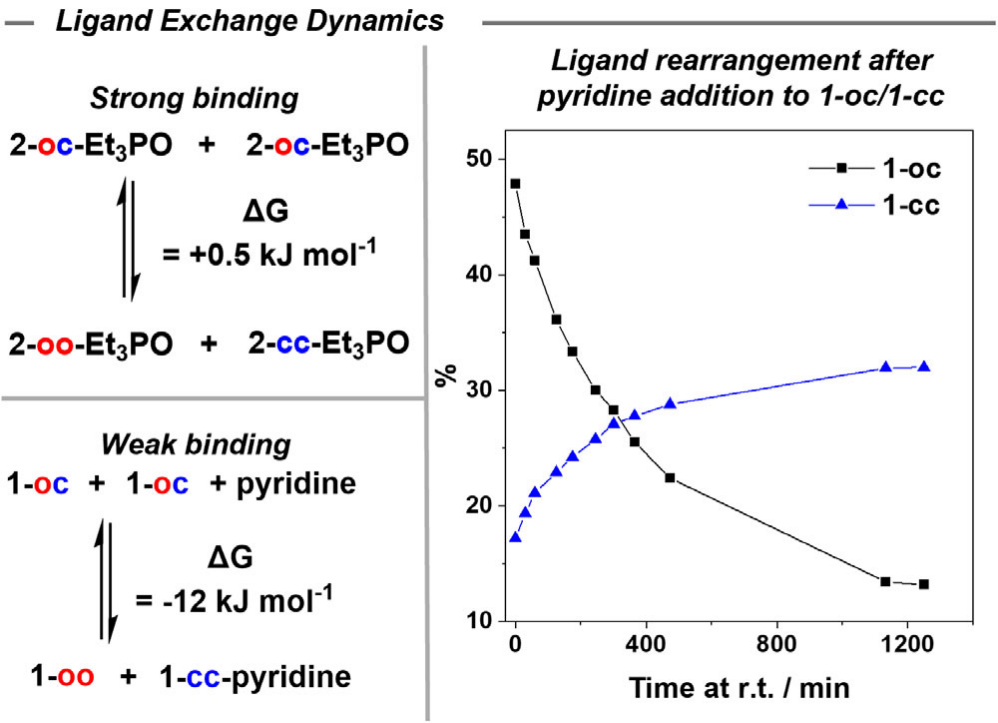
Left: Computed thermodynamics for ligand rearrangement for strongly binding donors (exergonic binding for all species) and weakly binding donors (exergonic only for **cc**). DSD‐BLYP(D3BJ)/def2‐TZVPP/SMD(C_6_H_6_)//PBEh‐3c. Right: Ligand rearrangement after pyridine addition to a **1‐oo**/**1‐oc**/**1‐cc** mixture in C_6_D_6_.

To allow for the targeting of the **cc** state beyond statistical distribution, the potential kinetic lability of Si─O and Si─N bonds towards bond metathesis was explored next [25]. Heating a solution of **2‐oc‐Et_3_PO** (generated by irradiation of **2** at 300 nm with 2 equivalents of Et_3_PO) to 60°C in the dark overnight shifts the ratio of **2‐oo‐Et_3_PO**/**2‐oc‐Et_3_PO**/**2‐cc‐Et_3_PO** from 2:96:2 to 12:76:12, while the total amount of closed ligands remains unchanged (Section S4.7, thermal ring opening can be excluded, vide infra). This indicates that ligand scrambling occurs at elevated temperature in the presence of Lewis bases, giving a thermal equilibrium of **2‐cc**, **2‐oc,** and **2‐oo** as their respective Et_3_PO adducts. The redistribution is unselective in the case of **2** and Et_3_PO since binding is exergonic for all species, giving close to thermoneutral reaction free energy for the redistribution (Figure 9). However, the thermodynamics can be manipulated by choosing a donor that does not bind strongly to either the **co** or **oo** form, but only to **cc**, providing a thermodynamic driving force for the **cc** form. The weaker Lewis acid **1** becomes beneficial for this purpose. For the combination of pyridine and **1**, the binding is close to thermoneutral (+ 2 kJ/mol) for the **oc** form, but gives a favorable computed reaction free enthalpy of −12 kJ/mol for the redistribution to **oo**/**cc** (Figure 9). While **2** requires heating for the ligand scrambling to occur, **1** was found to rearrange already at room temperature in the presence of donors. Indeed, addition of excess (5 equivalents) of pyridine to a statistical **oo/oc/cc** mixture in 35/48/17 ratio (generated by irradiating **1‐oo** for 2 h at 300 nm) leads to a ligand redistribution in favor of **oo** and **cc**. A final ratio of 55/13/32 is obtained after 20 h at room temperature, representing yet another handle to control this reaction network, and the first for enrichment of the **cc** form. It represents a unique example of narcistic ligand self‐sorting based on the hitherto underexplored field of Si─O/Si─O bond metathesis for constitutional dynamic chemistry [40, 41].

### 2.8 Thermal Ring‐Opening

**FIGURE 10:**
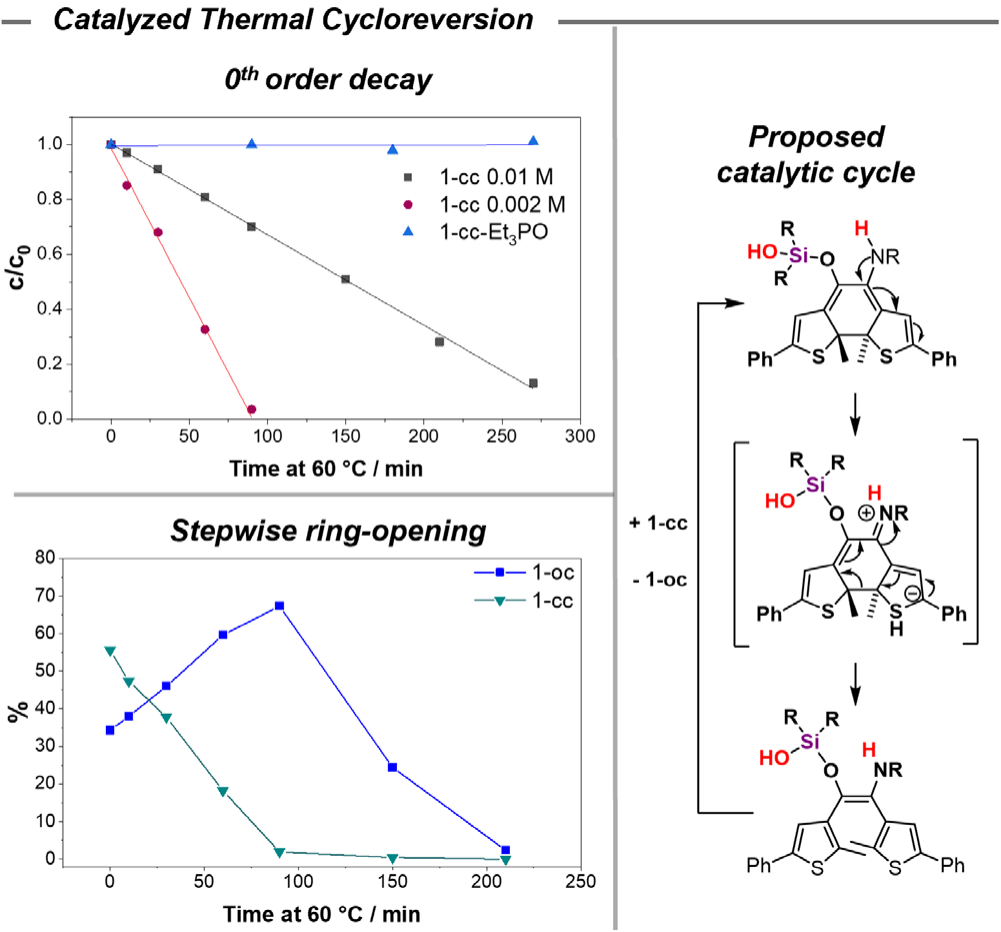
Thermal Ring‐opening. Top left: Decay rate (C_6_D_6_, 60°C) of **1‐cc** at different concentrations and of **1‐cc‐Et_3_PO**. Bottom left: Evolution of **1‐cc** and **1‐oc** at 60°C, showing stepwise ring‐opening (**cc**→**oc**→**oo**). Right: Proposed water catalyzed ring‐opening mechanism via dipolar intermediate.

During our experimental studies, we noted that in the absence of donors (but not in the donor adducts), compound **2‐cc** undergoes thermal ring‐opening at room temperature. Donor free **1‐cc** requires heating to 60°C to induce cycloreversion. These observations stand in contrast to the computed cycloreversion barriers (ΔG^‡^ = 137 kJ mol^−^^1^ for **1**‐**cc**; 135 kJ mol^−^^1^ for **2‐cc**, ωB97X‐D4/def2‐TZVPP/SMD(C_6_H_6_)// ωB97X‐D4/def2‐TZVP, Section S3.4), which predict negligible thermal reactivity under these conditions. Kinetic profiling of the ring‐opening of **1‐cc** at different temperatures and concentrations revealed a 0^th^ order decay, contrasting the typical first order thermal ring‐opening reactions of diarylethenes [42], but indicating catalyzed ring‐opening (Figure 10). Similarly for **2‐cc**, 0^th^ order decay was observed at room temperature (Section S4.8). Two observations narrowed down the possible catalytic species: (1) The first ring‐opening (**cc**→**co**) occurs strictly before the second opening (**co**→**oo**) starts, but the decay rate is similar for both processes (Figure 10, bottom). This observation suggests that the catalyst is Lewis basic and binds preferentially to **1‐cc**, as the Lewis acidity is the major difference between the photochemical isomers. (2) The Et_3_PO adduct **1‐cc‐Et_3_PO** decays orders of magnitude slower than the donor free **1‐cc,** with no detectable decomposition at 60°C in the same time period. Therefore, the ring‐opening is not accelerated by the coordination state of silicon itself, but by the specific nature of the donor. We propose that catalytic amounts of residual water in the solvent catalyze the ring‐opening by reversible Si─N bond hydrolysis (Figure 10, right). The hydrolysis might enable a dipolar ring‐opening, similar to suggested mechanisms for related triazole derivatives [43]. After ring‐opening, water is abstracted by remaining more Lewis acidic **1‐cc**, reformation of the Si─N bond, and closing the catalytic cycle. The thermal ring‐opening is significantly accelerated by adding 5% methanol (Section S4.8), supporting a protic impurity as the active catalyst.

The proposed dipolar intermediate is stabilized by electron‐withdrawing substituents at thiophene, explaining the lower stability of **2‐cc** compared to **1‐cc**. Notably, this mechanism–although unintended–represents a method for controlling switching‐state distributions of the donor‐free complexes (Section S4.9).

## 3 Conclusions

The present work establishes a conceptual advance at the interface of Lewis acid chemistry, photochemistry, and dynamic reaction networks. By exploiting the unique coordination flexibility of silicon, three orthogonal control strategies were realized within a single molecular framework. First, reversible interconversion between tetra‐ and pentacoordinate silicon modulates interligand electronic communication, enabling both independent and gated multiphotochromism. Second, donor coordination introduces charge‐transfer states that either unlock visible‐light addressability or completely suppress photoreactivity, depending on the bound ligand. Third, dynamic Si─N and Si─O bond metathesis enables thermodynamically directed redistribution of photoisomers beyond statistical photochemical limits. In parallel, the stepwise oo → oc → cc sequence progressively enhances both effective and global Lewis acidity. Owing to strongly electron‐withdrawing thiophene substituents, Lewis acidity is further enhanced in compound 2, with cyclization to 2‐cc constituting the first example of a photoswitchable Lewis superacid. Together, these findings expand multiphotochromism into a higher‐dimensional design space in which coordination number, valency, and constitutional dynamics serve as programmable parameters. More broadly, it can be anticipated that the underlying principle—coordination‐state‐dependent modulation of interchromophore coupling—should be transferable to other hypercoordinate main‐group systems, thus opening new opportunities for adaptive photoresponsive systems and functionally integrated molecular devices.

## Author Contributions

**Lennart Stoess**: conceptualization, data curation, investigation, validation, visualization, writing ‐ review and editing, writing ‐ original draft, methodology. **Valentin D. Hannibal**: data curation, investigation. **Lutz Greb**: conceptualization, methodology, supervision, resources, funding acquisition, writing ‐ review and editing, project administration.

## Conflicts of Interest

The authors declare no conflicts of interest.

## Supporting information




**Supporting File 1**: The authors have cited additional references within the Supporting Information. CCDC 2531569 contains the supplementary crystallographic data for this paper.

**Supporting File 2**: anie73095‐sup‐0002‐cif.zip.

## Data Availability

The data that supports the findings of this study are available in the supplementary material of this article.
