# Supplementary material for: Valency‐Controlled Multiphotochromism: Gated Switching and Photoswitchable Lewis Superacidity at Silicon
Source: Angew Chem Int Ed Engl. 2026 Jun 9;65(33):e2997357. doi: 10.1002/anie.2997357 (PMC13452506; doi:10.1002/anie.2997357)
Supplement: Supplementary file 1 [file ANIE-65-e2997357-s002.pdf]

Supporting Information

Valency-Controlled Multiphotochromism: Gated Switching and Photoswitchable Lewis Superacidity at Silicon

Lennart Stoess[a], Valentin Hannibal[a], and Lutz Greb*[a]

[a] Lennart Stoess, Valentin Hannibal, Prof. Dr. Lutz Greb
Anorganisch-Chemisches Institut,
Ruprechts-Karls Universität Heidelberg
Im Neuenheimer Feld 270, 69120 Heidelberg (Germany)
E-mail: greb@uni-heidelberg.de

Table of Contents

1 Synthesis and Characterization Data 1

1.1 General Considerations 1

1.2 L^1 2

1.3 L^2 5

1.4 $(L^1)_2Si$ (1-oo) 8

1.5 $(L^2)_2Si$ (2-oo) 12

2 UV-vis spectroscopy 16

2.1 Extinction Coefficients 16

2.2 Photocyclization and Photocycloreversion 17

3 Computational Section 18

3.1 General Information 18

3.2 Fluoride Ion Affinities 19

3.3 Lewis Base Affinities 20

3.4 Thermal Ring-Opening 21

3.5 TD-DFT 22

4 Reactivity Studies 28

4.1 NMR Scale Photocyclization (1) 30

4.2 NMR scale photocyclization (2) 33

4.3 Gutmann-Beckett Method 34

4.4 Photocyclization of 2 with excess Et_3PO 36

4.5 Photocyclization of 2-oo-Et_3PO at 460 nm and 300 nm 38

4.6 Photochemistry of Fluoride Adduct [2-oo-F][PPh_4] 40

4.7 Ligand Redistribution 43

4.8 Thermal Ring-Opening 47

4.9 Enrichment of oc Form by Selective Thermal Ring-Opening 51

5 X-Ray Crystallography 52

6 Coordinates 54

7 References 72

1 Synthesis and Characterization Data

1.1 General Considerations

All experimental manipulations were performed under inert and anhydrous conditions using standard Schlenk techniques with either argon or nitrogen atmospheres. Air- and moisture-sensitive compounds were handled and stored in a nitrogen-filled Sylatech glovebox. Unless stated otherwise, solvents were purified by established drying methods, stored over 3 Å or 4 Å molecular sieves, and degassed immediately before use by three consecutive freeze–pump–thaw cycles. p-Trifluoromethyl aniline was distilled and stored under argon prior to use. All other commercially available reagents were used without further purification. The identity and purity of newly prepared compounds were verified by multinuclear NMR spectroscopy and high-resolution mass spectrometry. ^{1}H, ^{13}C, ^{19}F, ^{31}P, and ^{29}Si NMR spectra were acquired at 298 K on either a *Bruker Ascend 400* or *Bruker Avance III 600* spectrometer at 298K unless otherwise noted. ^{13}C NMR spectra were recorded with ^{1}H decoupling, and ^{29}Si NMR spectra with inverse gated decoupling. Chemical shifts are reported in parts per million (δ, ppm) and referenced to the residual solvent resonances for ^{1}H and ^{13}C nuclei. Coupling constants (J) are expressed in hertz (Hz). Data processing and visualization were carried out using MestReNova 14.3. MALDI mass spectra were obtained using a Bruker timsTOFfleX instrument at the mass spectrometry facility of the Institute of Organic Chemistry, University of Heidelberg. UV-vis spectral measurements were carried out on a JASCO V-570 UV/VIS/NIR spectrophotometer in a 2 mm or 10 mm quartz cuvette with a J-Young valve. Ac^{1} and Ac^{2} were synthesized according to published procedures.[1]

1.2 L[1]

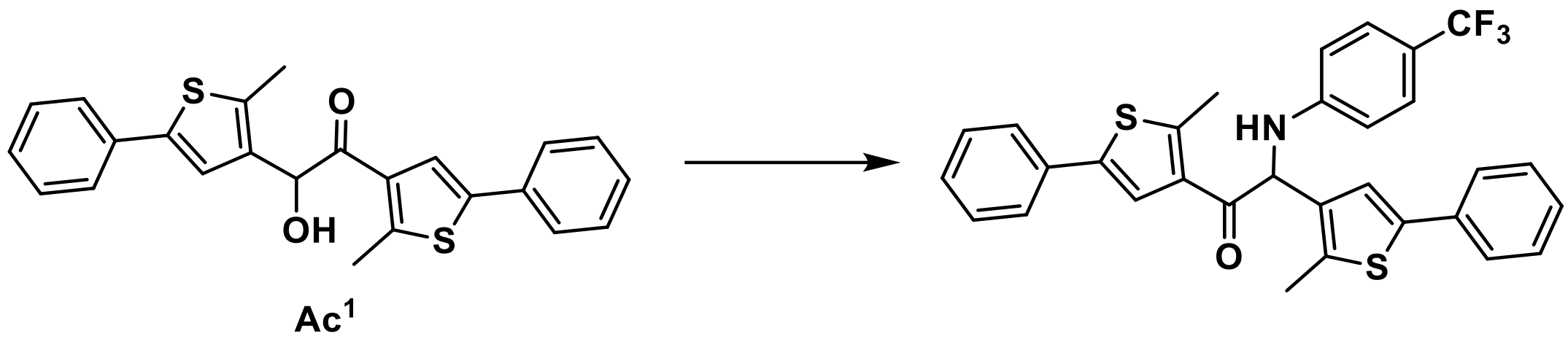

Ac[1] (0.93 g, 2.3 mmol, 1.0 eq), 4-(trifluoromethyl)aniline (0.87 ml, 6.9 mmol, 3.0 eq), and p-TsOH*H_2O (22 mg, 0.11 mmol, 0.05 eq) were suspended in toluene (30 ml) and degassed by argon sparging for 10 minutes. The mixture was subsequently heated to 100 °C overnight under an argon atmosphere. After cooling to room temperature, sat. Na_2CO_3 (50 ml) and ethyl acetate (100 ml) were added and the phases separated. The organic phase was washed with brine (2x50 ml), dried over $MgSO_4$ and filtered. Removal of the solvent in vacuo gave a yellow oil, which was triturated with PE and the suspension filtered. The solid was washed with PE (5x10 ml) and dried in vacuo. Off-white powder (0.77 g, 1.4 mmol, 61%).

^{1}H NMR (400 MHz, $CDCl_3$) δ 7.54 – 7.44 (m, 5H, *C**H***), 7.44 – 7.36 (m, 4H, *C**H***), 7.35 – 7.28 (m, 3H, *C**H***), 7.24 (t, *J* = 7.6 Hz, 1H, p-*C₆**H**₅*), 7.12 (s, 1H, *thiophene-C**H***), (d, *J* = 8.4 Hz, 2H, *ortho-NC₆**H**₄CF₃*), 5.76 (d, *J* = 5.5 Hz, 1H, *C**H**-N*), 5.53 (d, *J* = 5.5 Hz, 1H, *N**H***), 2.73 (s, 3H, *C**H**₃*), 2.64 (s, 3H, *C**H**₃*).

^{13}C NMR (101 MHz, $CDCl_3$) δ 191.7 (s, ***C***=*O*), 151.4 (s, ***C**q*), 148.8 (s, ***C**q*), 141.9 (s, ***C**q*), 140.5 (s, ***C**q*), 136.7 (s, ***C**q*), 134.2 (s, ***C**q*), 133.8 (s, ***C**q*), 133.4 (s, ***C**q*), 129.3 (s, ***C**H, ortho-**C**₆H₅*), 129.0 (s, ***C**H, ortho-**C**₆H₅*), 128.2 (s, ***C**H, para-**C**₆H₅*), 127.7 (s, ***C**H, para-**C**₆H₅*), 126.8 (q, *J* = 3.7 Hz, ***C**H, meta-N**C**₆H₄CF₃*), 125.8 (s, ***C**H, meta-**C**₆H₅*), 125.6 (s, ***C**H, meta-**C**₆H₅*), 125.0 (q, *J* = 270.5 Hz, ***C**F₃*) 122.8 (s, *thiophene-**C**H*), 122.3 (s, *thiophene-**C**H*), 119.5 (q, *J* = 32.6 Hz, ***C**q, **C**-CF₃*) , 112.7 (s, ***C**H, ortho-N**C**₆H₄CF₃*), 58.9 (s, ***C**H-N*), 16.4 (s, ***C**H₃*), 14.0 (s, ***C**H₃*).

Note: The signal at 133.4 ppm is the overlap of two quaternary carbon atoms.

^{19}F NMR (377 MHz, $CDCl_3$) δ -61.1 (s, 3F, *C**F**₃*).

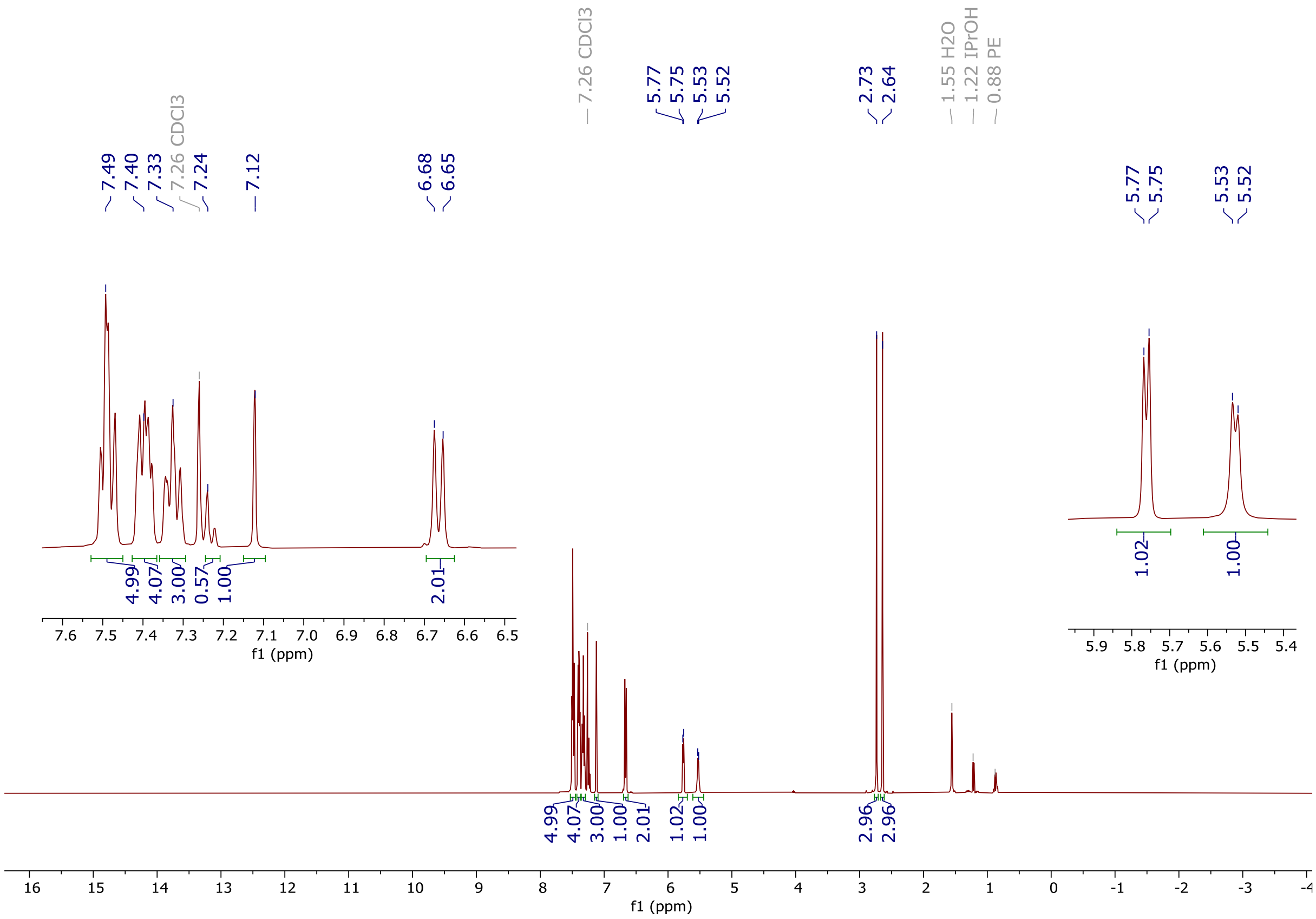

Figure S1.1. ^{1}H NMR spectrum (400 MHz, $CDCl_3$) of **L[1]**.

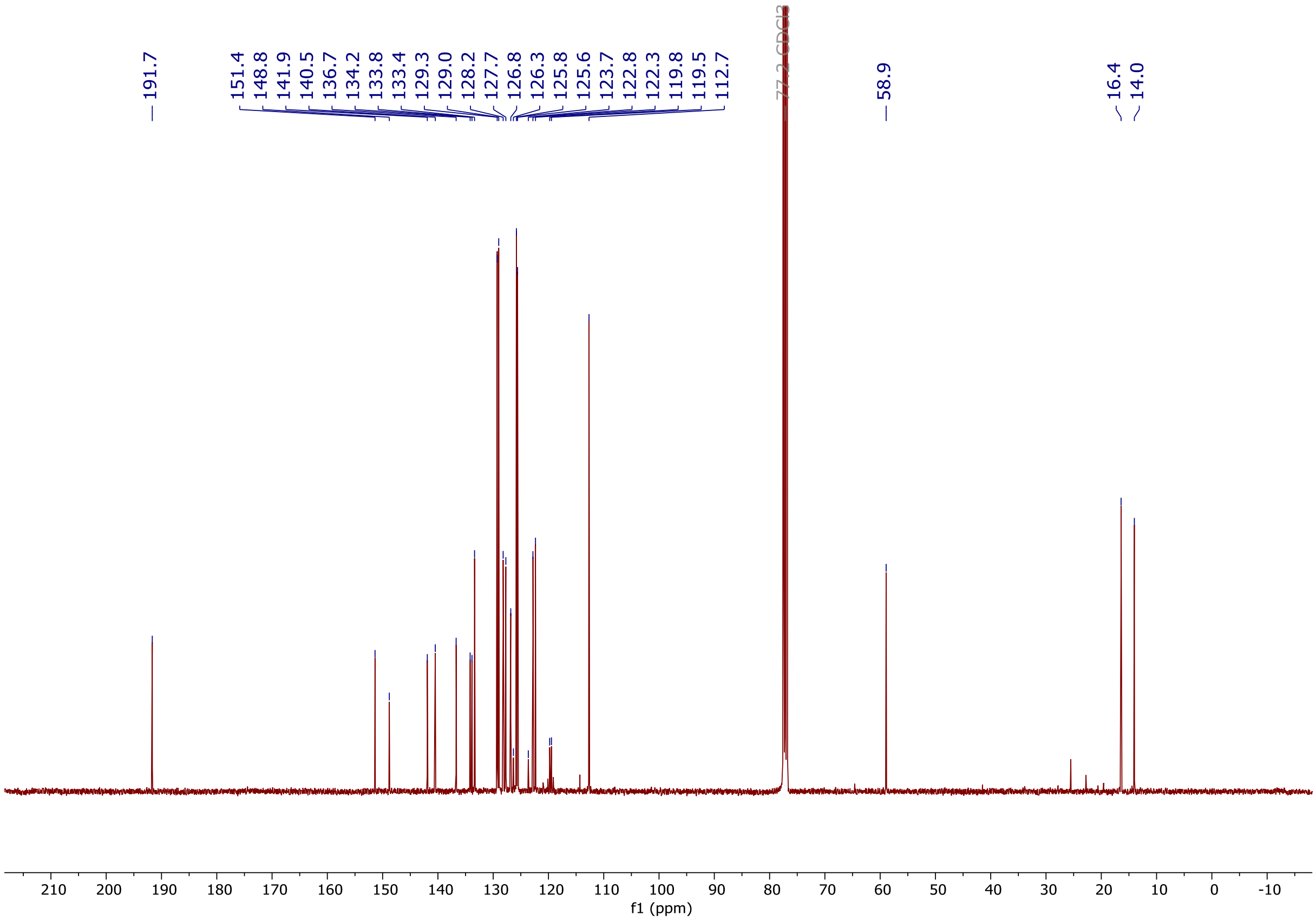

Figure S1.2. ^{13}C NMR spectrum (101 MHz, $CDCl_3$) of **L[1]**.

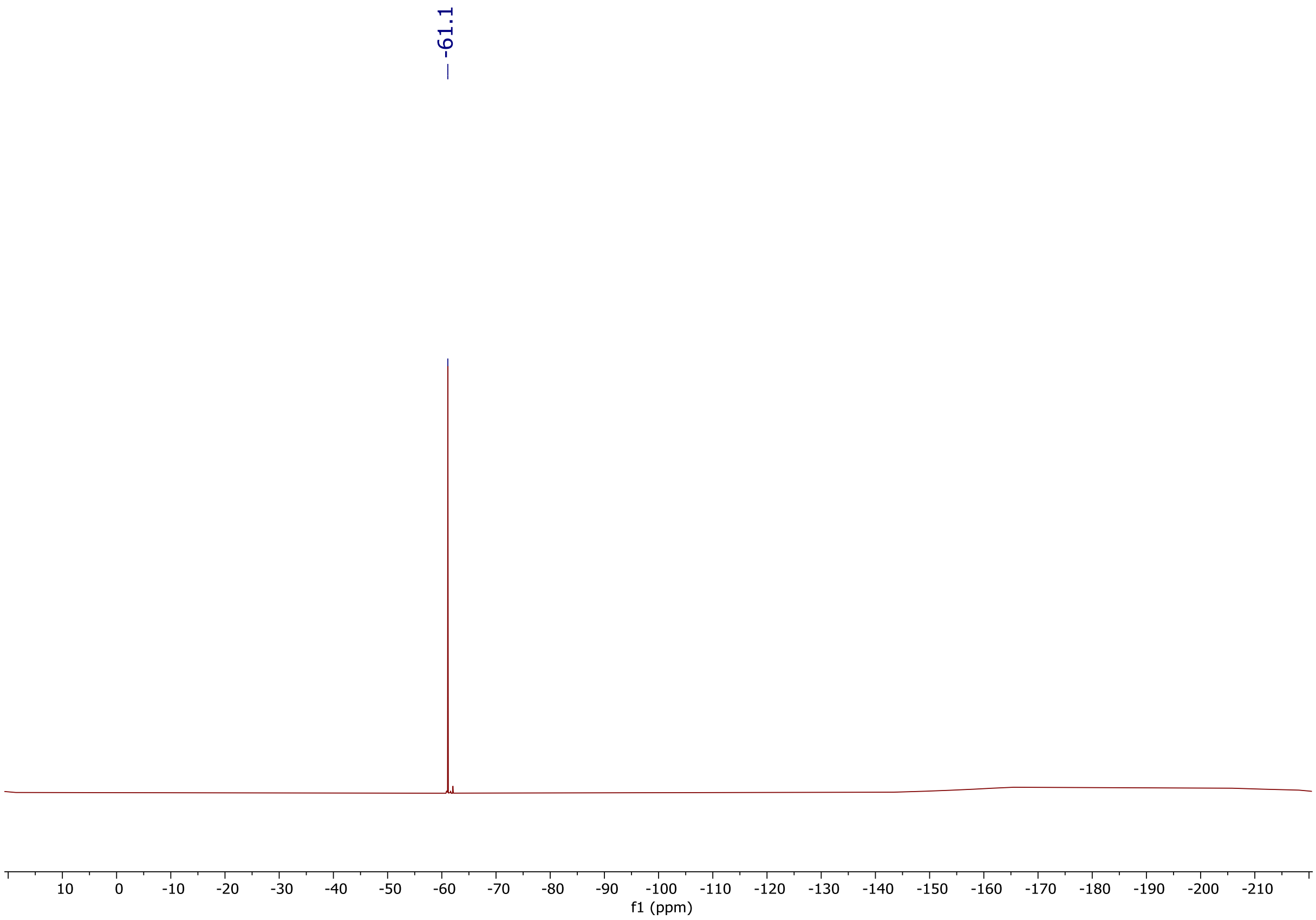

Figure S1.3. ^{19}F NMR spectrum (377 MHz, $CDCl_3$) of **L[1]**.

1.3 L[2]

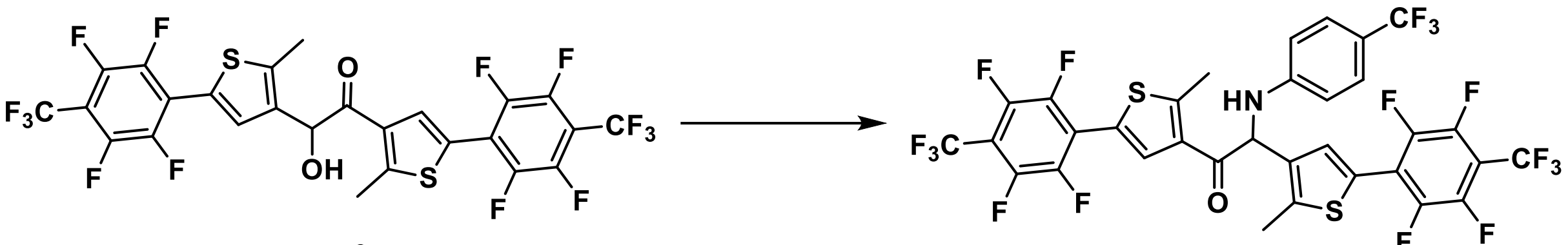

Ac^2 (0.44 g, 0.64 mmol, 1.0 eq), 4-(trifluoromethyl)aniline (0.24 ml, 1.9 mmol, 3.0 eq), and p-TsOH*H_2O (6 mg, 0.03 mmol, 0.05 eq) were suspended in toluene (20 ml) and degassed by argon sparging for 10 minutes. The mixture was subsequently heated to 100 °C overnight under an argon atmosphere. After cooling to room temperature, sat. Na_2CO_3 (50 ml) and ethyl acetate (200 ml) were added and the phases separated. The organic phase was washed with brine (2x100 ml), dried over $MgSO_4$ and filtered. Removal of the solvent in vacuo gave a yellow oil, which was dissolved in MeOH (50 ml) and the solvent removed again in vacuo.* Sonication with MeOH (30 ml) resulted in the precipitation of a colorless solid, which was filtered off, washed with cold MeOH (4x5 ml), and dried in vacuo. Colorless powder (0.31 g, 0.37 mmol, 58%).

**Note: Dissolution in MeOH and removal in vacuo is required in some cases to remove traces of toluene, which prevent precipitation from methanol.*

^{1}H NMR (400 MHz, $CDCl_3$) δ 7.95 (s, 1H, *thiophene-C**H***), 7.58 (s, 1H, *thiophene-C**H***), 7.40 (d, *J* = 8.5 Hz, 2H, *meta-NC_6**H_4**CF_3*), 6.66 (d, *J* = 8.5 Hz, 2H, *ortho-NC_6**H_4**CF_3*), 5.83 (d, *J* = 4.5 Hz, 1H, *C**H**-N*), 5.56 (d, *J* = 4.5 Hz, 1H, *N**H***), 2.80 (s, 3H, *C**H_3***), 2.64 (s, 3H, *C**H_3***).

^{13}C NMR (101 MHz, $CDCl_3$) δ 190.6 (s, ***C**=O*), 156.0 (t, *J* = 4.6 Hz, ***C_q***), 148.3 (s, ***C_q***), 144.7 (dm, *J* = 260 Hz, ***C**F*), 143.7 (d, *J* = 250 Hz, ***C**F*), 142.7 (t, *J* = 4.8 Hz, ***C_q***), 133.3 (s, ***C_q***), 133.1 (s, ***C_q***), 132.0 (t, *J* = 6.8 Hz, *thiophene-**C**H*), 130.9 (t, *J* = 6.8 Hz, *thiophene-**C**H*), 126.9 (q, *J* = 3.7 Hz, ***C**H, meta-N**C_6**H_4CF_3*), 124.9 (q, *J* = 270.3 Hz, *NC_6H_4-**CF_3***), 124.5 (s, ***C_q***), 123.2 (s, ***C_q***), 120.8 (q, *J* = 270 Hz, *perfluorotolyl-CF_3*) 120.2 (q, *J* = 32.8 Hz, ***C_q**, para-N**C_6**H_4CF_3*), 117.8 (t, *J* = 13.8 Hz, ***C_q***), 117.2 (t, *J* = 13.8 Hz, ***C_q***), 112.8 (s, ***C**H, ortho-N**C_6**H_4CF_3*), 108.7 – 106.9 (m, ***C_q**, perfluorotolyl-**C**-CF_3*), 59.0 (s, ***C**H-N*), 16.2 (s, ***C**H_3*), 13.6 (s, ***C**H_3*).

Note: Resonances at 144.7 (dm), 143.7 (dm), 120.8 (q), and 108.7-106.9 (m) ppm are overlapping signals for the two non-equivalent perfluorotoluene substituents. Coupling constants for these resonances are given with low accuracy due to the overlap.

^{19}F NMR (377 MHz, $CDCl_3$) δ -56.2 (t, *J* = 21.2 Hz, 3F, *perfluorotolyl-C**F_3***), -56.2 (t, *J* = 21.2 Hz, 3F, *perfluorotolyl-C**F_3***), -61.3 (s, 3F, *NC_6H_3C**F_3***), -138.3 – -138.8 (m, 4F, *C**F***), -139.5 – -140.3 (m, 2F, *C**F***), -140.5 – -141.0 (m, 2F, *C**F***).

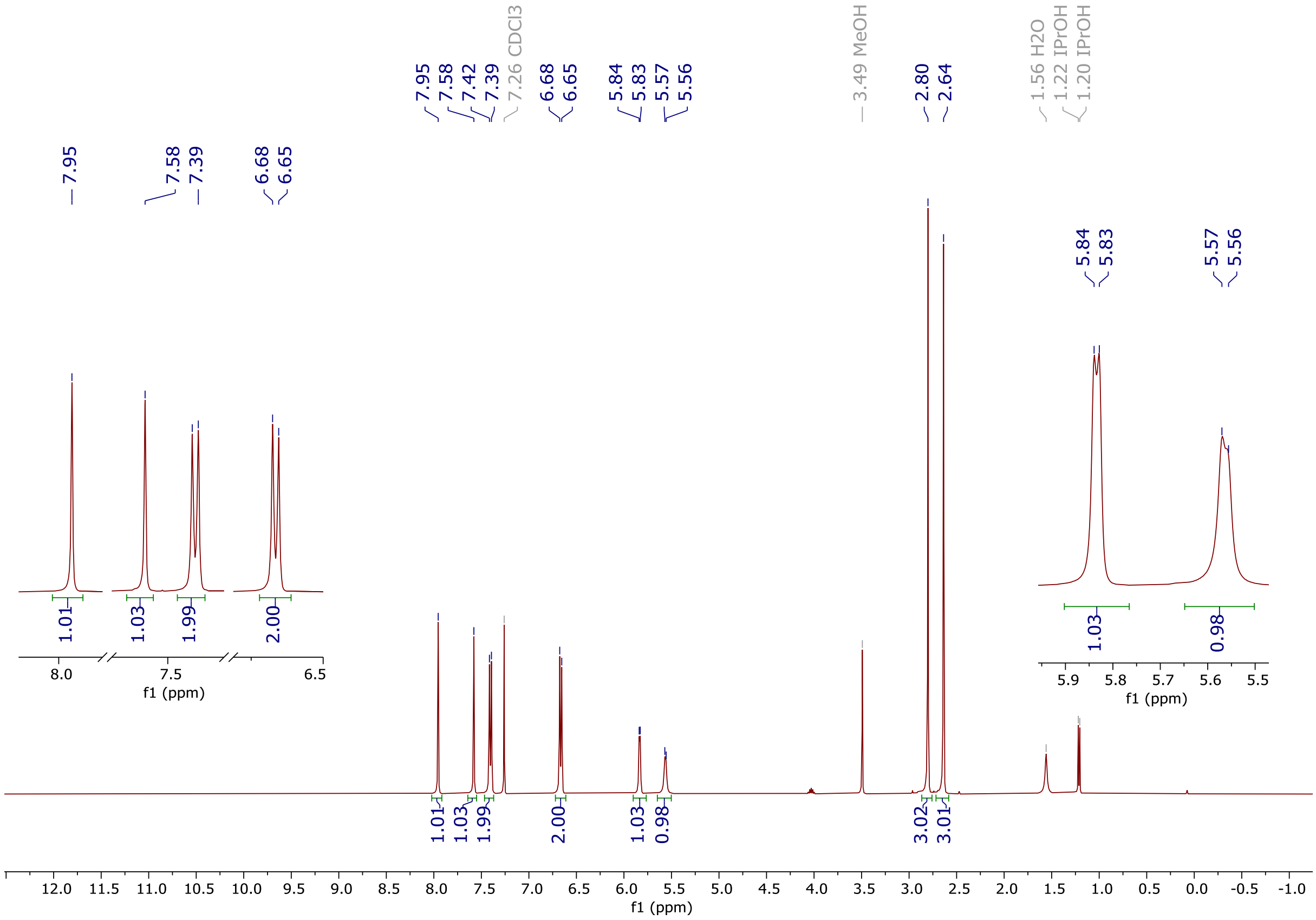

Figure S1.4. ^{1}H NMR spectrum (400 MHz, $CDCl_3$) of $\mathbf{L^2}$.

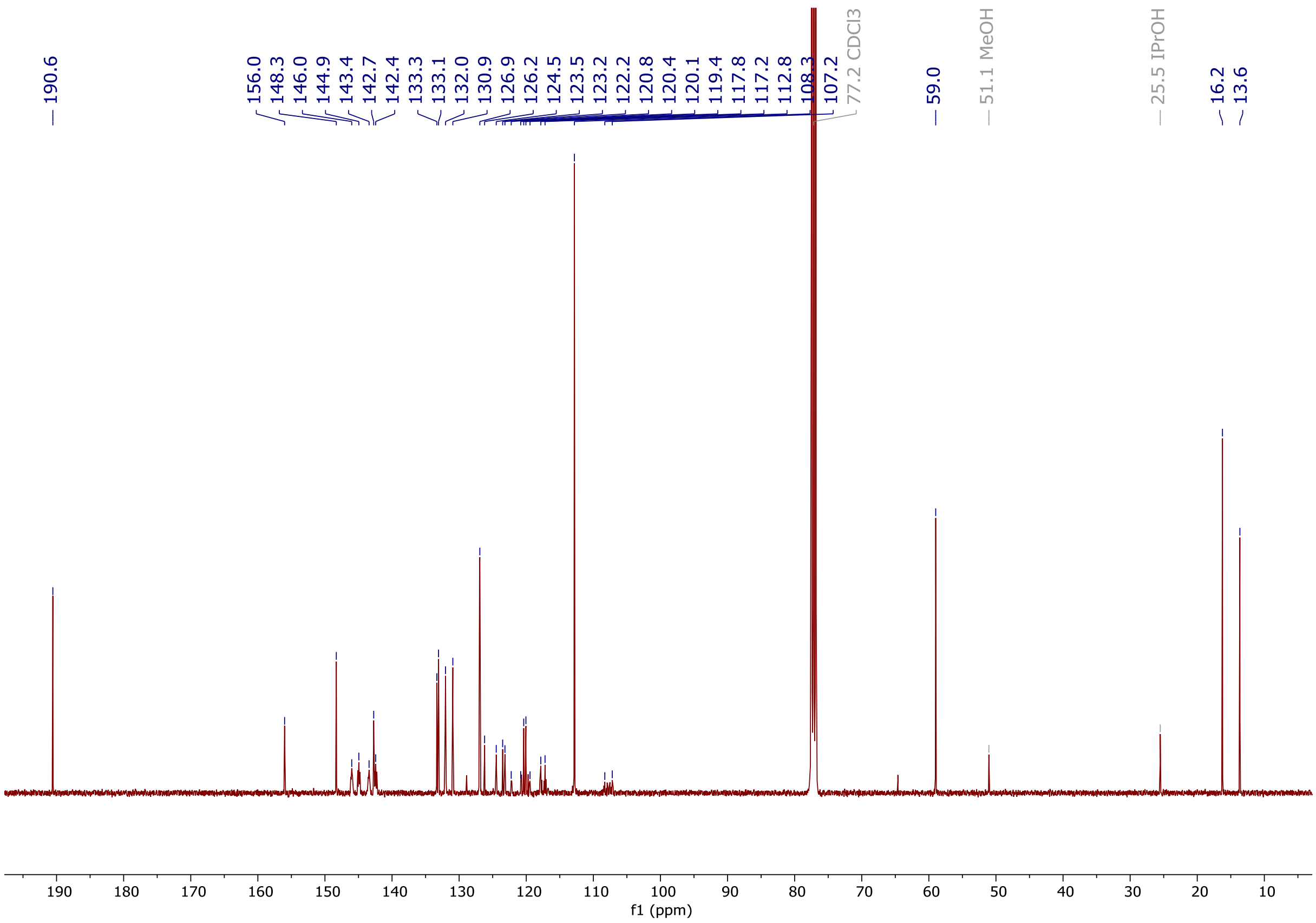

Figure S1.5. ^{13}C NMR spectrum (101 MHz, $CDCl_3$) of $\mathbf{L^2}$.

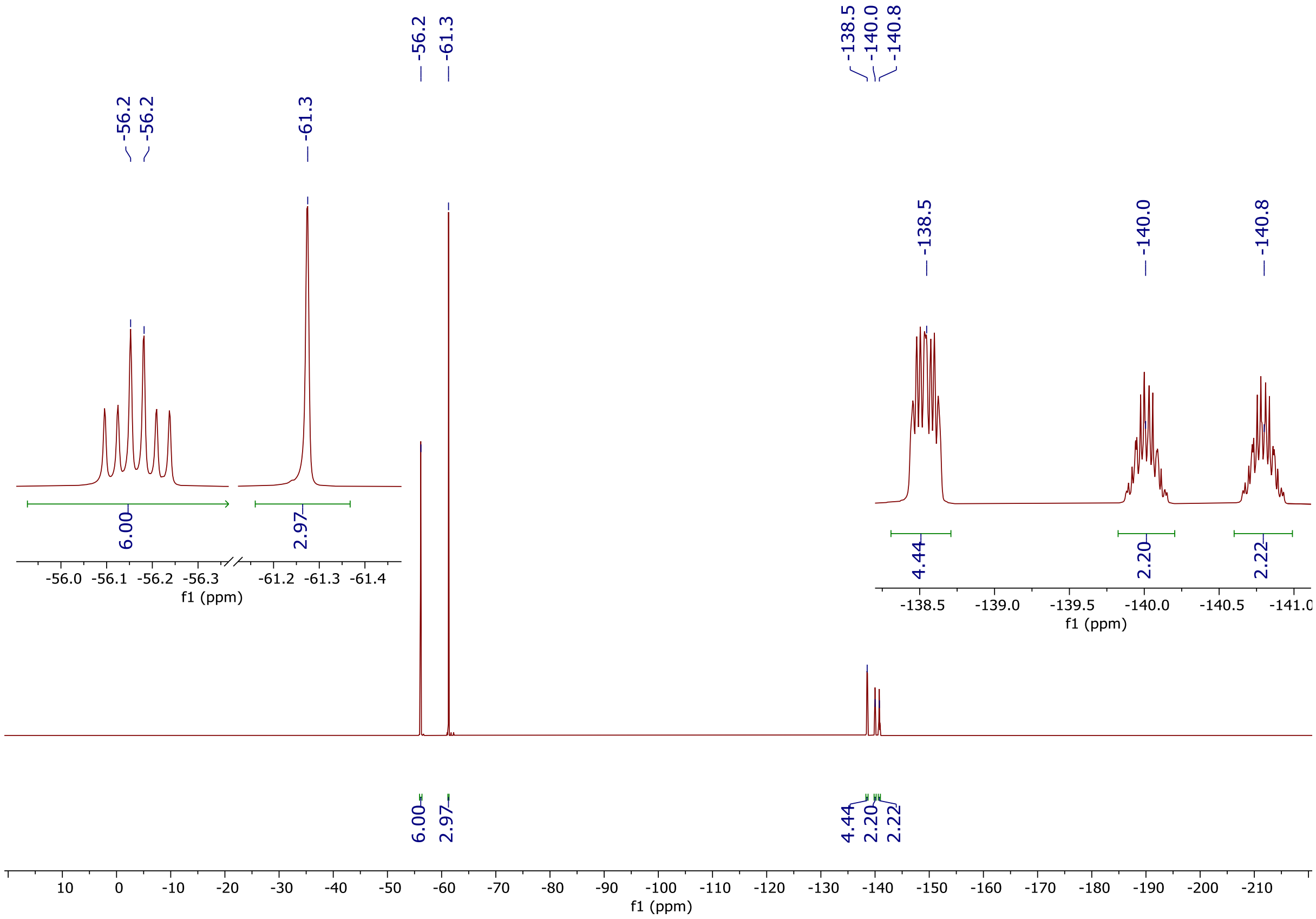

Figure S1.6. ^{19}F NMR spectrum (377 MHz, $CDCl_3$) of **L²**.

1.4 $(L^1)_2Si$ (1-oo)

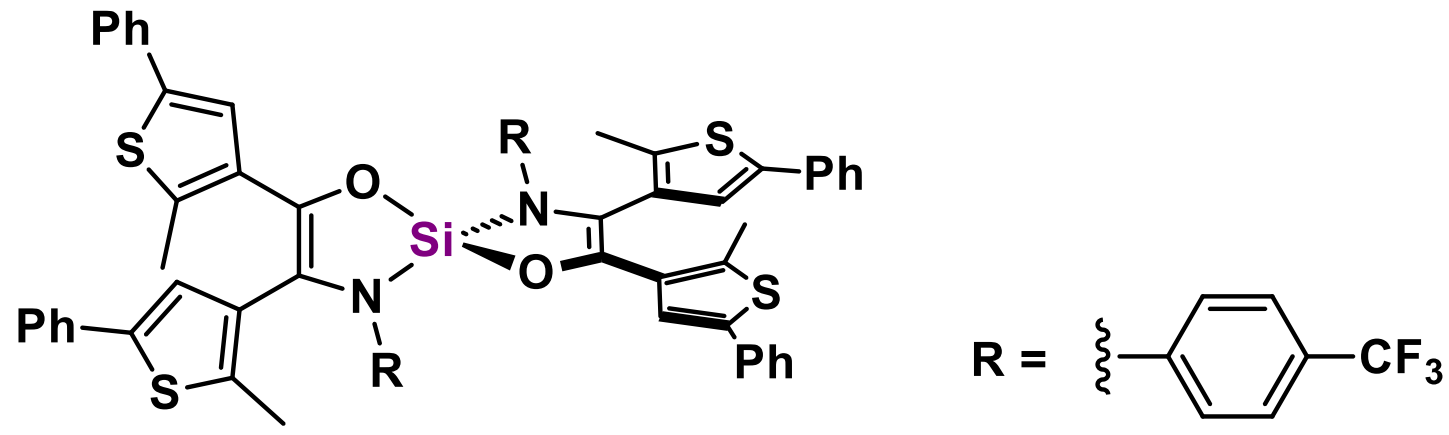

In a thick-walled J-Young ampoule, L^1 (541 mg, 988 µmol, 2.0 eq) and NEt_3 (275 µL, 1.98 mmol, 4.0 eq) were suspended in toluene (20 ml). $SiBr_4$ (61.5 µL, 494 µmol, 1.0 eq) was added and the flask was sealed. The mixture was heated to 130 °C for 36 hours. After cooling to room temperature, the precipitated triethylamine hydrobromide was filtered off, and the filtrate concentrated in vacuo. The resulting oil was triturated with MeCN (10 ml, repeated removal of solvent may be required to induce precipitation), and the resulting suspension filtered. The solid was washed with MeCN (3x2 ml) and subsequently dissolved in a 1:4 mixture of DCM and n-pentane. Storing the solution at -40 °C for a week resulted in the precipitation of a colorless microcrystalline solid, which was filtered off and dried in vacuo (312 mg, 279 µmol, 56%).

^{1}H NMR (338K, 600 MHz, C_6D_6) δ 7.43 (m, 4H, *ortho-C_6**H**$_5$*), 7.30 (m, 4H, *ortho-C_6**H**$_5$*), 7.17 (d, *J* = 8.5 Hz, 4H, *meta-NC_6**H**$_4CF_3$*), 7.12 (s, 2H, *thiophene-C**H***), 7.06 – 7.01 (m, 4H, *meta-C_6**H**$_5$*), 7.01 – 6.98 (m, 4H, *meta-C_6**H**$_5$*), 6.97 – 6.92 (m, 4H, *para-C_6**H**$_5$*), 6.91 (s, 2H, *thiophene-C**H***), 6.88 (d, *J* = 8.4 Hz, 4H, *ortho-NC_6**H**$_4CF_3$*), 2.32 (s, 6H, *C**H**$_3$*), 1.96 (s, 6H, *C**H**$_3$*).

Note: The signal at 7.17 ppm is partially hidden behind the solvent signal.

^{13}C NMR (151 MHz, CD_2Cl_2) δ 144.2 (s, ***C**$_q$*), 141.7 (s, ***C**$_q$*), 140.4 (s, ***C**$_q$*), 138.4 (s, ***C**$_q$*), 137.7 (s, ***C**$_q$*), 134.4 (s, ***C**$_q$*), 134.2 (s, ***C**$_q$*), 134.1 (s, ***C**$_q$*), 130.6 (s, ***C**$_q$*), 129.3 (s, ***C**H, ortho-**C**$_6H_5$*), 129.2 (s, ***C**H, ortho-**C**$_6H_5$*), 127.9 (s, ***C**H, para-**C**$_6H_5$*), 127.7 (s, ***C**H, para-**C**$_6H_5$*), 126.4 (s, ***C**H, meta-N**C**$_6H_4CF_3$*), 125.8 (s, ***C**H, meta-**C**$_6H_5$*), 125.7 (s, ***C**H, meta-**C**$_6H_5$*), 125.2 (q, *J* = 32.9 Hz, ***C**$_q$*, ***C**-CF_3*), 124.6 (d, *J* = 271.5 Hz, ***C**F_3*), 124.0 (br, *thiophene-**C**H*) 122.9 (s, ***C**$_q$*), 122.1 (s, ***C**H, ortho-N**C**$_6H_4CF_3$*), 14.9 (s, ***C**H_3*), 14.3 (s, ***C**H_3*).

Note: The broad signal at 124.0 ppm contains both thiophene CH resonances. One less quaternary carbon signal than expected is observed, and is likely not detected due to line broadening.

^{19}F NMR (377 MHz, C_6D_6) δ -61.8 (s, 6F, *C**F**$_3$*).

^{29}Si NMR (80 MHz, C_6D_6) δ -40.0 (s).

HRMS (MALDI): $[C_{62}H_{44}F_6N_2O_2S_4Si*H_2O]^+$, calcd.: 1136.2065, found: 1136.2083.

UV-vis (benzene): λ_{max} (ε) = 295 nm (87000 $M^{-1}cm^{-1}$).

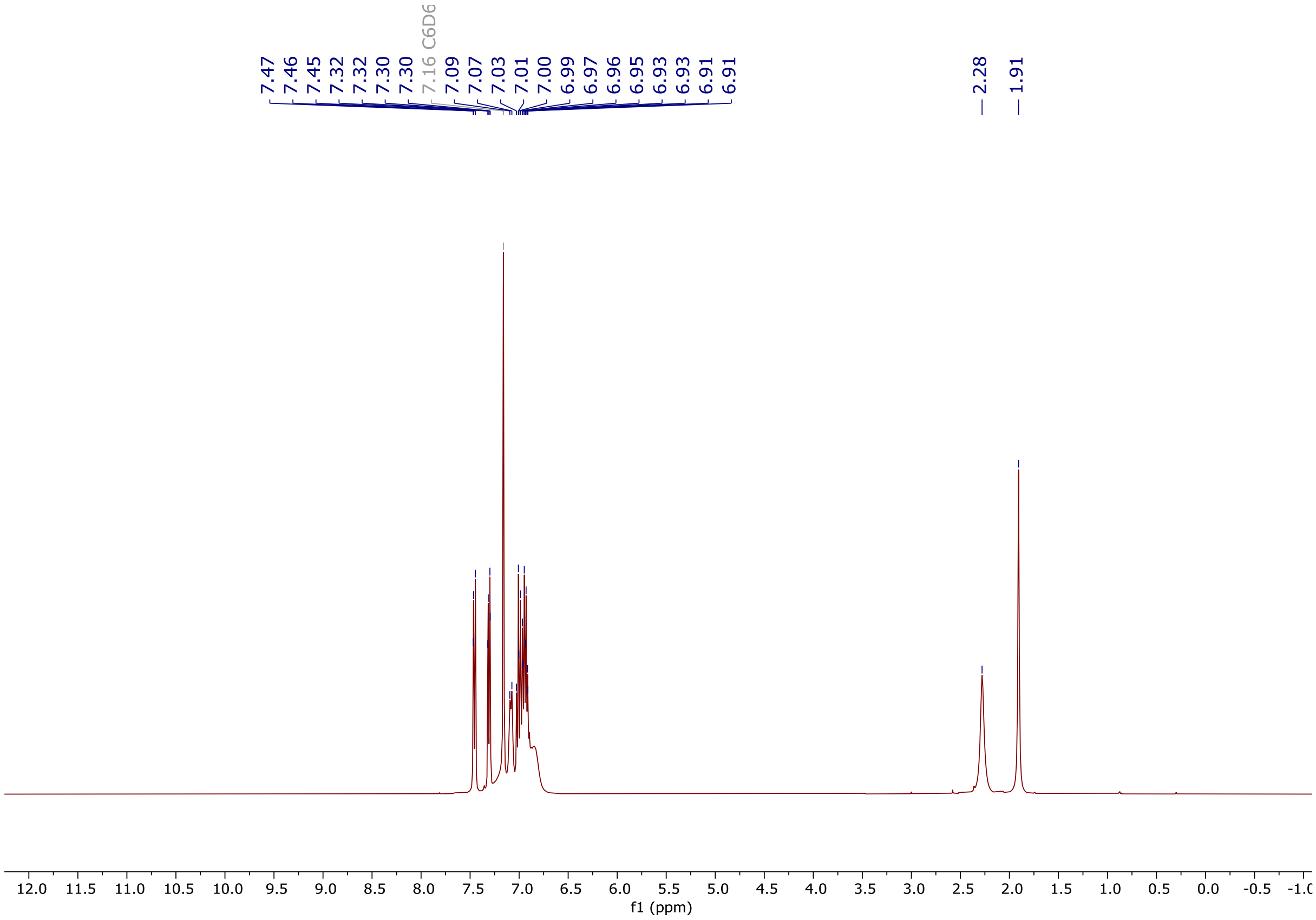

Figure S1.7. ^{1}H NMR spectrum (600 MHz, C_6D_6) of **1** at 298K.

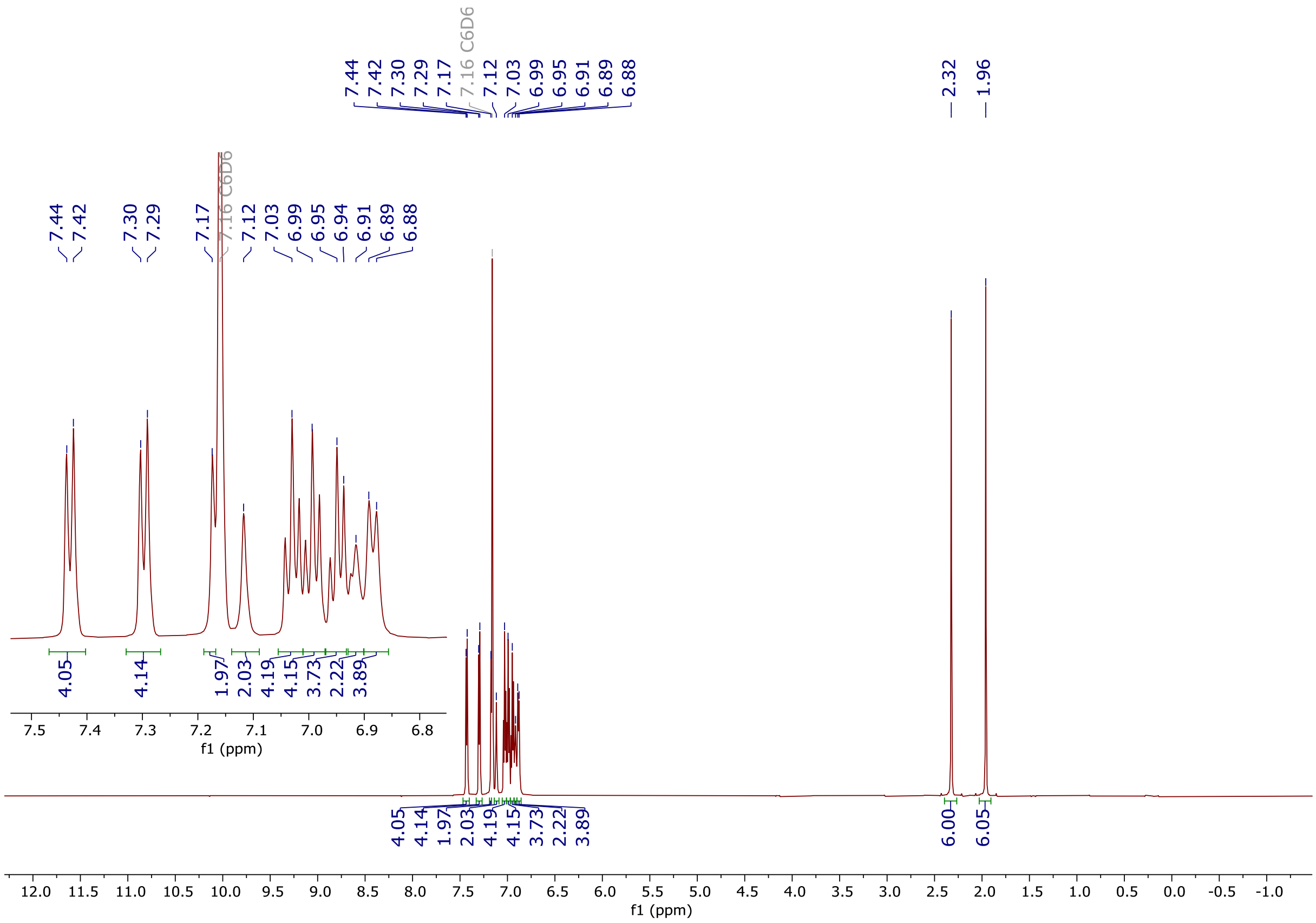

Figure S1.8. ^{1}H NMR spectrum (600 MHz, C_6D_6) of **1** at 328K.

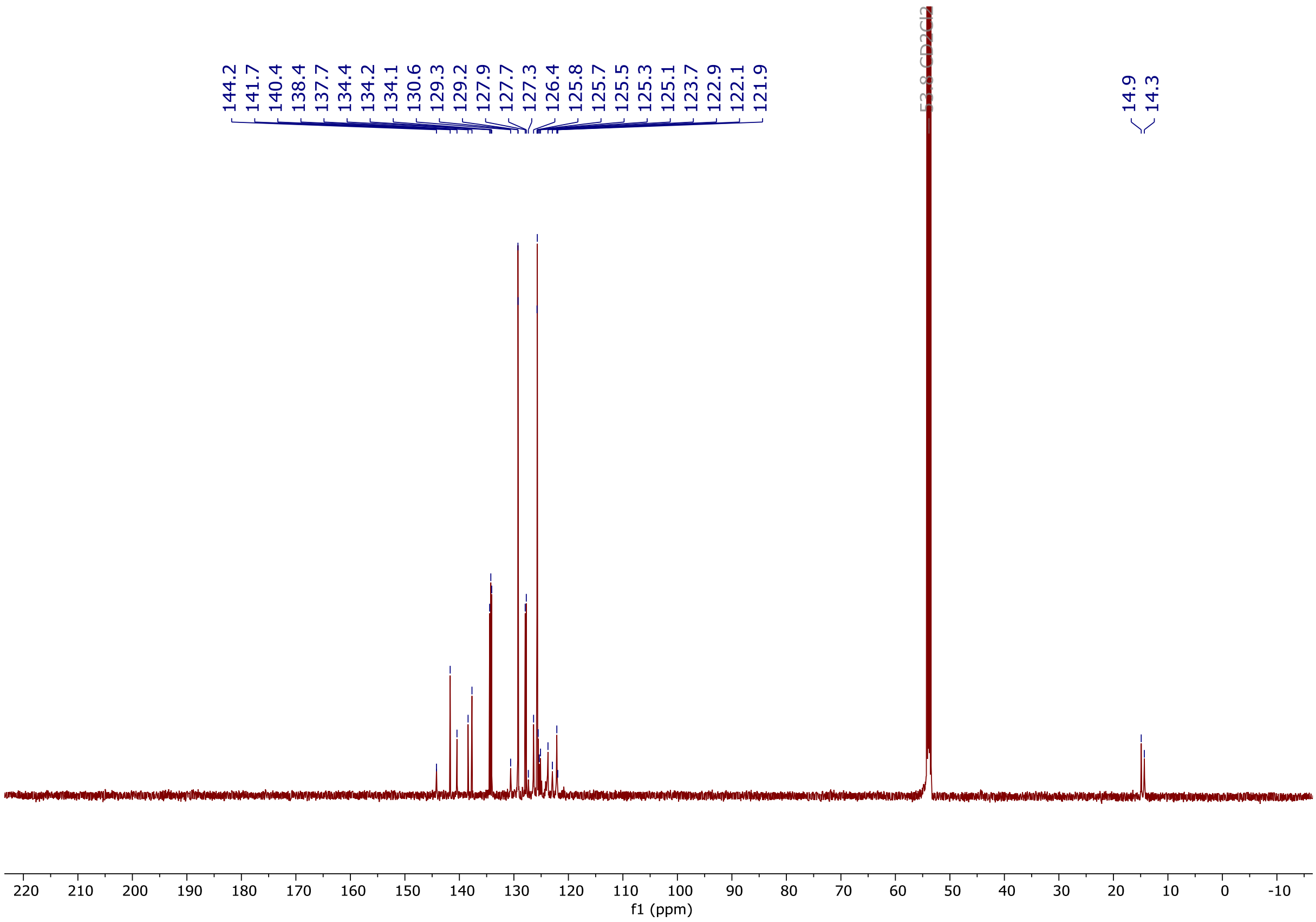

Figure S1.9. ^{13}C NMR spectrum (151 MHz, CD_2Cl_2) of **1**.

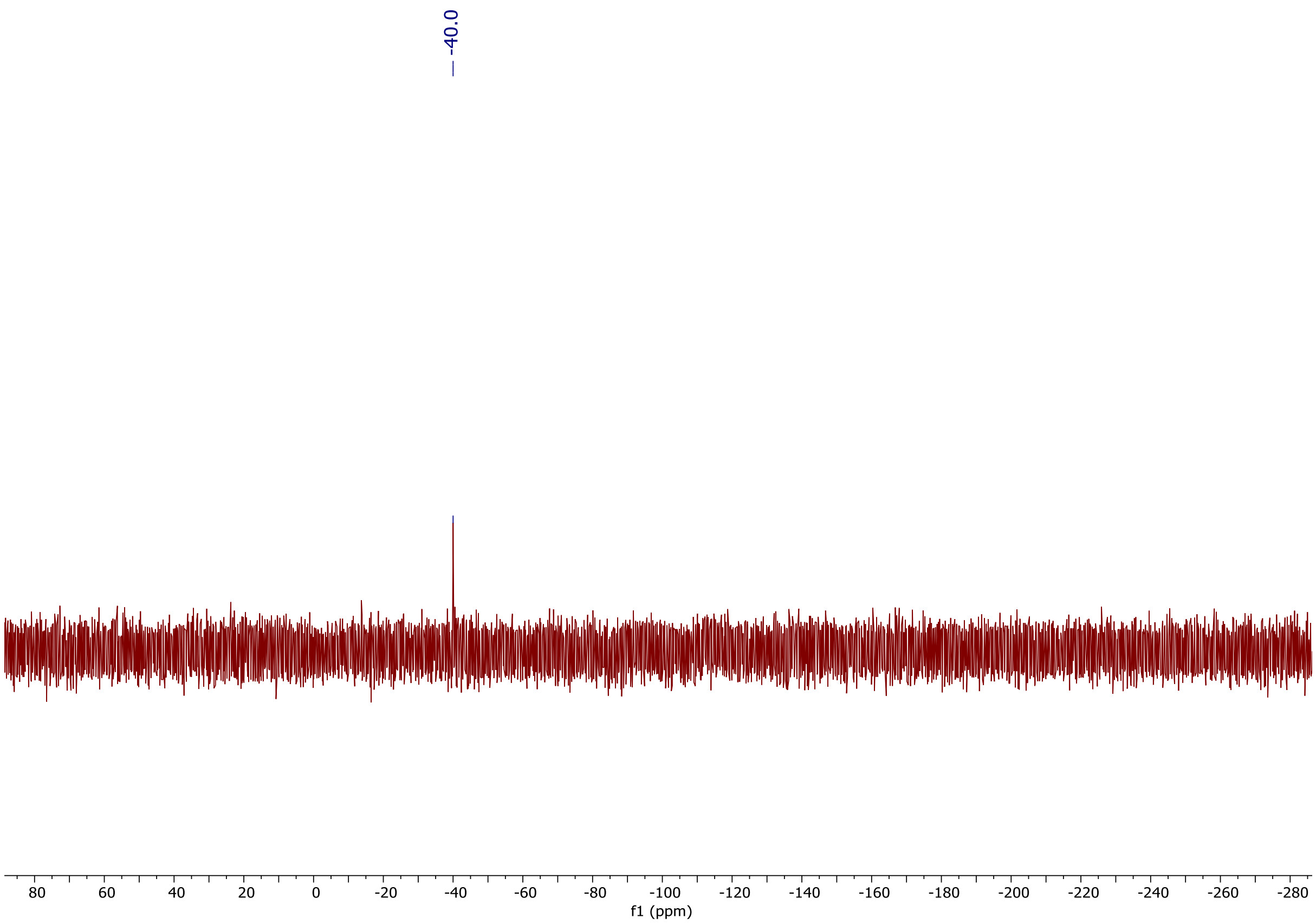

Figure S1.10. ^{29}Si NMR spectrum (80 MHz, C_6D_6) of **1**.

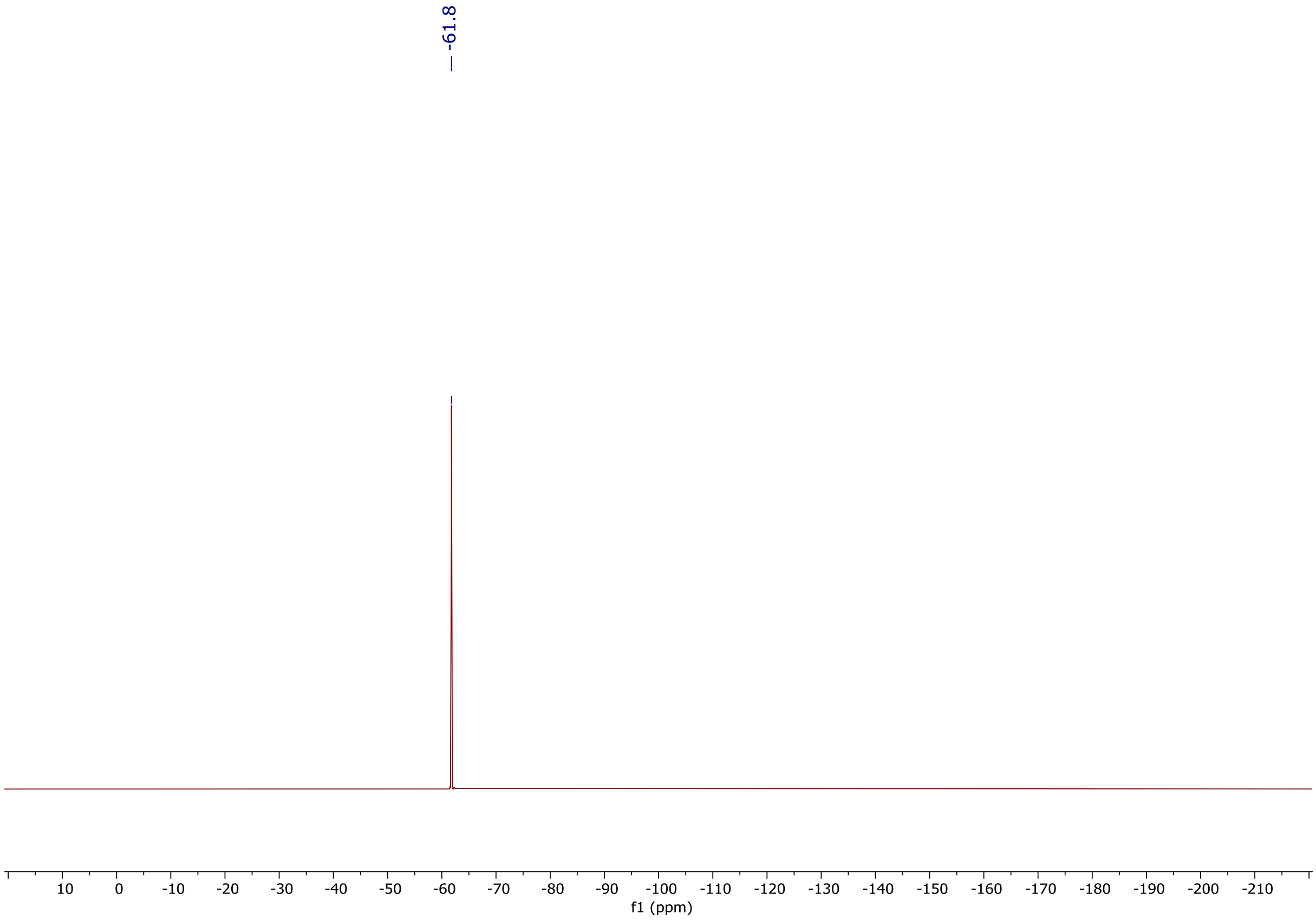

Figure S1.11. ^{19}F NMR spectrum (377 MHz, C_6D_6) of **1**.

1.5 $(L^2)_2Si$ (2-oo)

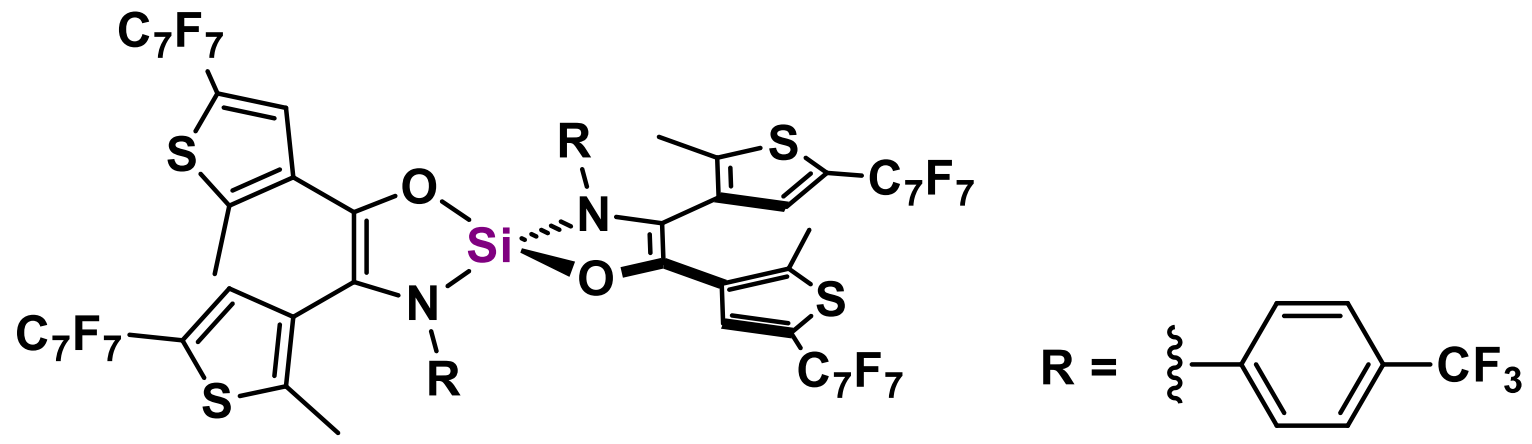

In a thick-walled J-Young ampoule, L^2 (303 mg, 366 µmol, 2.0 eq) and NEt_3 (102 µL, 0.73 mmol, 4.0 eq) were suspended in toluene (20 ml). $SiBr_4$ (27.5 µL, 220 µmol, 1.0 eq) was added and the flask was sealed. The mixture was heated to 130 °C for 16 hours. After cooling to room temperature, the precipitated triethylamine hydrobromide was filtered off, and the filtrate concentrated in vacuo. The resulting oil was triturated with MeCN (3x10 ml), and the resulting suspension filtered. The solid was washed with MeCN (5x5 ml) and subsequently dissolved in a 1:4 mixture of DCM and n-pentane. Storing the solution at -40 °C for 2 weeks resulted in the precipitation of a colorless microcrystalline solid, which was filtered off and dried in vacuo (170 mg, 101 µmol, 55%).

^{1}H NMR (348K, 600 MHz, C_6D_6) δ 7.34 (s, 2H, *thiophene-C**H***), 7.30 (d, *J* = 8.3 Hz, 4H, *meta-NC$_6$**H$_4$**CF$_3$*), 7.15 (s, 2H, *thiophene-C**H***), 6.85 (d, *J* = 8.2 Hz, 4H, *ortho-NC$_6$**H$_4$**CF$_3$*), 2.33 (s, 6H, *C**H$_3$***), 1.98 (s, 6H, *C**H$_3$***).

Note: The signal at 7.15 ppm is partially hidden behind the solvent signal.

^{13}C NMR (101 MHz, CD_2Cl_2) δ 144.9 (dm, *J* = 260 Hz, ***C**F*), 144.0 (dm, *J* = 250 Hz, ***C**F*), 143.2 (t, *J* = 4.4 Hz, ***C**$_q$*),133.6 (s, ***C**$_q$*), 132.8 (br, *thiophene-**C**H*) 132.6 (s, *thiophene-**C**H*), 130.2 (s, ***C**$_q$*), 128.7 (s, ***C**$_q$*), 126.6 (s, ***C**H, ortho-NC$_6$H$_4$CF$_3$*), 126.0 (q, J = 33.7 Hz, ***C**$_q$, para-N**C**$_6$H$_4$CF$_3$*), 124.5 (q, *J* = 271.6 Hz, *NC$_6$H$_4$**C**F$_3$*), 123.2 (s, ***C**$_q$*) 122.9 (s, ***C**$_q$*), 122.7 (s, ***C**H, ortho-N**C**$_6$H$_4$CF$_3$*), 121.3 (q, *J* = 270 Hz, *perfluorotolyl-**C**F$_3$*) 118.5 (t, *J* = 13.9 Hz, ***C**$_q$*), 118.3 (t, *J* = 13.9 Hz, ***C**$_q$*), 108.4 – 106.1 (m, ***C**$_q$, perfluorotolyl-**C**-CF$_3$*), 14.8 (s, ***C**H$_3$*), 14.2 (s, ***C**H$_3$*).

Note: Fluorine coupling and line broadening due to molecular dynamics on the NMR timescale did not allow to account for all carbon atoms. A subset is listed above. Resonances at 144.9, 144.0, 121.3 and 108.4-106.1 ppm are overlapping signals for the two non-equivalent perfluorotoluene substituents. Coupling constants for these resonances are given with low accuracy due to the overlap.

^{19}F NMR (377 MHz, C_6D_6) δ -56.1 (t, *J* = 21.2 Hz, 6F, *perfluorotolyl-C**F$_3$***), -56.2 (t, *J* = 21.2 Hz, 6F, *perfluorotolyl-C**F$_3$***), -62.1 (s, 6F, *NC$_6$H$_4$C**F$_3$***), -139.0 – -139.7 (m, 8F, *C**F***), -140.6 – -141.4 (m, 8F, *C**F***).

^{29}Si NMR (80 MHz, C_6D_6) δ -40.5 (s).

HRMS (MALDI): $[C_{66}H_{24}F_{34}N_2O_2S_4Si{*}H_2O]^+$, calcd.: 1696.0053, found: 1696.0082.

UV-vis (benzene): λ_{max} (ε) = 301 nm (106000 $M^{-1}cm^{-1}$).

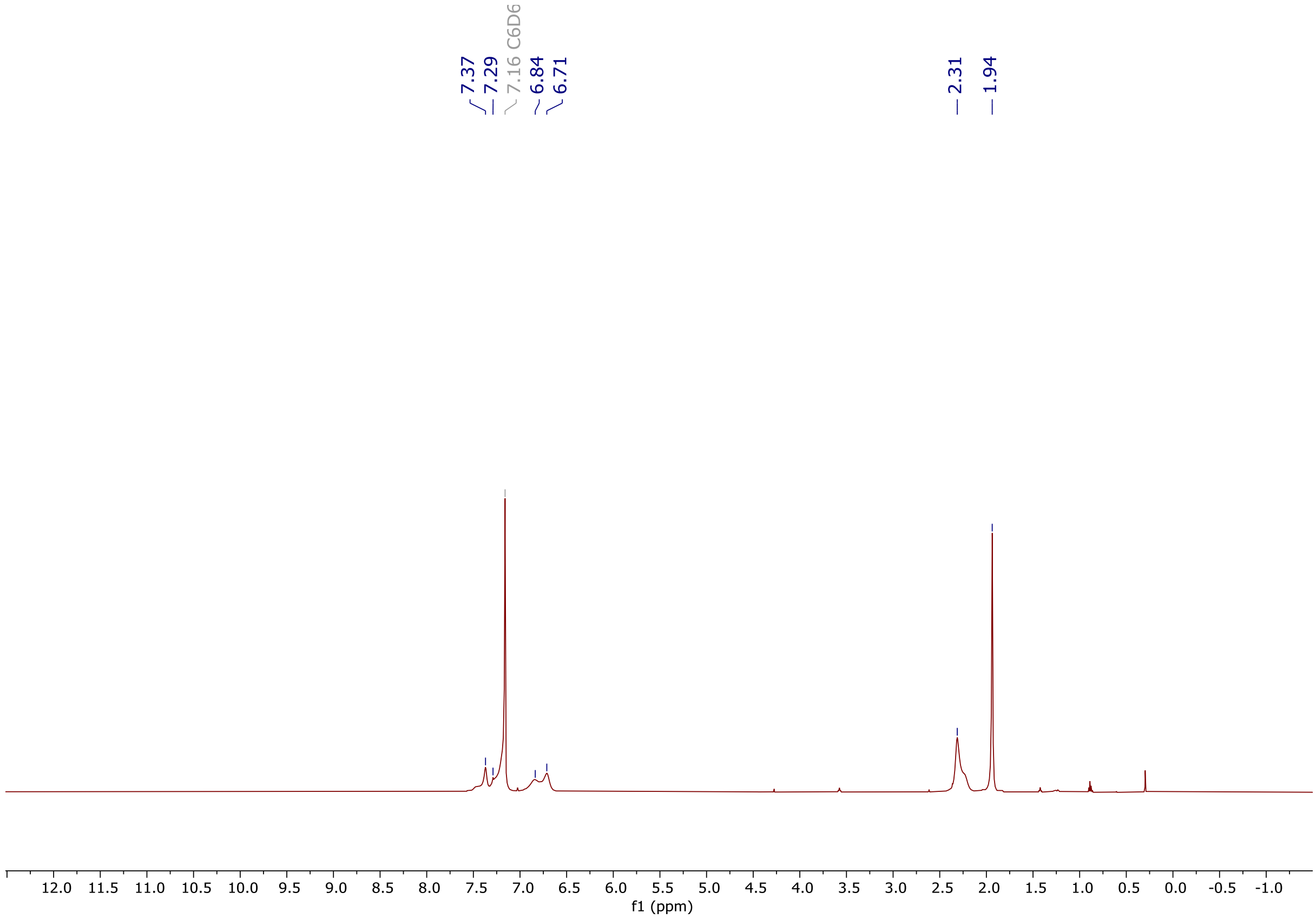

Figure S1.12. ^{1}H NMR spectrum (600 MHz, C_6D_6) of **2** at 298K.

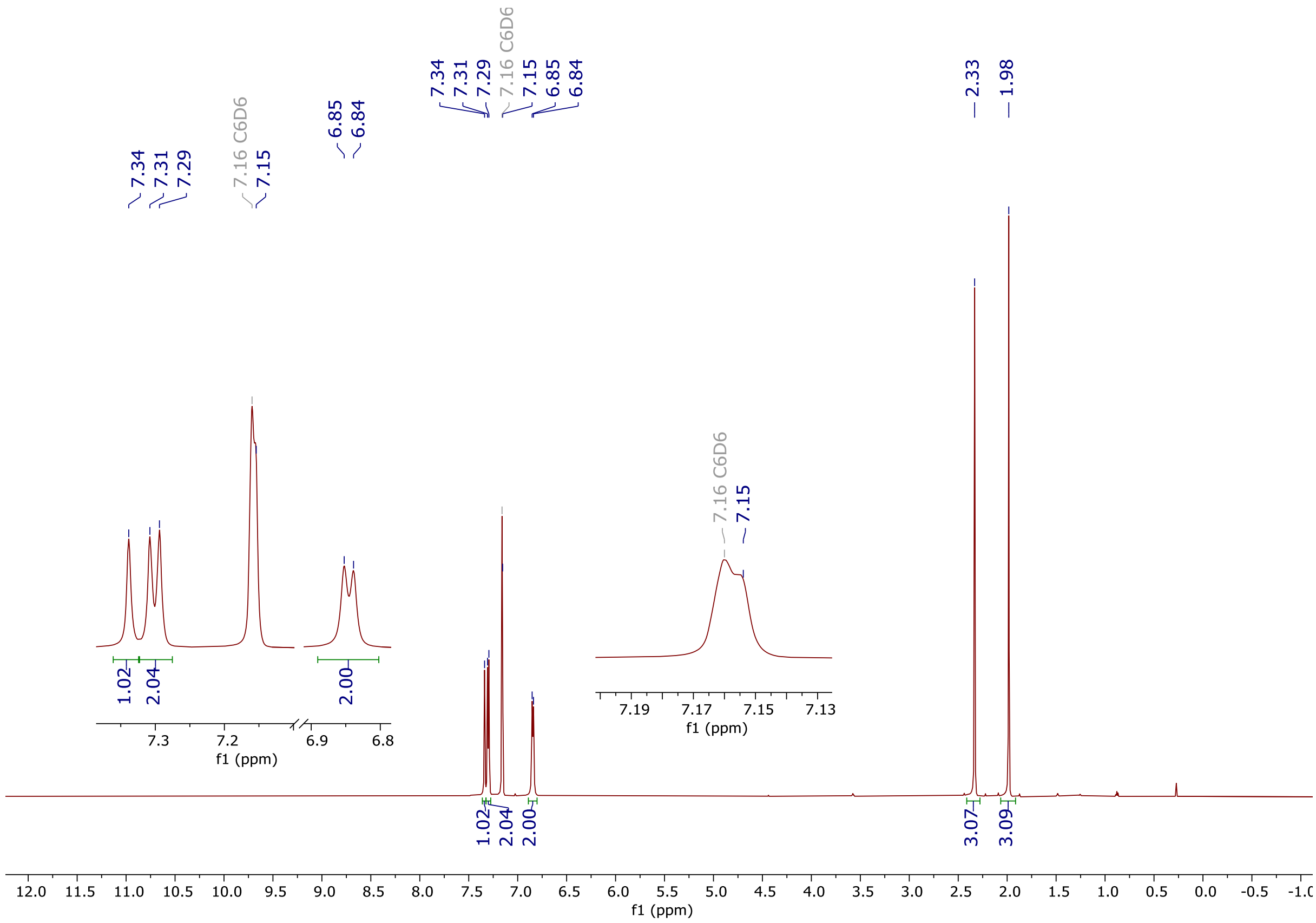

Figure S1.13. ^{1}H NMR spectrum (600 MHz, C_6D_6) of **2** at 348K.

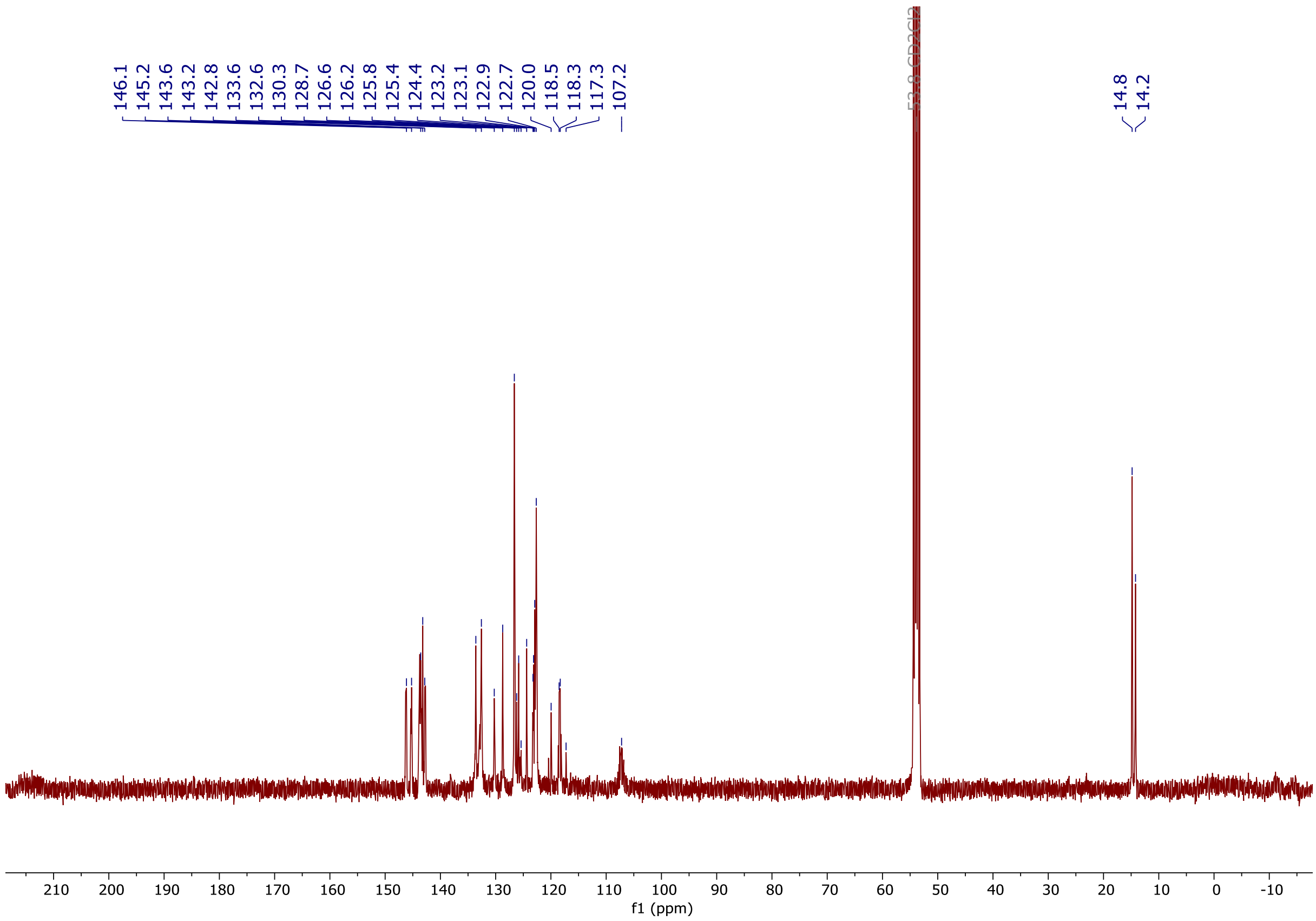

Figure S1.14. ^{13}C NMR spectrum (101 MHz, CD_2Cl_2) of **2**.

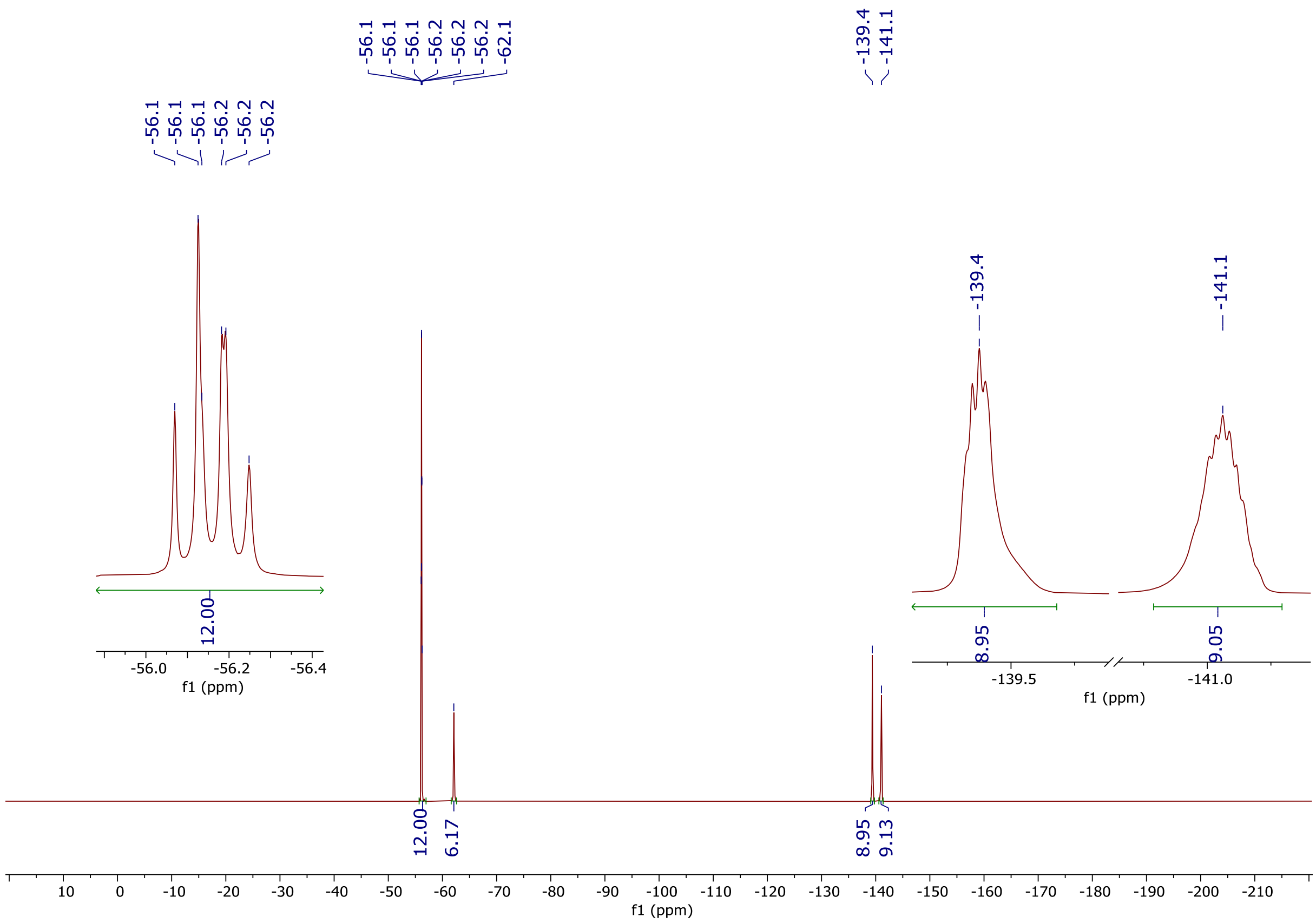

Figure S1.15. ^{19}F NMR spectrum (377 MHz, C_6D_6) of **2**.

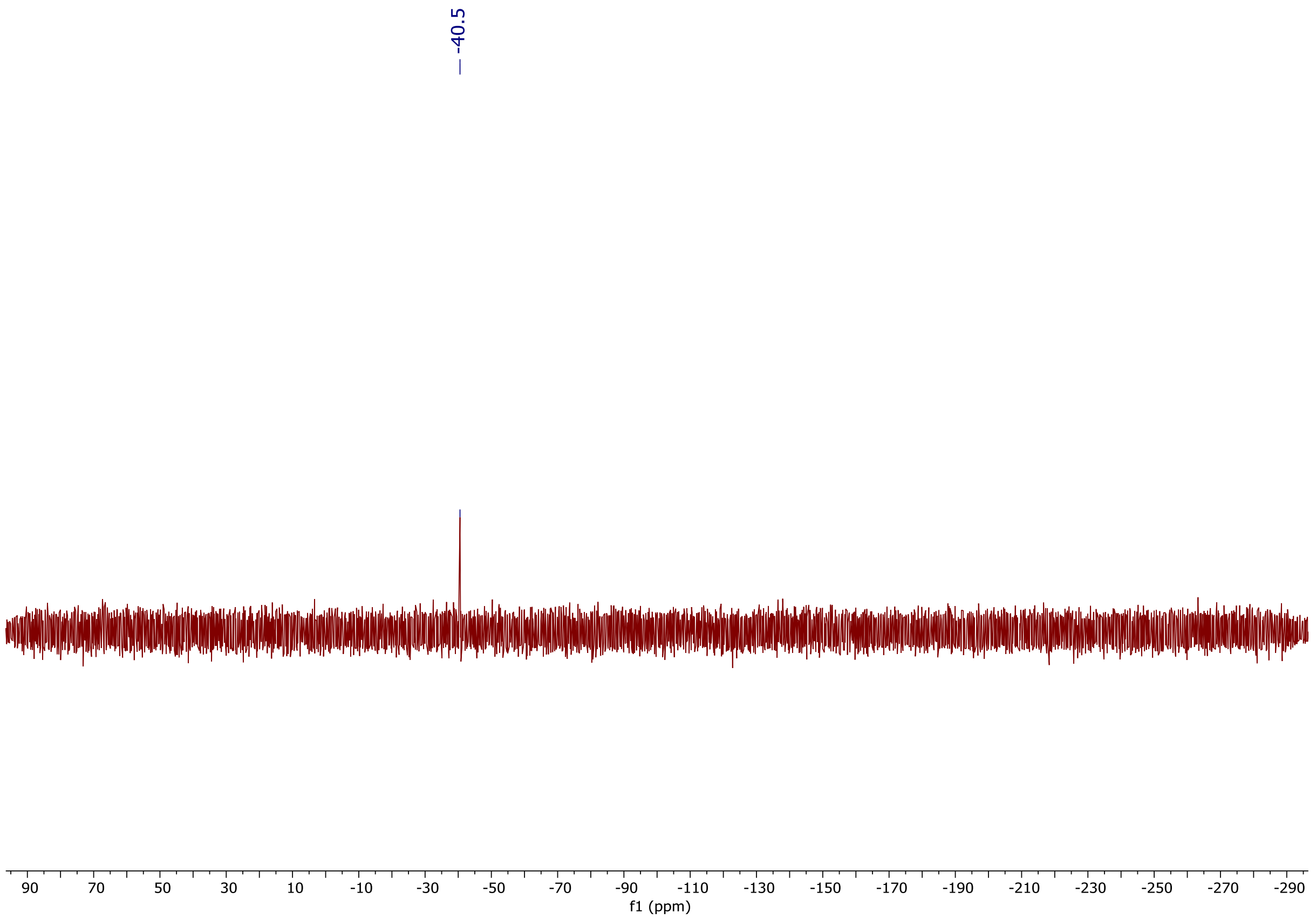

Figure S1.16. ^{29}Si NMR spectrum (80 MHz, C_6D_6) of **2.**

2 UV-vis spectroscopy

All UV–vis spectra were recorded on a JASCO V-570 UV/VIS/NIR spectrophotometer. Data analysis and plotting was done using OriginLab 2025. For UV irradiation, a MAX-303 light source (Asahi Spectra) was used, consisting of a 300 W xenon arc lamp coupled to a mirror module (250–385 nm) and a 10 nm band-pass filter centered at 300 nm. Visible-light irradiation was performed using commercial white LEDs.

2.1 Extinction Coefficients

1

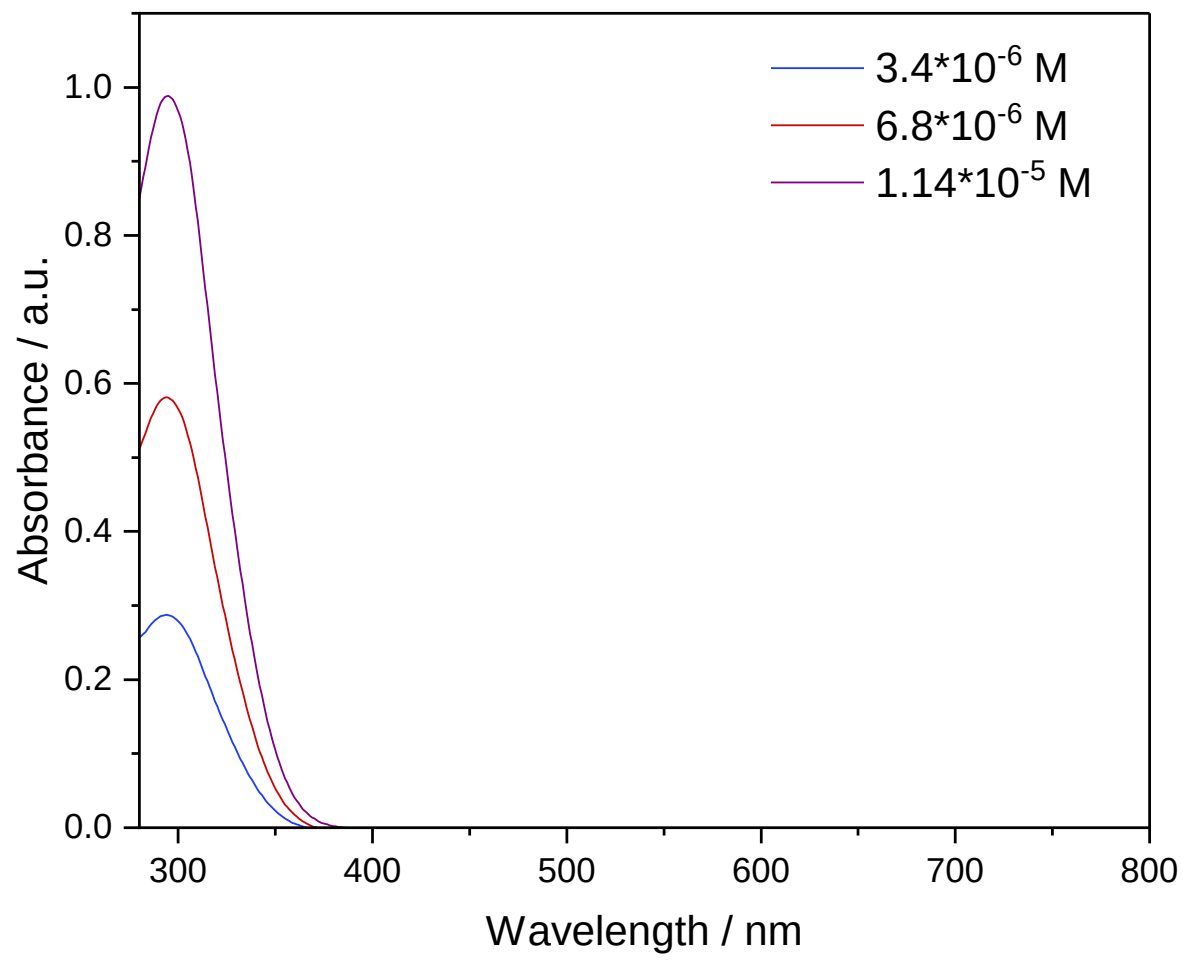

Figure S2.1. UV-vis spectra of **1** in benzene ($3.4*10^{-6}$ M, $6.8*10^{-6}$ M, $1.14*10^{-5}$ M). l = 1 cm.

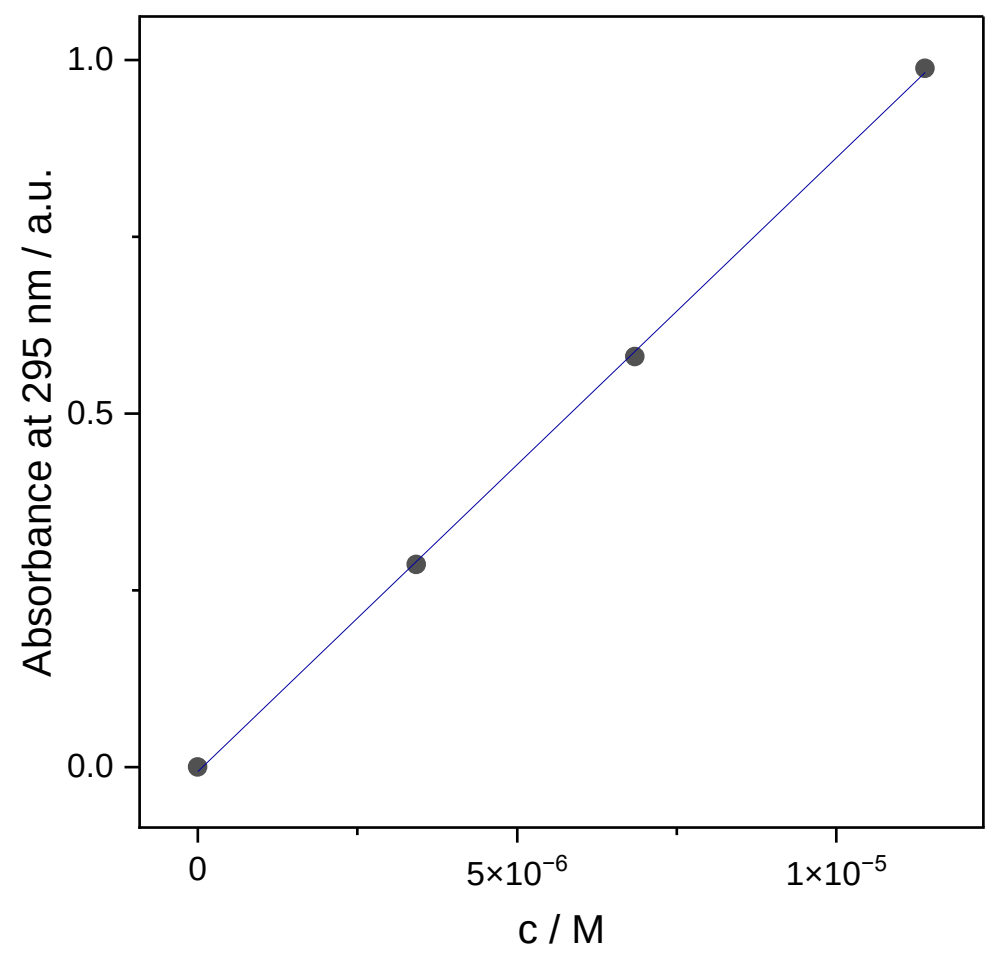

Figure S2.2. Linear Fit for the extinction of **1**.
y = 86808x-0.00614. R^2 =0.9996.

2

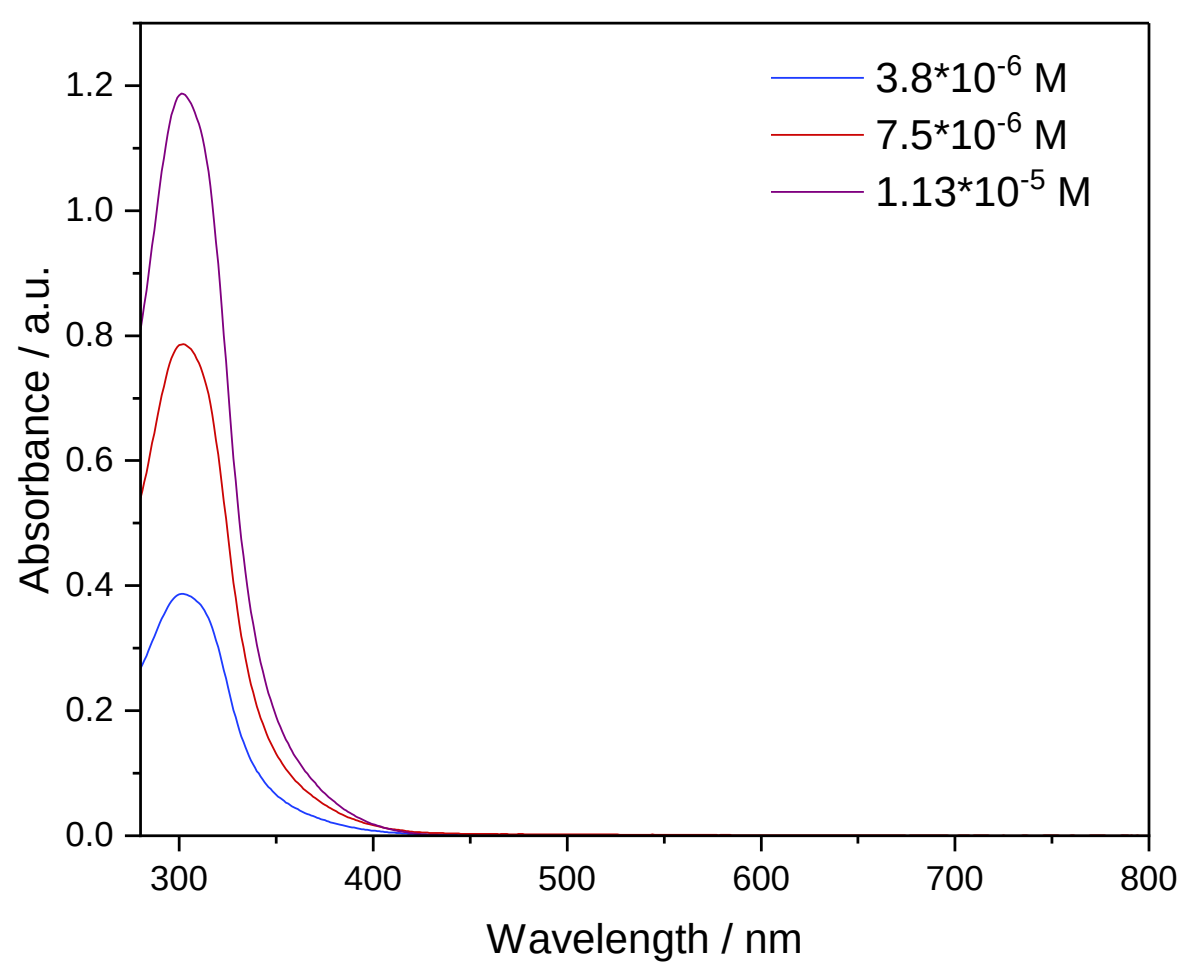

Figure S2.3. UV-vis spectra of **2** in benzene ($3.8*10^{-6}$ M, $7.5*10^{-6}$ M, $1.13*10^{-5}$ M). l = 1 cm.

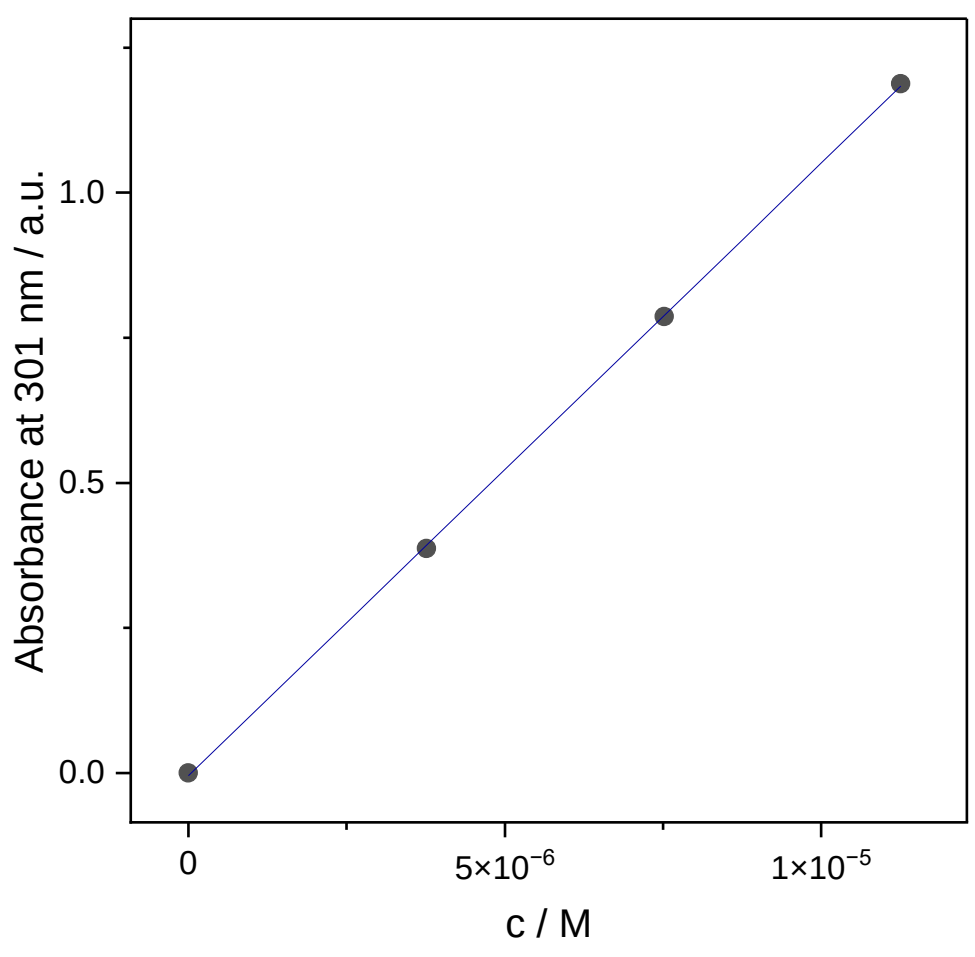

Figure S2.4. Linear Fit for the extinction of **1**.
y = 105558x-0.00484. R^2 =0.9998.

1: $\lambda_{max} = 295\ nm,\ \ \varepsilon = 87000\ M^{-1}cm^{-1}$

2: $\lambda_{max} = 301\ nm,\ \ \varepsilon = 106000\ M^{-1}cm^{-1}$

2.2 Photocyclization and Photocycloreversion

1

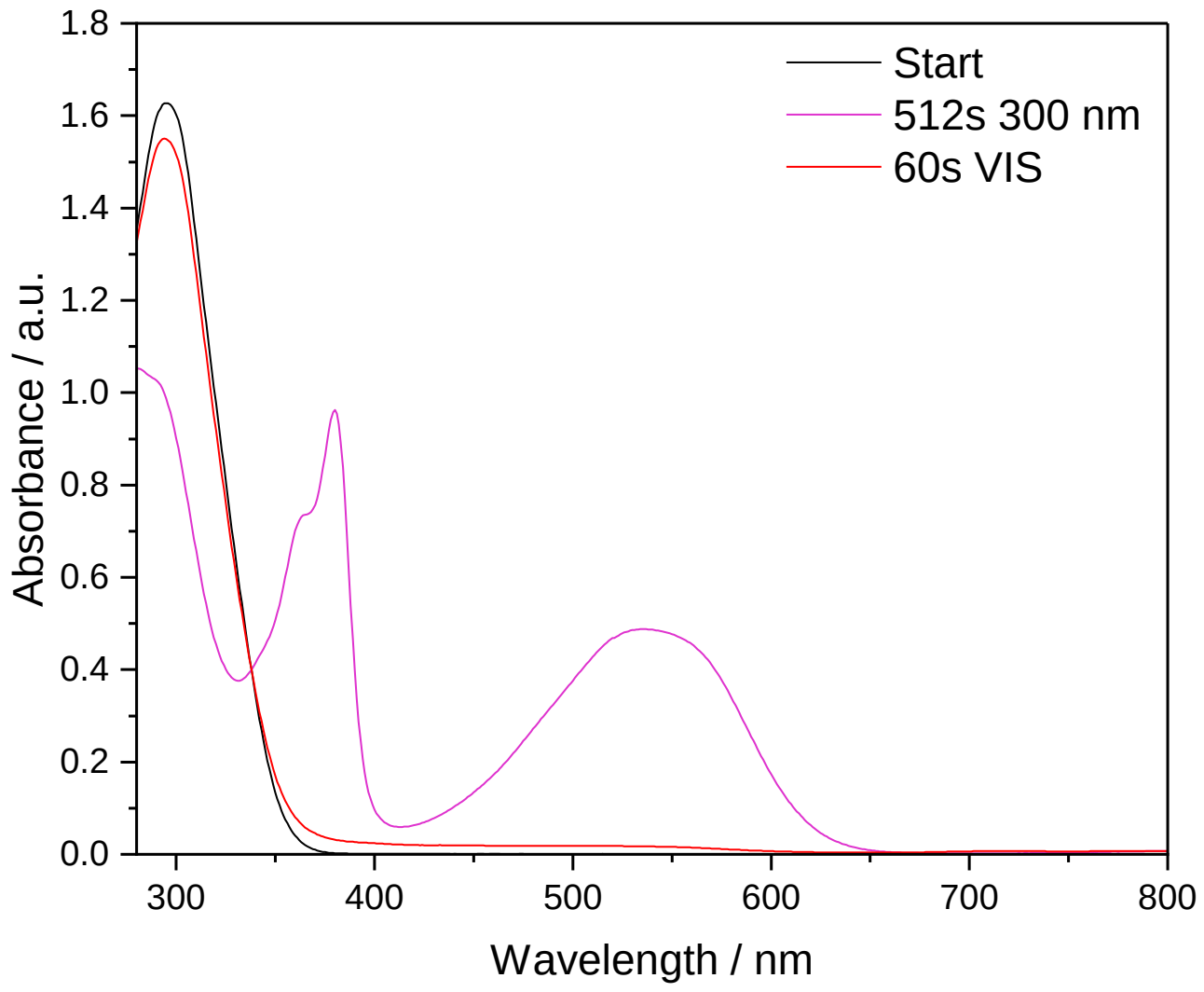

Figure S2.5. UV-vis spectra of **1** (1.9*10^{-5} M, benzene) before irradiation, after 512 seconds at 300 nm and after 60s broadband VIS irradiation.

2

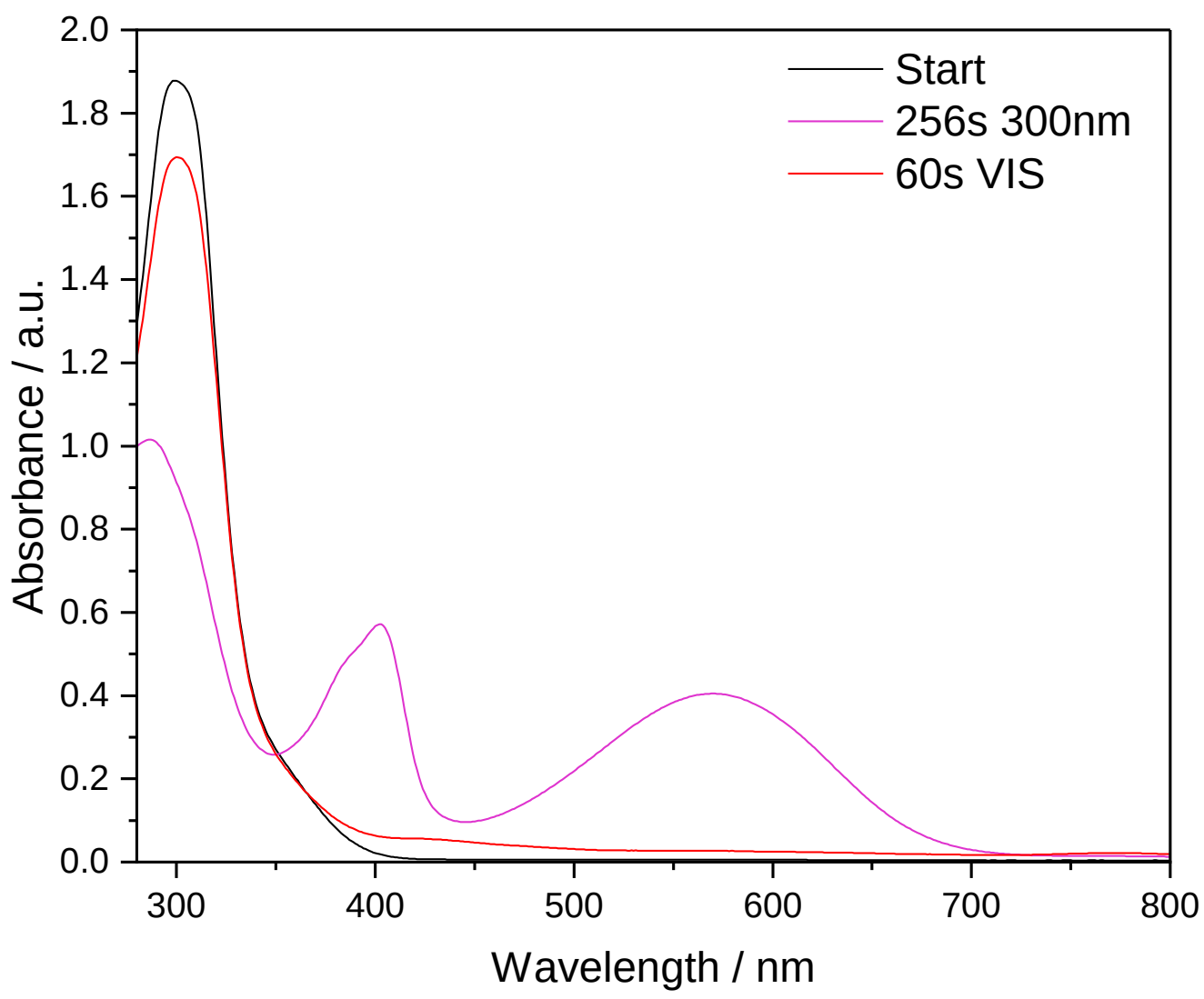

Figure S2.6. UV-vis spectra of **2** (1.8*10^{-5} M, benzene) before irradiation, after 256 seconds at 300 nm and after 60s broadband VIS irradiation.

3 Computational Section

3.1 General Information

All quantum chemical calculations were carried out with the Orca 6.0.1 program package.[2] High-performance computing resources were provided by JUSTUS2 (Ulm University) through the Baden-Württemberg High Performance Computing (bwHPC) program. For all calculations, the RI approximation for the coulomb integral (RIJCOSX) together with the corresponding auxiliary basis sets were used.[3] Geometries were optimized with the PBEh-3c composite method, and subsequent vibrational frequency analyses confirmed the structures as minima by the absence of imaginary modes. All single point calculations were carried out at the DSD-BLYP(D3BJ)/def2-TZVPP[4] level of theory. Solvent corrected energies were obtained with the SMD solvent model and DCM or benzene as solvent.[5] Gibbs free energy values were derived from frequency calculations on the PBEh-3c[6] level of theory using the rigid rotor harmonic oscillator (RRHO) approximation.[7] Time-dependant calculations with the Tamm-Dancoff approximation (TDA)[8] were carried out with the long-range corrected hybrid functional ωB97X-D3,[9] the def2-TZVP basis set, and benzene solvation correction with the SMD solvent model. The first 50 electronic singlet transitions were calculated. Natural transition orbitals were generated for the first 15 excitations. Molecular orbitals and natural transition orbitals were visualized with IboView v20211019-RevA. For the thermal ring-opening transition states, the ωB97X-D4 method was used and unrestricted DFT was enabled. Broken symmetry solutions were obtained by the "brokensym 1,1" keyword in the %scf block, with the triplet wavefunction as the initial guess.

Open Ligands were always optimized in the antiparallel conformation to obtain consistent data.

Inputs

Geometry Optimsation

```
! PBEh-3c RIJCOSX AutoAux TightSCF defgrid3 TightOPT FREQ
```

Single Point Calculation (benzene)

```
! DSD-BLYP D3BJ def2-TZVPP RIJCOSX AutoAux VeryTightSCF

%cpcm
smd true
SMDsolvent "benzene"
End
```

OptTS (brokensym)

```
! UKS wB97X-D3 def2-TZVP RIJCOSX AutoAux OptTS freq

%scf
Brokensym 1,1
Maxiter 500
End
```

TD-DFT (benzene)

```
! wB97X-D3 def2-TZVP RIJCOSX AutoAux TightSCF

%cpcm
smd true
SMDsolvent "benzene"
End

%tddft
  maxdim 5
  nroots 50
  DoNTO true
  NTOstates 1,2,3,4,5,6,7,8,9,10,11,12,13,14,15
end
```

3.2 Fluoride Ion Affinities

Fluoride ion affinities (FIA) were calculated utilizing isodesmic reactions with the TMS/TMSF anchor system as proposed by *Krossing*[10] and *Greb*.[11] The FIA values are given as the negative binding enthalpy (FIA= -ΔH(LA + F →LA-F)).

Reaction 1

LA + TMSF → TMS^+ + $LA\text{-}F^-$ ΔH (DSD-BLYP(D3BJ)/def2-TZVPP//PBEh-3c)

Reaction 2

TMSF → TMS^+ + F^- (ΔH = 952.5 kJ/mol[11b])

FIA (vacuum) = ΔH(reaction 2) – ΔH(reaction 1)

FIA (solv) = FIA(vacuum)-$\Delta H_{solv}(LA\text{-}F^-)$ + $\Delta H_{solv}(LA)$ + $\Delta H_{solv}(F^-)$

Table S3.1. Thermodynamic data for the calculation of fluoride ion affinities for **1**, **2**, and reference Lewis acids. DSD-BLPYP(D3BJ)/def2-TZVPP//PBEh-3c. Solvent correction with SMD(DCM).

	Charge/ Multiplicity	Total Correction [kJ/mol]	Entropy Term [kJ/mol]	SP Energy (vacuum) [kJ/mol]	Enthalpy (vacuum) [kJ/mol]	Enthalpy Correction (DCM) [kJ/mol]	Enthalpy (DCM) [kJ/mol]
**2_cc**	0/1	2408.48	554.99	-21165709.27	-21163298.31	-144.11	-21163442.42
**2_cc_F**	-1/1	2415.58	553.90	-21428280.72	-21425862.67	-185.64	-21426048.31
**2_co**	0/1	2407.00	563.85	-21165771.45	-21163361.97	-145.31	-21163507.28
**2_co_F**	-1/1	2413.85	565.19	-21428311.48	-21425895.16	-195.58	-21426090.74
**2_oo**	0/1	2405.48	572.61	-21165832.17	-21163424.21	-146.32	-21163570.53
**2_oo_F**	-1/1	2412.24	569.24	-21428340.02	-21425925.30	-201.64	-21426126.94
**1_cc**	0/1	2630.40	404.01	-13460673.24	-13458040.36	-178.40	-13458218.76
**1_cc_F**	-1/1	2638.22	409.05	-13723163.20	-13720522.50	-264.16	-13720786.66
**1_co**	0/1	2628.67	412.54	-13460722.61	-13458091.46	-179.83	-13458271.29
**1_co_F**	-1/1	2635.69	418.13	-13723191.14	-13720552.97	-271.59	-13720824.56
**1_oo**	0/1	2626.87	420.64	-13460770.56	-13458141.21	-181.24	-13458322.45
**1_oo_F**	-1/1	2634.23	421.61	-13723216.05	-13720579.34	-263.30	-13720842.64
**SbF_5**	0/1	49.58	106.38	-1940340.10	-1940288.04	-34.00	-1940322.04
**SbF_5_F**	-1/1	57.88	111.22	-2202869.97	-2202809.61	-187.50	-2202997.11
**BCF**	0/1	496.90	221.61	-5794610.36	-5794110.98	-25.80	-5794136.78
**BCF_F**	-1/1	503.87	226.90	-6057083.60	-6056577.25	-134.46	-6056711.71
**$Si(^Fam^Fph)_2$**	0/1	697.01	273.14	-8557152.51	-8556453.02	-38.83	-8556491.85
**$Si(^Fam^Fph)_2$_F**	-1/1	704.65	281.65	-8819698.74	-8818991.62	-124.93	-8819116.55
**TMS**	1/1	315.05	104.27	-1072919.90	-1072602.37		
**TMSF**	0/1	328.84	106.99	-1335913.99	-1335582.67		
**F**	-1/1					-347.86	

Table S3.2. Fluoride ion affinities of **1**, **2**, and reference Lewis acids.

Compound	FIA (vacuum) [kJ/mol]	FIA(DCM) [kJ/mol]
**1-oo**	410	145
**1-oc**	434	178
**1-cc**	454	192
**2-oo**	473	181
**2-oc**	505	208
**2-cc**	537	230
**BCF**	438	199
**Si(^{F}am^{F}ph)$_2$**	511	249
**SbF_5**	494	299

3.3 Lewis Base Affinities

Table S3.3. Thermodynamic data for the calculation of Et_3PO affinities of **1** and **2**, and pyridine affinities for **1**. DSD-BLPYP(D3BJ)/def2-TZVPP//PBEh-3c. Solvent correction with SMD(benzene). Charge (0) and multiplicity (1) identical for all species.

	Total Correction [kJ/mol]	Entropy Term [kJ/mol]	SP Energy (Benzene) [kJ/mol]	Enthalpy (Benzene) [kJ/mol]	Gibbs Free Energy (Benzene) [kJ/mol]
**2_cc**	2408.48	554.99	-21165822.01	-21163411.05	-21163966.04
**2_cc_Et_3PO**	2998.56	609.20	-22882597.55	-22879596.50	-22880205.70
**2_co**	2407.00	563.85	-21165885.60	-21163476.12	-21164039.97
**2_co_Et_3PO**	2996.77	619.62	-22882639.24	-22879639.99	-22880259.61
**2_oo**	2405.48	572.61	-21165947.52	-21163539.56	-21164112.17
**2_oo_Et_3PO**	2994.90	625.78	-22882684.62	-22879687.25	-22880313.03
**1_cc**	2630.40	404.01	-13460829.39	-13458196.51	-13458600.53
**1_cc_Et_3PO**	3221.53	462.23	-15177566.42	-15174342.41	-15174804.64
**1_cc_pyridine**	2892.83	431.29	-14112191.07	-14109295.76	-14109727.04
**1_oc**	2628.67	412.54	-13460880.09	-13458248.94	-13458661.48
**1_oc_Et_3PO**	3219.72	467.62	-15177603.01	-15174380.82	-15174848.43
**1_oc_pyridine**	2890.89	435.51	-14112230.86	-14109337.49	-14109773.00
**1_oo**	2626.87	420.64	-13460929.53	-13458300.18	-13458720.81
**1_oo_Et_3PO**	3217.68	477.67	-15177631.85	-15174411.69	-15174889.36
**1_oo_pyridine**	2888.97	443.50	-14112272.23	-14109380.79	-14109824.29
**Pyridine**	251.65	83.49	-651283.69	-651029.56	-651113.05
**Et_3PO**	580.87	126.26	-1716628.83	-1716045.47	-1716171.73

Table S3.4. Lewis Base Affinities for **1** and **2**.

	ΔH (Et_3PO binding) [kJ/mol]	ΔG (Et_3PO binding) [kJ/mol]	ΔH (pyridine binding) [kJ/mol]	ΔG (pyridine binding) [kJ/mol]
**1-oo**	-66.0	3.2	-51.0	9.6
**1-oc**	-86.4	-15.2	-59.0	1.5
**1-cc**	-100.4	-32.4	-69.7	-13.5
**2-oo**	-102.2	-29.1		
**2-oc**	-118.4	-47.9		
**2-cc**	-140.0	-67.9		

3.4 Thermal Ring-Opening

Table S3.5. Thermodynamic data for the calculation of thermal ring-opening barriers. ωB97X-D4/def2-TZVPP/SMD(C_6H_6)//wB97X-D4/def2-TZVP.

	⟨S²⟩	Total Correction [kJ/mol]	Entropy Term [kJ/mol]	SP Energy (Benzene) [kJ/mol]	Enthalpy (Benzene) [kJ/mol]	Gibbs Free Energy (Benzene) [kJ/mol]
**2_cc**	0	2360.58	560.50	-21189246.68	-21186883.62	-21187444.12
**2_cc_to_oc_TS**	1.02	2348.14	564.26	-21189095.18	-21186744.56	-21187308.82
**2_oc**	0	2359.10	571.83	-21189301.40	-21186939.82	-21187511.65
**2_oc_to_oo_TS**	1.03	2343.79	568.85	-21189148.80	-21186802.53	-21187371.38
**2_oo**	0	2358.35	577.43	-21189354.86	-21186994.03	-21187571.46
**1_cc**	0	2578.53	406.17	-13477377.52	-13474796.51	-13475202.68
**1_cc_to_oc_TS**	1.05	2565.48	414.61	-13477218.84	-13474650.88	-13475065.49
**1_oc**	0	2576.72	417.33	-13477420.68	-13474841.47	-13475258.80
**1_oc_to_oo_TS**	1.05	2563.66	423.00	-13477259.60	-13474693.46	-13475116.46
**1_oo**	0	2574.45	429.40	-13477461.70	-13474884.77	-13475314.17

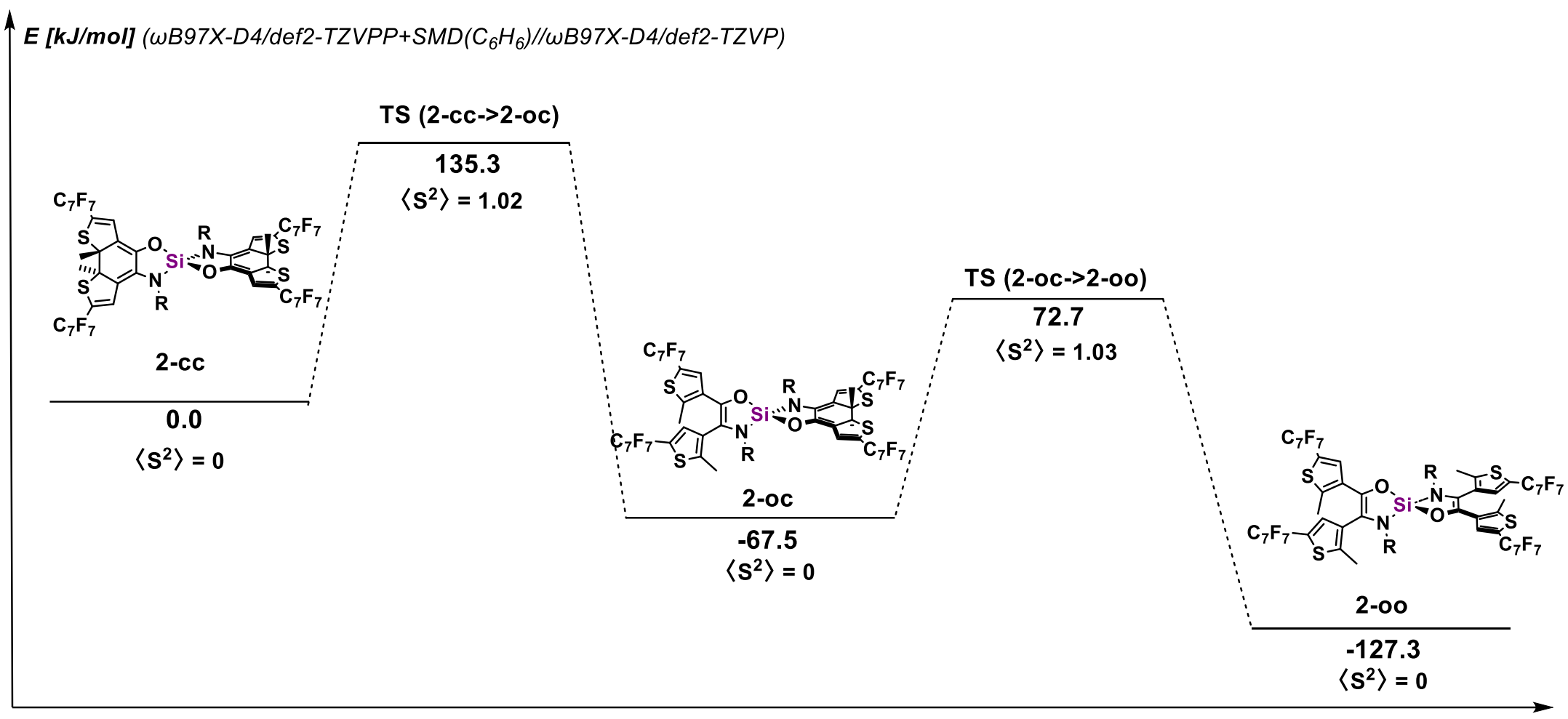

Figure S3.1. Energy profile for the thermal ring-opening of **2-cc** via **2-oc** to **2-oo**.

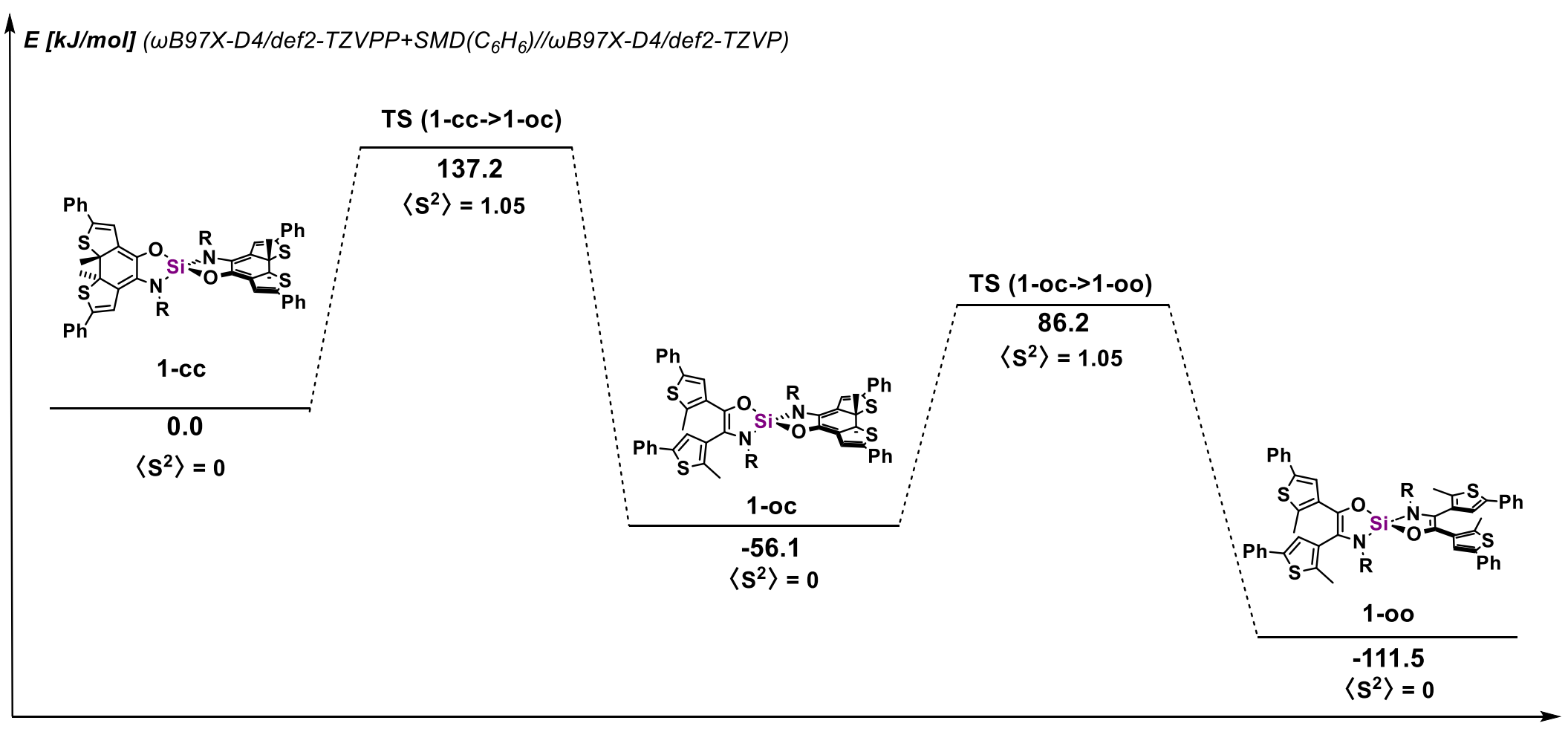

Figure S3.2. Energy profile for the thermal ring-opening of **1-cc** via **1-oc** to **1-oo**.

3.5 TD-DFT

In the following section, low energy excitations are given. In cases with more complicated orbital transitions, natural transition orbitals are displayed.

1-oo

Table S3.6. Low energy transitions of **1-oo**. Method: ωB97X-D3/def2-TZVP/SMD(C_6H_6).

	E / eV	*f*	Orbital Contributions
**S1**	4.455	0.092	HOMO → LUMO 44%, HOMO-1 → LUMO+1 22 %
**S2**	4.472	0.503	HOMO-1 → LUMO 41%, HOMO→LUMO+1 25%

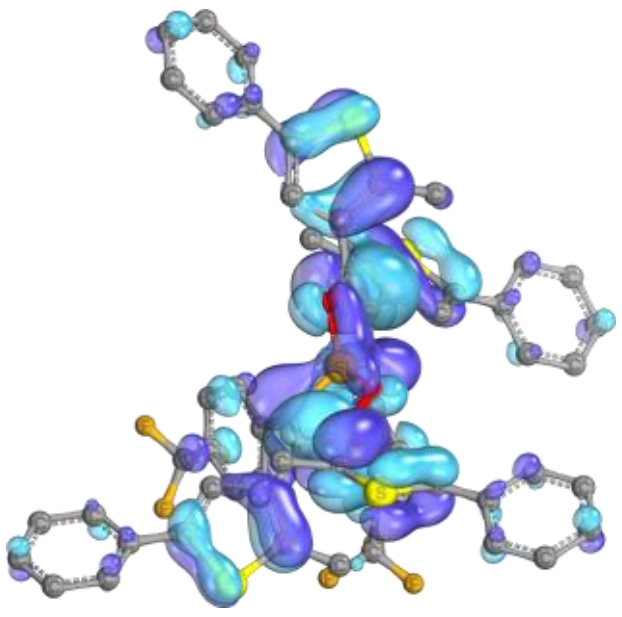

HOMO-1, E = –7.50 eV

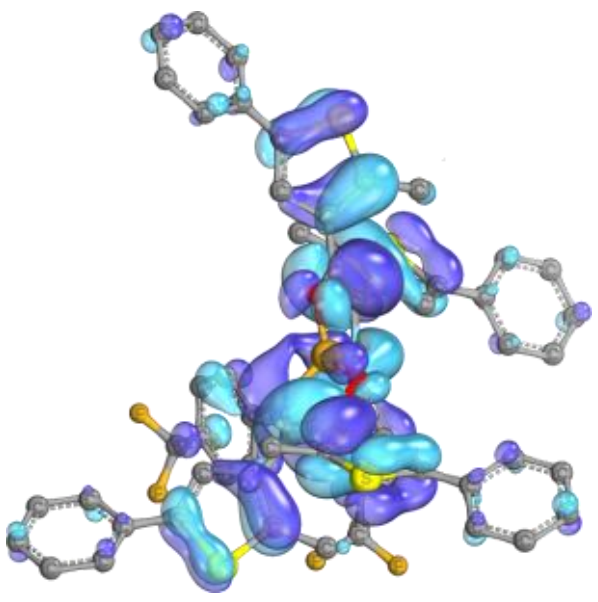

HOMO, E = –7.41 eV

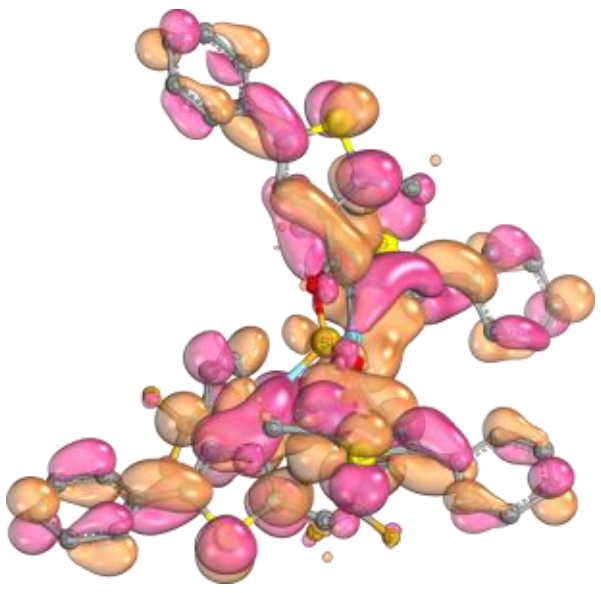

LUMO, E = 0.56 eV

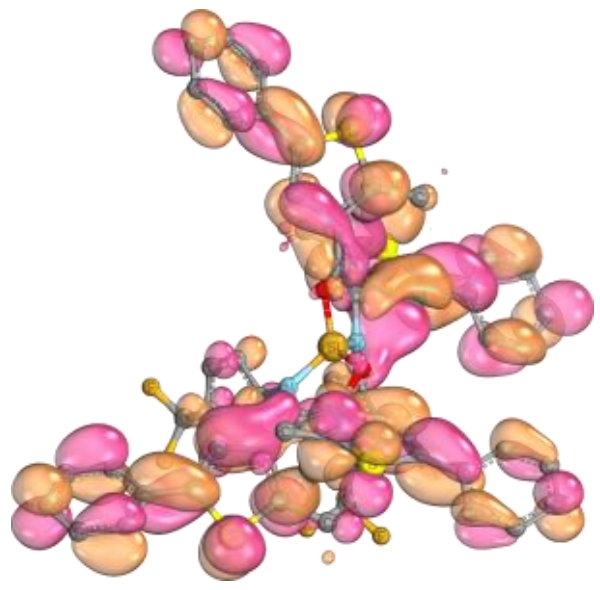

LUMO+1, E = 0.61 eV

1-oc

For S2-S5 of **1-oc**, 4 or more orbital transitions have major involvement. S2-S4 are entirely localized on the closed ligand. S5 is the low-energy excitation of the open ligand.

Table S3.7. Low energy transitions of **1-oc**. Method: ωB97X-D3/def2-TZVP/SMD(C_6H_6).

	E / eV	*f*	Orbital Contributions
**S1**	2.575	0.524	HOMO → LUMO 92%
**S5**	4.489	0.270	NTOs, see below

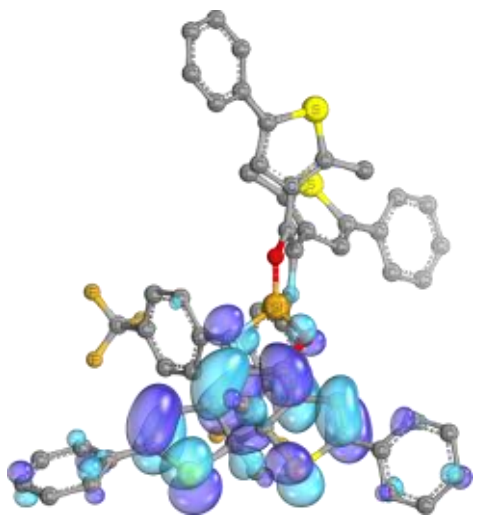

HOMO, E = –7.41 eV

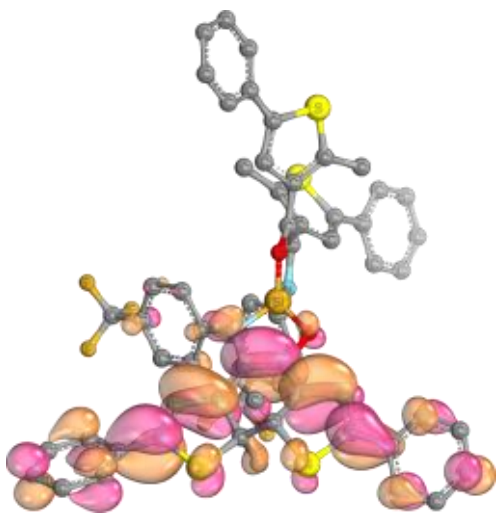

LUMO, E = –0.56 eV

NTOs for S5 of **1-oc. HONTO→LUNTO 91%, HONTO-1→LUNTO+1 3%**

HONTO-1

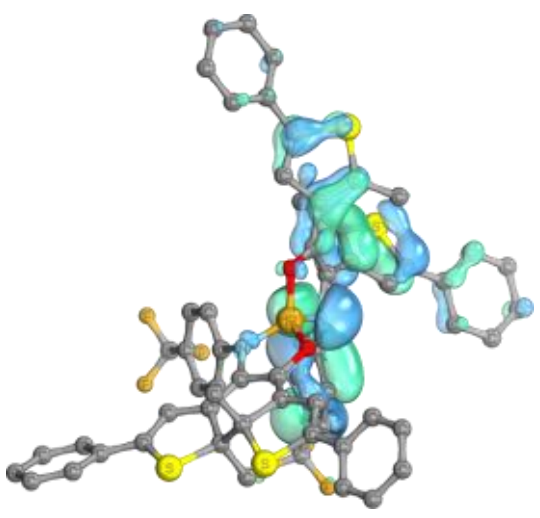

HONTO

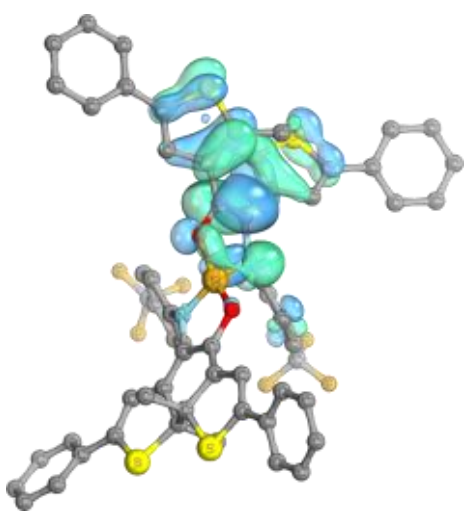

LUNTO

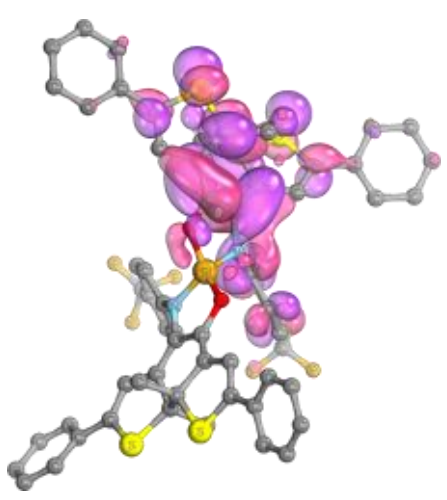

LUNTO+1

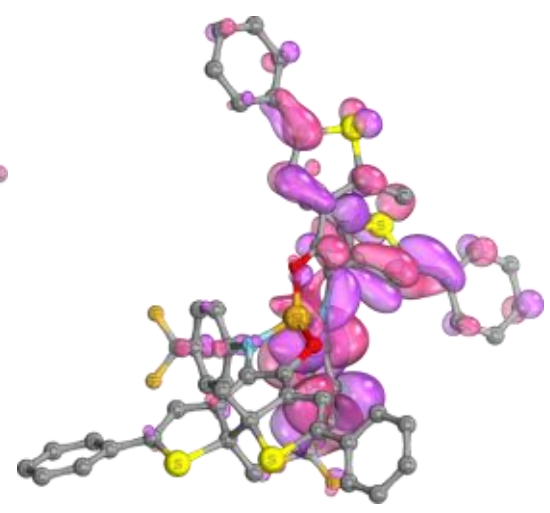

1-oc-Et_3PO

For S2-S5 of **1-oc-Et_3PO**, 4 or more orbital transitions have major involvement. S2-S4 are entirely localized on the closed ligand. S5 is the low-energy excitation of the open ligand.

Table S3.7. Low energy transitions of **1-oc**. Method: ωB97X-D3/def2-TZVP/SMD(C_6H_6).

	E / eV	*f*	Orbital Contributions
**S1**	2.508	0.577	HOMO → LUMO 92%
**S5**	4.152	0.263	NTOs, see below

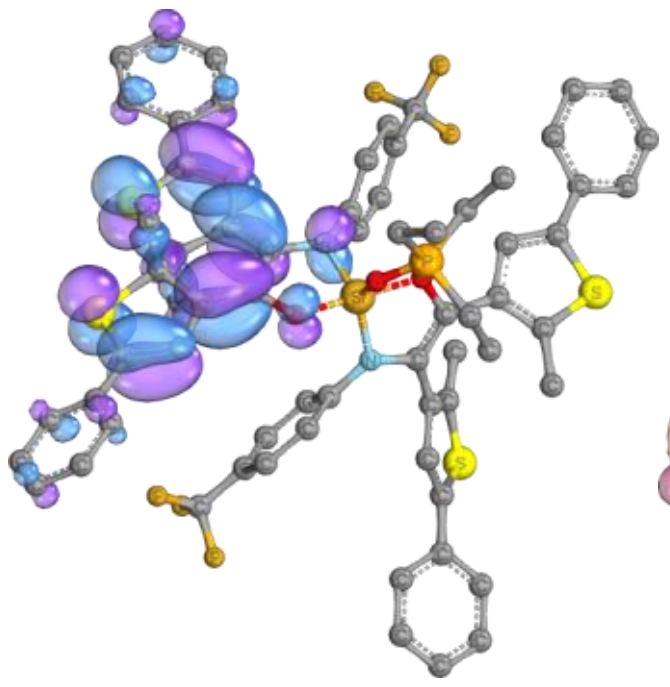

HOMO, E = –6.210 eV

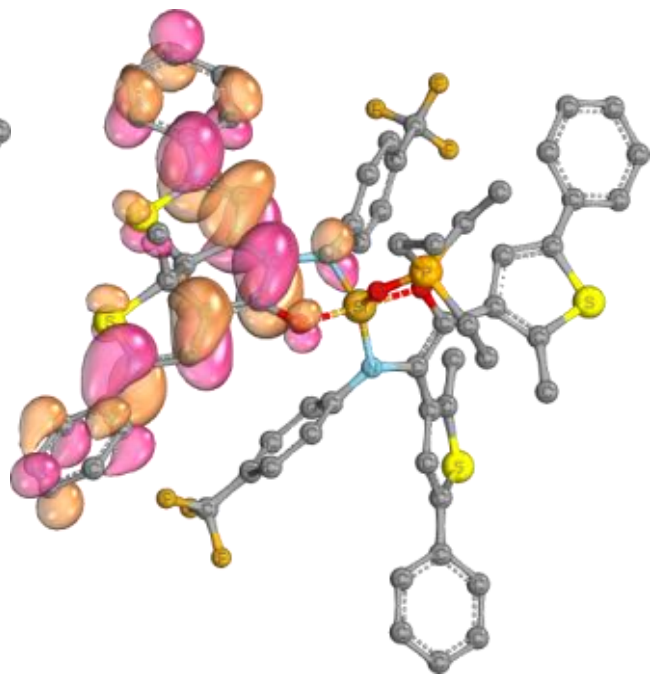

LUMO, E = –0.015 eV

NTOs for S4 of **1-oc. HONTO→LUNTO 44%, HONTO-1→LUNTO+1 30%, HONTO-2→LUNTO+2 22%**

HONTO-2

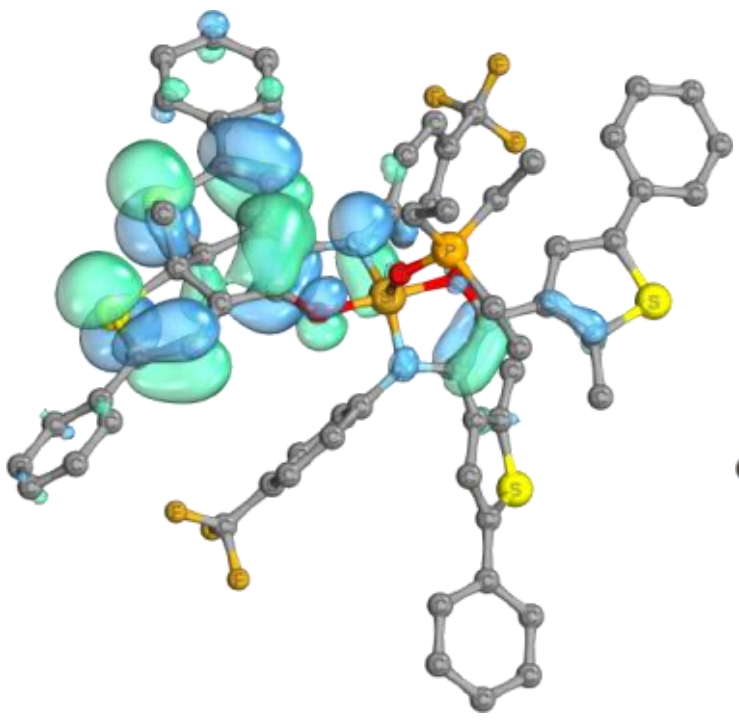

HONTO-1

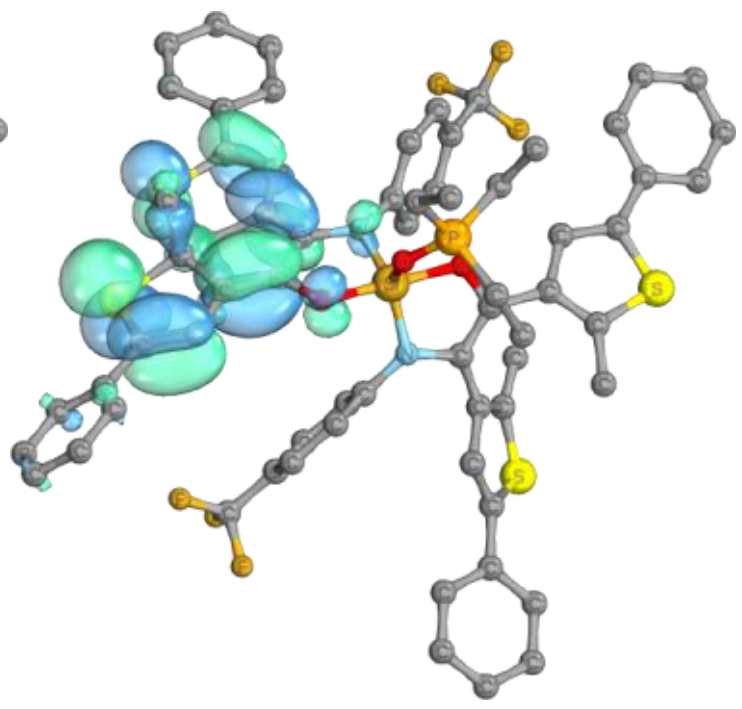

HONTO

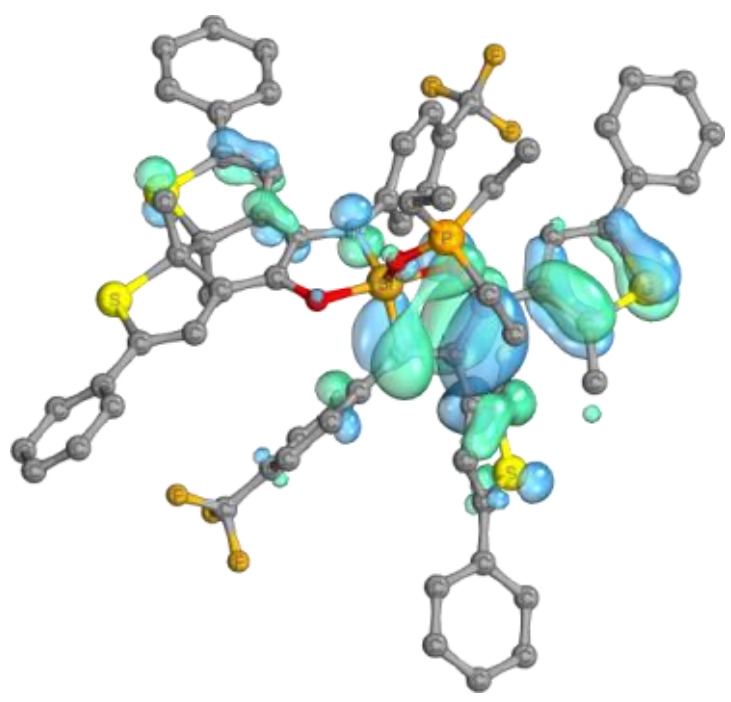

LUNTO+2

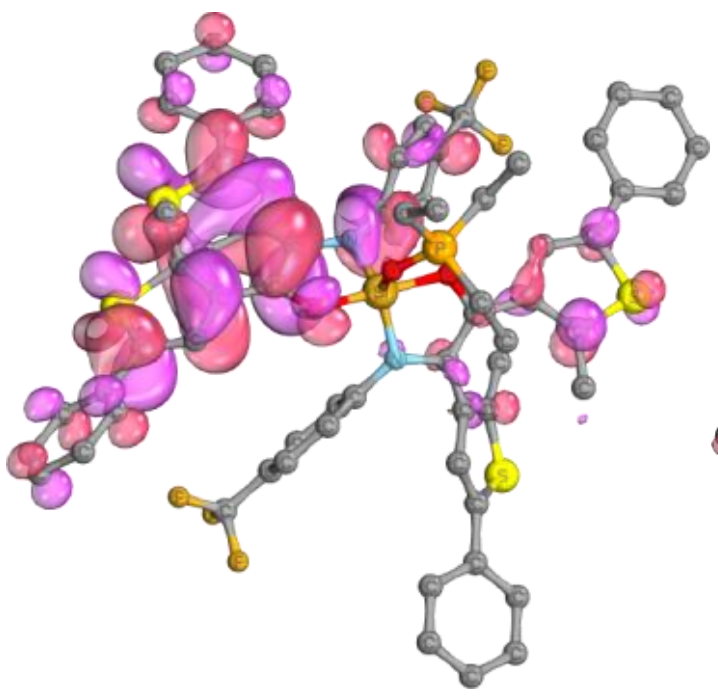

LUNTO+1

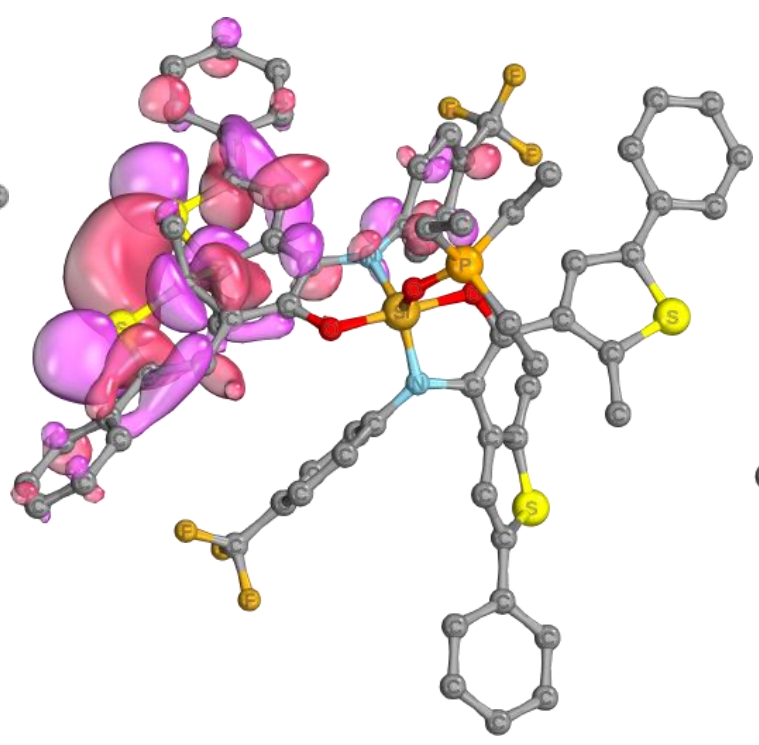

LUNTO

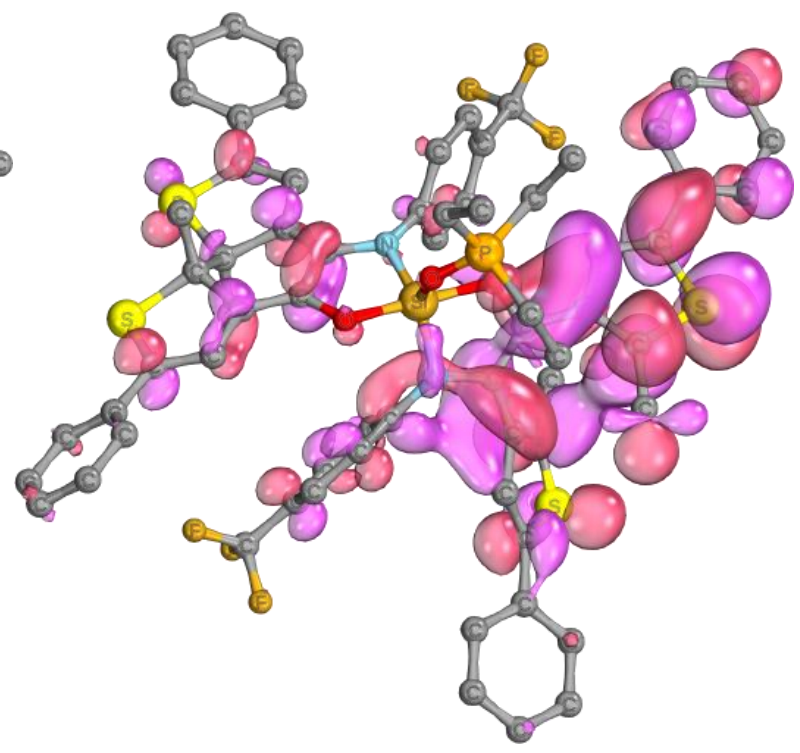

1-cc

Table S3.8. Low energy transitions of **1-cc**. Method: ωB97X-D3/def2-TZVP/SMD(C_6H_6).

	E / eV	*f*	Orbital Contributions
**S1**	2.567	0.531	HOMO → LUMO 50%, HOMO-1→LUMO+1 42%
**S2**	2.570	0.520	HOMO → LUMO+1 48%, HOMO-1→LUMO 44%

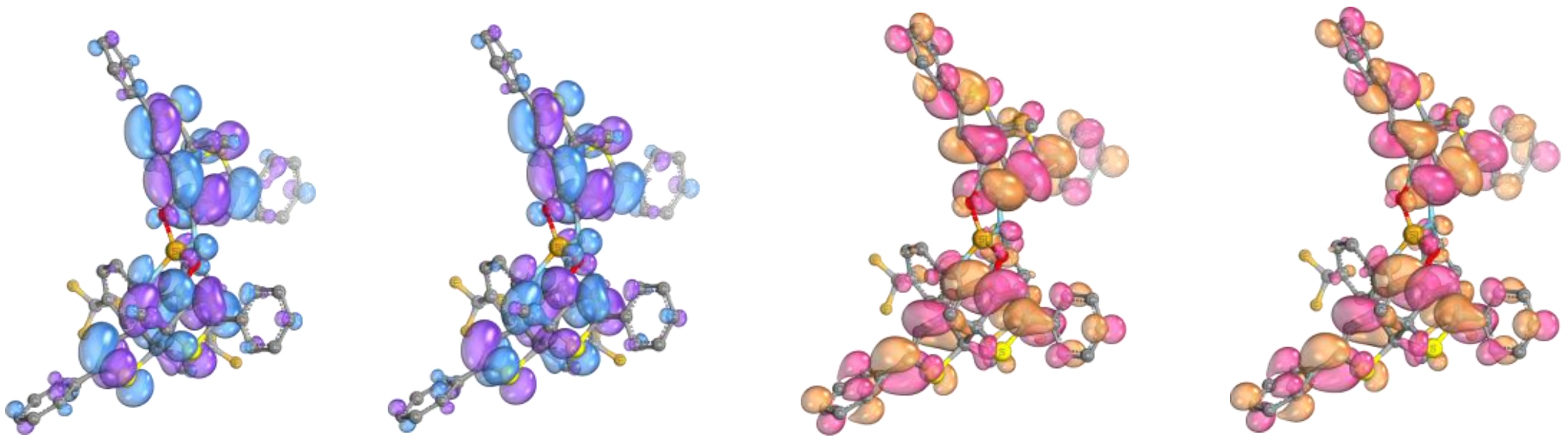

HOMO-1, E = –6.72 eV ***HOMO, E = –6.60 eV*** ***LUMO, E = –0.57 eV*** ***LUMO+1, E = –0.53 eV***

2-oo

For both S1 and S2 of **2-oo**, more than 5 MO transitions have major contributions. NTOs are given below.

Table S3.9. Low energy transitions of **2-oo**. Method: ωB97X-D3/def2-TZVP/SMD(C_6H_6).

	E / eV	*f*	Orbital Contributions
**S1**	4.400	0.416	NTOs, see below
**S2**	4.404	0.034	NTOs, see below

NTOs for S1 of **2-oo. HONTO→LUNTO 51%, HONTO-1→LUNTO+1 36%**

HONTO-1 ***HONTO*** ***LUNTO*** ***LUNTO+1***

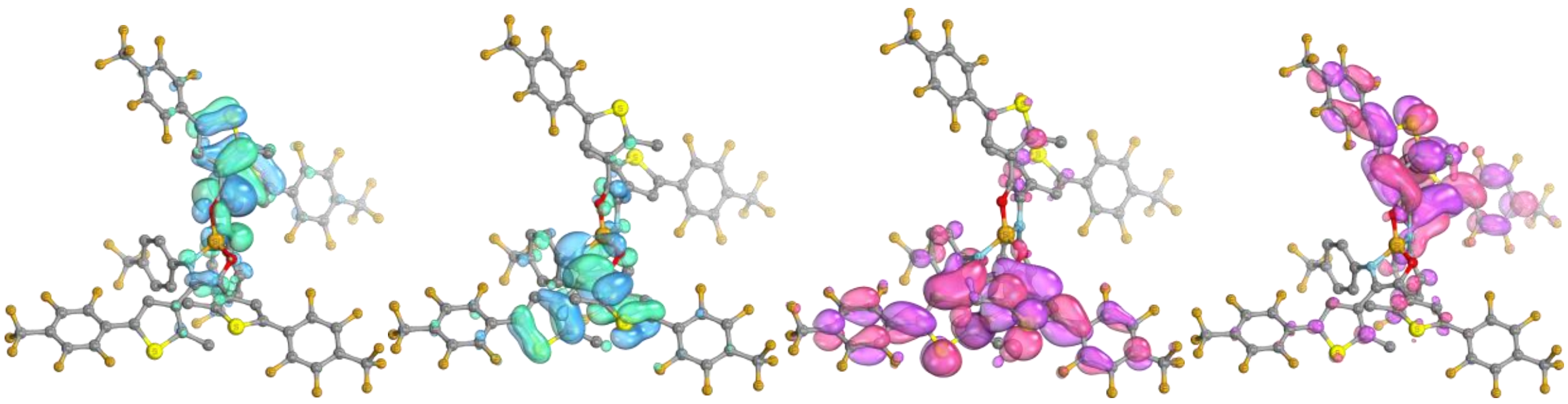

NTOs for S2 of **2-oo. HONTO→LUNTO 49%, HONTO-1→LUNTO+1 37%**

HONTO-1 ***HONTO*** ***LUNTO*** ***LUNTO+1***

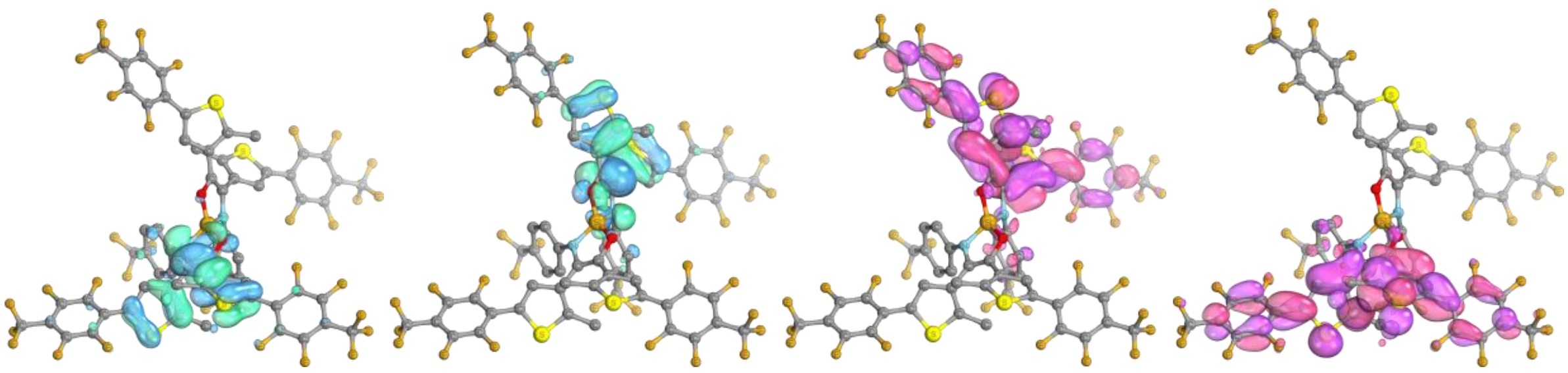

2-oc

For **2-oc**, S1-S3 are localized on the closed ligand. S4 is the low-energy excitation of the open ligand and has multiple significant MO contributions. NTOs are given below.

Table S3.10. Low energy transitions of **2-oc**. Method: ωB97X-D3/def2-TZVP/SMD(C_6H_6).

	E / eV	*f*	Orbital Contributions
**S1**	2.535	0.465	HOMO→LUMO 92%
**S4**	4.436	0.265	NTOs, see below

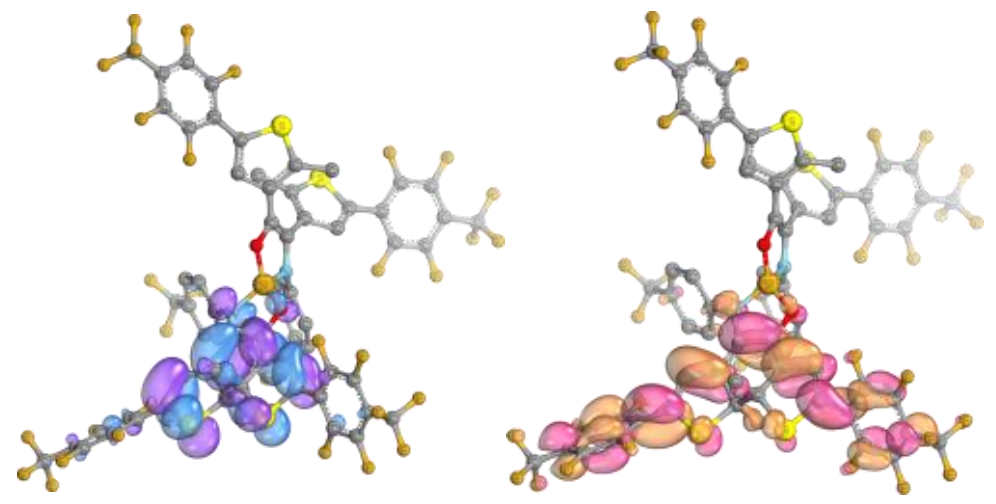

HOMO, E = –7.14 eV ***LUMO, E = –1.08 eV***

NTOs for S4 of **2-oc. HONTO→LUNTO 85%, HONTO-1→LUNTO+1 8%, HONTO-2→LUNTO+2 3%**

HONTO-2 ***HONTO-1*** ***HONTO***

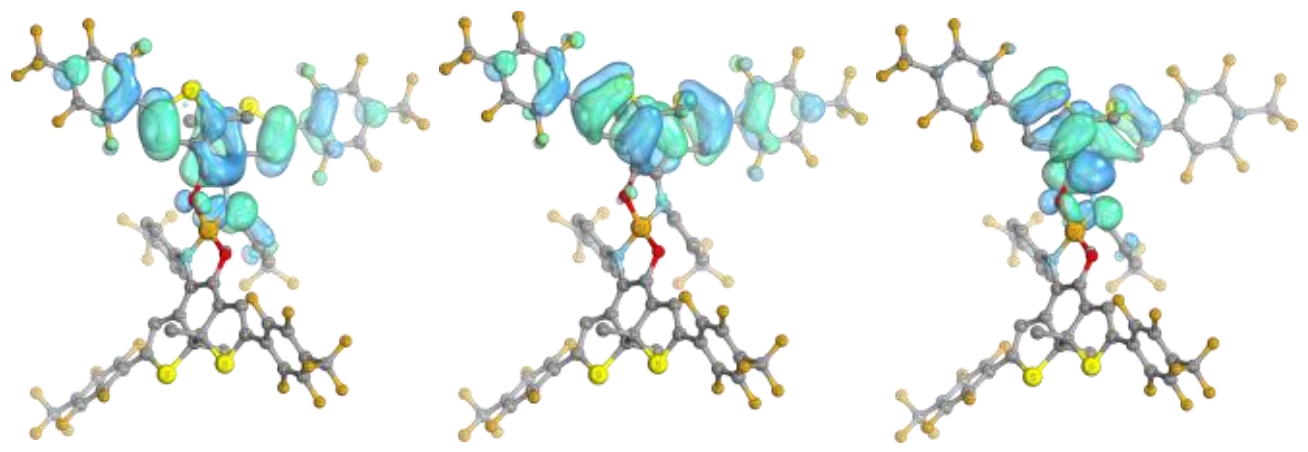

LUNTO+2 ***LUNTO+1*** ***LUNTO***

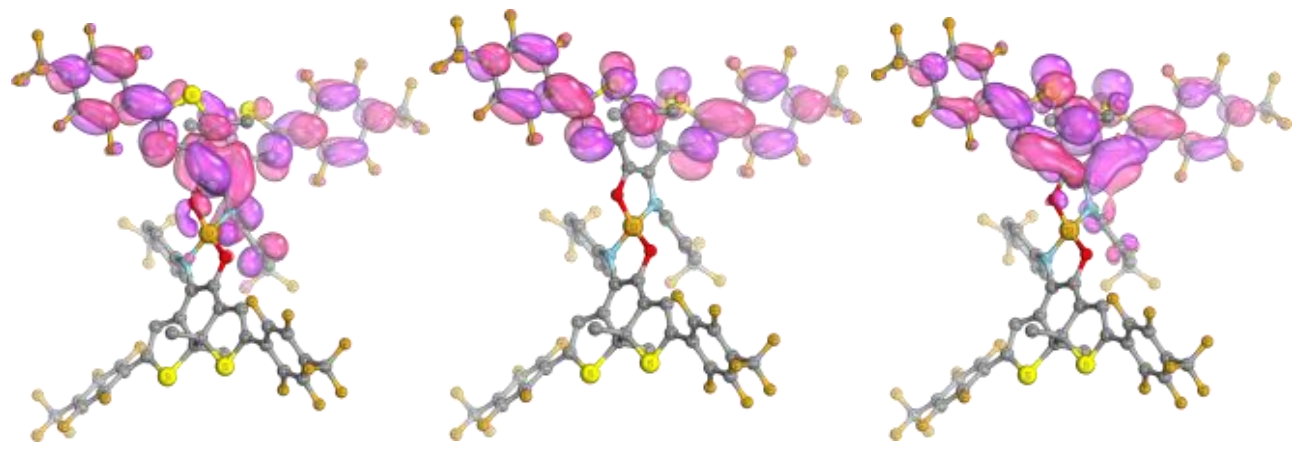

2-cc

Table S3.11. Low energy transitions of **2-cc**. Method: ωB97X-D3/def2-TZVP/SMD(C_6H_6).

	E / eV	*f*	Orbital Contributions
**S1**	2.536	0.448	HOMO → LUMO 49%, HOMO-1→LUMO+1 42%
**S2**	2.539	0.476	HOMO → LUMO+1 47%, HOMO-1→LUMO 44%

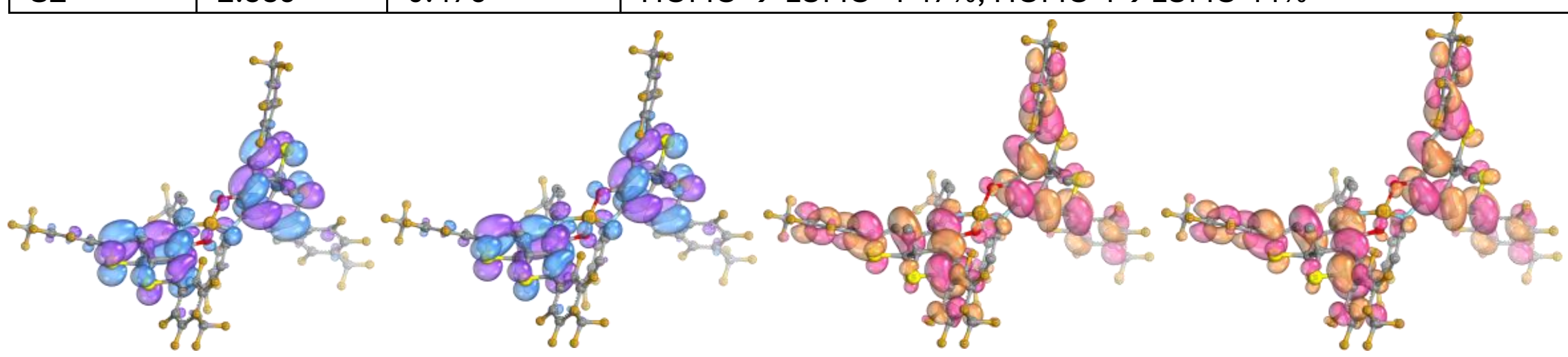

HOMO-1, E = –7.26 eV ***HOMO, E = –7.15 eV*** ***LUMO, E = –1.15 eV*** ***LUMO+1, E = –1.12 eV***

2-oo-Et_3PO

S1-S2 of **2-oo-Et_3PO** can be sufficiently described by 3 or less MO transitions. For simplicity, NTOs are discussed in the main manuscript. These are additionally shown below.

Table S3.12. Low energy transitions of **2-oo-Et_3PO**. Method: ωB97X-D3/def2-TZVP/SMD(C_6H_6).

	E / eV	*f*	Orbital Contributions
**S1**	3.948	0.059	HOMO → LUMO 49%, HOMO-1→LUMO 42%
**S2**	3.968	0.071	HOMO → LUMO+1 50%, HOMO-1→LUMO+1 17%, HOMO→LUMO+5 13%

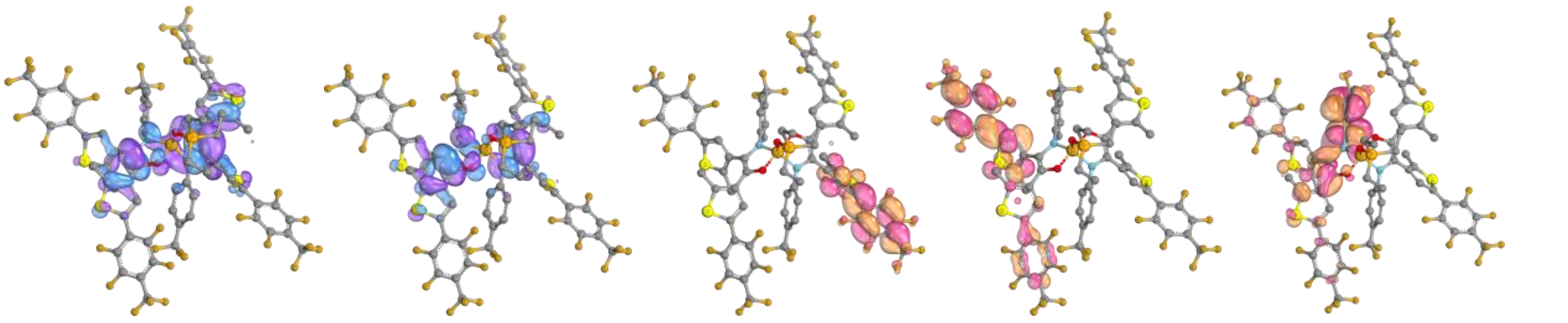

HOMO-1, E = –7.28 eV ***HOMO, E = –6.87 eV*** ***LUMO, E = –0.12 eV*** ***LUMO+1, E = 0.00 eV*** ***LUMO+5, E = 1.02 eV***

NTOs for S1 of **2-oo-Et_3PO. HONTO→LUNTO 97%**

HONTO ***LUNTO***

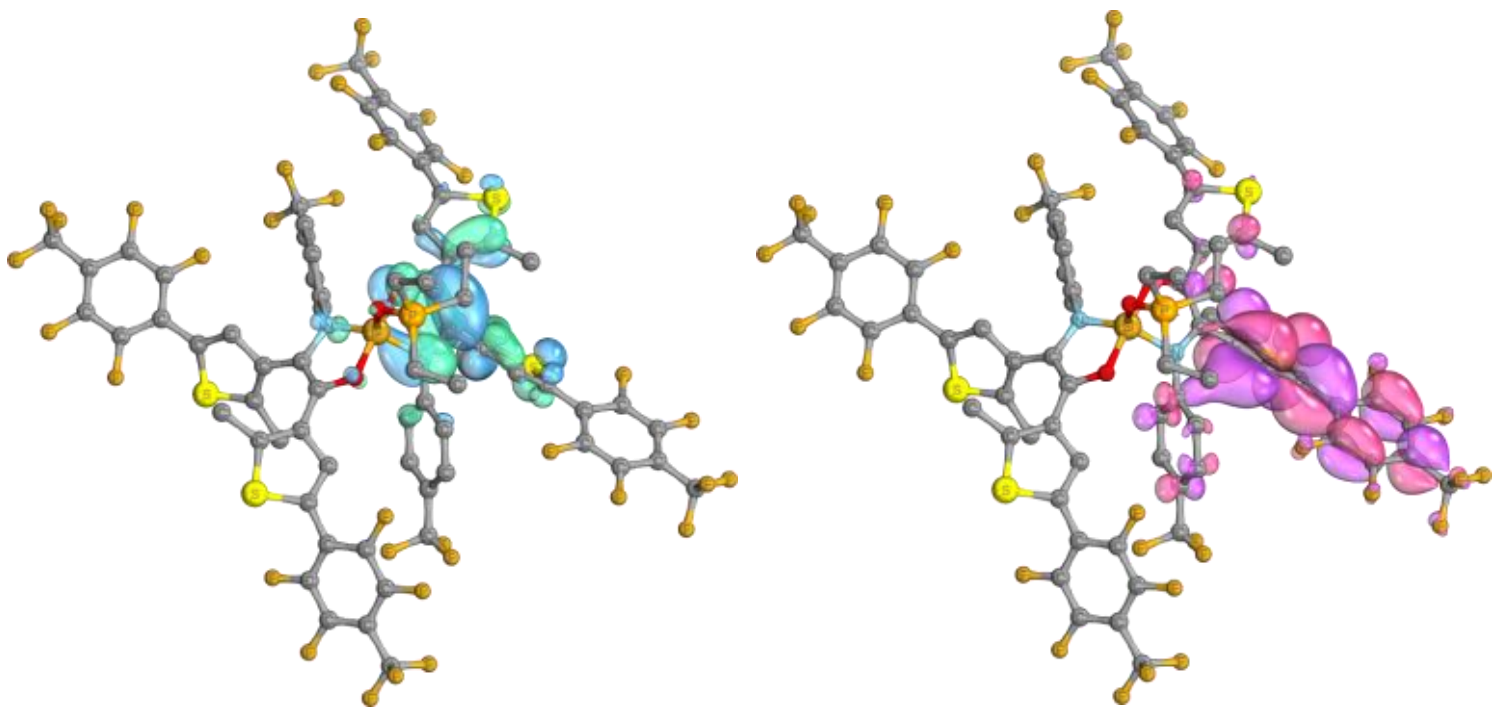

NTOs for S2 of **2-oo-Et_3PO. HONTO→LUNTO 96%**

HONTO ***LUNTO***

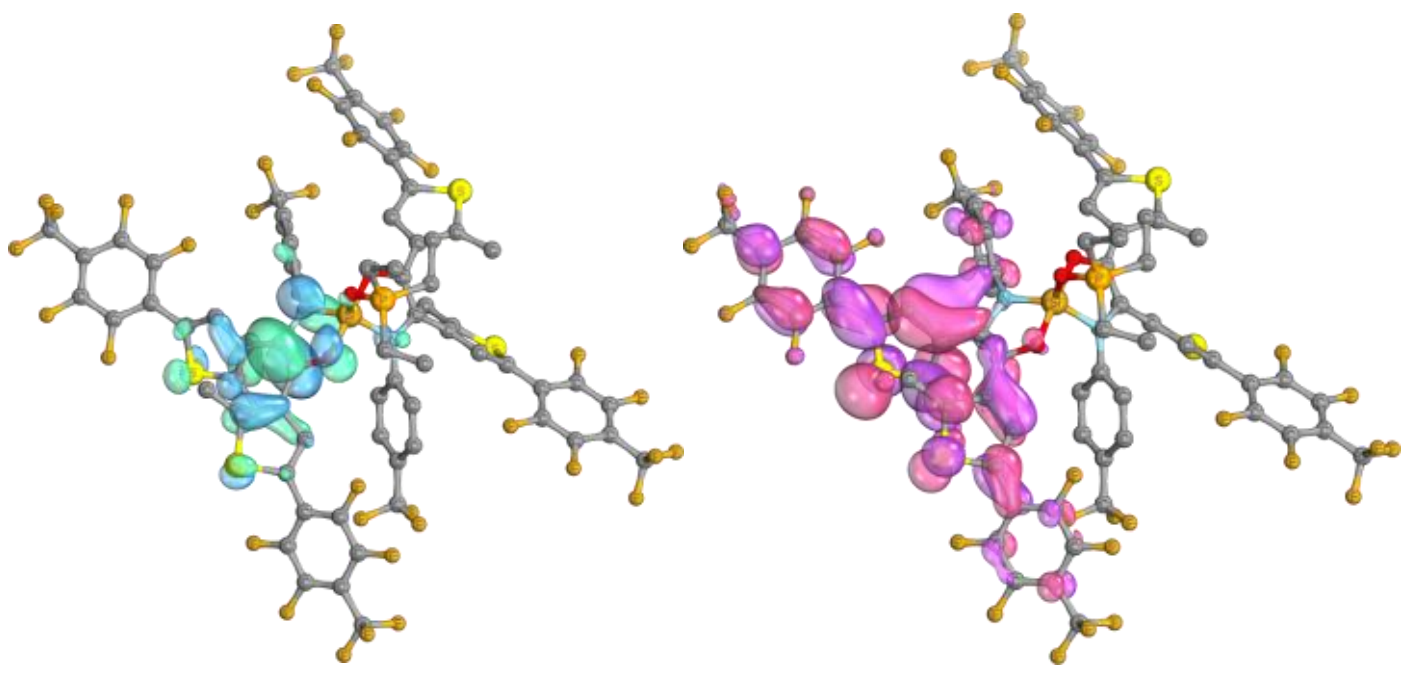

2-oc-Et_3PO

For **2-oc-Et_3PO**, S1-S3 are localized on the closed ligand. S4 is the low-energy excitation of the open ligand and has multiple significant MO contributions. NTOs are given below.

Table S3.13. Low energy transitions of **2-oc-Et_3PO**. Method: ωB97X-D3/def2-TZVP/SMD(C_6H_6).

	E / eV	*f*	Orbital Contributions
**S1**	2.466	0.535	HOMO → LUMO 90%
**S4**	4.046	0.149	NTOs, see below

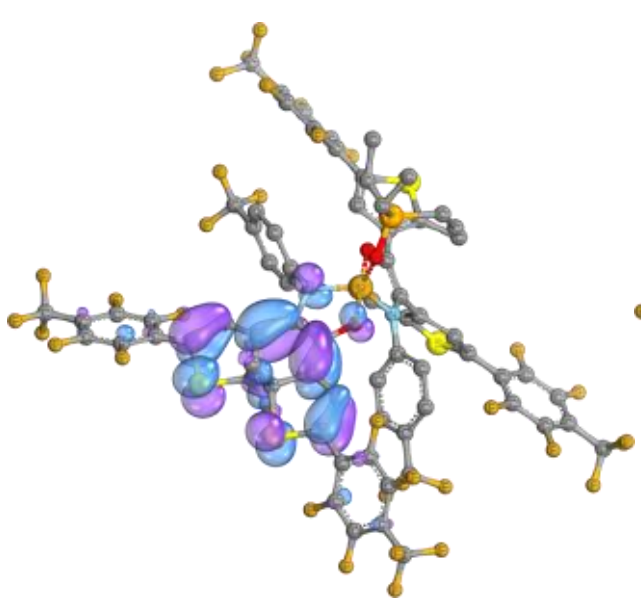

HOMO, E = –6.60 eV

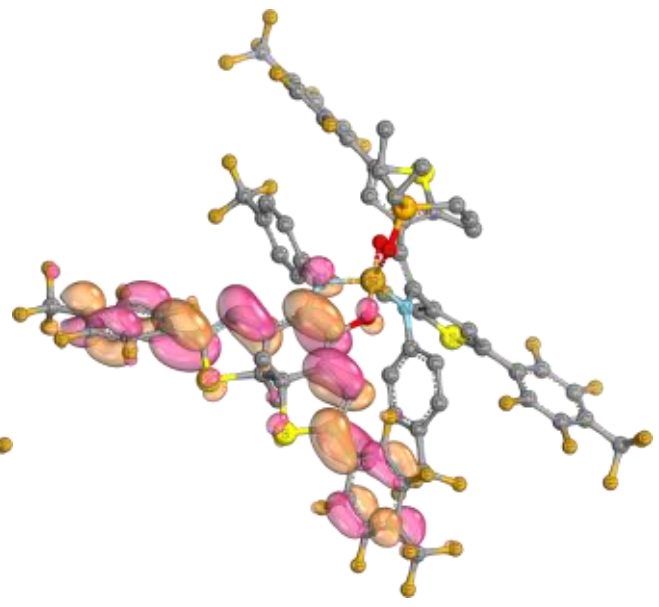

LUMO, E = –0.66 eV

NTOs for S4 of **2-oc-Et_3PO. HONTO→LUNTO 85%, HONTO-1→LUNTO+1 6%, HONTO-2→LUNTO+2 4%**

HONTO-2

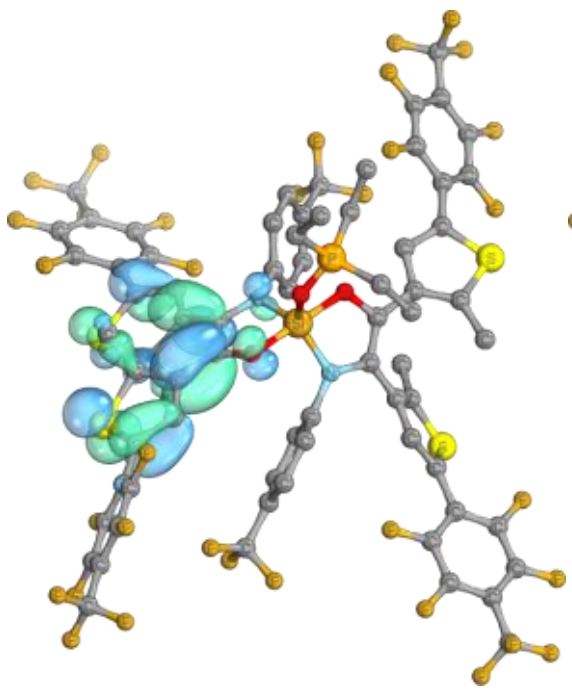

HONTO-1

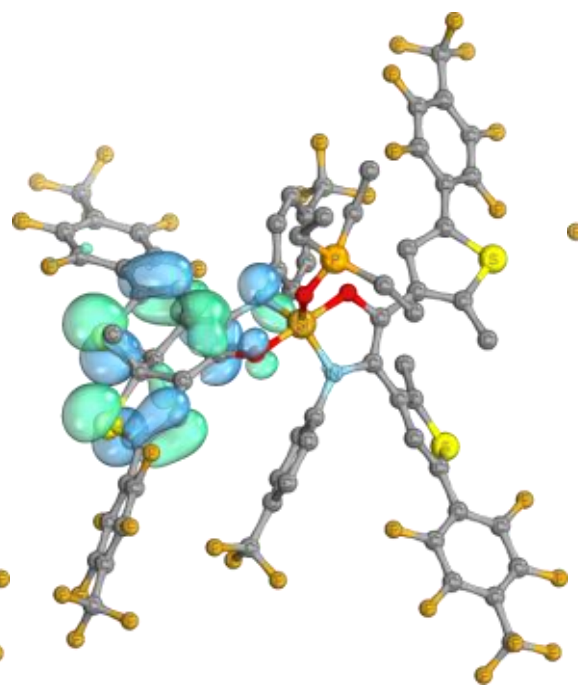

HONTO

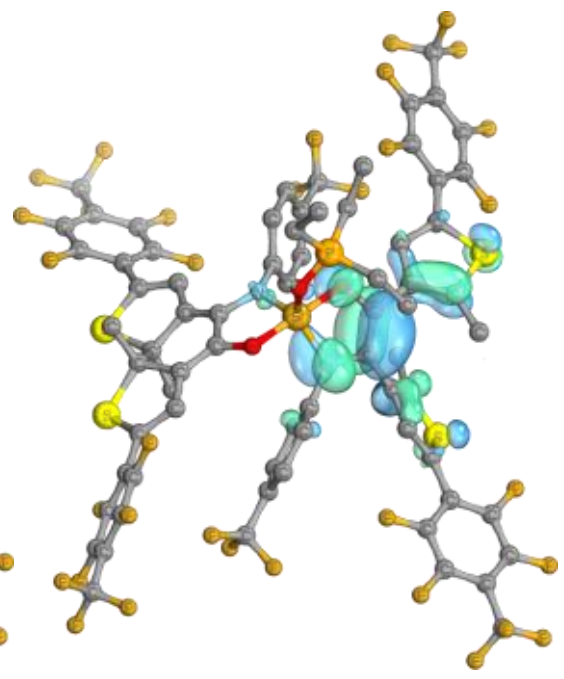

LUNTO+2

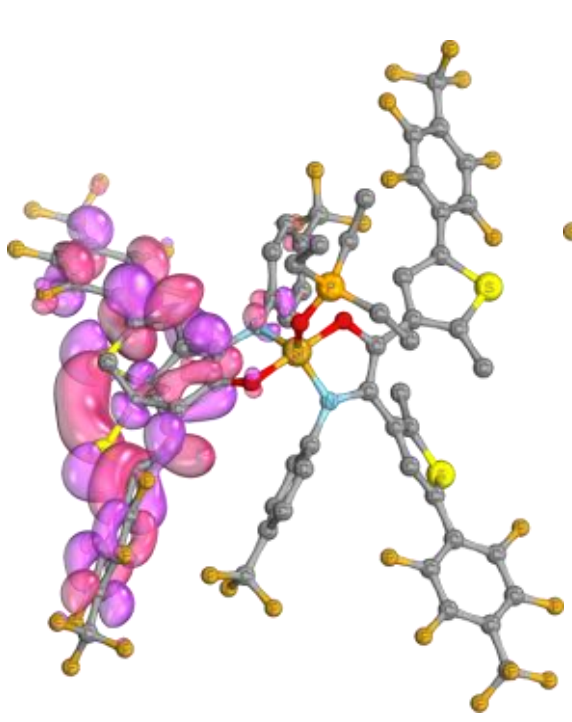

LUNTO+1

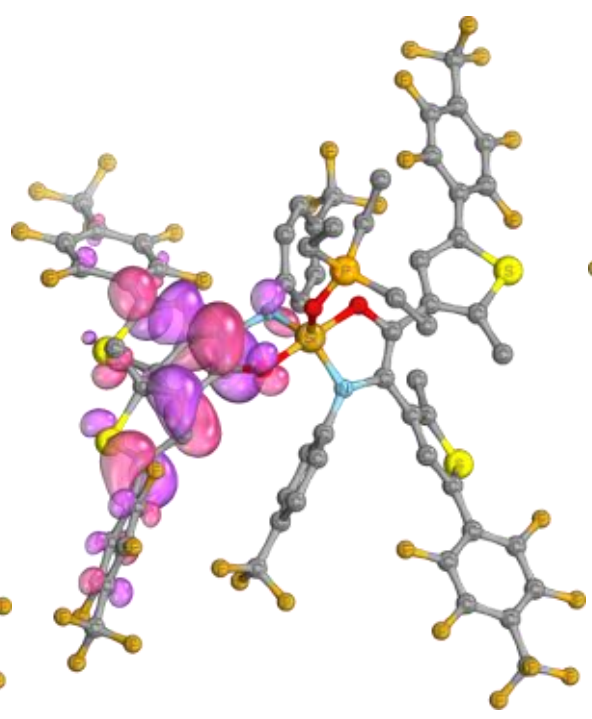

LUNTO

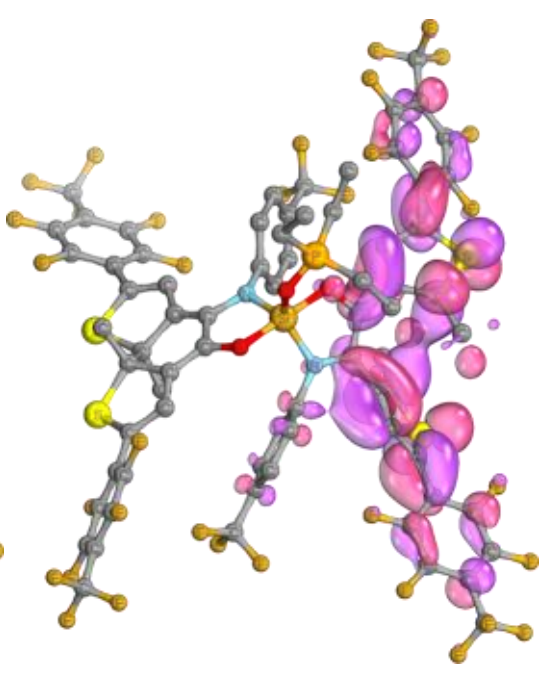

[2-oo-F]

Table S3.13. Low energy transitions of **[2-oo-F]$^{-}$**. Method: ωB97X-D3/def2-TZVP/SMD(C_6H_6).

	E / eV	*f*	Orbital Contributions
**S1**	3.547	0.029	NTOs, see below
**S2**	3.558	0.032	NTOs, see below

NTOs for S1 of **[2-oo-F]$^{-}$. HONTO→LUNTO 66%, HONTO-1→LUNTO+1 32%**

HONTO-1 ***HONTO*** ***LUNTO*** ***LUNTO+1***

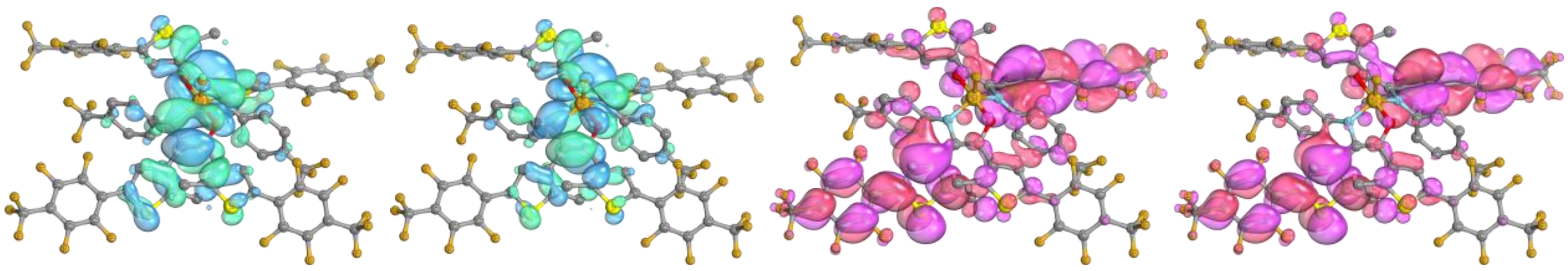

NTOs for S2 of **[2-oo-F]$^{-}$. HONTO→LUNTO 66%, HONTO-1→LUNTO+1 32%**

HONTO-1 ***HONTO*** ***LUNTO*** ***LUNTO+1***

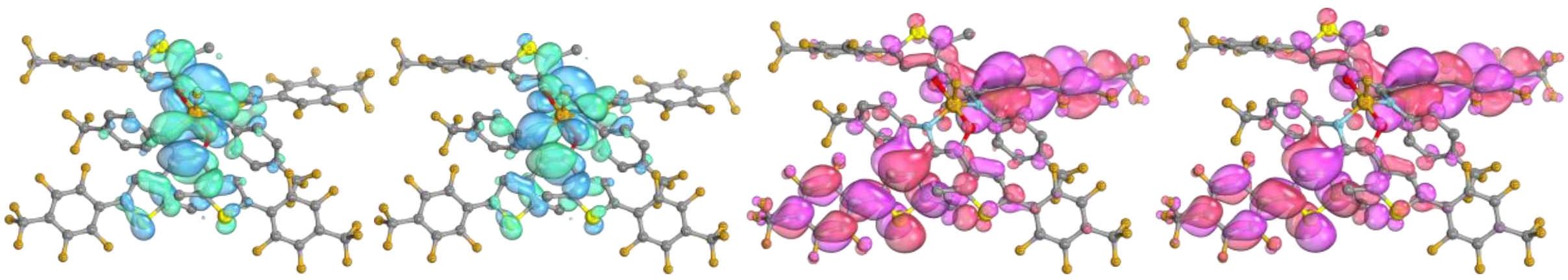

Calculated UV-vis spectra of 2-oo, 2-oo-Et_3PO, and [2-oo-F]$^-$

Calculated spectra were plotted in Chemcraft, with gaussian linebroadening overlayed (bandwidth 25 nm).

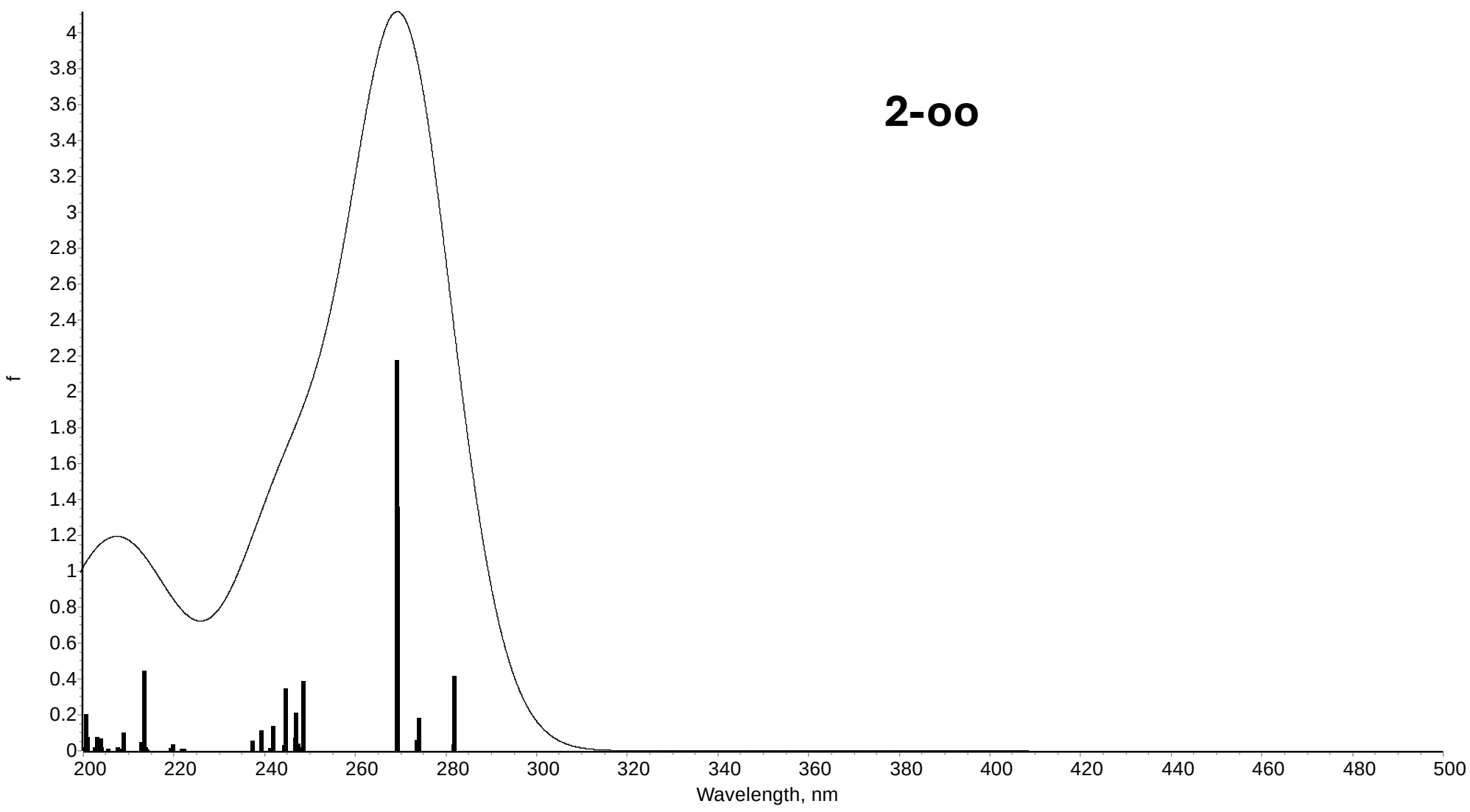

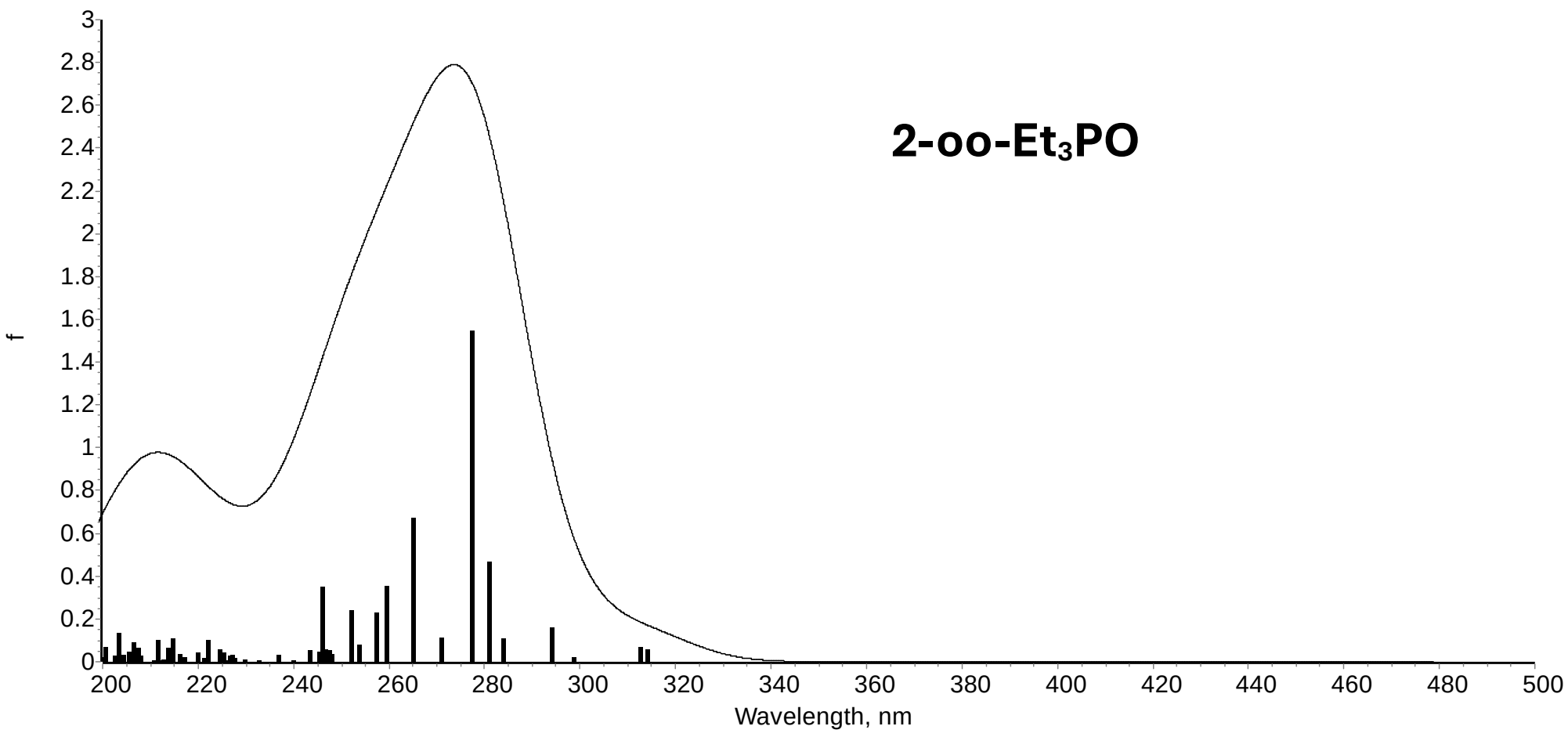

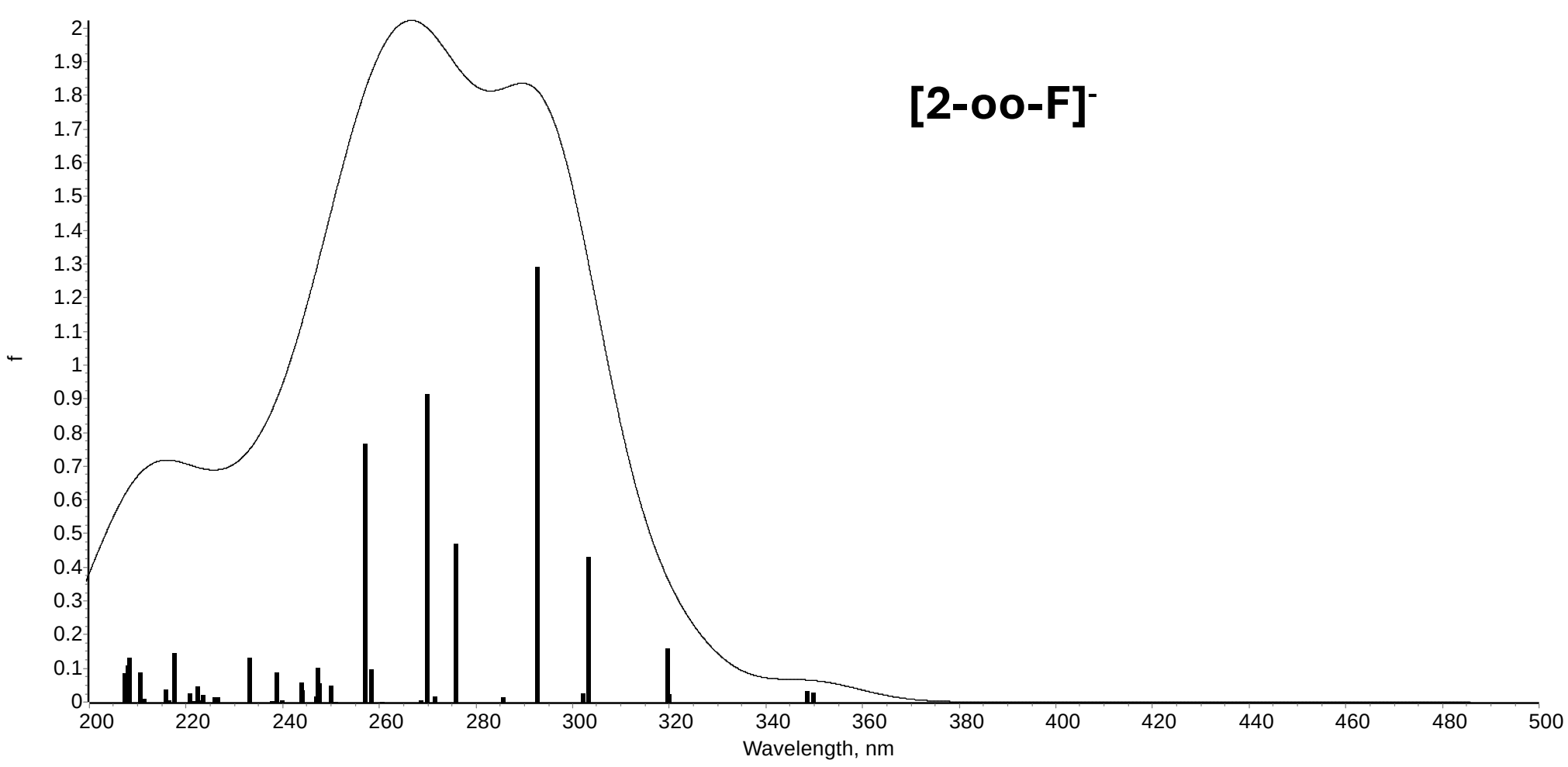

4 Reactivity Studies

4.1 NMR Scale Photocyclization (1)

1-oo (3.3 mg) was dissolved in 0.5 ml C_6D_6 with cyclooctane as an internal standard. NMR spectra were measured at different irradiation times.

Determination of 1-oo, 1-oc and 1-cc (Example ^{1}H NMR spectra at 60 and 690 minutes)

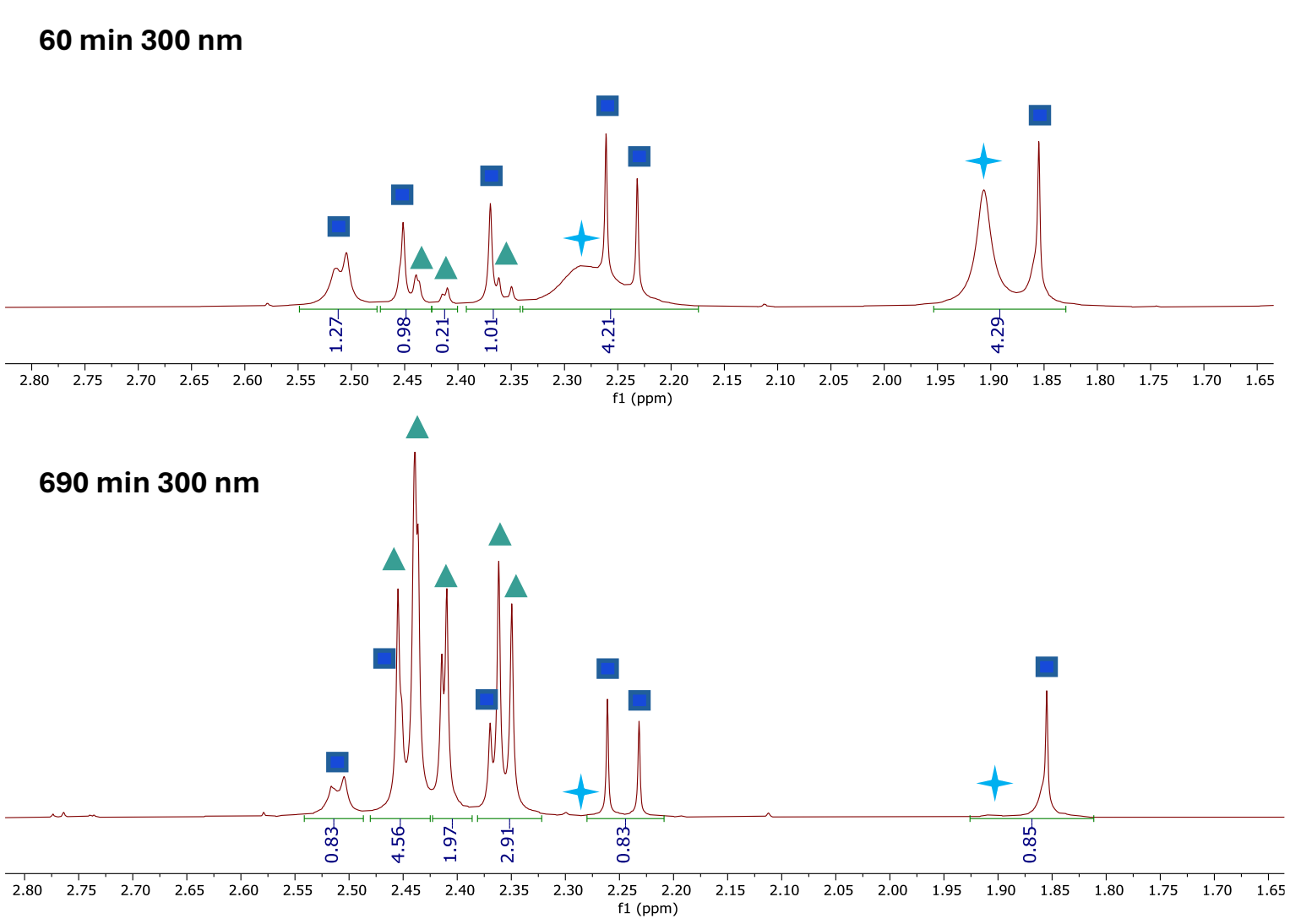

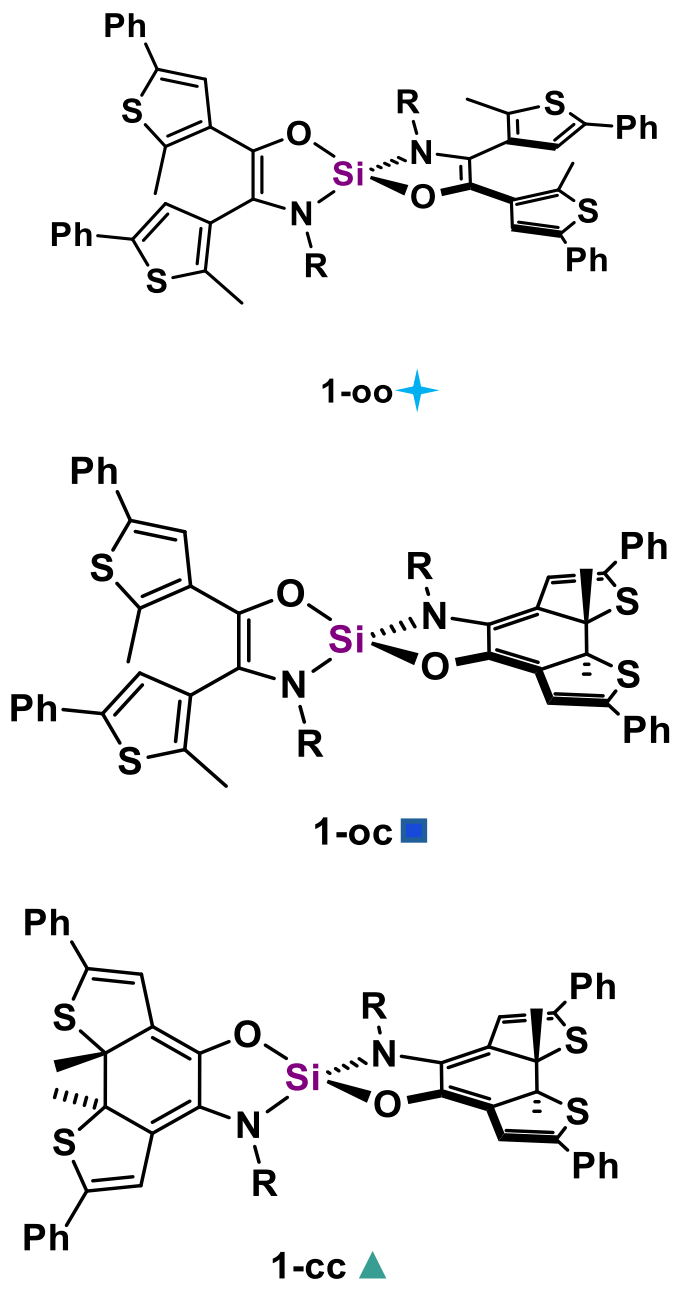

Figure S4.1. Excerpt of the ^{1}H NMR spectrum (600 MHz, C_6D_6) of **1** after 60 and 690 minutes irradiation at 300 nm.

Exemplary determination of 1-oo, 1-oc and 1-cc at 60 minutes irradiation

The sum of the integrals for all methyl signals was set to 12.

The **1-oc** ratio was determined by integrating the methyl resonances at 2.52 ppm. These represent 2 out of the 8 methyl signals for **1-oc**.

➡ Ratio of **1-oc** =1.27*4/12= 0.42

For **1-oo**, the methyl signal at 1.91 ppm (6 out of 12 protons of **1-oo**) was integrated together with the **1-oc** signal at 1.85 ppm (2 out of 8 **1-oc** methyl resonances). Subtracting the known integral of **1-oc** (1.27) allowed for the quantification of **1-oo**:

➡ Ratio of **1-oo** = (4.29-1.27)*2/12 = 0.50

The **1-cc** ratio was determined by two separate methods:

1) Subtracting **1-oo** and **1-oc** from the total:
   ➡ Ratio of **1-cc** = 1-0.42-0.50 = 0.08

2) Integrating all **1-cc** signals and dividing by 12. This method is potentially error prone due to significant overlap with the **1-oc** signals.

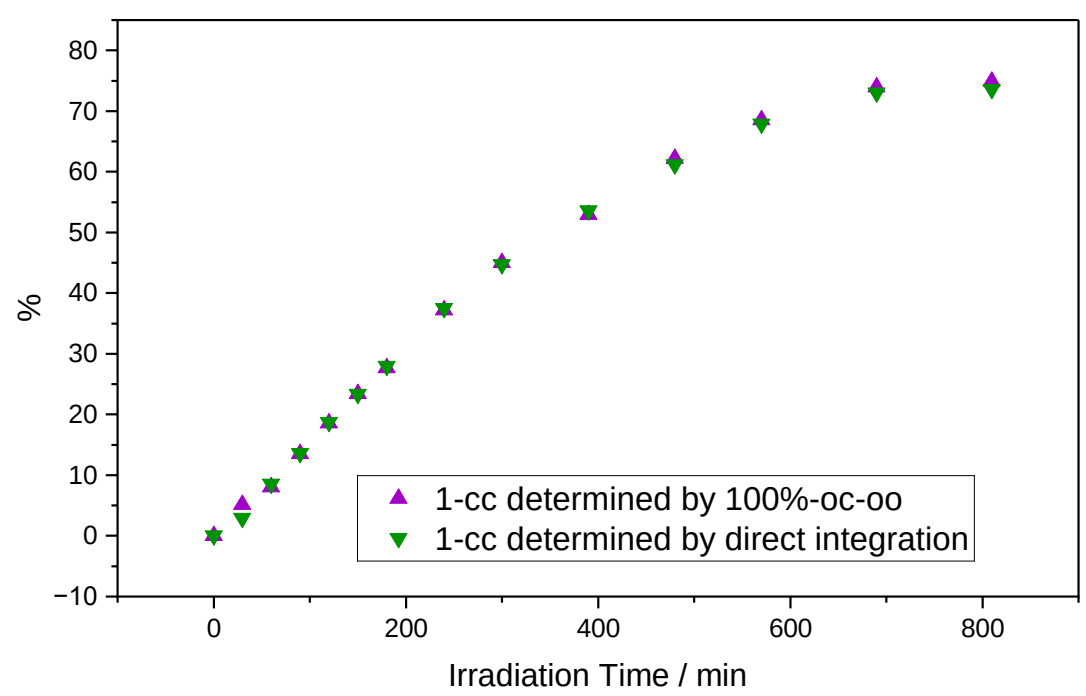

Figure S4.2. Comparison of quantification methods for **1-cc**.

^{1}H NMR spectra at 0, 60, and 690 minutes UV, and after VIS irradiation

In addition to characteristic methyl resonances, the CH resonances of closed ligands on the nitrogen-substituted side are readily assigned and appear at unusually high field, due to shielding by the aromatic ring current of the spatially adjacent nitrogen aryl substituent. These signals are broadened for **1-oc**, but well-resolved for **1-cc**, consistent with the loss of conformational dynamics upon the second ring-closure.

The initial spectrum is restored after VIS irradiation, with some reduction in signal intensity (92% of the pre-irradiation integral based on internal standard) due to hydrolysis and photochemical side-reactions.

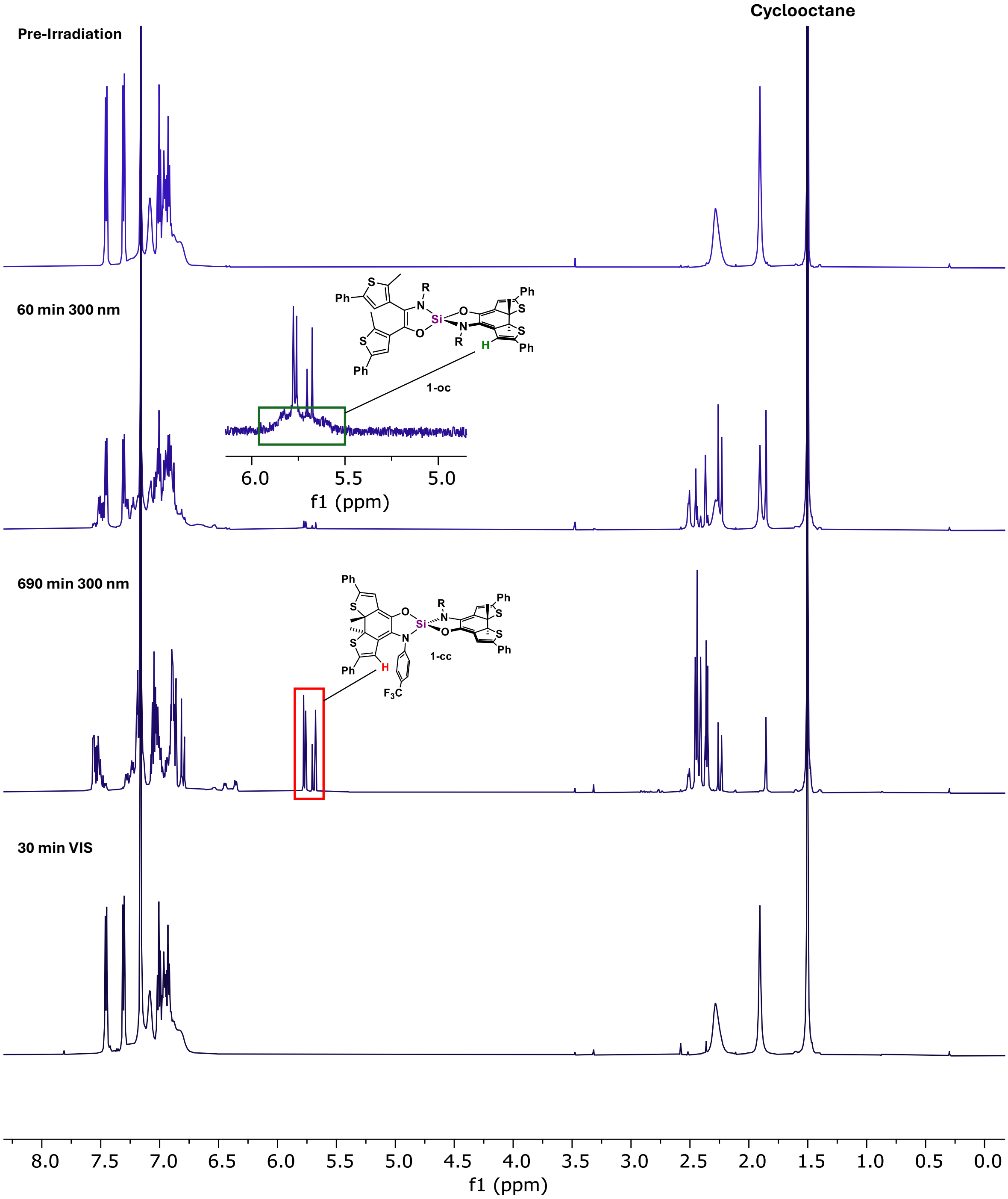

Figure S4.3. ^{1}H NMR spectra (600 MHz, C_6D_6) of **1** before irradiation, after 60 and 690 minutes irradiation at 300 nm, and after broadband visible light irradiation for 30 minutes.

Switching State Distribution

Assuming independent photoswitching, the **oo/oc/cc** ratio would be given by a binomial distribution, with x_{closed} being the total conversion to the closed isomer (irrespective of whether part of **oc** or **cc**):

$\mathbf{x_{oo}} = (1–x_{closed})^2$

$\mathbf{x_{oc}} = 1–(1–x_{closed})^2– x^2_{closed}$

$\mathbf{x_{cc}} = x^2_{closed}$

x_{closed}	x_{oo}	x_{oc}	x_{cc}
**0.00**	1.00	0.00	0.00
**0.05**	0.90	0.10	0.00
**0.10**	0.81	0.18	0.01
**0.15**	0.72	0.26	0.02
**0.20**	0.64	0.32	0.04
**0.25**	0.56	0.38	0.06
**0.30**	0.49	0.42	0.09
**0.35**	0.42	0.46	0.12
**0.40**	0.36	0.48	0.16
**0.45**	0.30	0.50	0.20
**0.50**	0.25	0.50	0.25
**0.55**	0.20	0.50	0.30
**0.60**	0.16	0.48	0.36
**0.65**	0.12	0.46	0.42
**0.70**	0.09	0.42	0.49
**0.75**	0.06	0.38	0.56
**0.80**	0.04	0.32	0.64
**0.85**	0.02	0.26	0.72
**0.90**	0.01	0.18	0.81
**0.95**	0.00	0.10	0.90
**1.00**	0.00	0.00	1.00

4.2 NMR scale photocyclization (2)

2-oo (6.4 mg) was dissolved in 0.5 ml C_6D_6 with cyclooctane as internal standard. After 200 minutes irradiation at 300 nm, a 2/26/72 mixture of **2-oo/2-oc/2-cc** was obtained. Ratios were determined as described for **1** via methyl resonances (section 4.1). Irradiation with visible light resulted in restoration of the initial NMR spectra, with 96% of signal intensity compared to pre-irradiation (based on integration against internal standard).

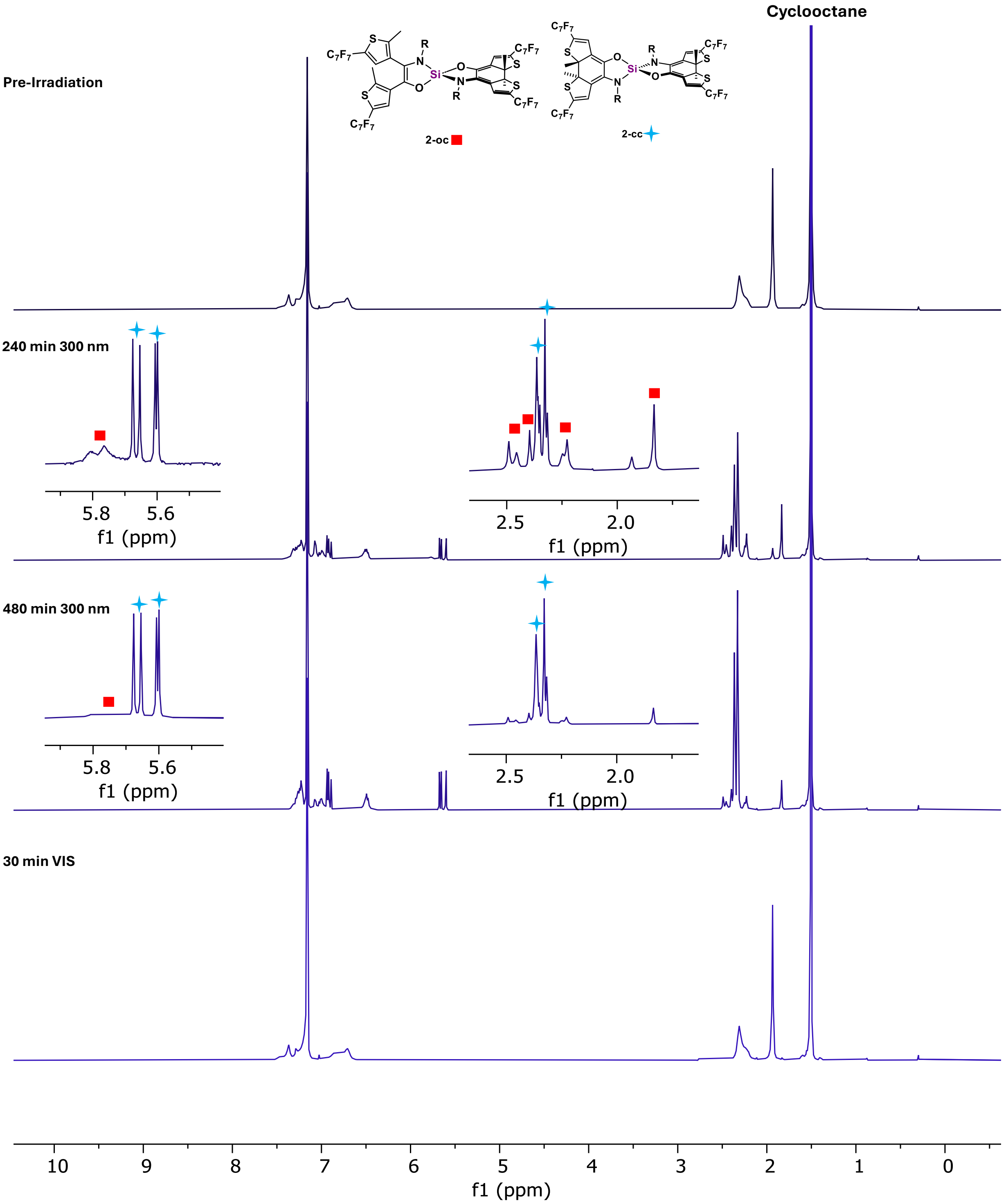

Figure S4.4. ^{1}H NMR spectra (600 MHz, C_6D_6) of **2** before irradiation, after 240 and 480 minutes irradiation at 300 nm, and after broadband visible light irradiation for 30 minutes.

4.3 Gutmann-Beckett Method

1-oo/2-oo were mixed with 0.5 equivalents of triethylphosphine oxide in C_6D_6. ^{31}P NMR spectra were measured pre-irradiation and after conversion to **oc/cc**.

1

For **1-oo**, only a very broad signal at 57.8 ppm could be observed. It should be noted that this signal is a result of an equilibrium between free and bound Et_3PO. The shift is therefore not a measure of effective Lewis acidity, and sensitive to the stoichiometry of Et_3PO and the overall concentration. In a different experiment, a shift of 55.8 ppm was observed. The signals at 77.7 ppm and 80.3 ppm are not subject to this variance. Resolved signals for **1-oc** and **1-cc** were obtained. For **1-cc**, three distinct signals are obtained due to slight differences in effective Lewis acidity between diastereomers.

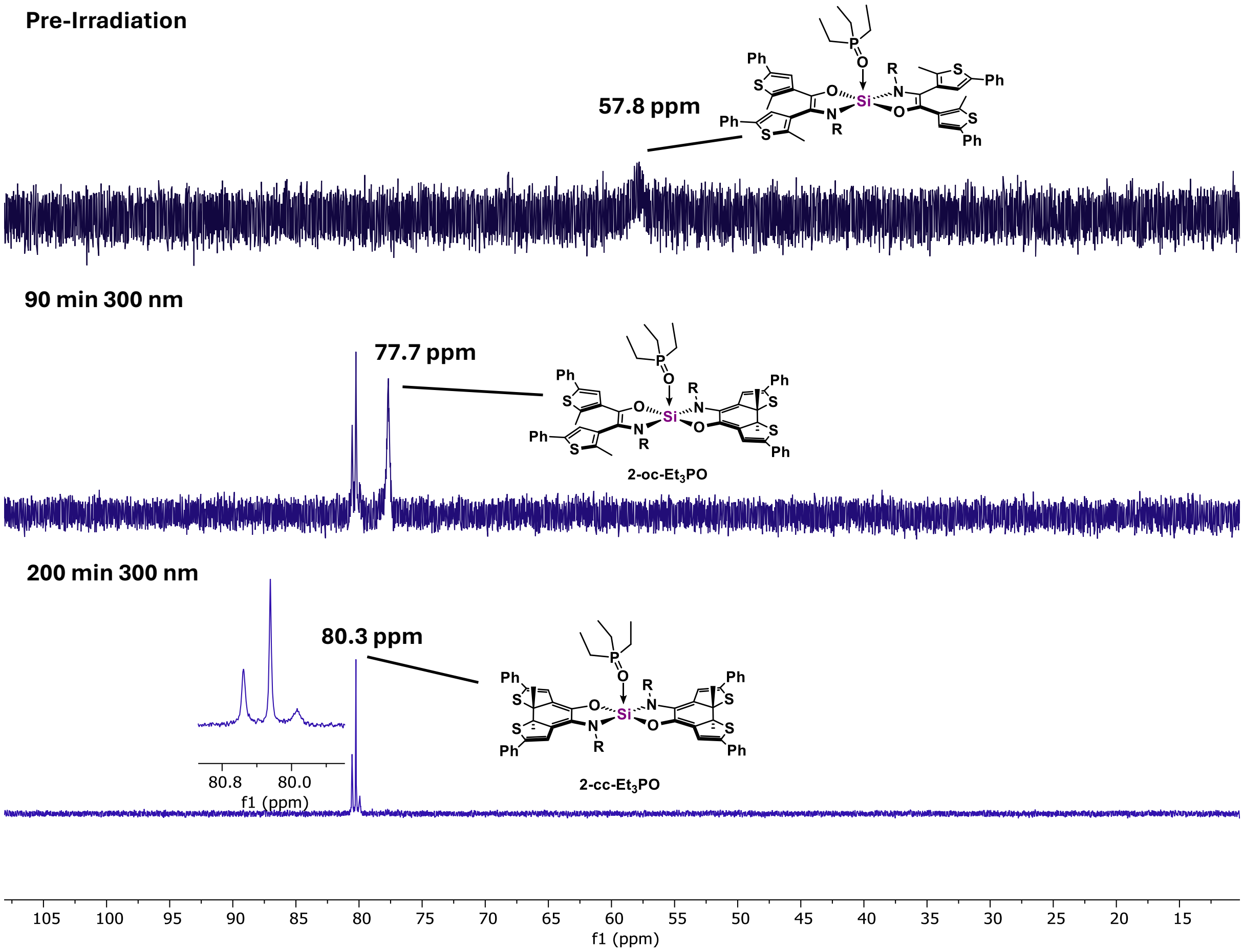

Figure S4.5. ^{31}P NMR (243 MHz, C_6D_6) spectra of **1** with 0.5 equivalents of Et_3PO before irradiation, after 90 minutes at 300 nm, and after 200 minutes at 300 nm.

2

For all isomers of **2**, no concentration dependence of the Gutmann-Beckett shift was observed, indicating that these values are measures of effective Lewis acidity.

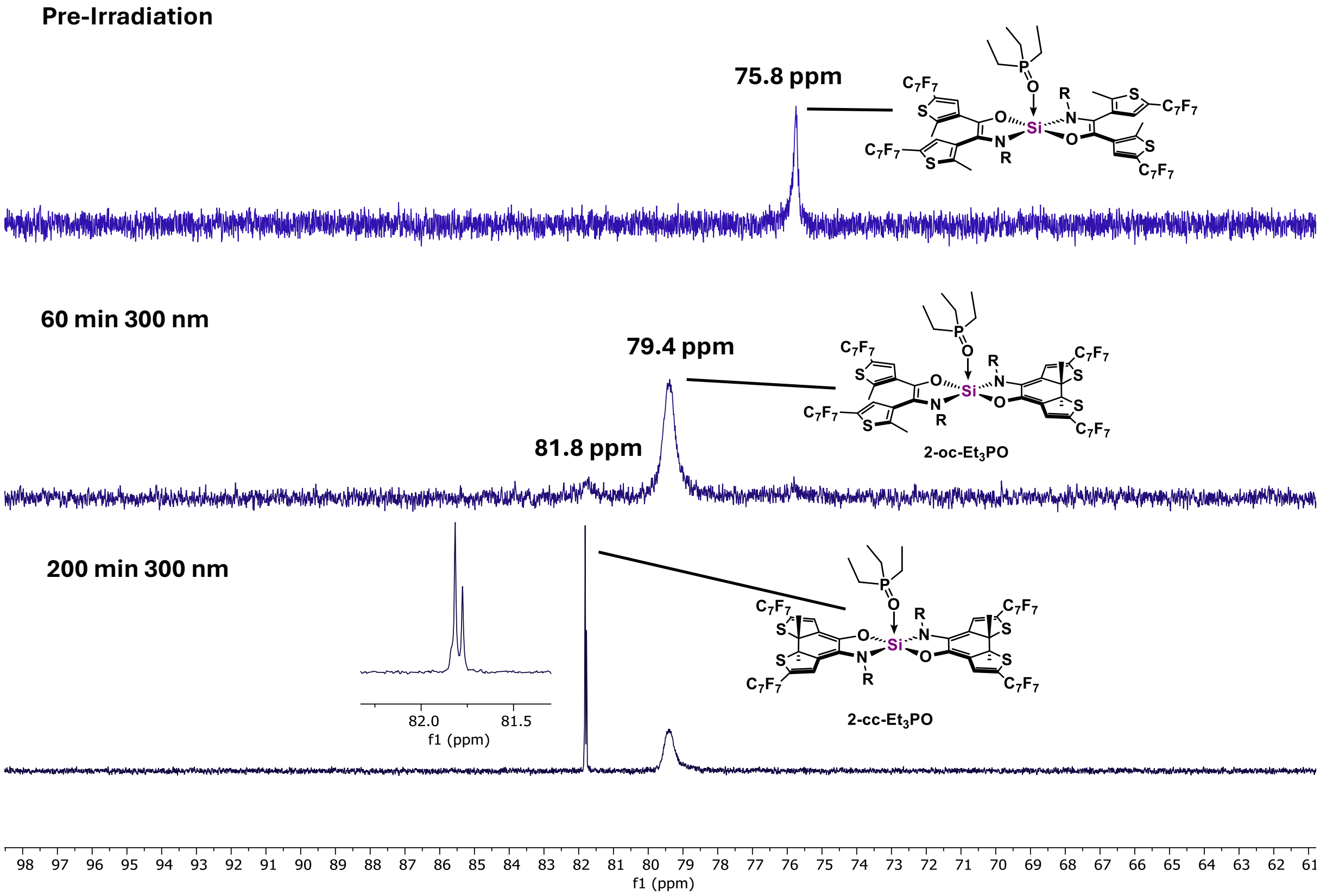

Figure S4.6. ^{31}P NMR (243 MHz, C_6D_6) spectra of **2** with 0.5 equivalents of Et_3PO before irradiation, after 60 minutes at 300 nm, and after 200 minutes at 300 nm.

4.4 Photocyclization of 2 with excess Et_3PO

2-oo (5.0 mg) and 2 equivalents of Et_3PO were mixed in C_6D_6. Quantification of **2-oc** and **2-cc** was done by integration against the CH_3 signal of Et_3PO. For **2-oc**, the characteristic upfield shifted CH proton of the closed ligand was integrated (1 proton). For **2-cc**, the CH protons at 6.15 ppm were integrated (2 protons).

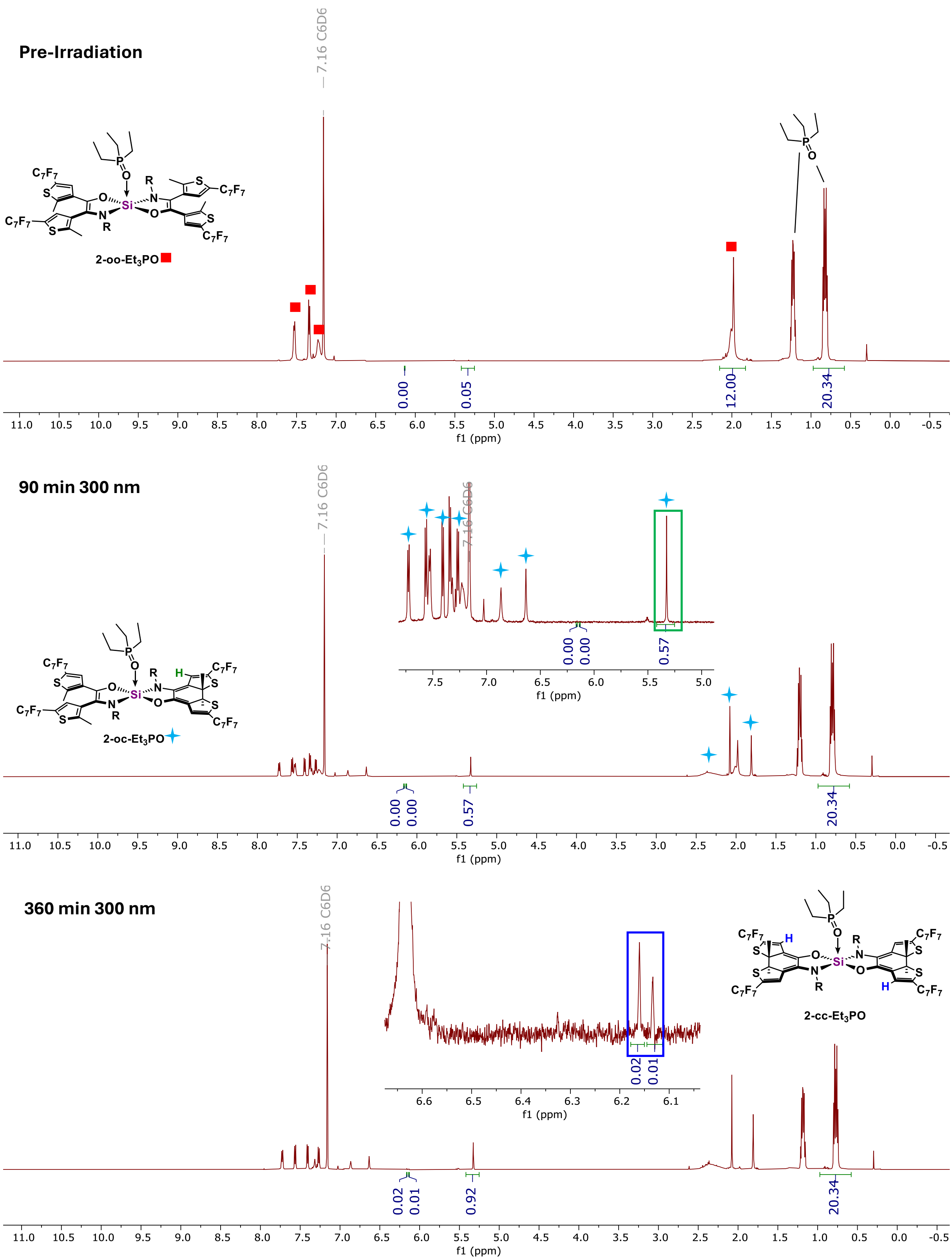

Figure S4.7. ^{1}H NMR (600 MHz, C_6D_6) spectra of **2** with 2 equivalents of Et_3PO before irradiation, after 90 minutes at 300 nm, and after 360 minutes at 300 nm.

Complete NMR spectra

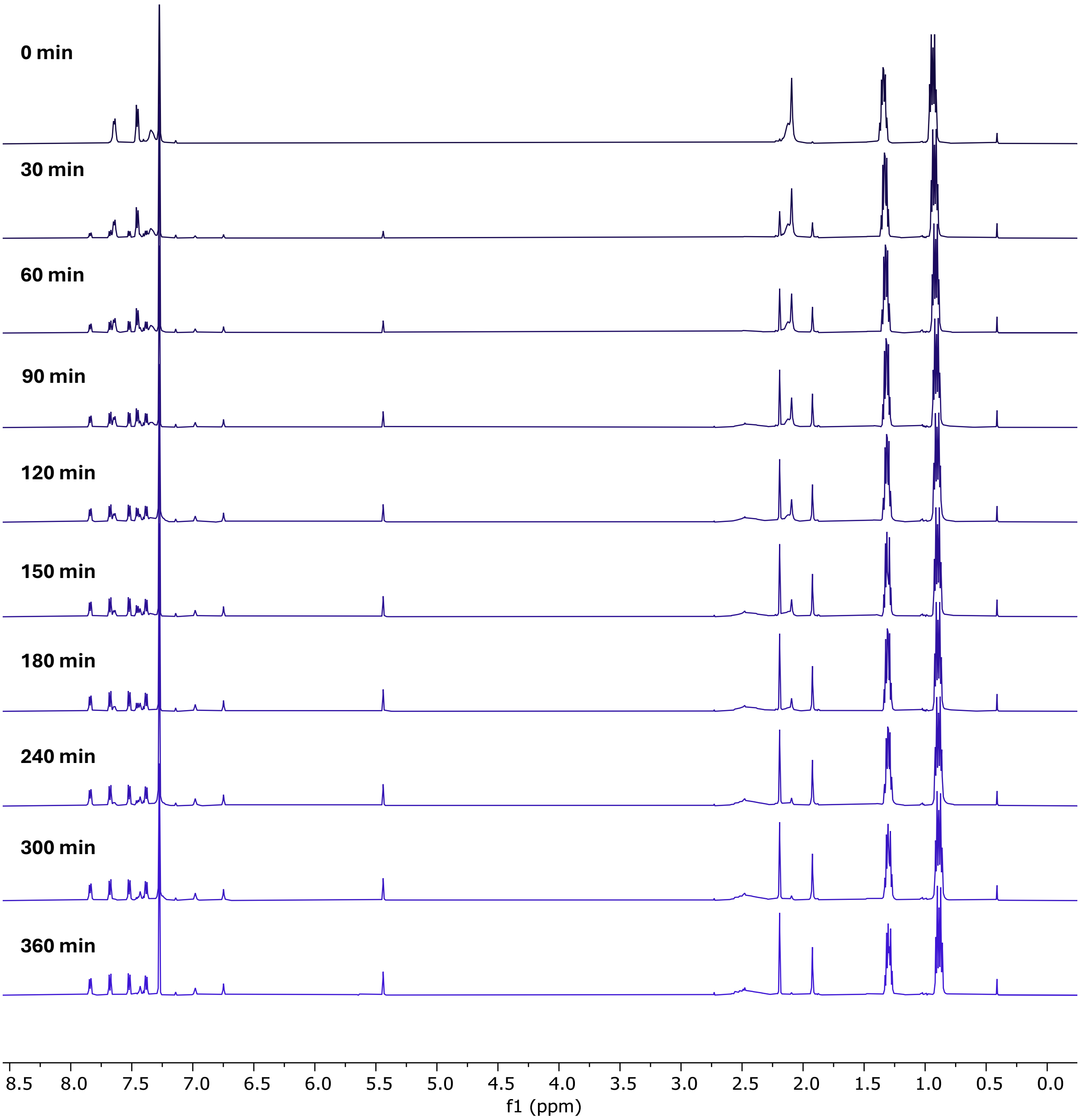

Figure S4.7. Stacked ^{1}H NMR (600 MHz, C_6D_6) spectra of **2** with 2 equivalents of Et_3PO before irradiation and with increasing irradiation time at 300 nm.

4.5 Photocyclization of 2-oo-Et$_3$PO at 460 nm and 300 nm

Photocyclization of 2-oo-Et$_3$PO at 460 nm

2-oo (5.3 mg) and 0.53 equivalents of Et$_3$PO were dissolved in 0.5 ml C_6D_6. The solution was irradiated at 460 nm for 10 minutes, giving 45% conversion to **2-oc-Et$_3$PO** (based on integration of characteristic CH proton of the closed ligand against Et$_3$PO signals). A 45 % overall conversion corresponds to an 85% conversion based on the available Et$_3$PO in solution. In the course of the irradiation, Et$_3$PO is abstracted from **2-oo**, resulting in the emergence of ^{1}H NMR resonances for the donor-free **2-oo** (marked below). No formation of **2-cc-Et$_3$PO** was observed. Longer irradiation times lead to no further changes. Irradiation at 620 nm restored the initial NMR spectrum, with some reduction in the signal intensity due to hydrolysis (resonances at 2.3 and 2.6 ppm can be assigned to hydrolysis products).

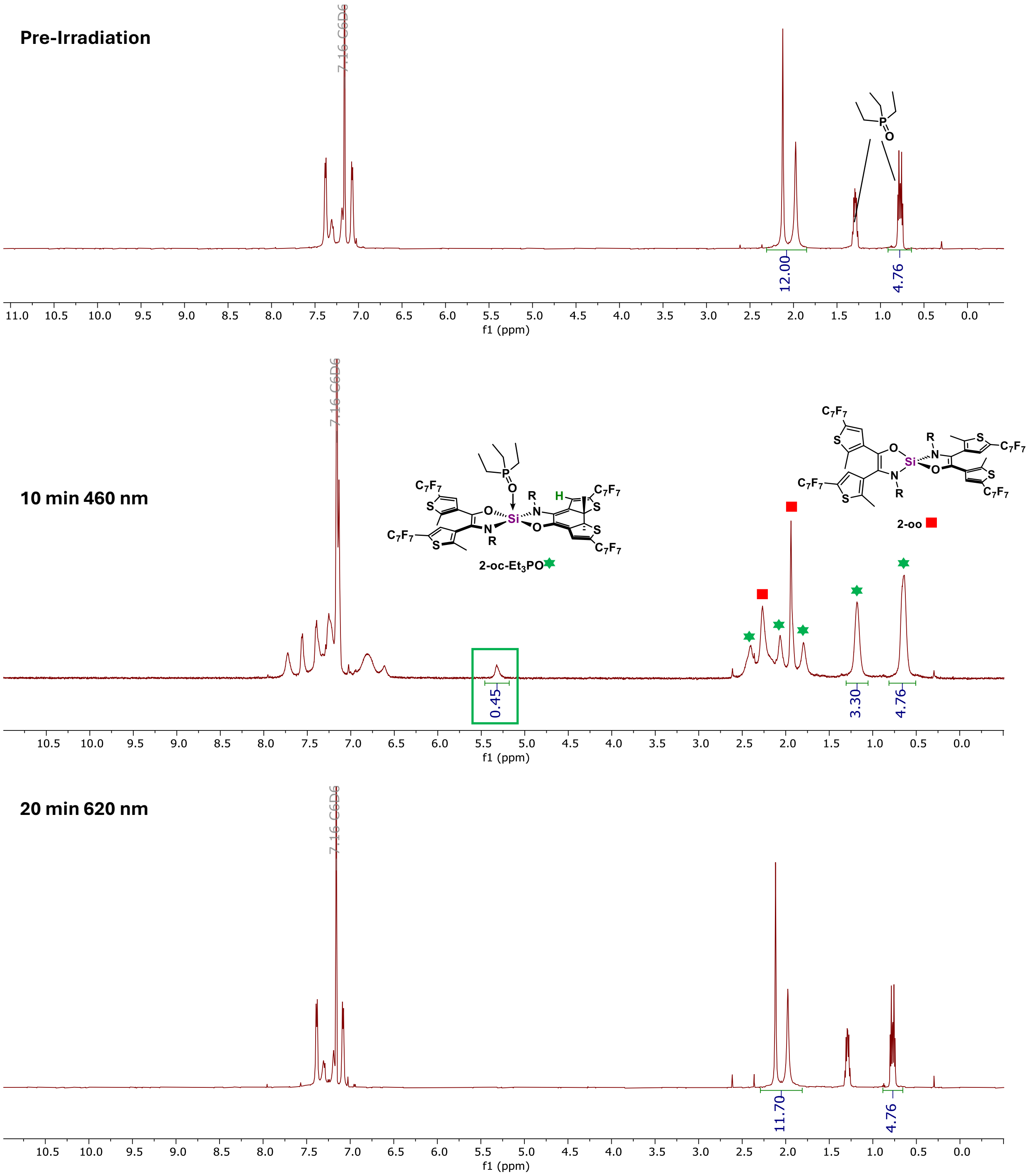

Figure S4.8. ^{1}H NMR (600 MHz, C_6D_6) spectra of **2** with 0.53 equivalents of Et$_3$PO before irradiation, after 10 minutes irradiation at 460 nm, and after 20 minutes irradiation at 620 nm.

Photocyclization of 2-oo-Et$_3$PO at 300 nm

2-oo (5.0 mg) and 0.44 equivalents of Et$_3$PO were dissolved in 0.5 ml C_6D_6. The solution was irradiated at 300 nm for 300 minutes, giving a mixture of **2-oc, 2-oc-Et$_3$PO,** and **2-cc-Et$_3$PO** as the photocyclization products. Complex signal patterns emerge in the ^{1}H NMR spectrum, but products remain identifiable by characteristic thiophene CH and Et$_3$PO signals, which are denoted below. An aliphatic impurity in the solvent is present and marked by ▲.

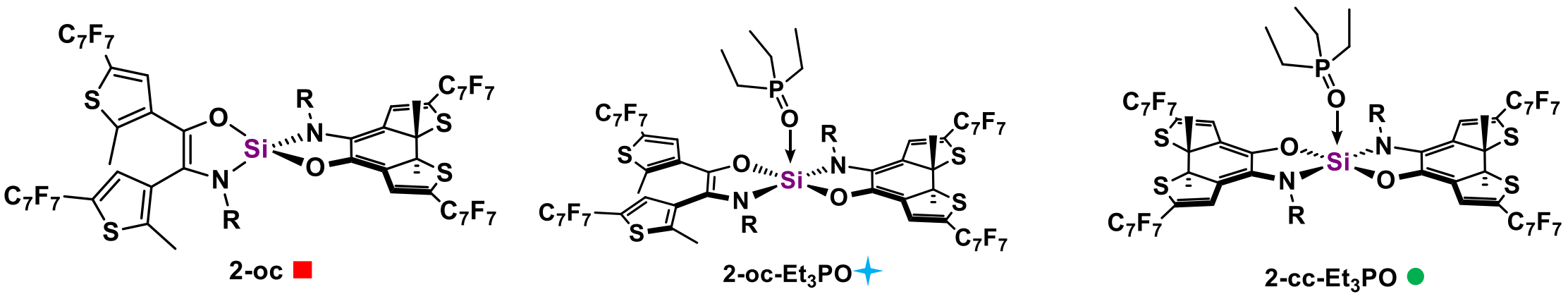

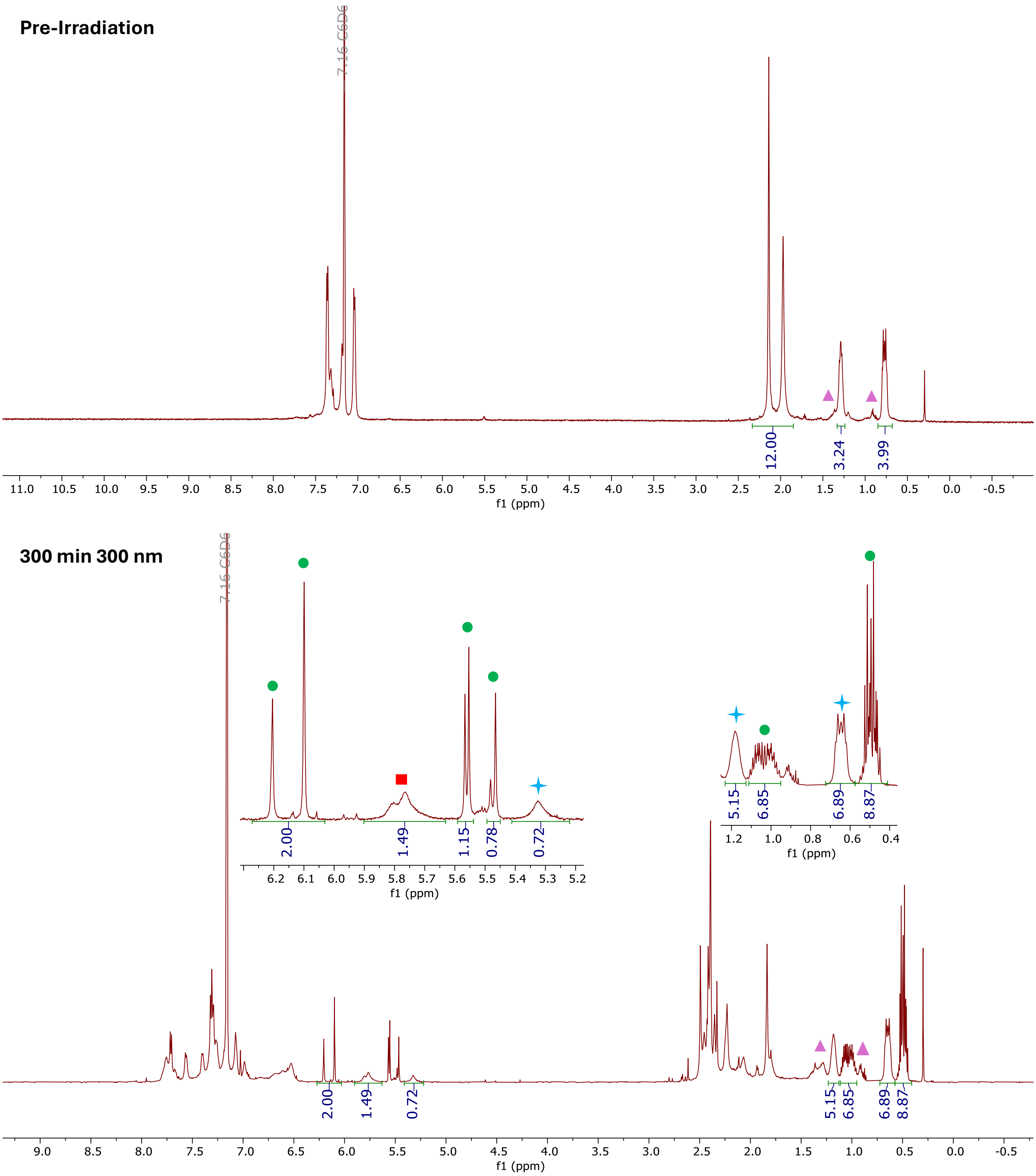

Figure S4.9. ^{1}H NMR (600 MHz, C_6D_6) spectra of **2** with 0.44 equivalents of Et$_3$PO before irradiation and after 5 hours irradiation at 300 nm.

4.6 Photochemistry of Fluoride Adduct [2-oo-F][PPh4]

2-oo (12.0 mg), and PPh_4F (2.7 mg, 1.05 eq) were dissolved in 0.5 ml C_6D_6, resulting in an orange solution. The mixture was analyzed by NMR spectroscopy, showing the formation of **[2-oo-F][PPh4]**. The mixture was irradiated at 460 nm for 10 minutes and subsequently at 300 nm for 1 hour, resulting in no color change and unchanged NMR spectra. Addition of $LiAl(O^FtBu)_4$ (1.5 eq) and sonication for 30 seconds resulted in the restoration of donor-free **2-oo**, which was irradiated at 300 nm giving a **2-oo/2-oc/2-cc** mixture.

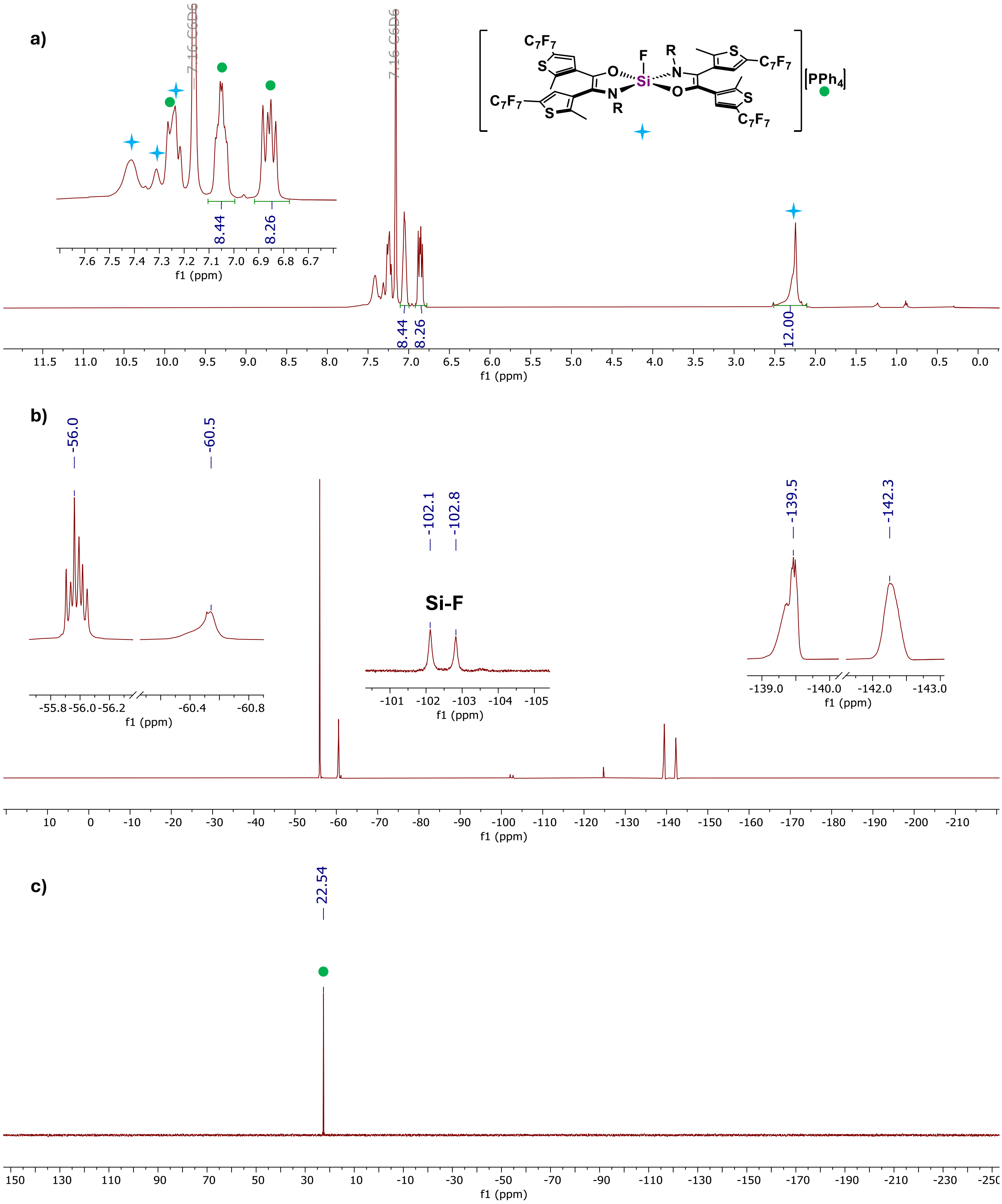

Figure S4.10. NMR spectra of **[2-oo-F][PPh4]**. a) ^{1}H NMR spectrum (400 MHz, C_6D_6). b) ^{19}F NMR spectrum (377 MHz, C_6D_6). Two Si-F resonances are observed due to diastereomers. A small impurity at -125 ppm is present, which might be due to hydrolysis or the bis-adduct due to excess PPh_4F. c) ^{31}P NMR spectrum (162 MHz, C_6D_6). d) ^{29}Si NMR spectrum (80 MHz, C_6D_6).

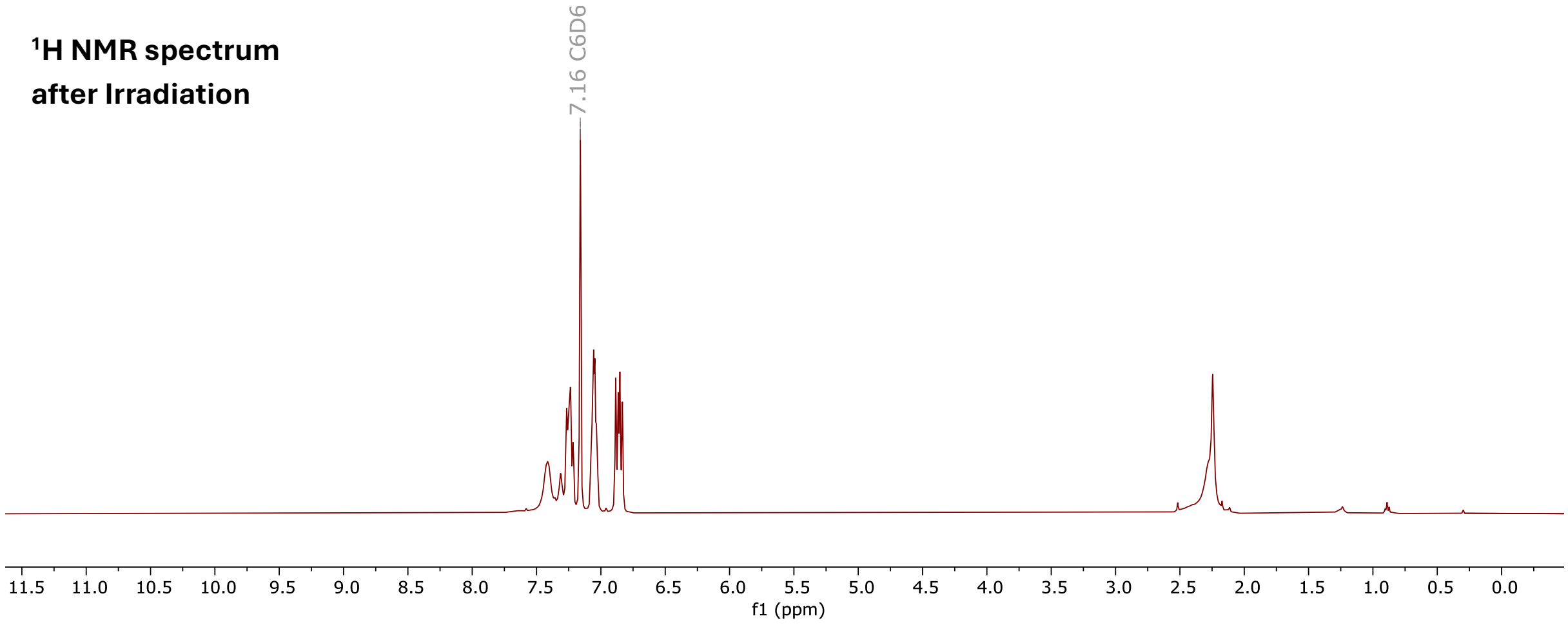

Figure S4.11. ^{1}H NMR spectrum (400 MHz, C_6D_6) of **[2-oo-F][PPh₄]** after irradiation at 460 nm for 10 minutes and 300 nm for 1 hour.

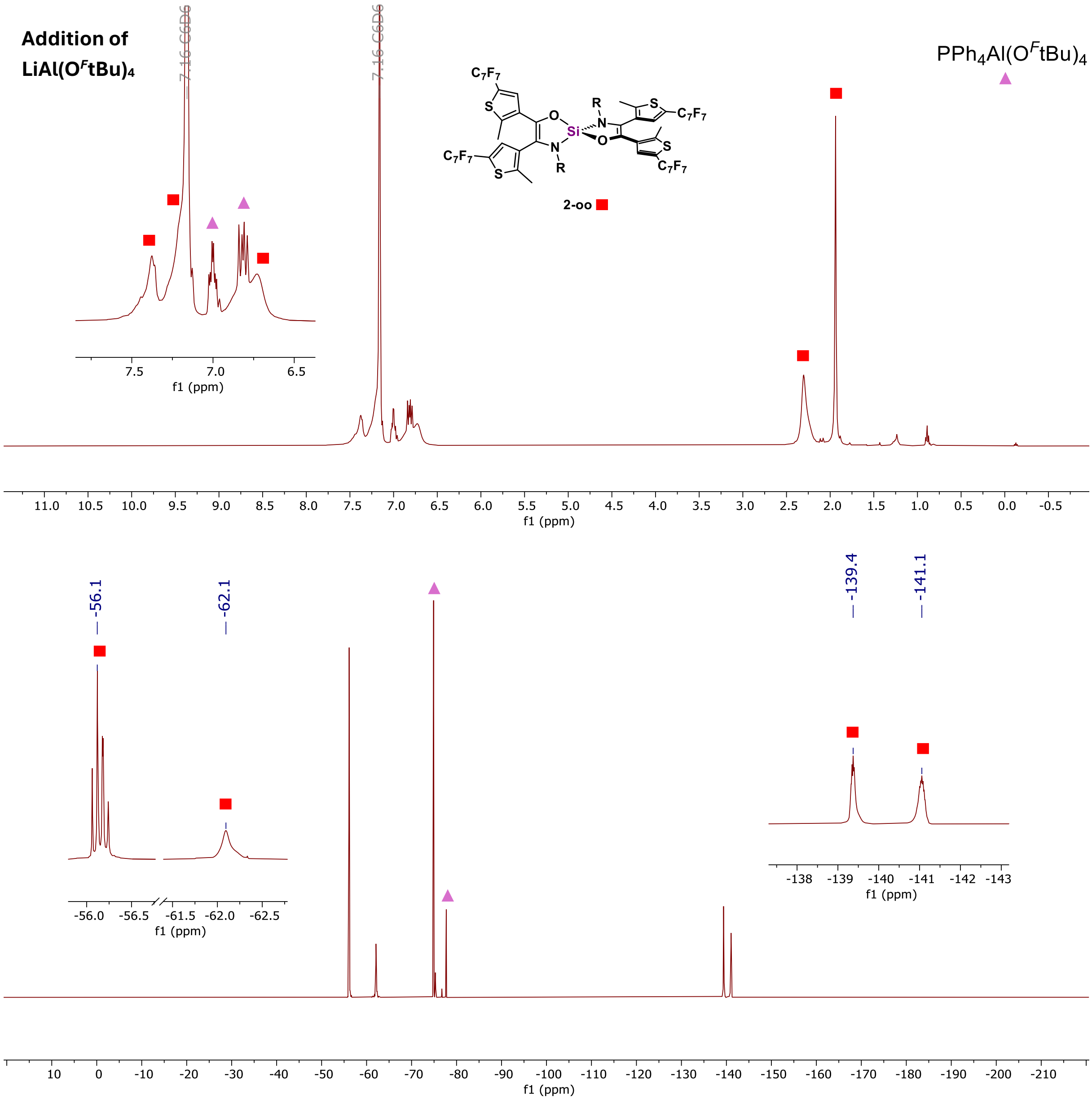

Figure S4.12. a) ^{1}H NMR spectrum (400 MHz, C_6D_6), and b) ^{19}F NMR spectrum (377 MHz, C_6D_6) after addition of LiAl(O^{F}tBu)₄ and sonication for 30 seconds. A small amount of [PPh₄][Al(O^{F}tBu)₄] remains dissolved in C_6D_6.

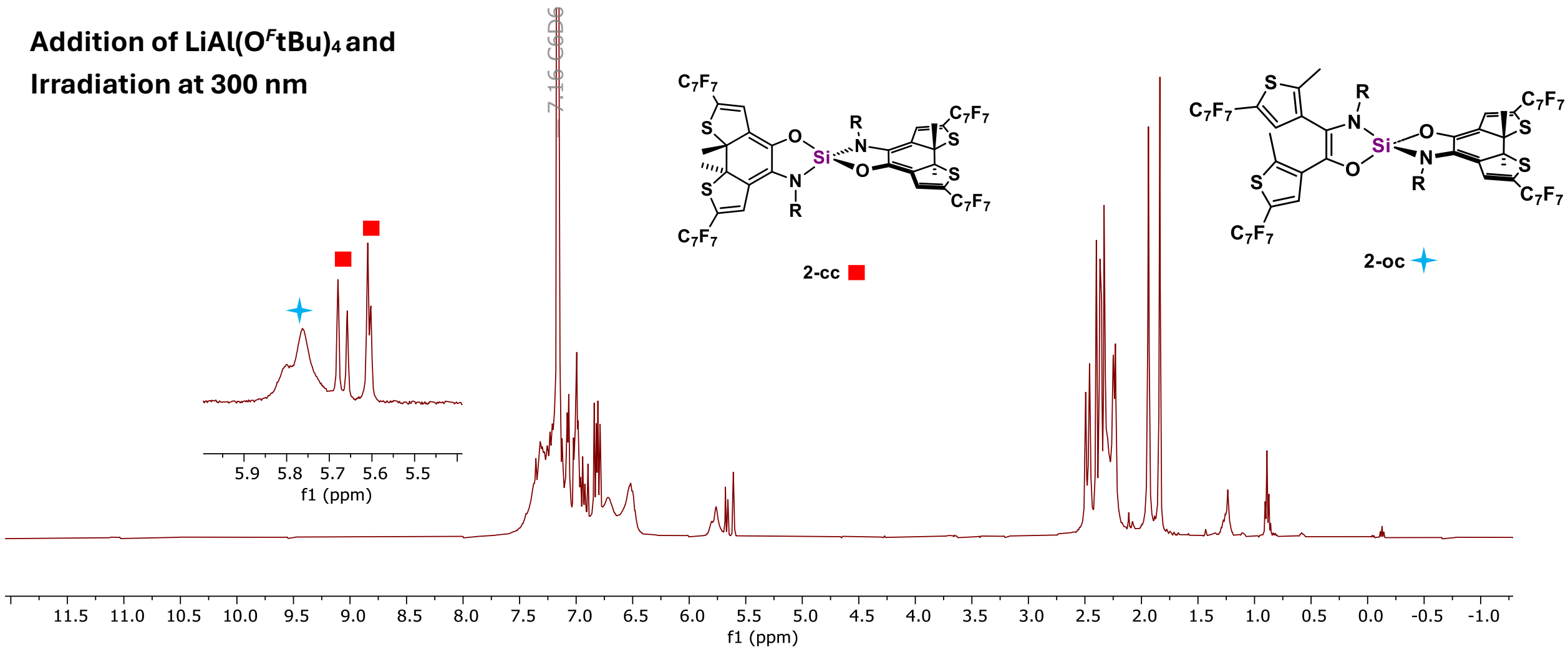

Figure S4.13. ^{1}H NMR spectrum (400 MHz, C_6D_6) after addition of LiAl(O^{F}tBu)$_4$ and irradiation at 300 nm for 2 hours.

4.7 Ligand Redistribution

2 + Et_3PO

A 100:2 mixture of **2-oc/2-cc** with excess Et_3PO was heated to 60 °C overnight. The ratio changed to 100:15, while the amount of total closed ligands remained unchanged. Ligand rearrangement from **2-oc-Et_3PO** to **2-cc-Et_3PO** also results in an increase of the amount of **2-oo-Et_3PO** (methyl resonances marked below).

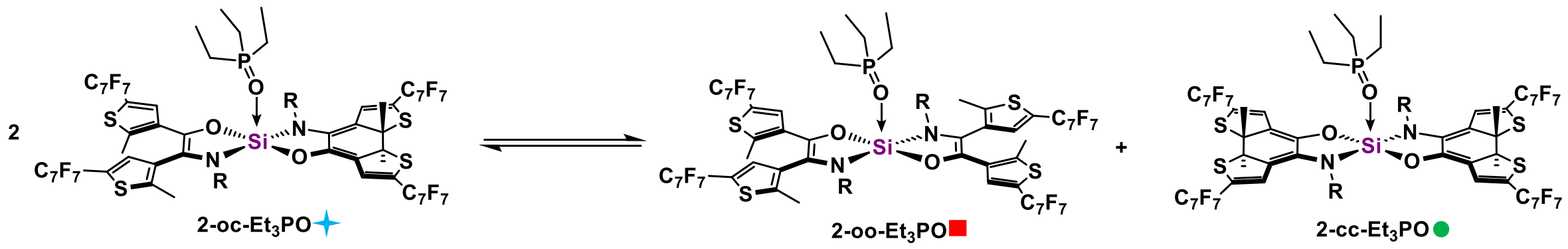

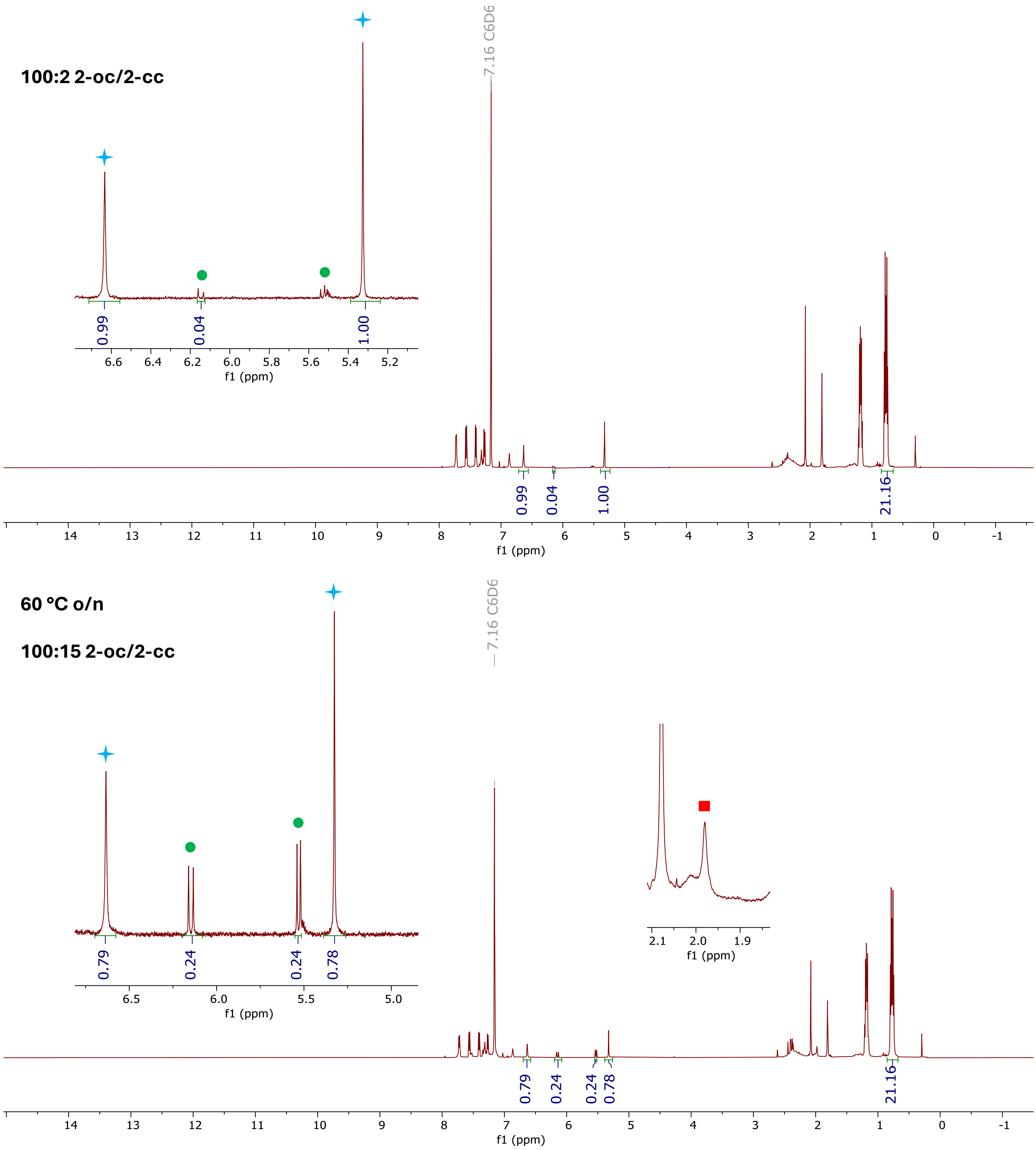

Figure S4.14. ^{1}H NMR (600 MHz, C_6D_6) spectra of **2-cc/2-oc/2-oo** with excess Et_3PO before and after heating to 60 °C overnight.

1 + pyridine

2-oo (3.6 mg) was dissolved in 0.5 ml C_6D_6. The mixture was irradiated at 300 nm for 2 hours, resulting in a solution with a **1-oo/1-oc/1-cc** ratio of 35/48/17. Pyridine (5 equivalents) was added and the rearrangement was followed by ^{1}H NMR spectroscopy over the next 21 hours. Integration of the characteristic CH protons was done against the pyridine signals.

Note: assignment as pyridine adducts or donor free species below was done for clarity and to emphasize the driving force of the rearrangement. All species are in dynamic equilibrium with their respective pyridine adducts to different extents.

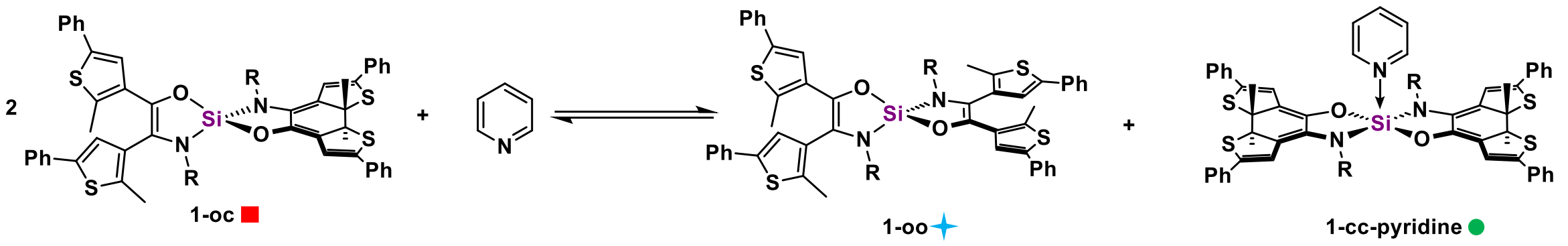

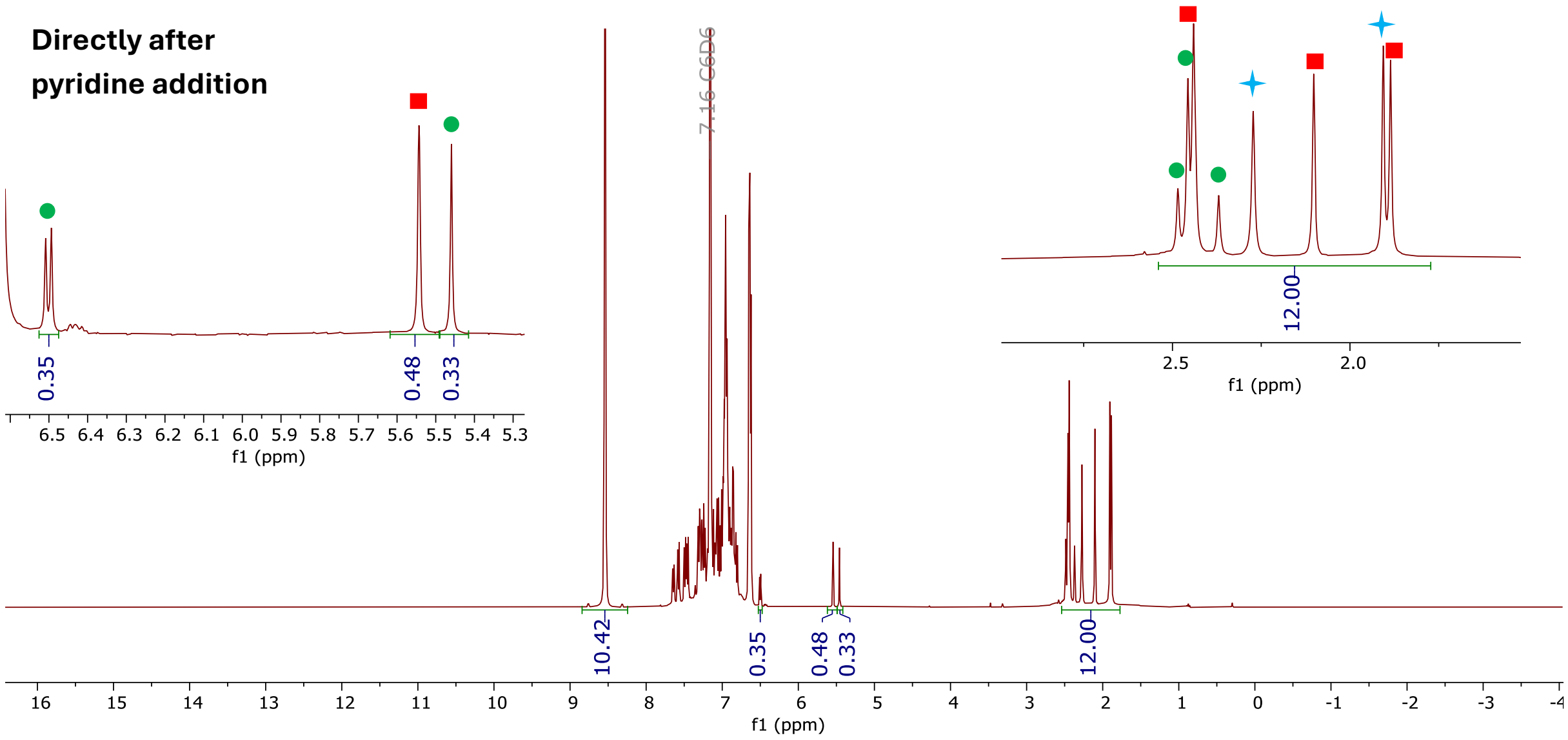

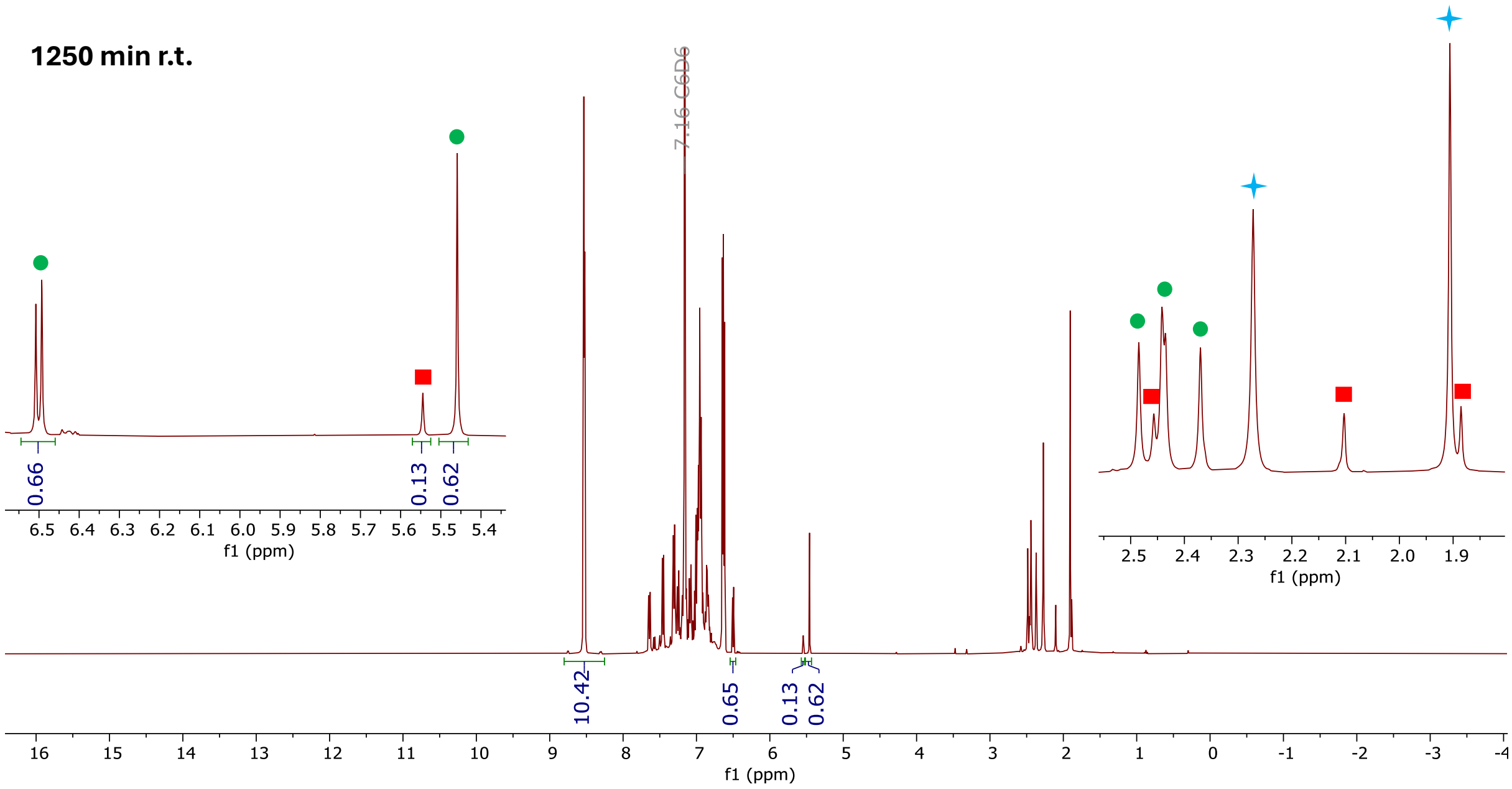

Figure S4.15. ^{1}H NMR (600 MHz, C_6D_6) spectra for the rearrangement of **1-oc** to **1-cc** and **1-oo** in the presence of pyridine, directly after addition and after 1250 minutes at room temperature.

Complete NMR spectra

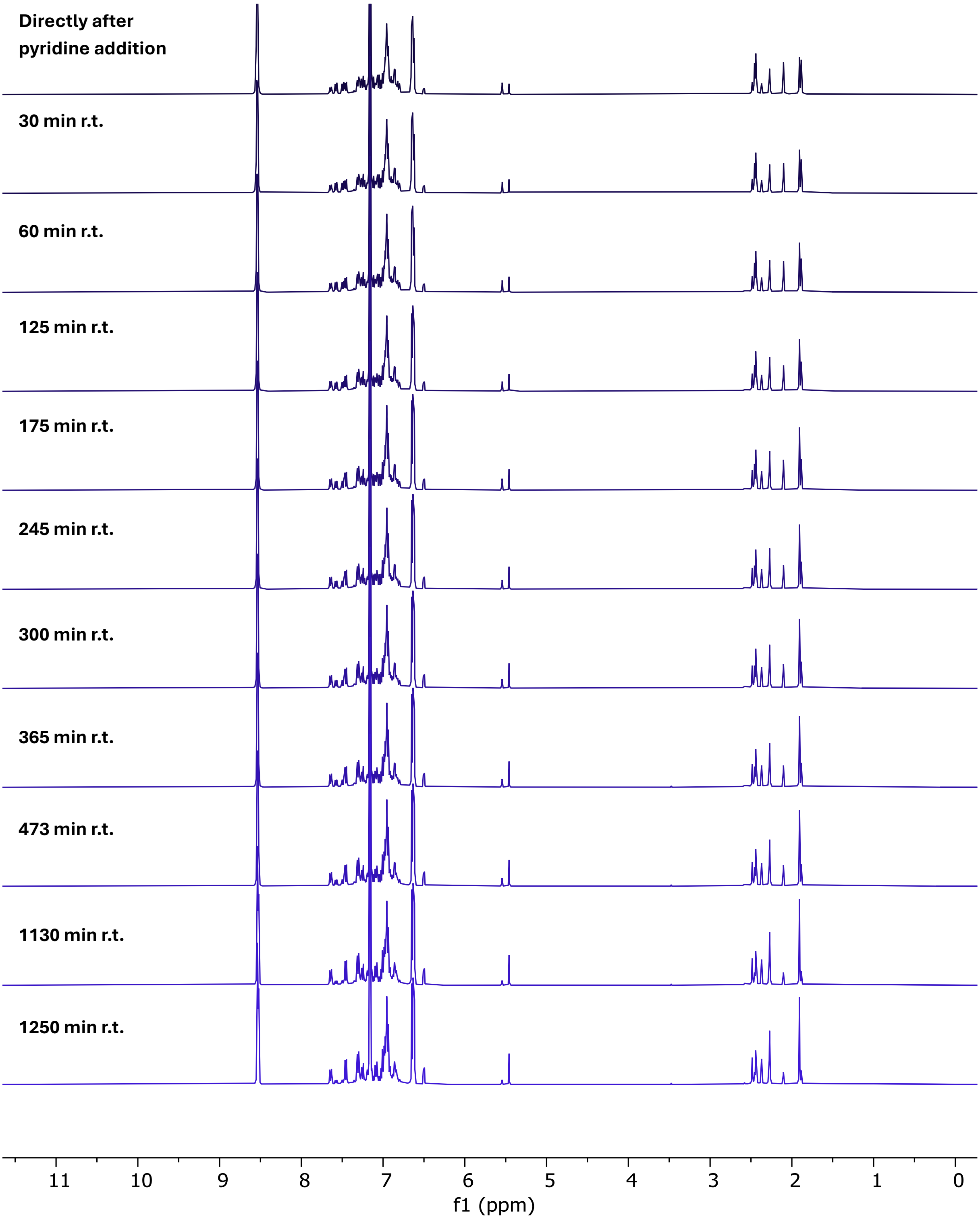

Figure S4.16. Stacked ^{1}H NMR (600 MHz, C_6D_6) spectra for the rearrangement of **1-oc** to **1-cc** and **1-oo** in the presence of pyridine, from 0 to 1250 minutes.

In the course of the rearrangement, the pyridine signals are shifted slightly as a larger proportion is bound to **1-cc**.

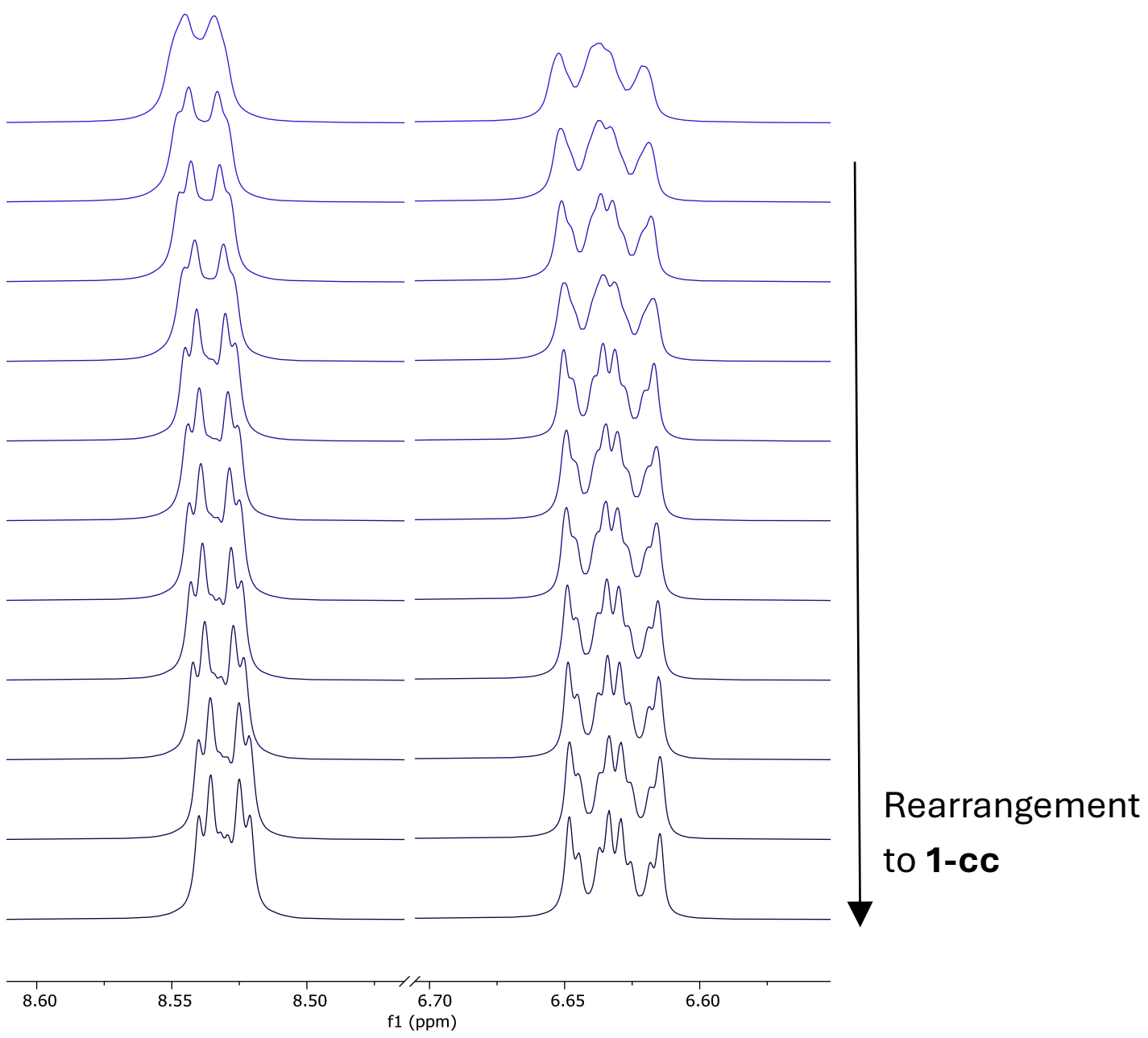

Figure S4.17. Spectral shift of pyridine ortho and meta protons during rearrangement to **1-cc**, from 0 to 1250 minutes.

4.8 Thermal Ring-Opening

1

A mixture of **1-oo/1-oc/1-cc** (obtained by irradiation of **1-oo** at 300 nm) and cyclohexane as internal standard was heated to 50 °C, 60 °C, or 80 °C. The concentration of **1-cc** and **1-oc** (as c/c_0) at each time step was determined by internal integration of signals at 2.41 (**1-cc**) and 2.50 ppm (**1-oc**). Exemplary NMR spectra for ring-opening at 50 °C are given below. For the reaction with added methanol, two identical samples were prepared, irradiated at 300 nm, and the cycloreversion at 60 °C was followed with 5-mol% methanol added to one of the samples.

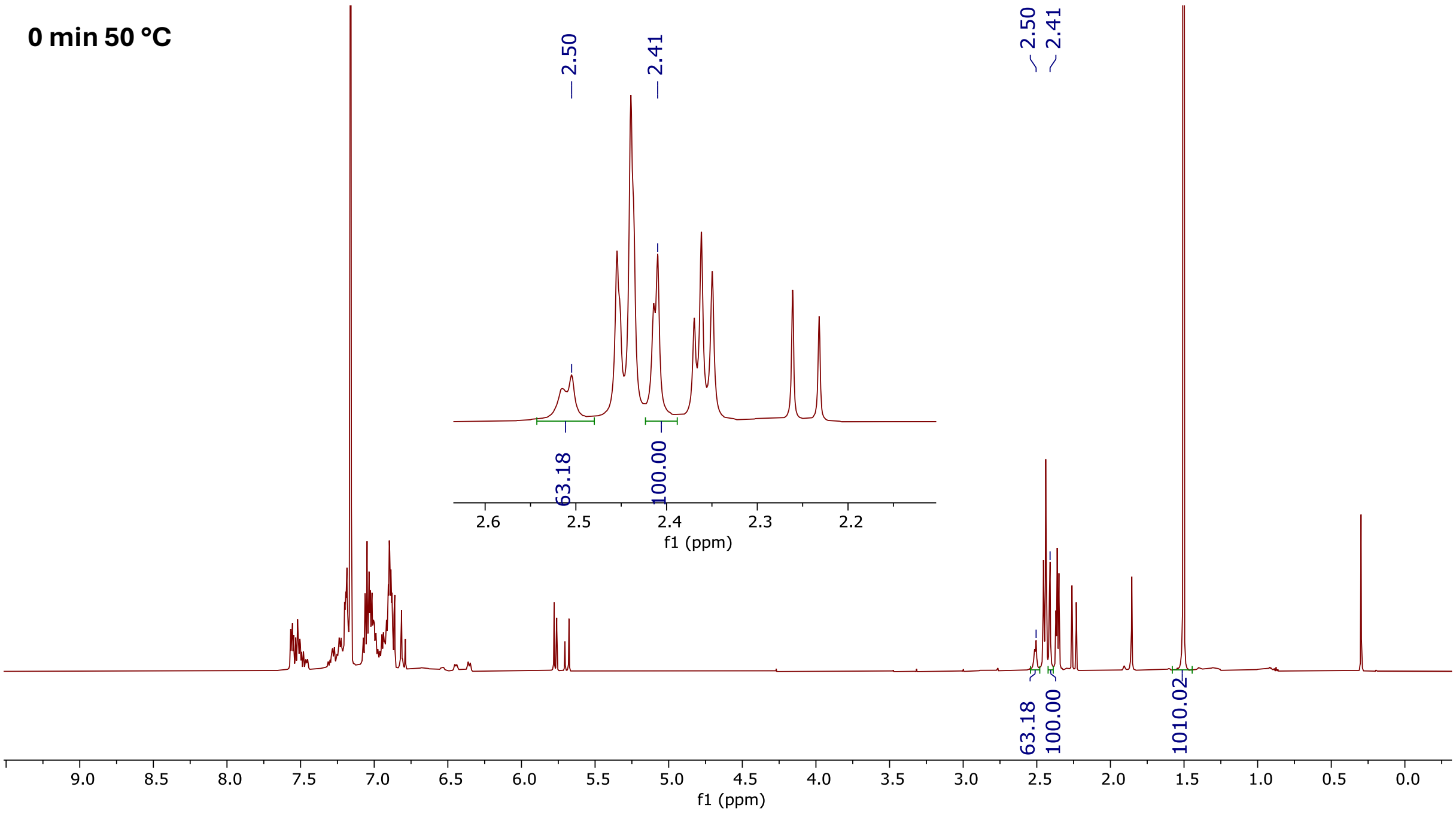

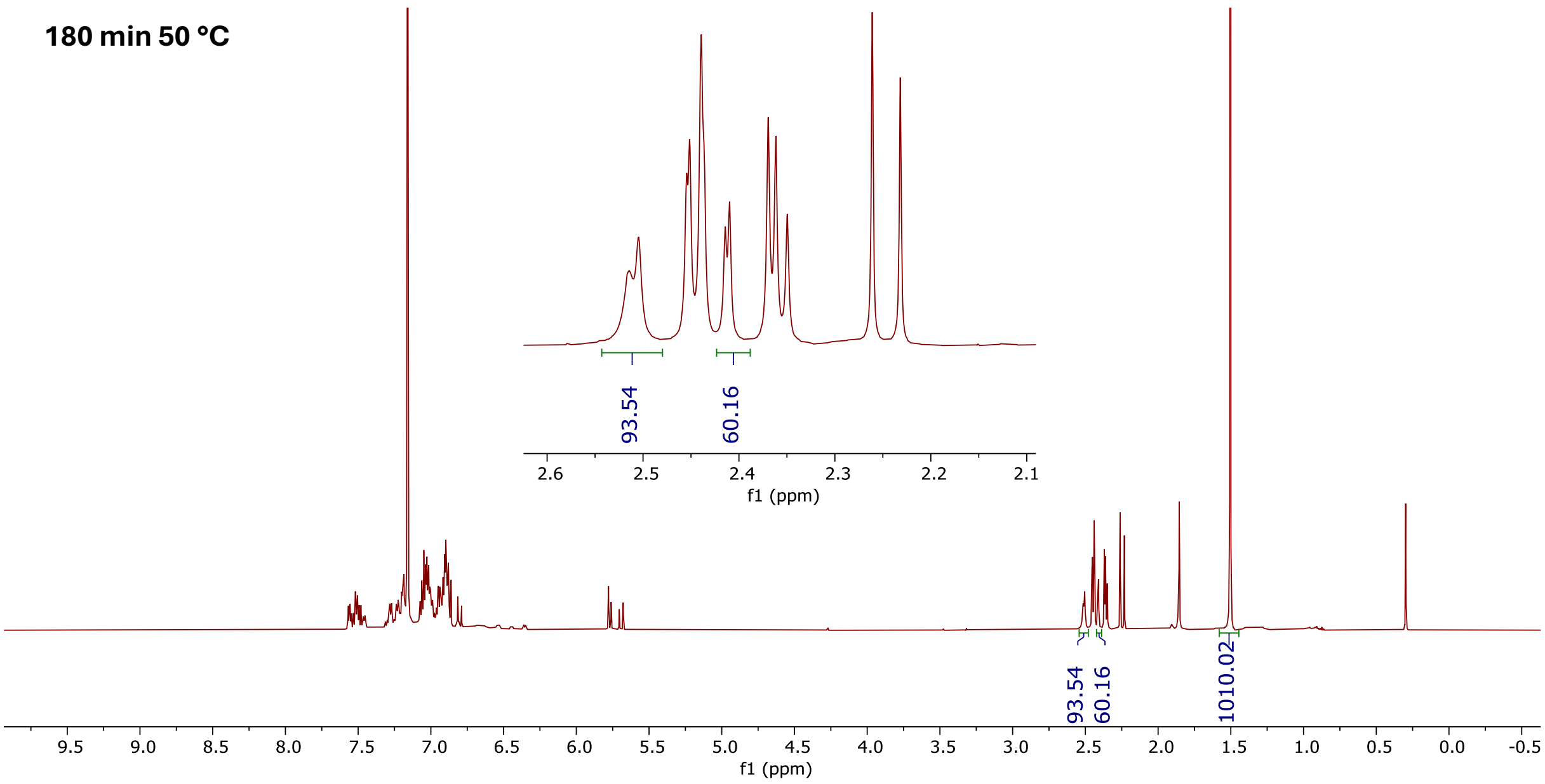

Figure S4.18. ¹H NMR (600 MHz, C_6D_6) spectra for the thermal ring-opening of **1-cc** at 50 °C, before heating and after 180 minutes.

Temperature Dependence (1-cc)

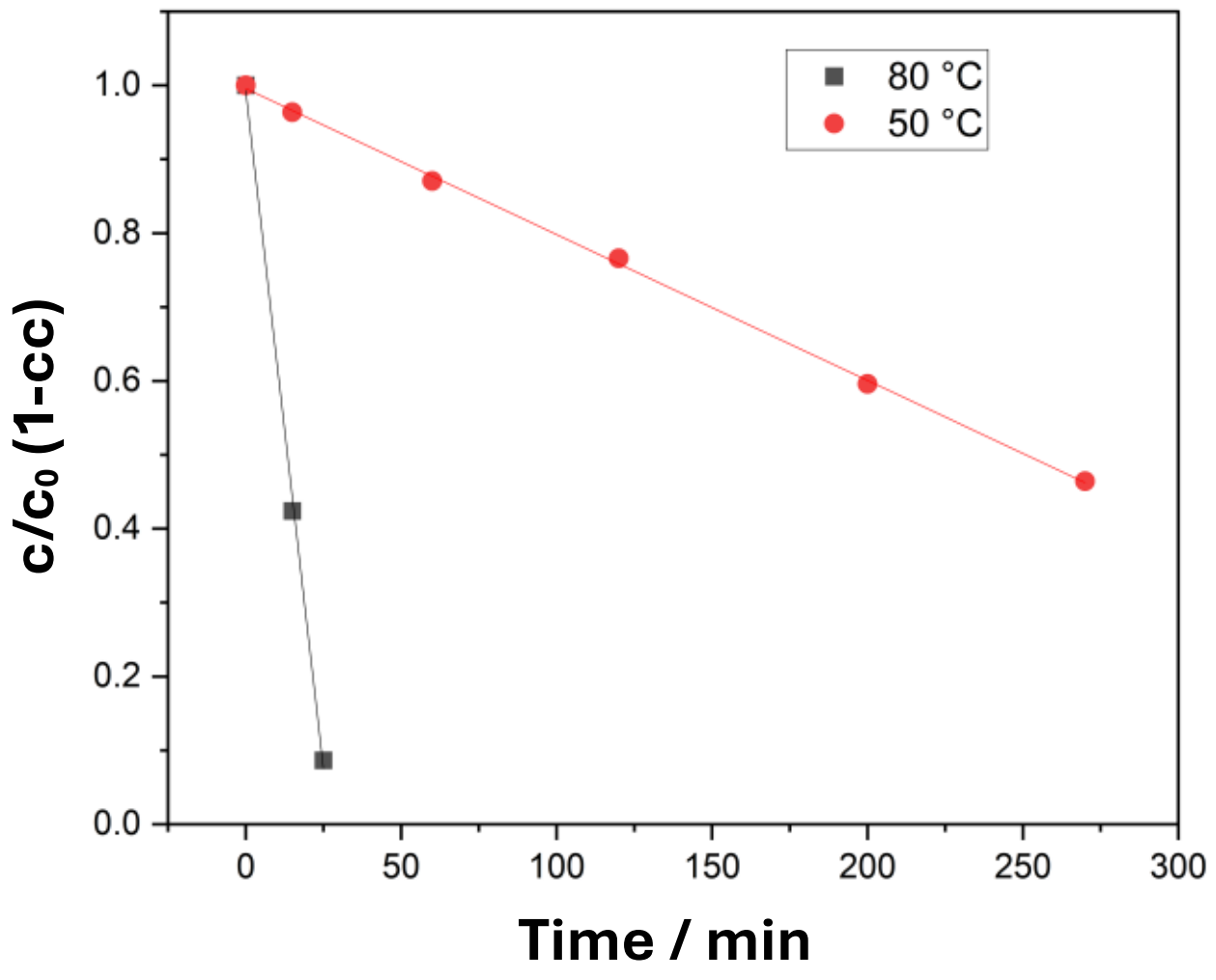

Figure S4.19. Decay of **1-cc** (c/c_0) at 50 °C and 80 °C.

Concentration and Additive Dependence (1-cc)

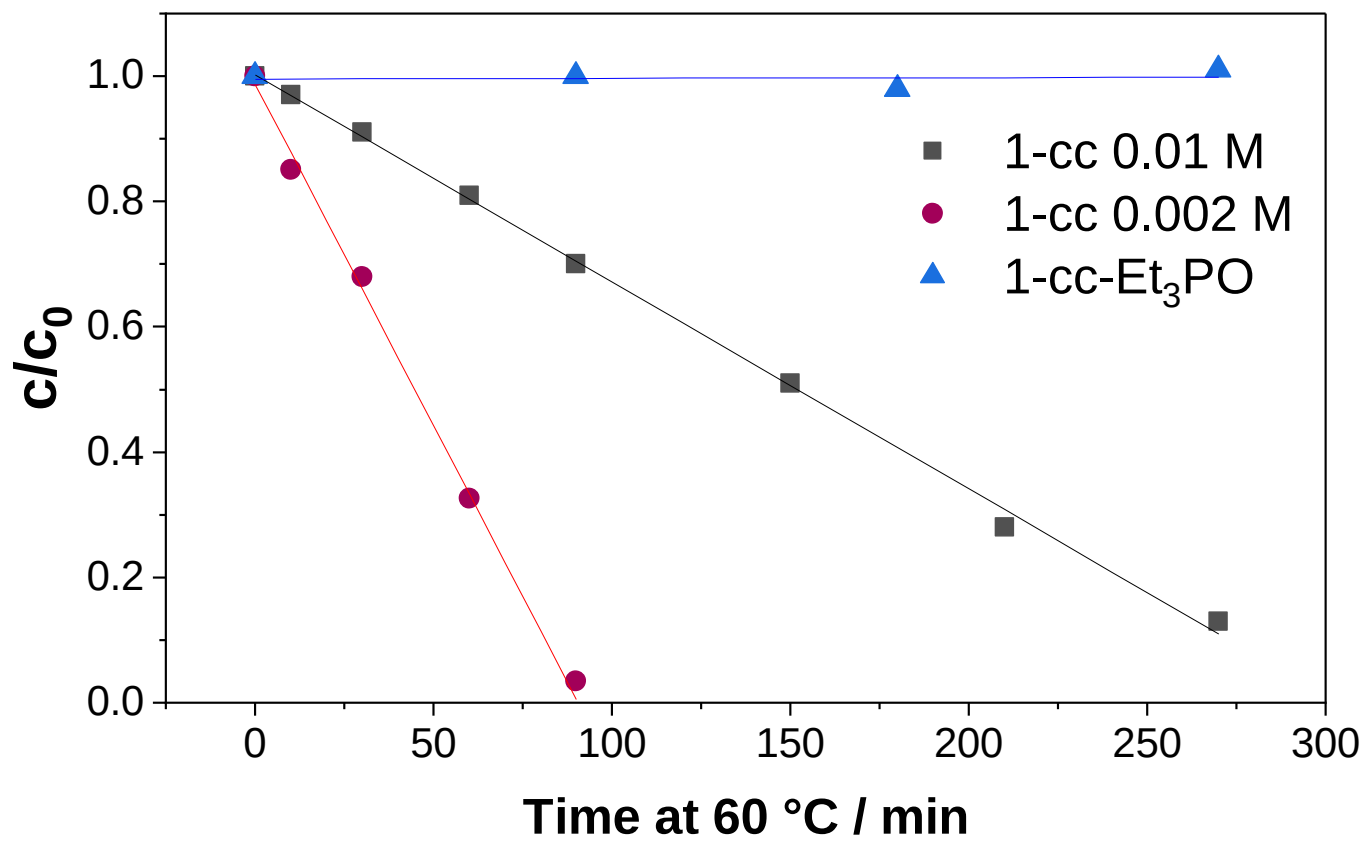

Figure S4.20. Decay of **1-cc** (c/c_0) at 0.01M concentration, 0.002M concentration, and for **1-cc-Et$_3$PO** at 60 °C .

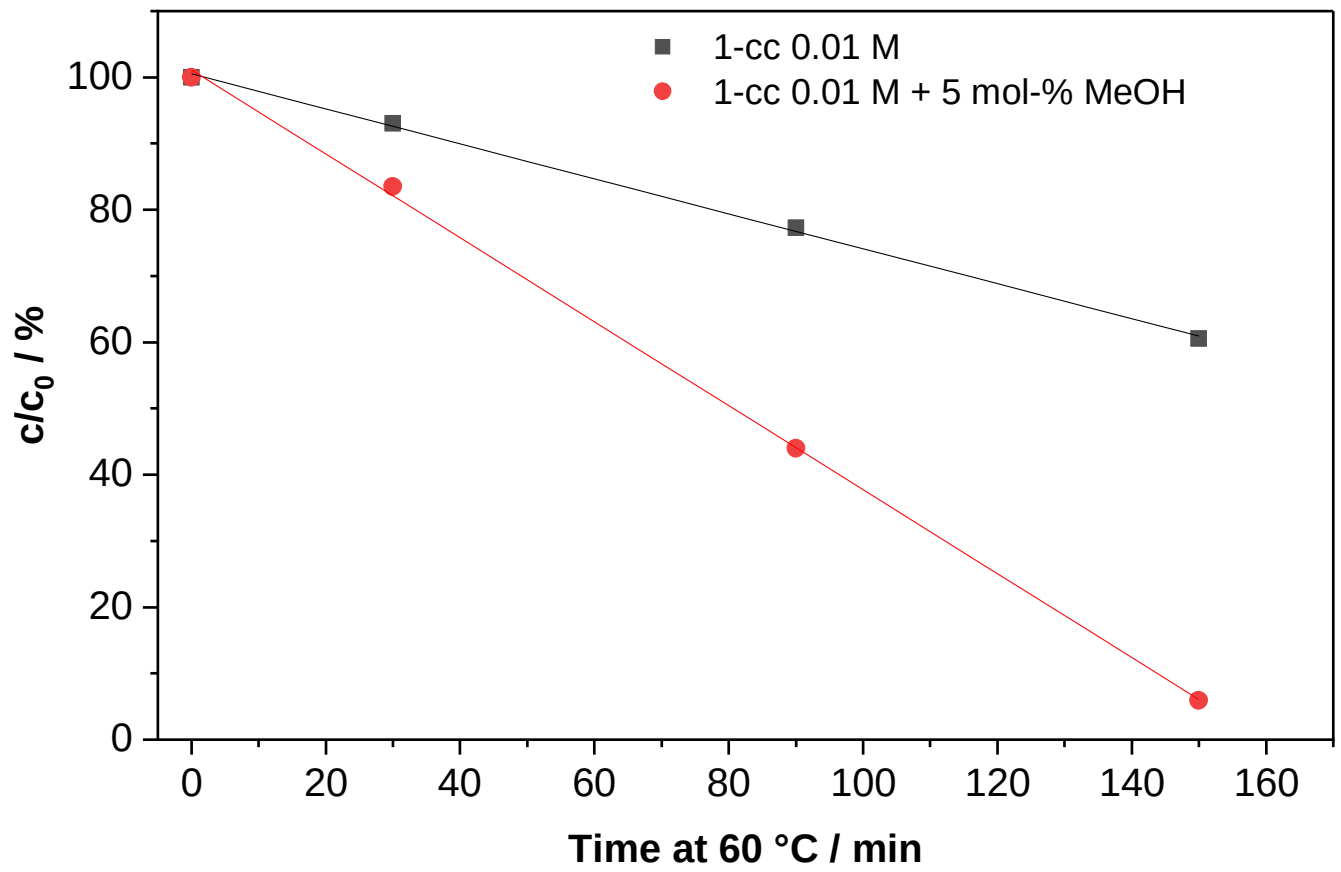

Figure S4.21. Decay of **1-cc** (c/c_0) at 0.01 M concentration and 60 °C, with and without 5 mol-% methanol added.

Stepwise Ring-Opening (1-cc and 1-co at 0.002 M concentration)

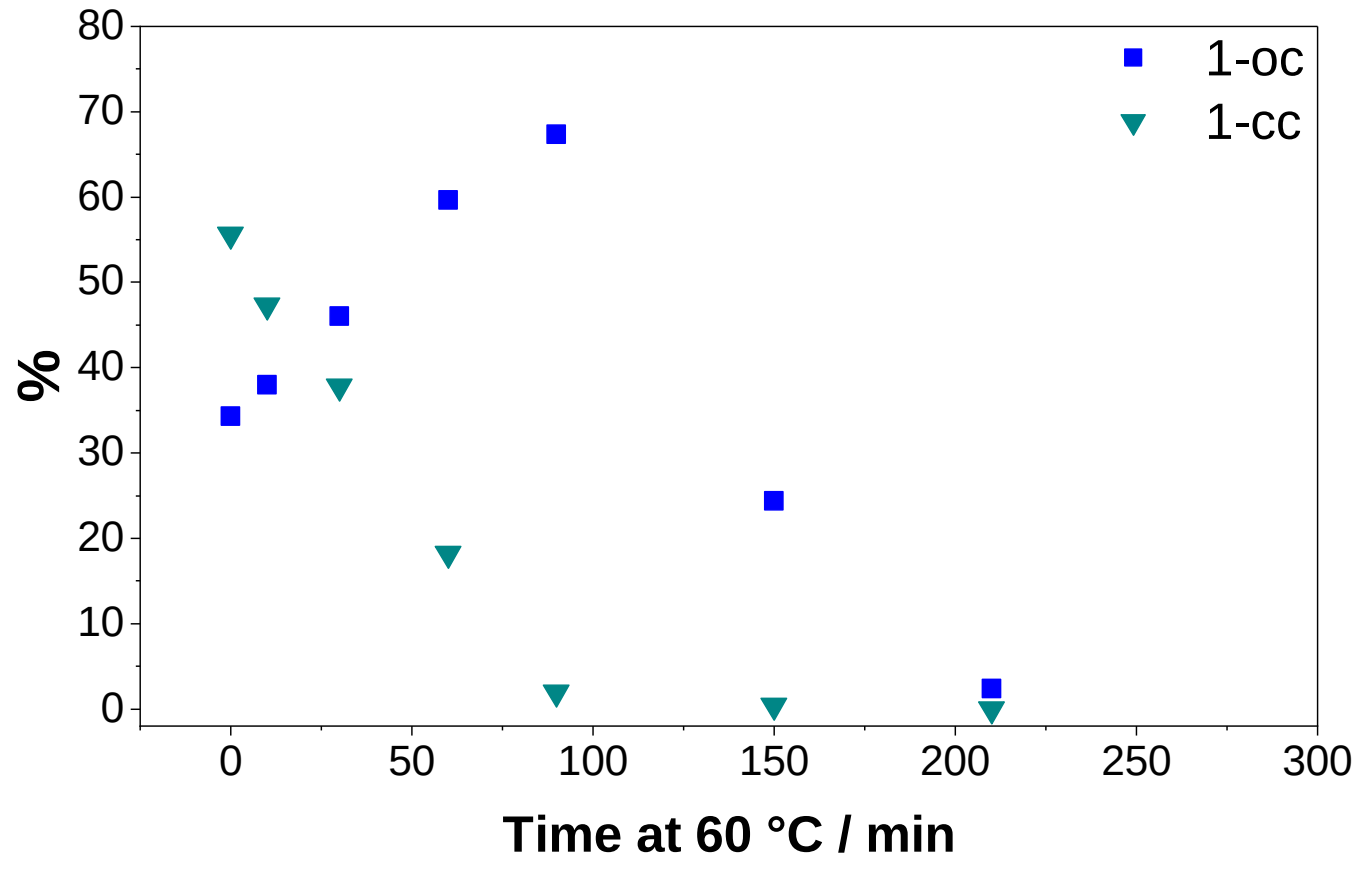

Figure S4.22. Ratios of **1-cc** and **1-oc** (% of total) at 0.002M concentration during heating to 60 °C.

2

For **2**, the ring-opening reaction was followed at room temperature. Because of stronger signal overlap in the methyl region, the CH protons at 5.60 ppm were integrated against cyclooctane instead to obtain c/c_0 for **2-cc**. Exemplary NMR spectra are given below. An uneven decay of the signals of **2-cc** was observed. This can be attributed to slight differences in Lewis acidity between diastereomers of **2-cc,** leading to preferential binding of the catalyst (water) to one of the diastereomers.

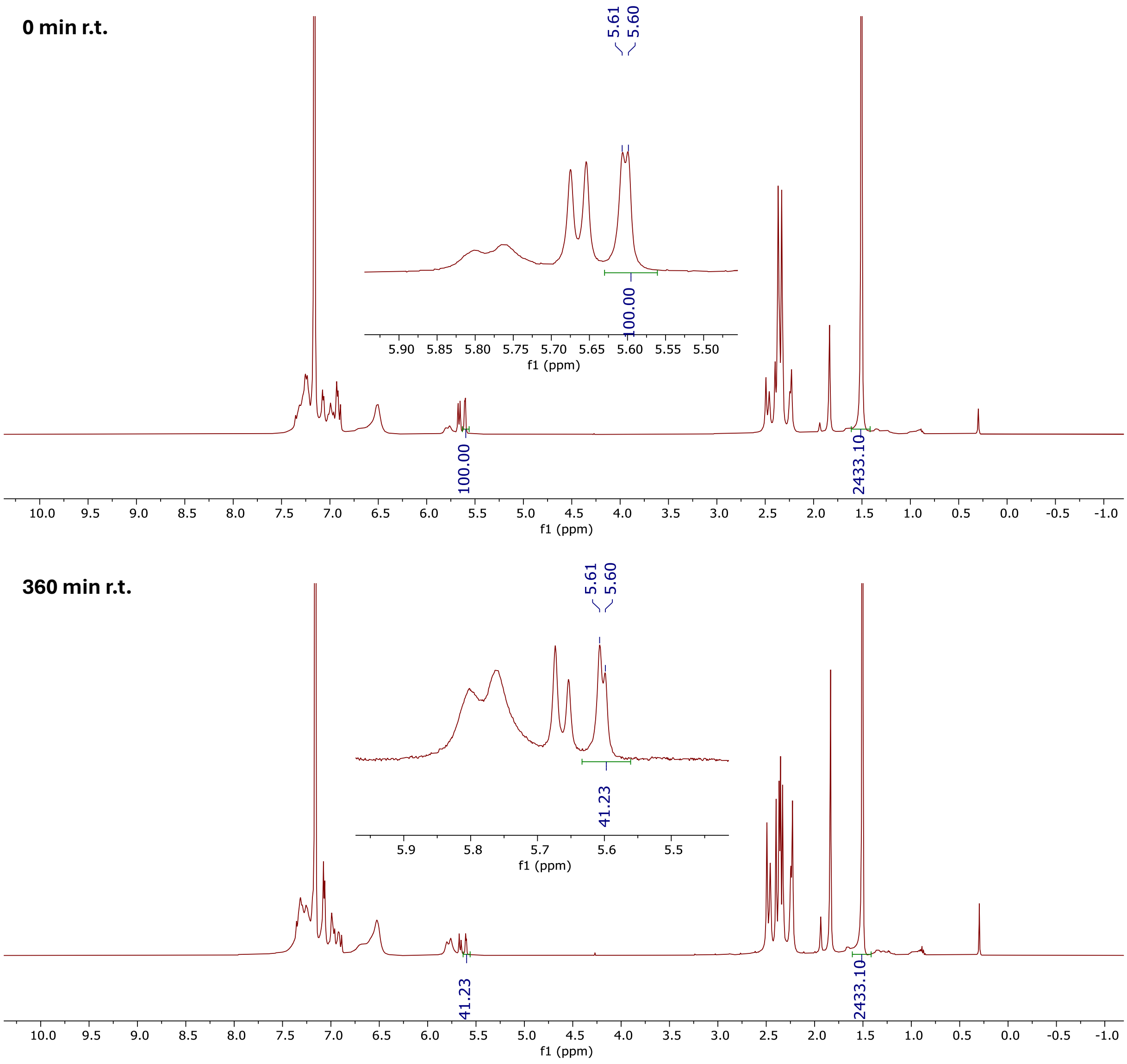

Figure S4.23. ^{1}H NMR (400 MHz, C_6D_6) spectra for the thermal ring-opening of **2-cc** at room temperature, directly after irradiation and after 6 hours.

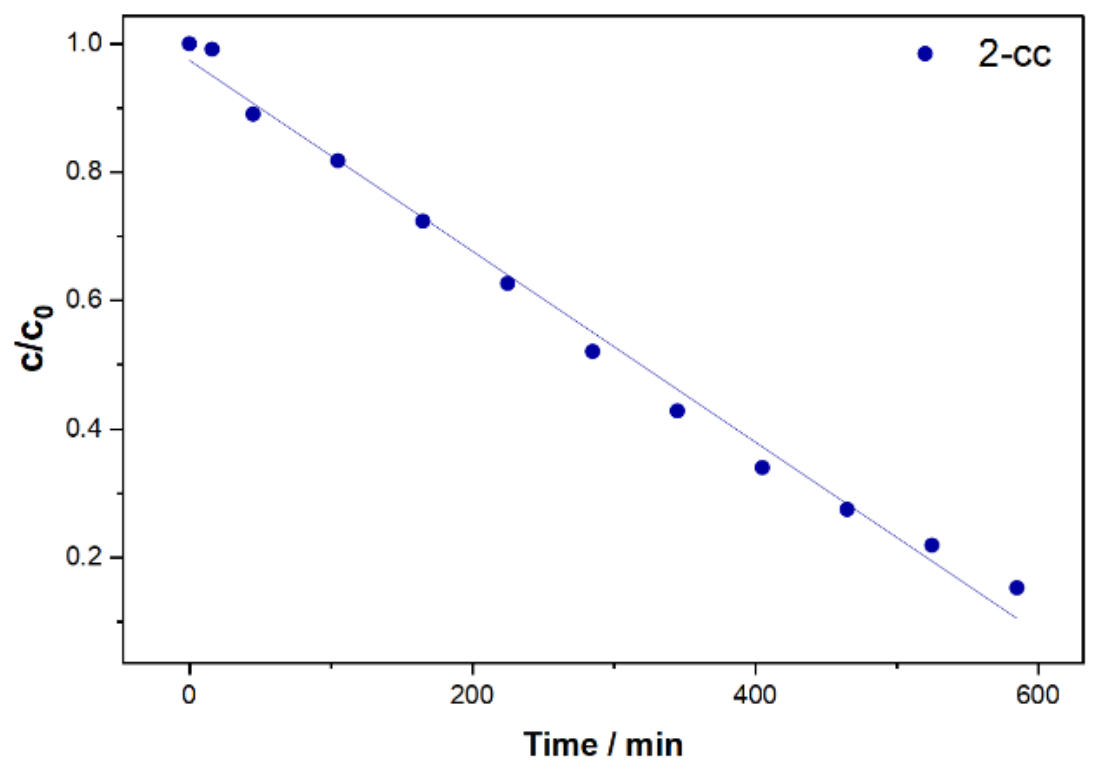

Figure S4.24. 0th order decay of **2-cc** (c/c_0) room temperature.

4.9 Enrichment of oc Form by Selective Thermal Ring-Opening

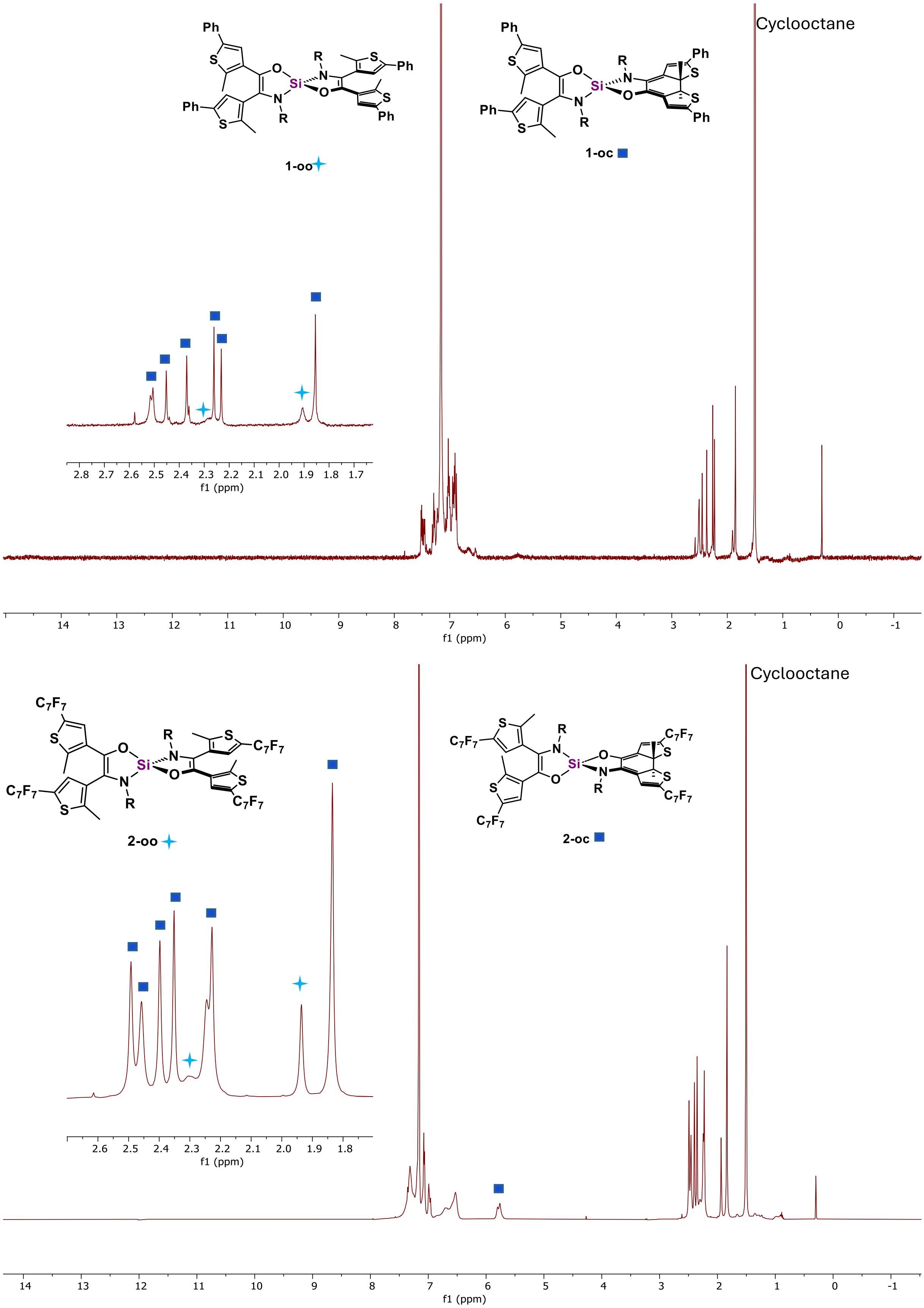

Figure S4.25. ^{1}H NMR spectra for **1** after 90 minutes at 60 °C (600 MHz, C_6D_6) and **2** after 16 hours at r.t (400 MHz, C_6D_6). Only the respective **oc** and **oo** forms remain after completed ring-opening of the **cc** form.

5 X-Ray Crystallography

A DCM solution of **2-oo-Et₃PO** was layered with n-pentane and stored at -40°C. A orange, block-shaped crystal was mounted on a MiTeGen micromount with perfluoroether oil. Crystals were immersed in perfluorinated polyether oil and fixed on a cryo-loop. Data were collected from a shock-cooled single crystal at 100(2) K on a Bruker D8 VENTURE dual wavelength Mo/Cu four-circle diffractometer with a microfocus sealed X-ray tube using a mirror optics as monochromator and a Bruker PHOTON III detector. The diffractometer was equipped with an Oxford Cryostream 700 low temperature device and used MoK_{α} radiation (λ = 0.71073 Å). All data were integrated with SAINT and a multi-scan absorption correction using SADABS was applied.[12] The structure was solved by direct methods using SHELXT and refined by full-matrix least-squares methods against F^2 by SHELXL-2019/2.[13] All non-hydrogen atoms were refined with anisotropic displacement parameters. All hydrogen atoms were refined isotropic on calculated positions using a riding model with their U_{iso} values constrained to 1.5 times the U_{eq} of their pivot atoms for terminal sp^3 carbon atoms and 1.2 times for all other carbon atoms. Crystallographic data for the structures reported here have been deposited with the Cambridge Crystallographic Data Centre.[14] CCDC 2531569 contain the supplementary crystallographic data for this paper. These data can be obtained free of charge from The Cambridge Crystallographic Data Centre via www.ccdc.cam.ac.uk/structures. This report and the CIF file were generated using FinalCif.[15]

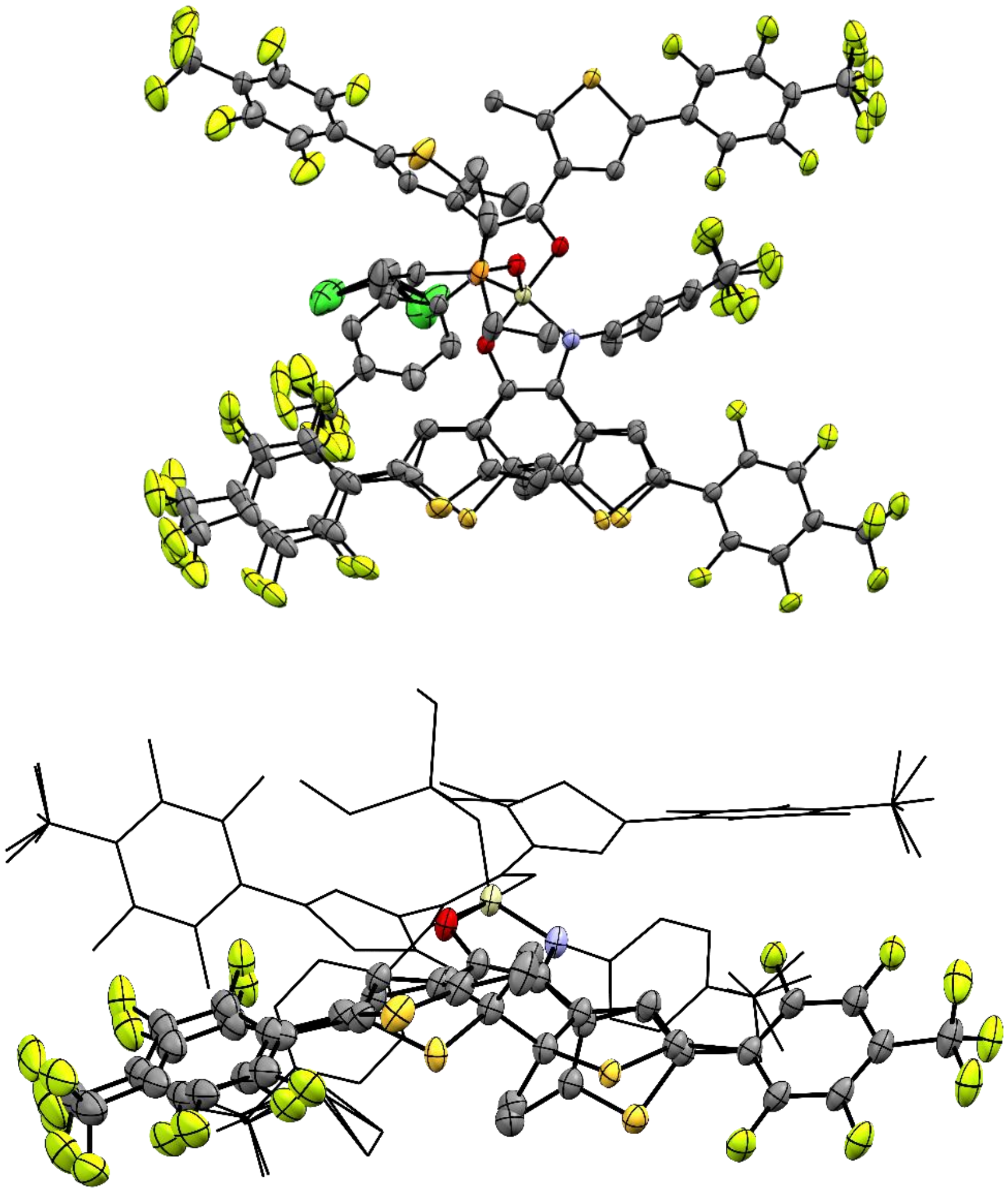

Figure S5.1. Solid-state structure of **2-oo-Et₃PO**, crystallized from DCM/*n*-pentane at –40 °C. The Structure contains 5% closed form on one side, likely due to in-crystal photocyclization by light from the diffractometer and microscope.

**CCDC number**	2531569
**Empirical formula**	$C_{72.81}H_{40.63}Cl_{1.63}F_{34}N_2O_3PS_4Si$
**Formula weight**	1882.39
**Temperature [K]**	100(2)
**Crystal system**	monoclinic
**Space group (number)**	$P2_1/c$ (14)
***a* [Å]**	15.812(5)
***b* [Å]**	37.744(10)
***c* [Å]**	12.655(3)
**α [°]**	90
**β [°]**	97.381(8)
**γ [°]**	90
**Volume [Å^3]**	7490(3)
Z	4
**ρ_{calc} [gcm^{-3}]**	1.669
**μ [mm^{-1}]**	0.358
***F*(000)**	3769
**Crystal size [mm^3]**	0.07×0.13×0.18
**Crystal colour**	orange
**Crystal shape**	block
**Radiation**	MoK_α (λ=0.71073 Å)
**2θ range [°]**	3.90 to 54.58 (0.78 Å)
**Index ranges**	−20 ≤ h ≤ 20<br>−48 ≤ k ≤ 48<br>−16 ≤ l ≤ 16
**Reflections collected**	377565
**Independent reflections**	16781<br>R_{int} = 0.0732<br>R_{sigma} = 0.0259
**Completeness to θ = 25.242°**	100.0 %
**Data / Restraints / Parameters**	16781 / 2315 / 1465
**Absorption correction T_{min}/T_{max} (method)**	0.7037 / 0.7455 (multi-scan)
**Goodness-of-fit on F^2**	1.049
**Final *R* indexes [$I \geq 2\sigma(I)$]**	R_1 = 0.0568<br>wR_2 = 0.1400
**Final *R* indexes [all data]**	R_1 = 0.0776<br>wR_2 = 0.1535
**Largest peak/hole [eÅ^{-3}]**	1.08/−0.85

6 Coordinates

1-oo

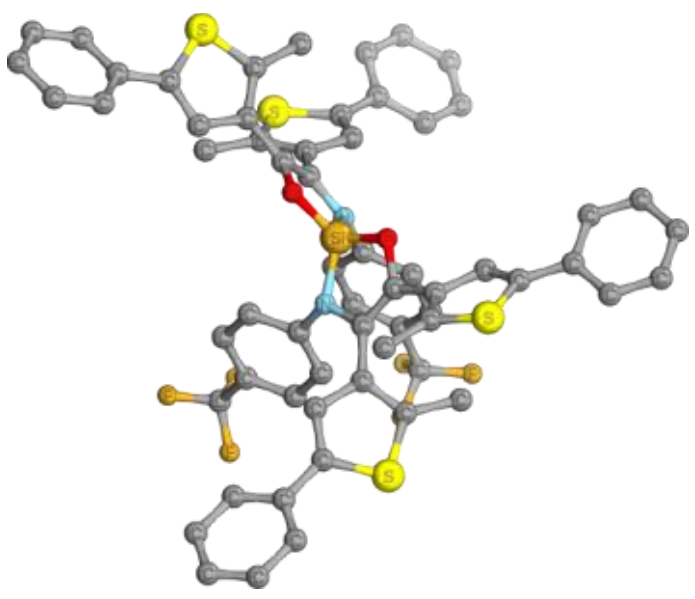

121
xyz, charge: 0, multiplicity: 1

C	0.242718	6.305708	0.804723
C	-0.772129	5.401439	0.753062
C	1.390642	5.921496	0.052127
C	1.218904	4.728304	-0.599984
S	-0.330044	4.070537	-0.252805
H	0.196511	7.210864	1.393860
C	-2.075066	5.459900	1.410429
C	-2.723821	4.302821	1.842088
C	-3.954821	4.375829	2.472405
C	-4.556644	5.605728	2.691853
C	-3.919821	6.762977	2.267309
C	-2.694805	6.691338	1.626718
H	-2.254258	3.336968	1.700368
H	-4.441715	3.467148	2.800860
H	-5.517020	5.662046	3.186534
H	-4.385940	7.726894	2.423535
H	-2.222859	7.596787	1.267500
C	2.593569	6.735333	0.025240
C	2.141254	4.009133	-1.520943
H	2.726990	3.247508	-1.002427
H	2.844411	4.706281	-1.973374
H	1.594184	3.516870	-2.325128
H	3.152874	3.965106	3.398294
S	4.821294	2.716068	1.395387
C	9.007882	2.080966	-1.512362
C	7.662443	0.146036	-1.073521
C	8.832500	0.705626	-1.564645
C	8.023420	2.890218	-0.971658
C	5.461613	4.465210	-0.329790
C	4.401346	5.033889	0.432401
C	6.837866	2.337951	-0.485533
C	3.956585	4.200947	1.424666
C	6.671421	0.954410	-0.542021
C	3.883659	6.369727	0.168996
C	2.900934	4.435584	2.447839
C	5.801938	3.208693	0.063611
H	5.929012	4.969318	-1.164311
H	1.931568	4.046382	2.129750
H	9.922636	2.525611	-1.881414
H	8.182906	3.958616	-0.899894
H	7.515926	-0.925302	-1.111118
H	9.605178	0.073492	-1.981315
H	5.751912	0.507310	-0.184200
H	2.775659	5.503647	2.622622
N	4.770451	7.449188	-0.040089
O	2.392388	8.083133	-0.188444
C	6.084585	7.521899	0.439361
C	6.477274	6.946844	1.646281
C	7.756102	7.147442	2.126963
C	8.664670	7.927009	1.420895
C	8.280097	8.494188	0.214805
C	7.005197	8.282164	-0.278053
H	6.722497	8.707156	-1.233575
H	5.776339	6.368603	2.230097
H	8.028639	6.706123	3.075574
H	8.971088	9.095465	-0.360362
C	10.015147	8.211236	1.994423
F	10.921064	8.471690	1.045777
F	10.479608	7.191994	2.721238
F	9.987643	9.282814	2.803656
H	6.697920	13.608533	-1.858822
C	4.529329	8.785216	4.122032
C	5.796195	9.193611	4.511818
C	6.056172	10.353502	2.418068
C	6.550415	9.985308	3.653547
C	4.024033	9.165631	2.891740
C	4.787059	9.938134	2.019445
C	4.569309	11.317560	-0.053338
C	6.378609	8.729584	5.807540
N	4.279660	10.183135	0.737012
F	5.435521	8.446365	6.712210
F	7.191523	9.639844	6.349658
F	7.104227	7.611099	5.645340
H	6.674589	10.937563	1.752535
H	3.023189	8.864111	2.607247
H	7.545476	10.305230	3.930214
H	3.916766	8.178828	4.775295
C	7.003724	13.100672	-0.941843
H	8.004062	13.449800	-0.685669
C	4.982696	12.565276	0.574492
C	6.041488	13.334337	0.169778
H	2.311048	14.612136	-1.963080
C	4.314099	13.103226	1.711123
S	6.189026	14.726236	1.166789
S	4.250939	14.042542	-4.014757
C	6.368987	11.472159	-7.938954
C	4.421078	12.013588	-2.487103
C	3.888845	13.276141	-2.520186
C	5.101914	12.668261	-4.619617
C	5.779678	11.488604	-6.686540
C	6.808601	13.821225	-7.765448
C	6.229813	13.837438	-6.507377
C	5.707222	12.670908	-5.948748
C	4.349729	11.093847	-1.364995
C	5.093738	11.669847	-3.695779
C	3.075656	13.989929	-1.497769
C	6.884866	12.638500	-8.485366
O	3.994641	9.799301	-1.681757
C	4.843497	14.276713	2.149531
H	3.446565	12.641804	2.162205
H	6.200277	14.763806	-5.946632
H	3.687764	14.634192	-0.863508
H	7.339280	12.626189	-9.466966
H	7.209203	14.736327	-8.181082
H	6.413211	10.546123	-8.496904
H	5.348274	10.580990	-6.284383
H	5.585897	10.719810	-3.849860
H	7.065491	12.037083	-1.169859
C	4.416589	15.095471	3.280920
C	4.500720	16.487145	3.245374
C	3.907194	14.484752	4.427405
H	4.878565	16.981878	2.358848
H	3.870953	13.404278	4.485139
C	4.083427	17.246845	4.325564
C	3.480640	15.246827	5.501480
H	2.574492	13.275939	-0.846417
C	3.567825	16.630826	5.456062
H	4.154358	18.325522	4.279974
H	3.091653	14.756440	6.384028
H	3.240342	17.225197	6.298501
Si	3.839167	8.880827	-0.311638

1-oo-F

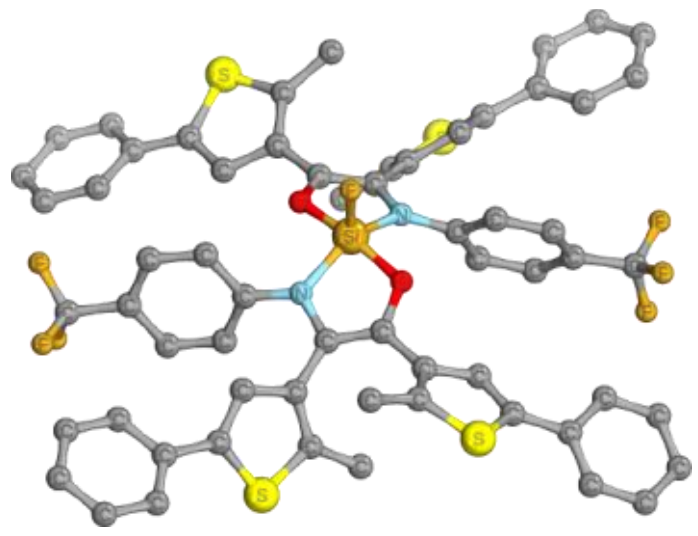

122
xyz, charge: -1, multiplicity: 1

C	-4.585590	-2.601874	1.101074
C	-5.261458	-3.535297	1.824663
C	-4.190686	-3.047876	-0.194676
C	-4.620654	-4.320001	-0.462129
S	-5.454082	-4.980252	0.894112
H	-4.334182	-1.619178	1.477173
C	-5.776901	-3.428458	3.185444
C	-5.966919	-4.555910	3.986711
C	-6.446736	-4.435821	5.280349
C	-6.736937	-3.186025	5.806533
C	-6.550217	-2.057242	5.020997
C	-6.083469	-2.176715	3.723615
H	-5.719862	-5.537559	3.600521
H	-6.584604	-5.324613	5.882792
H	-7.106665	-3.091459	6.819192
H	-6.776874	-1.075927	5.415041
H	-5.969055	-1.291303	3.112403
C	-3.418896	-2.182451	-1.076573
C	-4.485456	-5.119108	-1.710354
H	-3.632902	-5.800869	-1.675503
H	-4.332257	-4.454429	-2.559143
H	-5.380372	-5.713358	-1.900735
H	-0.478725	-4.549569	1.183631
S	-0.467229	-5.962742	-1.338688
C	1.018764	-6.585754	-6.197141
C	0.389212	-8.572386	-5.012352
C	0.969623	-7.970508	-6.119474
C	0.493293	-5.809215	-5.178364
C	-1.301362	-4.377066	-3.137430
C	-1.647985	-3.763283	-1.896339
C	-0.104642	-6.405223	-4.066444
C	-1.235504	-4.508201	-0.822490
C	-0.145427	-7.797805	-3.995792
C	-2.325082	-2.474256	-1.811516
C	-1.355056	-4.208662	0.630455
C	-0.654303	-5.563838	-3.008276
H	-1.544586	-3.944790	-4.098069
H	-2.234928	-4.678172	1.075741
H	1.485565	-6.103387	-7.045931
H	0.571633	-4.730577	-5.224828
H	0.345735	-9.651736	-4.941916
H	1.387017	-8.576539	-6.913049
H	-0.614619	-8.275692	-3.144381
H	-1.458405	-3.134453	0.779378
N	-1.908805	-1.359834	-2.561296
O	-3.819038	-0.911988	-1.147164
C	-0.726738	-1.411684	-3.292770
C	0.471100	-1.861864	-2.730151
C	1.610279	-2.013311	-3.498113
C	1.594579	-1.693210	-4.850726
C	0.419619	-1.201532	-5.413192
C	-0.719178	-1.061252	-4.645755
H	-1.630682	-0.698781	-5.099707
H	0.503687	-2.110454	-1.678279
H	2.512926	-2.381863	-3.029043
H	0.378863	-0.935120	-6.461142
C	2.787223	-1.959265	-5.700665
F	2.842605	-1.156575	-6.775780

F 2.800855 -3.218292 -6.177780
F 3.940426 -1.798230 -5.037651
H 0.901326 4.437407 -1.234537
C -5.817885 1.371638 1.360410
C -5.165136 1.678660 2.551473
C -3.079998 1.746838 1.353718
C -3.786589 1.856109 2.536648
C -5.110624 1.273633 0.179029
C -3.729272 1.483674 0.144182
C -2.204323 2.552865 -1.408187
C -5.939588 1.904285 3.802402
N -3.049815 1.481329 -1.069735
F -7.087827 1.208458 3.829354
F -6.285880 3.195894 3.959934
F -5.254705 1.563812 4.902553
H -2.007250 1.882533 1.362595
H -5.632423 1.056003 -0.742218
H -3.249272 2.079269 3.448822
H -6.888905 1.218574 1.341602
C 0.441587 3.920972 -0.389088
H 1.059267 4.105502 0.490997
C -2.127436 3.765520 -0.601494
C -0.966210 4.350915 -0.168842
H -2.073995 6.084720 -4.116133
C -3.282877 4.461264 -0.134575
S -1.296715 5.760435 0.767126
S 0.626819 5.089906 -4.397257
C 4.385616 1.902522 -5.974541
C -0.608426 3.154149 -3.282713
C -0.758546 4.484410 -3.570305
C 1.409203 3.547758 -4.394141
C 3.126230 2.100191 -5.435607
C 4.891095 4.220932 -5.651449
C 3.635970 4.417681 -5.099942
C 2.731248 3.360844 -4.982463
C -1.546136 2.305070 -2.560104
C 0.618558 2.625385 -3.781068
C -1.910984 5.385043 -3.295034
C 5.274890 2.961932 -6.087907
O -1.764697 1.097046 -3.081814
C -3.005097 5.553990 0.622256
H -4.292160 4.156145 -0.373521
H 3.362379 5.402069 -4.739668
H -1.771923 5.967717 -2.381766
H 6.257385 2.807000 -6.514314
H 5.575591 5.055787 -5.731002
H 4.668108 0.915730 -6.315167
H 2.432464 1.271926 -5.378955
H 0.900046 1.589554 -3.646677
H 0.472649 2.855361 -0.613142
C -3.946980 6.435527 1.304449
C -3.725358 7.808640 1.411483
C -5.098409 5.897265 1.881934
H -2.841685 8.243535 0.960525
H -5.260372 4.827572 1.845577
C -4.633462 8.621443 2.070215
C -6.009175 6.713219 2.531255
H -2.817358 4.795391 -3.166275
C -5.781593 8.078753 2.628770
H -4.446223 9.685262 2.141190
H -6.891013 6.274056 2.979053
H -6.490574 8.714699 3.142786
Si -2.941796 0.107765 -2.257861
F -4.120548 0.226049 -3.386646

1-oo-Et$_3$PO

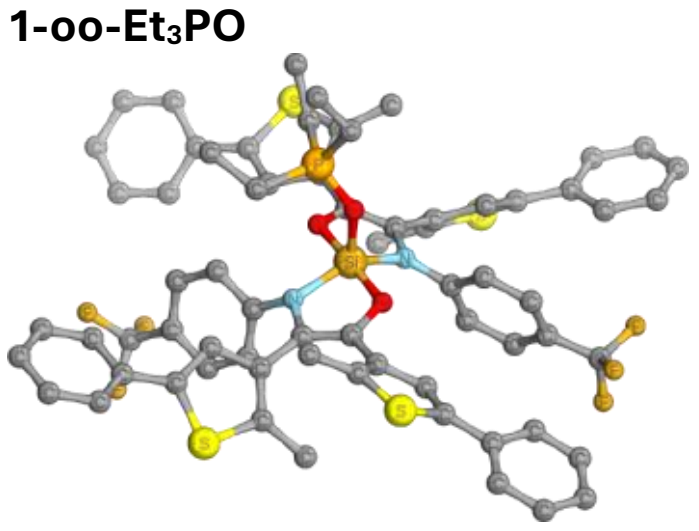

144
xyz, charge: 0, multiplicity: 1

C -4.973915 -2.537666 0.434979
C -5.897711 -3.393282 0.949292
C -4.351666 -3.003324 -0.759577
C -4.840863 -4.218020 -1.163551
S -6.018992 -4.799108 -0.047619
H -4.728149 -1.586119 0.884653
C -6.700487 -3.218598 2.156406
C -7.935522 -3.847147 2.315381
C -8.690917 -3.643907 3.458778
C -8.233660 -2.800310 4.459997
C -7.005911 -2.169964 4.313602
C -6.243122 -2.384762 3.179210
H -8.319836 -4.490343 1.532779
H -9.645688 -4.142340 3.563365
H -8.826816 -2.639304 5.350268
H -6.630222 -1.516530 5.089672
H -5.271659 -1.916010 3.097212
C -3.354624 -2.185961 -1.432535
C -4.488929 -5.023238 -2.365076
H -3.707979 -5.756700 -2.153896
H -4.103518 -4.375116 -3.152057
H -5.352821 -5.561588 -2.756472
H -1.052316 -4.805691 1.206253
S -0.621679 -6.117474 -1.328299
C 1.709620 -6.698943 -5.843085
C 0.730457 -8.671346 -4.893072
C 1.551856 -8.077512 -5.840242
C 1.051332 -5.919443 -4.907045
C -1.021547 -4.434745 -3.185632
C -1.557874 -3.841721 -2.004451
C 0.212760 -6.506975 -3.958816
C -1.399711 -4.641857 -0.903009
C 0.064058 -7.893297 -3.960672
C -2.171992 -2.518572 -1.985350
C -1.781962 -4.386107 0.513249
C -0.475180 -5.661913 -2.986244
H -1.054029 -3.963086 -4.157675
H -2.756276 -4.814996 0.757130
H 2.363202 -6.226966 -6.564762
H 1.210895 -4.848876 -4.887205
H 0.603190 -9.745884 -4.882749
H 2.072584 -8.686751 -6.567051
H -0.589720 -8.363943 -3.236421
H -1.848802 -3.314874 0.698429
N -1.550572 -1.401886 -2.574567
O -3.636829 -0.865358 -1.489835
C -0.256876 -1.513524 -3.106648
C 0.802677 -1.977797 -2.332297
C 2.056105 -2.169957 -2.886589
C 2.280235 -1.878048 -4.224976
C 1.234321 -1.386469 -4.999057
C -0.018476 -1.208929 -4.445392
H -0.828640 -0.842971 -5.059941
H 0.640386 -2.206003 -1.287580
H 2.854396 -2.546144 -2.261768
H 1.385296 -1.147907 -6.043829
C 3.602127 -2.157895 -4.859885
F 3.912591 -1.249271 -5.797707
F 3.610596 -3.350491 -5.477743
F 4.603358 -2.172176 -3.977616
H 0.781190 4.953459 -1.652601
C -4.769827 1.061272 1.807453
C -3.826917 1.327410 2.792342
C -2.175636 1.670769 1.078232
C -2.526230 1.634644 2.415052
C -4.415714 1.107654 0.471833
C -3.112098 1.407896 0.078598
C -1.994778 2.603179 -1.698885
C -4.201116 1.224797 4.234238
N -2.755347 1.486300 -1.278869
F -4.076756 -0.036067 4.689178
F -5.473141 1.578890 4.452522
F -3.434286 1.988428 5.015910
H -1.155696 1.904137 0.802849
H -5.169747 0.900561 -0.273961
H -1.770892 1.845140 3.159908
H -5.791990 0.821549 2.071485

C 0.380195 4.461977 -0.763619
H 0.930729 4.835855 0.100077
C -2.123187 3.879472 -1.007855
C -1.084423 4.681875 -0.615702
H -2.148177 5.830846 -4.766052
C -3.389436 4.419932 -0.639443
S -1.669154 6.102599 0.161750
S 0.643860 5.091169 -4.916871
C 4.778463 2.168701 -6.006222
C -0.398230 3.183723 -3.587093
C -0.685622 4.445043 -4.034231
C 1.592446 3.661257 -4.709223
C 3.491967 2.291702 -5.511648
C 5.000689 4.553610 -6.028651
C 3.718046 4.678077 -5.521300
C 2.941984 3.549397 -5.255341
C -1.259368 2.320476 -2.791704
C 0.893222 2.739087 -3.994041
C -1.930929 5.244842 -3.872246
C 5.539021 3.298957 -6.270144
O -1.313403 1.039011 -3.187143
C -3.316661 5.618497 -0.003006
H -4.329354 3.935859 -0.866497
H 3.324715 5.665958 -5.313623
H -1.870615 5.935396 -3.028372
H 6.542940 3.201686 -6.661395
H 5.584857 5.442725 -6.226587
H 5.184584 1.183736 -6.192077
H 2.903311 1.400977 -5.335244
H 1.286364 1.768259 -3.725476
H 0.591445 3.398272 -0.868113
C -4.414558 6.428613 0.516266
C -4.378489 7.822245 0.473975
C -5.535331 5.805496 1.067699
H -3.522468 8.323826 0.039305
H -5.556326 4.725760 1.148987
C -5.435829 8.571024 0.963815
C -6.595559 6.556032 1.546846
H -2.776668 4.584985 -3.683567
C -6.550839 7.942046 1.497547
H -5.390489 9.651318 0.922245
H -7.453120 6.056392 1.978240
H -7.375980 8.528047 1.879838
Si -2.507641 0.089100 -2.392088
O -3.615233 0.254386 -3.789544
P -5.158570 0.338823 -3.931102
C -5.758097 1.826008 -3.079181
H -5.104072 1.937698 -2.211482
H -5.528888 2.676230 -3.727305
C -7.221484 1.829435 -2.654886
H -7.445385 1.012502 -1.969394
H -7.907016 1.760032 -3.498108
H -7.445233 2.757999 -2.131791
C -5.446527 0.517680 -5.715522
H -4.610041 1.122203 -6.074661
H -5.322287 -0.469695 -6.167255
C -6.768874 1.152382 -6.133066
H -6.873504 2.164180 -5.743024
H -7.634908 0.576649 -5.813097
H -6.811191 1.223715 -7.218739
C -5.993791 -1.155374 -3.324664
H -6.056122 -1.088800 -2.238208
H -5.296092 -1.973631 -3.522510
C -7.354866 -1.468847 -3.936019
H -7.298953 -1.633660 -5.011466
H -8.093742 -0.691430 -3.748282
H -7.734067 -2.386737 -3.489437

1-oo-pyridine

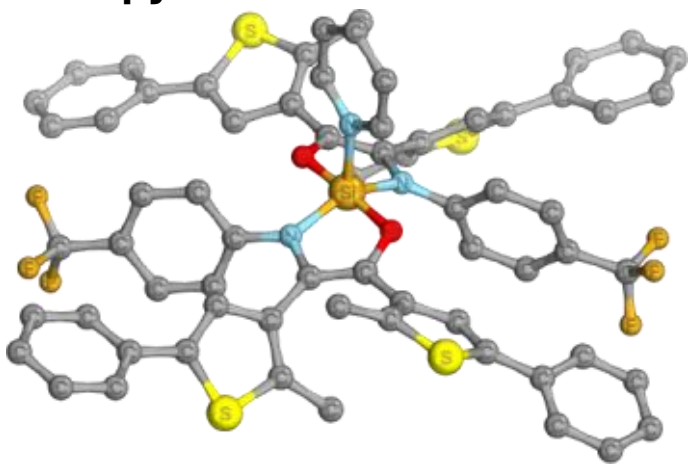

132
xyz, charge: 0, multiplicity: 1
C -4.711924 -2.656837 1.066532
C -5.507474 -3.574044 1.682430
C -4.256792 -3.064264 -0.223623
C -4.750211 -4.289536 -0.592441
S -5.731150 -4.949262 0.658533
H -4.416156 -1.721934 1.523028
C -6.118359 -3.503409 3.007262
C -6.438800 -4.657346 3.723670
C -7.008799 -4.574394 4.983175
C -7.259901 -3.337478 5.557299
C -6.942170 -2.182806 4.856372
C -6.384216 -2.263841 3.592278
H -6.224241 -5.632245 3.302625
H -7.248213 -5.482067 5.521416
H -7.700433 -3.272937 6.543263
H -7.135939 -1.212012 5.291460
H -6.166226 -1.355258 3.046292
C -3.375964 -2.216920 -1.010693
C -4.558776 -5.043569 -1.861916
H -3.732691 -5.754784 -1.796975
H -4.324733 -4.358364 -2.675749
H -5.455574 -5.601122 -2.134104
H -0.656908 -4.793175 1.237526
S -0.553913 -6.076040 -1.343362
C 1.081934 -6.614881 -6.158684
C 0.282134 -8.602858 -5.081240
C 0.944946 -7.995576 -6.137942
C 0.560325 -5.846478 -5.131516
C -1.270041 -4.401836 -3.114365
C -1.641201 -3.824214 -1.862814
C -0.119109 -6.447549 -4.071106
C -1.300095 -4.622247 -0.800758
C -0.247747 -7.836033 -4.056405
C -2.284646 -2.520090 -1.739378
C -1.484204 -4.383580 0.657516
C -0.668219 -5.615101 -3.003229
H -1.463467 -3.934449 -4.069577
H -2.406831 -4.833676 1.030032
H 1.613856 -6.133024 -6.968438
H 0.705856 -4.773767 -5.130381
H 0.171675 -9.679005 -5.056754
H 1.359141 -8.595974 -6.937039
H -0.780535 -8.317134 -3.245149
H -1.545995 -3.315514 0.861475
N -1.781091 -1.379204 -2.398138
O -3.679777 -0.896014 -1.005761
C -0.568544 -1.459944 -3.103335
C 0.606031 -1.850989 -2.466284
C 1.778142 -2.015900 -3.181934
C 1.802630 -1.773008 -4.549678
C 0.641076 -1.356521 -5.189221
C -0.530063 -1.204106 -4.470746
H -1.435558 -0.908701 -4.982939
H 0.595590 -2.045076 -1.402223
H 2.671498 -2.334859 -2.662292
H 0.635892 -1.159347 -6.252903
C 3.043010 -2.038225 -5.339007
F 3.103705 -1.287649 -6.449099
F 3.107021 -3.316272 -5.741865
F 4.153521 -1.796997 -4.638489
H 0.867983 4.625329 -1.412514
C -5.565786 1.494073 1.601569
C -4.840254 1.724007 2.764918
C -2.810975 1.728083 1.476007
C -3.457655 1.838045 2.694179
C -4.915300 1.396748 0.386020
C -3.531529 1.522070 0.302970
C -2.134090 2.604862 -1.374069
C -5.552433 1.928917 4.062287
N -2.890000 1.493162 -0.946823
F -6.692047 1.223699 4.126449
F -5.893527 3.214408 4.240352
F -4.810146 1.575515 5.113514
H -1.734141 1.819555 1.430909
H -5.491304 1.247500 -0.517127
H -2.871872 2.011530 3.586543
H -6.643275 1.400758 1.631548
C 0.485113 4.119183 -0.523787
H 1.138725 4.373674 0.310884
C -2.085124 3.841727 -0.601000
C -0.929902 4.487451 -0.241602
H -2.278043 6.033862 -4.203052
C -3.250381 4.504508 -0.111162
S -1.281072 5.907140 0.667717
S 0.414063 5.103181 -4.659951
C 4.215518 2.016572 -6.324845
C -0.645179 3.183393 -3.356990
C -0.878332 4.489709 -3.702521
C 1.271658 3.602110 -4.625137
C 2.984412 2.183222 -5.714538
C 4.631117 4.368471 -6.134036
C 3.405059 4.535056 -5.511800
C 2.561534 3.445129 -5.292463
C -1.495940 2.342370 -2.530458
C 0.573221 2.681160 -3.905927
C -2.040155 5.362240 -3.377458
C 5.044164 3.108607 -6.539932
O -1.655702 1.068391 -2.964367
C -2.984181 5.632788 0.597522
H -4.257574 4.159635 -0.298086
H 3.111128 5.522309 -5.176166
H -1.858343 5.975587 -2.492454
H 6.004251 2.977864 -7.021106
H 5.270184 5.227132 -6.292739
H 4.524516 1.029576 -6.640335
H 2.336633 1.327303 -5.576806
H 0.923286 1.673064 -3.729172
H 0.568590 3.048737 -0.706150
C -3.933566 6.509637 1.278825
C -3.782288 7.895820 1.275334
C -5.017768 5.952902 1.958398
H -2.950978 8.343493 0.744352
H -5.120051 4.876187 2.006073
C -4.698202 8.705089 1.927420
C -5.936155 6.764816 2.602629
H -2.920402 4.755744 -3.168566
C -5.780418 8.143609 2.589508
H -4.568857 9.779305 1.913137
H -6.766100 6.315840 3.132193
H -6.494402 8.777093 3.098900
Si -2.712740 0.090773 -2.027118
N -4.157763 0.219664 -3.388120
C -3.975984 0.930771 -4.508453
C -5.327391 -0.405343 -3.199658
C -4.955912 1.039458 -5.473905
C -6.349586 -0.340428 -4.124403
C -6.166059 0.393739 -5.283961
H -3.016747 1.410863 -4.624409
H -5.436376 -0.958955 -2.280077
H -4.760982 1.626964 -6.359174
H -7.273054 -0.864869 -3.926818
H -6.950825 0.461461 -6.025218

1-oc

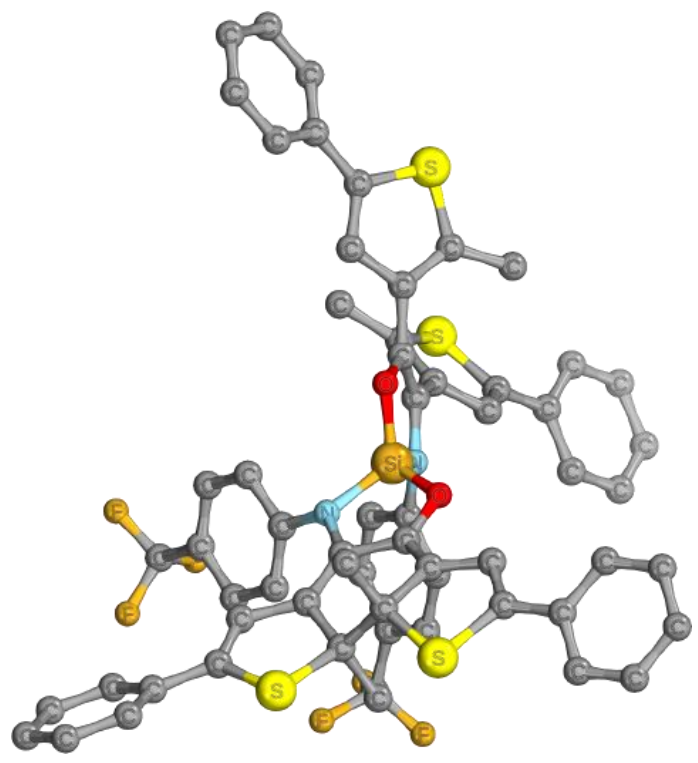

121
xyz, charge: 0, multiplicity: 1
C 0.288605 6.164549 0.473956
C -0.708819 5.250544 0.332134
C 1.510531 5.779631 -0.150830
C 1.416333 4.575215 -0.797997
S -0.155086 3.908807 -0.601581
H 0.175340 7.077918 1.040945
C -2.073103 5.307342 0.850423
C -2.756126 4.150971 1.227582
C -4.046141 4.222902 1.726291
C -4.674607 5.451050 1.866361
C -4.004189 6.607588 1.494962
C -2.719075 6.536919 0.985440
H -2.268956 3.186733 1.147783
H -4.558946 3.314820 2.014446
H -5.681280 5.506579 2.258413
H -4.489512 7.569996 1.589779
H -2.218154 7.441497 0.665434
C 2.703807 6.603557 -0.066612
C 2.434167 3.850349 -1.607295
H 2.968646 3.102119 -1.018676
H 3.175288 4.546431 -1.996325
H 1.976398 3.340809 -2.455269
H 2.938895 3.903602 3.399828
S 4.799319 2.625890 1.592150
C 9.252901 1.960379 -0.880265
C 7.878601 0.026732 -0.535035
C 9.088562 0.583332 -0.921700
C 8.217641 2.774402 -0.453658
C 5.601541 4.348176 -0.092035
C 4.470090 4.924956 0.552847
C 6.992708 2.224868 -0.073640
C 3.931791 4.106987 1.510656
C 6.837657 0.839603 -0.118270
C 3.974510 6.252786 0.216692
C 2.779158 4.353781 2.420048
C 5.905182 3.100250 0.355030
H 6.147052 4.839705 -0.885670
H 1.848320 3.951072 2.015582
H 10.197260 2.403004 -1.168188
H 8.365595 3.844867 -0.388559
H 7.740350 -1.045897 -0.565229
H 9.900312 -0.052365 -1.249051
H 5.889518 0.394167 0.157524
H 2.631460 5.424020 2.560111
N 4.871405 7.338202 0.084955
O 2.516171 7.946115 -0.324615
C 6.141564 7.415900 0.671411
C 6.431819 6.846772 1.909331
C 7.669315 7.043360 2.490057
C 8.634767 7.815793 1.855927
C 8.351952 8.377381 0.619104
C 7.120783 8.166866 0.024921
H 6.921294 8.583744 -0.954832
H 5.683540 6.274683 2.437798
H 7.863754 6.603353 3.458042
H 9.090011 8.972396 0.098379
C 9.936346 8.102472 2.532663
F 10.926400 8.301231 1.656733

F 10.313101 7.112413 3.344788
F 9.860214 9.213399 3.284669
H 7.404732 13.842869 -1.469271
C 4.226230 8.733915 4.175122
C 5.477158 9.114404 4.634447
C 5.901958 10.222739 2.541487
C 6.310562 9.863517 3.809363
C 3.808035 9.103491 2.907230
C 4.643788 9.841393 2.077174
C 4.560064 11.226190 0.011066
C 5.966819 8.677807 5.978242
N 4.237209 10.084358 0.754190
F 4.966010 8.343669 6.797672
F 6.678424 9.632606 6.584309
F 6.765374 7.603477 5.882704
H 6.571552 10.778782 1.900149
H 2.819905 8.824460 2.563001
H 7.299817 10.151117 4.138669
H 3.557416 8.159099 4.800720
C 6.923301 13.120742 -0.812968
H 7.432392 13.144538 0.149951
C 4.874771 12.473662 0.431756
C 5.437181 13.438536 -0.608552
H 2.593386 13.607639 -2.620624
C 4.816569 13.057652 1.731630
S 5.333862 15.095773 0.182173
S 5.308885 14.252713 -3.311971
C 4.624457 12.425163 -8.107902
C 4.580372 11.889050 -2.347313
C 4.601116 13.333856 -1.883134
C 4.975536 12.910314 -4.404187
C 4.463918 12.264509 -6.743889
C 6.102530 14.271364 -7.719175
C 5.944297 14.110738 -6.352859
C 5.128499 13.099863 -5.842303
C 4.489318 10.931837 -1.411167
C 4.619314 11.772111 -3.768865
C 3.150310 13.798286 -1.704393
C 5.445964 13.428815 -8.602753
O 4.272098 9.612561 -1.696581
C 5.036733 14.391409 1.763161
H 4.536965 12.505286 2.616308
H 6.476068 14.770970 -5.678789
H 3.093488 14.862071 -1.484825
H 5.567027 13.557048 -9.670074
H 6.743278 15.058494 -8.093659
H 4.096816 11.770913 -8.789193
H 3.796145 11.497427 -6.374295
H 4.445256 10.836754 -4.281497
H 7.051886 12.128653 -1.247411
C 5.014959 15.245619 2.944509
C 4.755156 16.613144 2.841394
C 5.250900 14.707176 4.212527
H 4.563028 17.056748 1.872158
H 5.492997 13.657156 4.315155
C 4.716971 17.413426 3.970792
C 5.209286 15.507651 5.339208
H 2.668721 13.254767 -0.890611
C 4.941044 16.864937 5.224119
H 4.509157 18.470295 3.869748
H 5.398934 15.072323 6.311342
H 4.914401 17.491337 6.105682
Si 3.962501 8.748502 -0.315973

1-oc-F

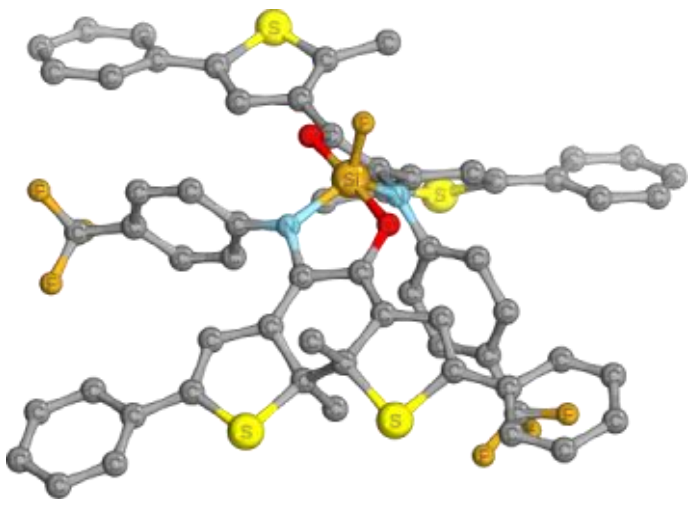

122
xyz, charge: -1, multiplicity: 1

C -14.771624 -2.332575 3.546698
C -15.461689 -3.290632 4.224646
C -14.034053 -2.829072 2.434047
C -14.205443 -4.173917 2.249813
S -15.233516 -4.828553 3.466366
H -14.739994 -1.292688 3.842199
C -16.281168 -3.144802 5.422903
C -16.588417 -4.236520 6.237450
C -17.358298 -4.080769 7.377872
C -17.829555 -2.828124 7.739692
C -17.526316 -1.733528 6.942597
C -16.769107 -1.890257 5.795481
H -16.208518 -5.219761 5.987455
H -17.581938 -4.943818 7.991793
H -18.424872 -2.704673 8.634817
H -17.884044 -0.749182 7.213051
H -16.566178 -1.030978 5.170251
C -13.197728 -1.947667 1.626181
C -13.653565 -5.058138 1.187367
H -12.753547 -5.581568 1.517005
H -13.381355 -4.465209 0.315837
H -14.381229 -5.808247 0.874569
H -10.669464 -3.461568 4.918198
S -9.790220 -5.155571 2.735630
C -7.156509 -6.230209 -1.555001
C -7.688927 -8.049749 -0.090796
C -7.004090 -7.561450 -1.193545
C -7.981911 -5.395488 -0.821507
C -10.301719 -3.945693 0.562380
C -11.058442 -3.271650 1.564806
C -8.686549 -5.879595 0.282599
C -10.858840 -3.806382 2.810241
C -8.525076 -7.219538 0.637280
C -11.913658 -2.134194 1.255545
C -11.405811 -3.358522 4.119854
C -9.567341 -4.989433 1.030916
H -10.337804 -3.666915 -0.481582
H -12.294935 -3.921946 4.411048
H -6.616849 -5.833506 -2.405031
H -8.064484 -4.349050 -1.086262
H -7.579110 -9.086276 0.200978
H -6.352831 -8.210967 -1.763458
H -9.073536 -7.619200 1.481533
H -11.702796 -2.311245 4.059019
N -11.460419 -1.116042 0.389979
O -13.763084 -0.821332 1.193004
C -10.091968 -0.825516 0.354929
C -9.327577 -0.781445 1.524163
C -7.985838 -0.459183 1.485822
C -7.367584 -0.160801 0.277383
C -8.114229 -0.212157 -0.891933
C -9.452688 -0.556837 -0.854492
H -10.021652 -0.606344 -1.771711
H -9.795900 -0.991728 2.476725
H -7.427305 -0.425264 2.412501
H -7.657480 0.014196 -1.845646
C -5.932250 0.242905 0.274693
F -5.413832 0.296620 -0.957757
F -5.170409 -0.610398 0.979880
F -5.748392 1.449670 0.828477
H -7.974739 3.922633 1.728105
C -15.104000 1.682081 4.213292
C -14.285501 1.284520 5.262501
C -12.485658 0.934419 3.709079
C -12.975335 0.893224 5.000166
C -14.608768 1.716366 2.920088
C -13.289947 1.372639 2.659281
C -11.832855 2.499647 1.125652
C -14.760887 1.340282 6.676912
N -12.761242 1.510270 1.365192
F -16.094567 1.436264 6.766877
F -14.258434 2.407520 7.322737
F -14.392934 0.265603 7.380105
H -11.459026 0.658689 3.505389
H -15.238099 2.035715 2.101004
H -12.328276 0.563021 5.802370
H -16.127860 1.979981 4.393241

C -8.917157 3.410710 1.913681
H -8.994545 3.210374 2.982058
C -11.400626 3.531524 1.903206
C -10.120629 4.252027 1.473896
H -11.313666 5.536768 -1.538191
C -11.891637 4.038335 3.142531
S -10.101489 5.807857 2.462507
S -8.579765 5.118390 -0.731745
C -6.699095 3.242356 -5.189887
C -10.394490 3.185395 -0.733352
C -10.173487 4.509092 -0.030776
C -8.707130 4.021050 -2.114396
C -7.542642 3.099555 -4.104459
C -6.321210 5.530139 -4.598159
C -7.171052 5.390561 -3.512793
C -7.805897 4.174376 -3.247228
C -11.251852 2.289902 -0.196335
C -9.669845 3.086872 -1.955241
C -11.274804 5.487226 -0.450516
C -6.081578 4.460282 -5.445965
O -11.660250 1.187554 -0.783167
C -11.316150 5.181590 3.576888
H -12.711101 3.588753 3.680323
H -7.359730 6.241144 -2.869996
H -11.095695 6.489032 -0.064289
H -5.415619 4.569152 -6.291787
H -5.845280 6.484970 -4.781684
H -6.510862 2.393005 -5.834190
H -7.989801 2.134958 -3.902782
H -9.883662 2.321215 -2.688242
H -8.893423 2.456662 1.385739
C -11.640855 5.902024 4.798753
C -11.199039 7.211194 5.008687
C -12.411164 5.304931 5.805109
H -10.597758 7.704090 4.254757
H -12.744983 4.281475 5.697224
C -11.526362 7.899899 6.165614
C -12.740079 5.995085 6.955858
H -12.245937 5.147977 -0.087803
C -12.301148 7.299815 7.145524
H -11.171879 8.914028 6.299038
H -13.336273 5.504270 7.714418
H -12.554731 7.837301 8.049887
Si -12.718656 0.151582 0.170650
F -13.773995 0.073349 -1.067027

1-oc-Et$_3$PO

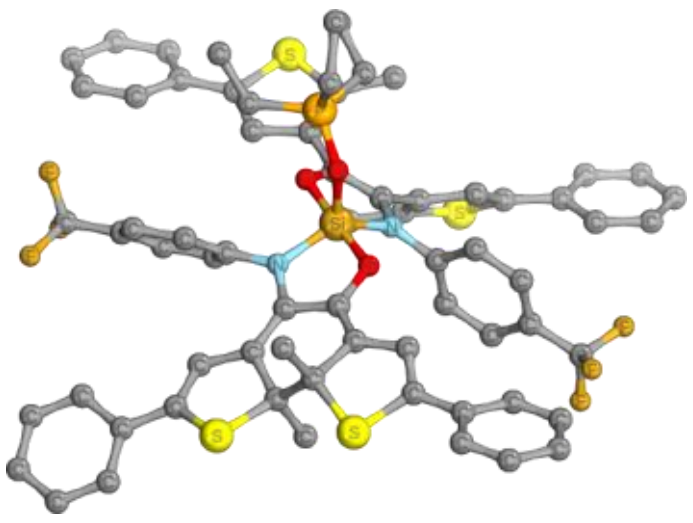

144
xyz, charge: 0, multiplicity: 1

C -14.585677 -2.458919 3.132486
C -15.446574 -3.401724 3.603722
C -13.679383 -2.937978 2.140646
C -13.886979 -4.261823 1.844680
S -15.160476 -4.906764 2.807395
H -14.560220 -1.443377 3.500538
C -16.469375 -3.253692 4.635499
C -16.952306 -4.352484 5.346706
C -17.912673 -4.193162 6.331682
C -18.402236 -2.932088 6.634622
C -17.926947 -1.830391 5.937807
C -16.977087 -1.989542 4.944274
H -16.562220 -5.342766 5.144655
H -18.271790 -5.059204 6.871582
H -19.147505 -2.807567 7.408666
H -18.300683 -0.841292 6.163770
H -16.637368 -1.121582 4.393490

C -12.730823 -2.031460 1.513025
C -13.217197 -5.131884 0.839597
H -12.362586 -5.665680 1.260270
H -12.833702 -4.535060 0.012509
H -13.904461 -5.875363 0.434201
H -10.343878 -3.911663 4.630159
S -9.376990 -5.358762 2.335443
C -6.497507 -5.953648 -1.854181
C -7.162274 -7.965434 -0.729933
C -6.378330 -7.324809 -1.678409
C -7.393587 -5.227851 -1.087911
C -9.874284 -4.011985 0.242981
C -10.634684 -3.402350 1.284444
C -8.195074 -5.865845 -0.140037
C -10.456608 -4.027199 2.491653
C -8.065539 -7.243179 0.032567
C -11.466059 -2.221868 1.081796
C -11.041629 -3.697709 3.820479
C -9.135769 -5.076035 0.650423
H -9.885010 -3.673552 -0.783257
H -11.958432 -4.259163 4.013083
H -5.877644 -5.440217 -2.577232
H -7.452480 -4.152670 -1.201225
H -7.074096 -9.034219 -0.585946
H -5.672402 -7.890446 -2.271881
H -8.686559 -7.754726 0.757966
H -11.300586 -2.640358 3.865987
N -10.987422 -1.091930 0.392893
O -13.212019 -0.790199 1.266465
C -9.665130 -1.045981 -0.085350
C -8.579312 -1.175588 0.770963
C -7.285424 -1.183002 0.275532
C -7.059473 -1.042815 -1.085210
C -8.141824 -0.896394 -1.947173
C -9.428845 -0.902128 -1.450474
H -10.267743 -0.801018 -2.125389
H -8.745873 -1.280523 1.834511
H -6.459804 -1.291988 0.964661
H -7.986840 -0.784332 -3.012640
C -5.681881 -1.091206 -1.660424
F -5.478529 -0.107470 -2.548087
F -5.468612 -2.243157 -2.319886
F -4.730497 -0.991843 -0.731864
H -8.023996 4.906538 2.059787
C -15.248455 2.626449 3.609259
C -15.119929 1.819662 4.736381
C -13.140648 0.837322 3.788755
C -14.063884 0.923639 4.819580
C -14.329330 2.533504 2.583675
C -13.261167 1.639006 2.661760
C -11.533012 2.714082 1.366189
C -16.123728 1.957989 5.834746
N -12.316627 1.594737 1.627718
F -17.365010 1.679603 5.398582
F -16.163540 3.208598 6.311744
F -15.879760 1.145969 6.862948
H -12.304366 0.155458 3.867780
H -14.409004 3.186153 1.723126
H -13.940894 0.294101 5.690279
H -16.060106 3.338616 3.530325
C -8.848340 4.220146 2.240498
H -8.950225 4.080000 3.315994
C -11.283966 3.812752 2.120280
C -10.167636 4.754652 1.669492
H -11.362868 5.552541 -1.508851
C -11.806232 4.230987 3.388124
S -10.547259 6.342401 2.522884
S -8.659807 5.730812 -0.512319
C -5.706511 3.692900 -4.205228
C -10.087983 3.491283 -0.447114
C -10.161345 4.884204 0.146002
C -8.411649 4.478755 -1.731122
C -6.680411 3.543009 -3.234266
C -6.071547 6.061933 -4.210072
C -7.049117 5.914833 -3.239547
C -7.376532 4.651678 -2.744568
C -10.839895 2.521569 0.103902
C -9.215092 3.407726 -1.572601
C -11.390101 5.601374 -0.420951
C -5.398645 4.952982 -4.700147
O -11.011143 1.307961 -0.413061
C -11.509437 5.501760 3.733420
H -12.366204 3.587905 4.048625
H -7.576552 6.786259 -2.872054
H -11.423768 6.648380 -0.125413
H -4.631616 5.069893 -5.454183
H -5.835323 7.049165 -4.585036
H -5.172445 2.822648 -4.563790
H -6.880407 2.560870 -2.825211
H -9.179926 2.542214 -2.219684
H -8.596729 3.258902 1.790817
C -11.919235 6.183160 4.956376
C -11.207574 7.277440 5.450791
C -13.046192 5.751833 5.664103
H -10.327093 7.625331 4.925429
H -13.638269 4.927892 5.286398
C -11.600436 7.909309 6.619703
C -13.432523 6.380213 6.833144
H -12.307294 5.121071 -0.076965
C -12.711399 7.463551 7.318384
H -11.030973 8.752832 6.987182
H -14.308166 6.028161 7.362779
H -13.017739 7.958504 8.230362
Si -12.106934 0.283434 0.433680
O -13.279096 0.433106 -0.891301
P -14.656103 -0.284756 -1.083128
C -15.279146 0.454096 -2.624873
H -15.622317 1.463984 -2.387122
H -14.393435 0.581151 -3.252037
C -16.347326 -0.325622 -3.384052
H -17.265079 -0.454203 -2.814539
H -15.996067 -1.312919 -3.682030
H -16.606125 0.208867 -4.296805
C -14.445552 -2.067279 -1.363687
H -14.163105 -2.189180 -2.412780
H -13.583431 -2.389293 -0.777018
C -15.657598 -2.926142 -1.017638
H -16.538250 -2.670404 -1.605900
H -15.910715 -2.855025 0.039087
H -15.427103 -3.971362 -1.217583
C -15.830809 0.059845 0.263908
H -15.664164 -0.676800 1.048508
H -15.510009 1.014992 0.687126
C -17.304433 0.123361 -0.120956
H -17.680674 -0.826011 -0.498503
H -17.512378 0.894552 -0.861616
H -17.887072 0.365490 0.766660

1-oc-pyridine

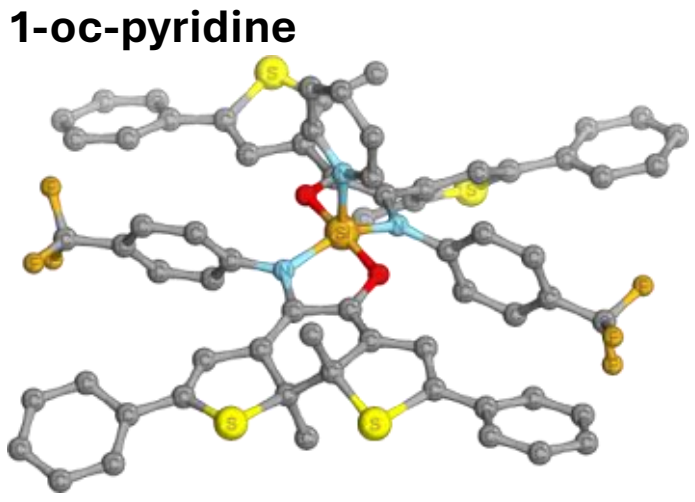

132
xyz, charge: 0, multiplicity: 1
C -14.211313 -2.517057 3.999260
C -15.026587 -3.413051 4.618328
C -13.567468 -3.031145 2.833107
C -13.937377 -4.324082 2.559181
S -15.030997 -4.908526 3.753797
H -14.042177 -1.516355 4.371881
C -15.820341 -3.223604 5.829844
C -16.095616 -4.284398 6.693237
C -16.841139 -4.085033 7.843453
C -17.317431 -2.821173 8.158018
C -17.049001 -1.758054 7.307592
C -16.314354 -1.957628 6.151255
H -15.707907 -5.272024 6.474345
H -17.042759 -4.920517 8.500827
H -17.896707 -2.665506 9.058330
H -17.424099 -0.770309 7.537832
H -16.141531 -1.128613 5.476835
C -12.655379 -2.201202 2.064741
C -13.558357 -5.199210 1.415751
H -12.696828 -5.829768 1.645194
H -13.288990 -4.594706 0.550714
H -14.380767 -5.854598 1.127527
H -9.992177 -4.473171 4.680641
S -9.539597 -5.866170 2.198649
C -7.445960 -6.483072 -2.432139
C -8.128261 -8.485859 -1.302421
C -7.443354 -7.867559 -2.338448
C -8.127701 -5.722042 -1.497546
C -10.240907 -4.365998 0.273226
C -10.762638 -3.751791 1.451097
C -8.829596 -6.336002 -0.458751
C -10.450776 -4.456969 2.585571
C -8.818027 -7.727481 -0.370591
C -11.496801 -2.491148 1.439220
C -10.786666 -4.156729 4.004679
C -9.547561 -5.510175 0.509208
H -10.392168 -3.975795 -0.723015
H -11.708209 -4.652719 4.317194
H -6.900113 -5.989589 -3.225481
H -8.092521 -4.640998 -1.552658
H -8.131026 -9.564902 -1.221956
H -6.904583 -8.461452 -3.064756
H -9.366228 -8.219829 0.423468
H -10.940924 -3.086855 4.138371
N -10.990763 -1.360038 0.765056
O -13.002470 -0.890496 1.981611
C -9.753369 -1.413766 0.095585
C -8.567452 -1.636810 0.781965
C -7.361104 -1.709460 0.103546
C -7.324471 -1.546740 -1.273212
C -8.509211 -1.326099 -1.968925
C -9.708592 -1.268231 -1.288962
H -10.630742 -1.125316 -1.837153
H -8.587900 -1.758762 1.856490
H -6.453281 -1.885472 0.663272
H -8.502506 -1.207649 -3.045057
C -6.047671 -1.636353 -2.044659
F -5.894256 -0.587137 -2.867730
F -6.021435 -2.730658 -2.821054
F -4.971483 -1.684353 -1.260178
H -8.383010 5.278901 2.173928
C -15.410130 2.361960 3.834385
C -15.096977 1.932440 5.120289
C -12.967199 1.150380 4.330340
C -13.878181 1.314038 5.362341
C -14.499653 2.193465 2.809113
C -13.262968 1.602947 3.052697
C -11.613923 2.663793 1.642990
C -16.083646 2.177323 6.217220
N -12.313362 1.523269 2.017473
F -17.263165 1.591949 5.958617
F -16.333044 3.485670 6.365842
F -15.666465 1.715829 7.396382
H -12.006249 0.691980 4.520952
H -14.727095 2.556135 1.814966
H -13.620503 0.969122 6.354038
H -16.358725 2.841468 3.629171
C -9.158569 4.554155 2.412481
H -9.337310 4.584228 3.486587
C -11.538291 3.897928 2.202022
C -10.466977 4.857010 1.672324
H -11.464260 5.010109 -1.669087
C -12.218384 4.477737 3.322195
S -11.042593 6.522027 2.208749
S -8.872516 5.598545 -0.549823
C -5.649539 3.267010 -3.856783
C -10.113056 3.279437 -0.195595
C -10.346523 4.736010 0.151125
C -8.452541 4.203515 -1.546164
C -6.655116 3.178469 -2.911783

C -6.047389 5.621702 -4.063747
C -7.058237 5.535361 -3.119935
C -7.383797 4.310946 -2.534114
C -10.808523 2.348608 0.478717
C -9.171048 3.099040 -1.252501
C -11.582386 5.229572 -0.608320
C -5.340696 4.489600 -4.438840
O -10.834551 1.049408 0.158628
C -12.054830 5.810731 3.463094
H -12.801496 3.907410 4.027625
H -7.611352 6.425428 -2.846968
H -11.727570 6.302048 -0.495986
H -4.548661 4.559357 -5.172555
H -5.812642 6.579999 -4.508035
H -5.094853 2.378409 -4.128203
H -6.857467 2.223884 -2.443425
H -9.018610 2.146113 -1.741264
H -8.788830 3.561149 2.153820
C -12.633750 6.649554 4.507208
C -12.089702 7.896776 4.819688
C -13.756039 6.219435 5.224182
H -11.214838 8.249554 4.288094
H -14.220667 5.271154 4.986302
C -12.640506 8.681079 5.820659
C -14.299615 7.000946 6.226252
H -12.480480 4.726783 -0.246408
C -13.745150 8.237771 6.530516
H -12.199152 9.642492 6.048158
H -15.166934 6.645298 6.766935
H -14.174617 8.850956 7.311571
Si -11.977381 0.086308 1.014706
N -13.316772 0.223447 -0.431454
C -13.010898 0.797557 -1.602680
C -14.562727 -0.226618 -0.232975
C -13.939320 0.936942 -2.612898
C -15.541295 -0.115899 -1.199779
C -15.229515 0.473985 -2.412765
H -11.998083 1.150348 -1.720272
H -14.764057 -0.677423 0.726694
H -13.644745 1.408378 -3.539027
H -16.531774 -0.493438 -0.992062
H -15.977522 0.572437 -3.187781

1-cc

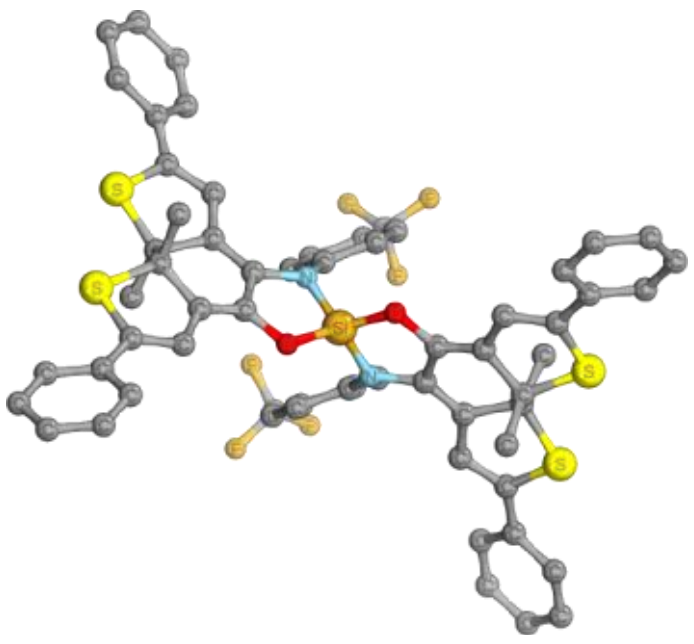

121
xyz, charge: 0, multiplicity: 1
C -5.672181 -2.686833 -1.378911
C -6.425890 -3.789131 -1.170700
C -4.266730 -2.929502 -1.338127
C -3.930394 -4.406809 -1.422871
S -5.478787 -5.249431 -0.895159
H -6.089959 -1.697069 -1.496309
C -7.882158 -3.859265 -1.126710
C -8.533257 -4.887887 -0.444009
C -9.916026 -4.936419 -0.387483
C -10.675863 -3.961570 -1.015007
C -10.040576 -2.938015 -1.704910
C -8.659936 -2.889056 -1.763971
H -7.957168 -5.651618 0.063840
H -10.400621 -5.739846 0.151044
H -11.756086 -4.001799 -0.973488
H -10.624719 -2.179697 -2.209191
H -8.180004 -2.102346 -2.331122
C -3.247806 -2.058277 -1.279758
C -3.689940 -4.756792 -2.896871
H -3.558927 -5.827142 -3.038995
H -2.800571 -4.250356 -3.273941
H -4.544869 -4.434992 -3.489762
H -2.092666 -4.651519 1.534836
S -2.082585 -6.408264 -0.729498
C 3.016258 -7.285394 -0.611127
C 1.517725 -9.006956 -1.344315
C 2.798518 -8.598460 -1.005988
C 1.964042 -6.390035 -0.557251
C -0.330680 -4.485616 -0.966712
C -1.575043 -3.789844 -0.927584
C 0.670137 -6.786189 -0.907142
C -2.731580 -4.700276 -0.522433
C 0.462975 -8.111188 -1.294008
C -1.864657 -2.485599 -1.145811
C -3.011775 -4.495135 0.971157
C -0.430595 -5.830814 -0.870965
H 0.615201 -3.996655 -1.145216
H -3.759706 -5.193211 1.341669
H 4.009717 -6.960016 -0.332452
H 2.144354 -5.378361 -0.217138
H 1.336801 -10.028338 -1.651491
H 3.621498 -9.299595 -1.042480
H -0.527942 -8.445089 -1.576343
H -3.367679 -3.482484 1.164288
N -1.004439 -1.391242 -1.311283
O -3.403706 -0.704031 -1.394533
C 0.310409 -1.316551 -0.821064
C 0.664821 -1.857032 0.413438
C 1.938562 -1.670469 0.911440
C 2.876386 -0.936021 0.193378
C 2.525392 -0.396872 -1.035000
C 1.253240 -0.594935 -1.544832
H 0.994700 -0.195277 -2.517556
H -0.063460 -2.403943 0.996051
H 2.184810 -2.083581 1.879682
H 3.240042 0.169867 -1.616085
C 4.228081 -0.671656 0.776540
F 5.154353 -0.478158 -0.166302
F 4.646800 -1.676328 1.549865
F 4.220782 0.431143 1.542778
H 1.404200 5.138342 -2.971221
C -1.251015 0.000002 2.921869
C 0.035072 0.377488 3.272534
C 0.287692 1.476031 1.146621
C 0.799150 1.120774 2.377247
C -1.773841 0.368288 1.692377
C -1.007700 1.099951 0.793126
C -1.279378 2.507331 -1.243407
C 0.637800 -0.044906 4.574554
N -1.526258 1.352410 -0.488311
F -0.282347 -0.451815 5.451954
F 1.327461 0.946313 5.147270
F 1.495664 -1.063745 4.407906
H 0.902545 2.027671 0.449048
H -2.789812 0.094668 1.436124
H 1.812661 1.407568 2.623116
H -1.867152 -0.570595 3.602747
C 0.990321 4.407273 -2.279677
H 1.597644 4.413575 -1.375217
C -0.920704 3.747883 -0.837211
C -0.464427 4.727468 -1.915696
H -3.503074 4.938530 -3.614518
C -0.847833 4.314138 0.469730
S -0.475338 6.372584 -1.094257
S -0.872588 5.586996 -4.577167
C -2.067347 3.839774 -9.303115
C -1.506641 3.210026 -3.579727
C -1.429981 4.646608 -3.096959
C -1.327333 4.264631 -5.649670
C -2.083116 3.656511 -7.932651
C -0.556355 5.681864 -9.042057
C -0.569470 5.498528 -7.669529
C -1.327003 4.478067 -7.092568
C -1.502386 2.238099 -2.654551
C -1.619912 3.116824 -4.998947
C -2.852044 5.111978 -2.758919
C -1.302560 4.852906 -9.865150
O -1.759101 0.924429 -2.937195
C -0.619608 5.646708 0.497888
H -1.043494 3.751436 1.370103
H 0.030594 6.148523 -7.044629
H -2.880490 6.172050 -2.517164
H -1.294601 4.998638 -10.937028
H 0.041367 6.476057 -9.469051
H -2.663681 3.195743 -9.935715
H -2.708003 2.882106 -7.507545
H -1.851336 2.190814 -5.505793
H 1.067543 3.422113 -2.741106
C -0.522040 6.484017 1.687432
C -0.778508 7.854741 1.627817
C -0.171894 5.925890 2.920087
H -1.057863 8.313801 0.687420
H 0.069198 4.872685 2.985848
C -0.702458 8.639174 2.766361
C -0.099542 6.710540 4.056239
H -3.248111 4.556363 -1.908009
C -0.365478 8.071318 3.985282
H -0.909727 9.698843 2.699625
H 0.177145 6.259969 5.000123
H -0.303137 8.685290 4.873747
Si -1.935837 0.046691 -1.544706

1-cc-F

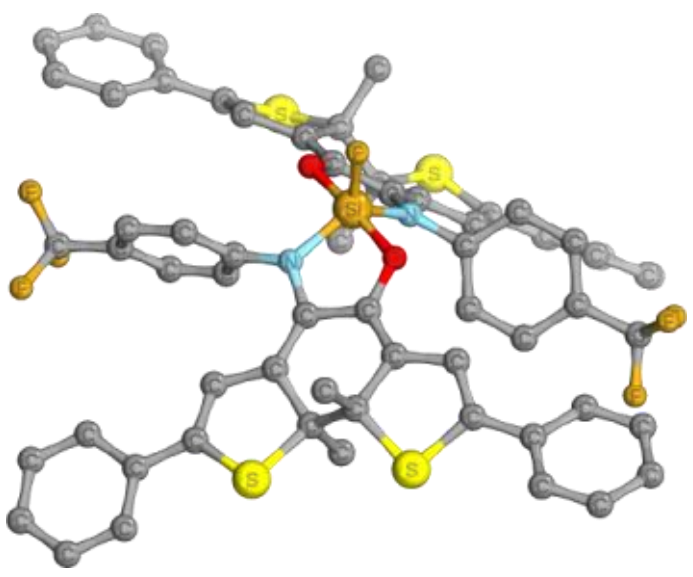

122
xyz, charge: -1, multiplicity: 1
C -5.164353 -2.776248 0.377565
C -5.317234 -3.720173 1.329757
C -4.005596 -2.940547 -0.434472
C -3.365364 -4.304916 -0.288558
S -4.037136 -4.940993 1.309794
H -5.838863 -1.940039 0.253734
C -6.356667 -3.777725 2.348627
C -6.728409 -4.984122 2.946059
C -7.715766 -5.020451 3.917686
C -8.350260 -3.854620 4.318110
C -7.983370 -2.646863 3.738338
C -6.996922 -2.608022 2.770811
H -6.249882 -5.903940 2.633689
H -7.990355 -5.968092 4.363265
H -9.116766 -3.883761 5.081498
H -8.453780 -1.724703 4.053739
H -6.701449 -1.656528 2.349866
C -3.473354 -2.054240 -1.301926
C -3.875447 -5.214798 -1.409249
H -3.546184 -6.244382 -1.280217
H -3.520795 -4.861069 -2.378230
H -4.964612 -5.198375 -1.415102
H -0.255906 -3.220287 0.853499
S -0.936749 -5.719870 -0.409332
C 3.484108 -5.965637 -3.152427
C 3.130464 -7.416969 -1.283079
C 3.910110 -7.023868 -2.358817
C 2.297775 -5.314273 -2.874951
C -0.130548 -3.798728 -1.974196
C -1.402065 -3.333784 -1.514548
C 1.503232 -5.693467 -1.785182
C -1.847679 -4.125034 -0.282830
C 1.943346 -6.760871 -0.998301
C -2.163444 -2.275995 -1.907283

C	-1.326816	-3.391075	0.958628
C	0.259330	-4.996911	-1.487368
H	0.492090	-3.242323	-2.655933
H	-1.498421	-3.962310	1.868747
H	4.077731	-5.646987	-3.999585
H	1.981268	-4.506896	-3.522391
H	3.448057	-8.238513	-0.653884
H	4.837590	-7.535498	-2.580184
H	1.358604	-7.069641	-0.140948
H	-1.822191	-2.424621	1.065739
N	-1.899586	-1.240021	-2.774867
O	-4.045109	-0.924856	-1.666589
C	-0.690552	-1.204184	-3.493350
C	0.421735	-0.540399	-2.993691
C	1.619298	-0.547805	-3.688789
C	1.717143	-1.232992	-4.892271
C	0.600918	-1.886202	-5.405922
C	-0.594472	-1.864690	-4.713396
H	-1.465364	-2.371980	-5.105901
H	0.349756	-0.017986	-2.048842
H	2.468301	-0.017477	-3.281060
H	0.657587	-2.415267	-6.348736
C	3.002254	-1.309240	-5.649544
F	2.865176	-0.863929	-6.910492
F	3.441171	-2.574700	-5.747849
F	3.980360	-0.597585	-5.088758
H	1.723997	3.771195	-0.838521
C	-5.625538	1.894404	1.181372
C	-5.068574	1.190515	2.244460
C	-3.216350	0.572178	0.848621
C	-3.866843	0.517117	2.069205
C	-4.976389	1.936484	-0.038065
C	-3.757808	1.290308	-0.209489
C	-2.145948	2.386516	-1.633481
C	-5.760479	1.218462	3.567841
N	-3.077817	1.392827	-1.437166
F	-7.070285	0.941818	3.454108
F	-5.684236	2.434097	4.135680
F	-5.250370	0.348404	4.439846
H	-2.273301	0.058561	0.714203
H	-5.401755	2.483991	-0.868174
H	-3.427770	-0.052978	2.875776
H	-6.567483	2.414683	1.298508
C	0.775089	3.254216	-0.710971
H	0.645225	3.024802	0.346078
C	-1.695023	3.370986	-0.807742
C	-0.407600	4.101908	-1.194182
H	-1.430567	5.444805	-4.243651
C	-2.145029	3.793497	0.480441
S	-0.434478	5.641378	-0.185792
S	1.261057	4.936592	-3.319521
C	3.533375	2.745375	-7.418324
C	-0.621345	3.069205	-3.425646
C	-0.385232	4.375791	-2.697820
C	1.201862	3.806729	-4.679620
C	2.559699	2.711082	-6.437761
C	3.899926	5.070430	-6.974127
C	2.922449	5.038864	-5.992336
C	2.228358	3.859894	-5.711638
C	-1.549810	2.210635	-2.954102
C	0.183358	2.925623	-4.592182
C	-1.433696	5.393177	-3.155520
C	4.210028	3.926833	-7.693849
O	-1.985251	1.147585	-3.599169
C	-1.582363	4.928280	0.950980
H	-2.890158	3.259960	1.047363
H	2.681828	5.942964	-5.446826
H	-1.235100	6.388484	-2.761904
H	4.977833	3.951851	-8.456109
H	4.421439	5.997073	-7.177139
H	3.776490	1.838299	-7.956080
H	2.061981	1.776814	-6.215353
H	0.002966	2.148841	-5.322512
H	0.823652	2.314858	-1.264350
C	-1.856951	5.574554	2.226580
C	-1.101834	6.666806	2.661987
C	-2.890974	5.125255	3.059312
H	-0.285110	7.032322	2.052264
H	-3.507494	4.287556	2.760830
C	-1.367488	7.284370	3.874045
C	-3.150066	5.738826	4.269136
H	-2.428540	5.087976	-2.828586
C	-2.391179	6.825824	4.686801
H	-0.764359	8.128170	4.183834
H	-3.953950	5.364730	4.890010
H	-2.597625	7.306479	5.634086
Si	-3.081081	0.113614	-2.700974
F	-4.227129	0.181564	-3.850451

1-cc-Et$_3$PO

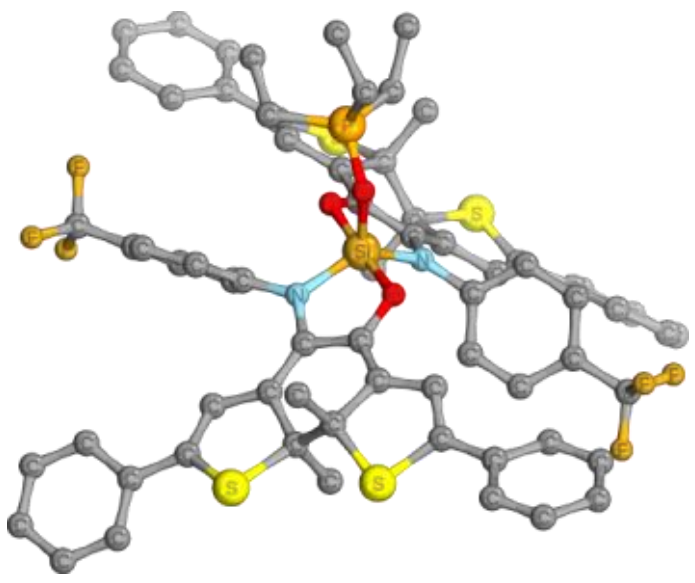

144

xyz, charge: 0, multiplicity: 1

C	-5.238350	-2.738661	-0.139796
C	-5.574065	-3.762557	0.673925
C	-3.977173	-2.896787	-0.788453
C	-3.435850	-4.306777	-0.694855
S	-4.361557	-5.042321	0.721252
H	-5.846607	-1.851061	-0.250460
C	-6.765229	-3.857762	1.510388
C	-7.280731	-5.093753	1.902991
C	-8.416552	-5.166596	2.693240
C	-9.056220	-4.009237	3.109504
C	-8.547602	-2.773246	2.732403
C	-7.413275	-2.698089	1.945729
H	-6.798389	-6.006147	1.575115
H	-8.802399	-6.134448	2.984824
H	-9.939316	-4.068288	3.731637
H	-9.025134	-1.861240	3.065591
H	-7.011569	-1.727018	1.688800
C	-3.269844	-1.988264	-1.483965
C	-3.833883	-5.072814	-1.961215
H	-3.578346	-6.128472	-1.897174
H	-3.328724	-4.656999	-2.834834
H	-4.911560	-4.992431	-2.106413
H	-0.458088	-3.537780	0.947868
S	-1.098248	-5.861751	-0.627101
C	3.565817	-6.261463	-2.857035
C	2.991651	-7.688505	-1.019948
C	3.872847	-7.333306	-2.028780
C	2.391541	-5.554738	-2.677970
C	0.030195	-3.871791	-1.878396
C	-1.271152	-3.366236	-1.556568
C	1.498904	-5.895434	-1.656322
C	-1.925250	-4.226614	-0.472224
C	1.816610	-6.978159	-0.834538
C	-1.922142	-2.246201	-1.959978
C	-1.541179	-3.636731	0.890137
C	0.270275	-5.133476	-1.462472
H	0.781945	-3.295754	-2.394208
H	-1.872277	-4.267657	1.712353
H	4.241699	-5.979295	-3.653538
H	2.157638	-4.739385	-3.350615
H	3.219944	-8.521318	-0.367971
H	4.790073	-7.888502	-2.173063
H	1.148299	-7.255848	-0.029163
H	-1.982091	-2.647280	1.021300
N	-1.512845	-1.174693	-2.741057
O	-3.734684	-0.788886	-1.832814
C	-0.255478	-1.186668	-3.379714
C	0.819254	-0.483361	-2.855382
C	2.047866	-0.497484	-3.494596
C	2.216329	-1.233898	-4.658488
C	1.143425	-1.945856	-5.184826
C	-0.083883	-1.915709	-4.550986
H	-0.919162	-2.470483	-4.957848
H	0.694356	0.082602	-1.941511
H	2.866698	0.068445	-3.073160
H	1.257375	-2.522975	-6.093352
C	3.522984	-1.256192	-5.386592
F	3.445505	-0.588788	-6.549467
F	3.894568	-2.506312	-5.692028
F	4.512601	-0.706647	-4.683808
H	2.004950	4.196649	-0.971287
C	-5.557512	2.590298	0.571455
C	-5.537261	1.656741	1.601609
C	-3.570128	0.661158	0.642258
C	-4.535736	0.694078	1.632590
C	-4.599975	2.543423	-0.425688
C	-3.601780	1.571576	-0.408351
C	-1.775230	2.552175	-1.691512
C	-6.621050	1.651234	2.631505
N	-2.667110	1.510330	-1.454470
F	-7.663188	0.895909	2.235327
F	-7.105370	2.874820	2.862507
F	-6.210619	1.154601	3.799371
H	-2.783444	-0.079977	0.686665
H	-4.612987	3.274046	-1.224608
H	-4.492025	-0.034678	2.430884
H	-6.319497	3.357389	0.535166
C	1.094645	3.622548	-0.813252
H	0.992711	3.423943	0.252924
C	-1.373606	3.567425	-0.888671
C	-0.146204	4.373298	-1.314230
H	-1.276433	5.532438	-4.402763
C	-1.814859	3.975911	0.411722
S	-0.276983	5.937529	-0.355268
S	1.444685	5.261617	-3.473137
C	3.938968	3.096376	-7.433852
C	-0.287297	3.250155	-3.505739
C	-0.149806	4.597090	-2.826120
C	1.477069	4.079071	-4.783147
C	2.970961	3.020617	-6.449287
C	4.017885	5.478037	-7.174600
C	3.046925	5.404880	-6.188972
C	2.501608	4.175245	-5.816908
C	-1.152045	2.357477	-2.991109
C	0.531403	3.124109	-4.667308
C	-1.274984	5.514618	-3.313622
C	4.466783	4.326522	-7.802995
O	-1.501889	1.218400	-3.587362
C	-1.330802	5.167147	0.825080
H	-2.462296	3.381193	1.037606
H	2.696057	6.312729	-5.714248
H	-1.153623	6.534753	-2.954290
H	5.229410	4.385004	-8.568222
H	4.424977	6.441421	-7.452305
H	4.294293	2.187757	-7.901863
H	2.593302	2.052649	-6.147890
H	0.419633	2.311607	-5.371682
H	1.200635	2.668858	-1.332809
C	-1.613639	5.828167	2.094354
C	-0.727919	6.759420	2.639233
C	-2.788827	5.541944	2.796238
H	0.192614	6.991557	2.118703
H	-3.509537	4.850650	2.378228
C	-1.001063	7.372488	3.851275
C	-3.056433	6.151176	4.008059
H	-2.244013	5.146711	-2.973290
C	-2.163423	7.070397	4.543088
H	-0.297950	8.087572	4.257574
H	-3.972592	5.915639	4.533602
H	-2.376394	7.550651	5.488836
Si	-2.592240	0.237303	-2.693559
O	-3.786363	0.412398	-3.979937
P	-5.190063	-0.251848	-4.169930
C	-6.387976	0.309102	-2.924378
H	-5.840945	0.435954	-1.990392
H	-6.719271	1.305444	-3.228202

C -7.576843 -0.623359 -2.709810
H -7.260876 -1.607350 -2.365076
H -8.183184 -0.753314 -3.605076
H -8.223742 -0.208891 -1.938246
C -5.715142 0.380353 -5.791392
H -5.368719 1.416614 -5.812424
H -5.128943 -0.144348 -6.549794
C -7.205497 0.321779 -6.108222
H -7.795499 0.899580 -5.397753
H -7.597044 -0.693111 -6.124012
H -7.382964 0.751425 -7.092818
C -5.066804 -2.065022 -4.231491
H -4.044862 -2.268033 -4.564066
H -5.130677 -2.450126 -3.213345
C -6.064474 -2.785078 -5.132102
H -5.983450 -2.481096 -6.174822
H -7.094969 -2.641752 -4.811429
H -5.863814 -3.854689 -5.091619

1-cc-pyridine

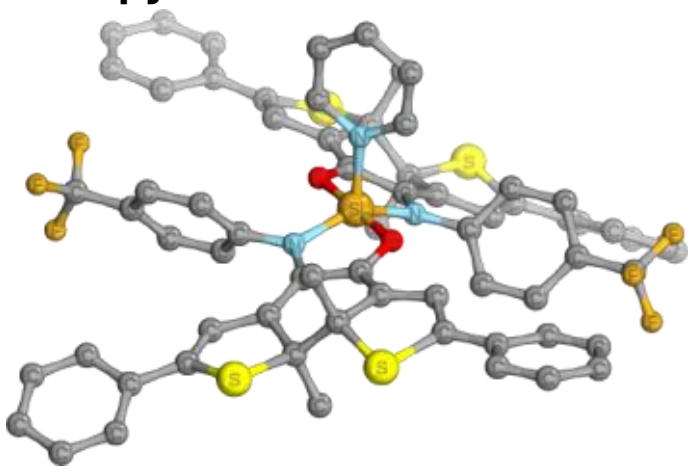

132
xyz, charge: 0, multiplicity: 1
C -4.900495 -2.958764 0.576545
C -5.290440 -4.065029 1.245071
C -3.732669 -3.139327 -0.223733
C -3.370386 -4.597604 -0.421201
S -4.248883 -5.460599 0.953111
H -5.408222 -2.007611 0.658870
C -6.415448 -4.177465 2.167641
C -7.000675 -5.412259 2.452677
C -8.073648 -5.502893 3.324981
C -8.582557 -4.364605 3.930503
C -8.005225 -3.130648 3.659816
C -6.931956 -3.038430 2.793562
H -6.625048 -6.308759 1.975404
H -8.514265 -6.469673 3.529798
H -9.418230 -4.437383 4.613743
H -8.384171 -2.234997 4.134261
H -6.478448 -2.072098 2.617667
C -2.970064 -2.211722 -0.825793
C -3.975180 -5.083250 -1.742344
H -3.852449 -6.156190 -1.875571
H -3.503939 -4.580450 -2.588164
H -5.040575 -4.856219 -1.755249
H -0.182137 -4.462368 0.989803
S -1.250611 -6.395202 -0.855859
C 3.072287 -6.952590 -3.644644
C 2.577174 -8.527237 -1.906751
C 3.360792 -8.138524 -2.982093
C 2.012649 -6.164407 -3.234808
C 0.008188 -4.356118 -1.890030
C -1.186331 -3.773959 -1.352919
C 1.222716 -6.538861 -2.143520
C -1.847241 -4.727995 -0.353885
C 1.517668 -7.737169 -1.490980
C -1.725846 -2.539297 -1.496096
C -1.269857 -4.425998 1.034803
C 0.119807 -5.689657 -1.705035
H 0.782684 -3.785782 -2.379792
H -1.607002 -5.147009 1.776404
H 3.670538 -6.645263 -4.492315
H 1.781299 -5.257827 -3.779601
H 2.792547 -9.449537 -1.383553
H 4.187050 -8.756899 -3.306674
H 0.924904 -8.043476 -0.638282
H -1.566335 -3.430883 1.368430
N -1.250347 -1.401699 -2.146897
O -3.254352 -0.904539 -0.860678
C -0.223809 -1.500959 -3.106745
C 0.996604 -0.860714 -2.923229
C 1.973032 -0.921683 -3.900142
C 1.750621 -1.637469 -5.071728
C 0.544504 -2.302648 -5.248591
C -0.434934 -2.231882 -4.272168
H -1.370755 -2.760081 -4.406924
H 1.177765 -0.306999 -2.012657
H 2.911482 -0.408048 -3.737510
H 0.354019 -2.875646 -6.145633
C 2.792417 -1.631296 -6.145533
F 2.765509 -0.483426 -6.843161
F 2.622443 -2.620366 -7.026400
F 4.025690 -1.746613 -5.645879
H 1.692272 5.003164 -0.928341
C -5.448449 2.385535 1.131098
C -5.280440 1.635210 2.288514
C -3.184310 0.815796 1.440339
C -4.139833 0.855040 2.439713
C -4.495490 2.335174 0.128545
C -3.361268 1.539981 0.267700
C -1.717474 2.586740 -1.194133
C -6.346309 1.610836 3.338139
N -2.427849 1.459787 -0.784638
F -7.203032 0.594831 3.138265
F -7.075885 2.729810 3.345336
F -5.841814 1.453026 4.563743
H -2.296758 0.211476 1.565915
H -4.622942 2.928253 -0.768598
H -3.983733 0.273413 3.338360
H -6.321750 3.009592 0.997856
C 0.912642 4.304193 -0.633299
H 0.868197 4.278418 0.454753
C -1.505611 3.776938 -0.582025
C -0.457299 4.713545 -1.188791
H -1.846427 5.108761 -4.365607
C -2.007480 4.314315 0.648412
S -0.869191 6.367065 -0.492427
S 0.891468 5.513276 -3.544655
C 3.492249 3.259567 -7.417489
C -0.415649 3.234966 -3.166801
C -0.530514 4.677809 -2.716839
C 1.108195 4.167542 -4.666711
C 2.611982 3.153246 -6.356944
C 3.287268 5.646596 -7.350474
C 2.401145 5.542233 -6.290198
C 2.044022 4.293267 -5.779268
C -1.069437 2.298175 -2.459930
C 0.375947 3.080029 -4.344279
C -1.821332 5.272442 -3.288869
C 3.835030 4.508042 -7.920519
O -1.178668 1.014374 -2.823328
C -1.752338 5.627216 0.837954
H -2.524620 3.725646 1.390546
H 1.969963 6.440515 -5.866193
H -1.893125 6.342303 -3.103786
H 4.529353 4.591233 -8.746019
H 3.548553 6.624499 -7.732831
H 3.921236 2.362713 -7.844701
H 2.380121 2.173472 -5.960903
H 0.406386 2.157573 -4.907738
H 1.193159 3.311783 -0.988256
C -2.145579 6.430938 1.990879
C -1.432882 7.575153 2.353607
C -3.253913 6.066278 2.761552
H -0.566182 7.872530 1.776794
H -3.844487 5.203841 2.479380
C -1.807507 8.321390 3.459287
C -3.622609 6.810214 3.867230
H -2.694797 4.792272 -2.845323
C -2.900590 7.942034 4.222693
H -1.238163 9.201806 3.726612
H -4.484458 6.511284 4.449273
H -3.192914 8.526296 5.085029
Si -2.229502 0.054557 -1.856155
N -3.609361 0.141170 -3.233596
C -3.267556 0.169405 -4.527090
C -4.902346 0.170679 -2.890248
C -4.216906 0.216743 -5.526271
C -5.901993 0.240821 -3.837677
C -5.557348 0.258743 -5.179293
H -2.211903 0.158148 -4.752939
H -5.127498 0.131557 -1.835065
H -3.899484 0.228860 -6.558525
H -6.933323 0.271780 -3.518531
H -6.321901 0.304741 -5.942905

2-oo

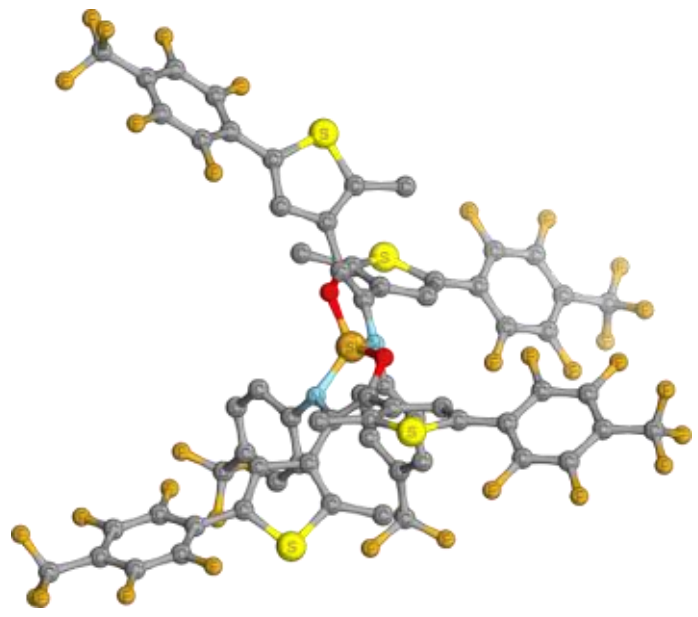

133
xyz, charge: 0, multiplicity: 1
C 1.013635 5.344925 0.241816
C 0.264702 4.229610 0.011962
C 2.257903 5.340352 -0.444699
C 2.418027 4.230937 -1.236726
S 1.082436 3.171667 -1.079648
H 0.712160 6.144741 0.901162
C -1.055302 3.911230 0.542464
C -2.028886 4.891823 0.716809
C -3.274822 4.591594 1.225189
C -3.627152 3.294456 1.581394
C -2.667762 2.307671 1.403941
C -1.416869 2.618578 0.899421
F -1.774740 6.150499 0.381749
F -4.150816 5.576717 1.361592
C -5.011051 3.048498 2.128026
F -2.898105 1.042345 1.714629
F -0.537234 1.631865 0.763040
C 3.220671 6.417125 -0.288910
C 3.535574 3.878536 -2.155230
H 4.061982 4.777610 -2.470574
H 3.169997 3.373551 -3.049034
H 4.264113 3.220822 -1.677367
H 3.132884 3.419836 1.486298
S 6.272323 2.896616 0.832475
C 10.704608 3.609091 -1.627729
C 9.792133 1.422015 -1.718546
C 10.871326 2.265086 -1.943698
C 9.520225 4.091510 -1.111809
C 6.566256 4.952276 -0.638426
C 5.363295 5.155981 0.088882
C 8.428865 3.255414 -0.891250
C 5.089808 4.129017 0.955584
C 8.604254 1.915038 -1.206853
C 4.550370 6.353441 -0.075619
C 3.960423 3.988127 1.915467
C 7.175134 3.768105 -0.352071
H 6.940583 5.660016 -1.363142
H 3.573624 4.969687 2.186690
F 11.702746 4.460969 -1.811112
F 9.441263 5.381755 -0.815327
F 9.845031 0.127828 -1.988487
C 12.199108 1.816927 -2.501932
F 7.601130 1.063558 -1.021882
H 4.271723 3.482456 2.829165
N 5.156202 7.626933 -0.099178
O 2.709482 7.692506 -0.384470
C 6.386417 7.943603 0.497032
C 7.164492 8.940339 -0.083553
C 8.325965 9.372881 0.531931
C 8.737689 8.798152 1.724803
C 7.973683 7.786684 2.294861

C 6.806483 7.363122 1.691284
H 6.206153 6.604142 2.171470
H 6.863657 9.373572 -1.029709
H 8.911442 10.150716 0.061189
H 8.268684 7.334393 3.231588
C 9.951868 9.306528 2.434781
F 10.596288 8.336104 3.085844
F 9.626080 10.236199 3.347815
F 10.822994 9.877618 1.598466
H 6.301837 12.748770 -0.581062
C 4.335344 8.471757 4.164458
C 5.457932 9.111225 4.669477
C 5.563391 10.472677 2.688464
C 6.062009 10.117921 3.926554
C 3.824210 8.834995 2.931423
C 4.442201 9.826364 2.174862
C 4.001928 11.316446 0.221305
C 6.057574 8.668522 5.966015
N 3.963217 10.068971 0.878396
F 6.966881 7.698675 5.772749
F 5.140093 8.172500 6.801065
F 6.681777 9.663242 6.598981
H 6.072227 11.229183 2.108957
H 2.930774 8.351352 2.555582
H 6.945599 10.620272 4.294687
H 3.838448 7.696278 4.731446
C 5.956869 13.733895 -0.269443
H 5.596105 14.249342 -1.161682
C 4.037792 12.552393 0.991392
C 4.890356 13.601600 0.760940
H 2.325801 14.366521 -0.366222
C 3.182682 12.776488 2.103186
S 4.600324 14.876502 1.866686
S 3.242658 14.307877 -3.471511
C 6.062678 15.116943 -6.988777
C 3.833537 12.218192 -2.140314
C 2.989326 13.299923 -2.111213
C 4.450824 13.263030 -4.125909
C 5.444612 14.837934 -5.782093
C 6.033732 12.803970 -7.504515
C 5.411911 12.535255 -6.303652
C 5.101639 13.547713 -5.398928
C 3.929349 11.189427 -1.118977
C 4.643357 12.190065 -3.307743
C 1.949167 13.660536 -1.108815
C 6.374885 14.099024 -7.878735
O 3.934856 9.891403 -1.579316
C 3.360590 13.996172 2.682712
H 2.449259 12.055956 2.433552
F 5.100351 11.275329 -6.026795
H 1.614280 12.771835 -0.576887
C 7.050907 14.309365 -9.210354
F 6.296424 11.789443 -8.315770
F 6.347211 16.383460 -7.244891
F 5.181372 15.854203 -4.967319
H 5.358944 11.406153 -3.504532
H 6.814481 14.288615 0.110357
C 2.675322 14.539065 3.849350
C 2.370869 13.741350 4.949242
C 2.296222 15.872062 3.929854
F 2.719054 12.461667 4.960565
F 2.555368 16.687611 2.913377
C 1.717023 14.252059 6.050749
C 1.645740 16.393670 5.034730
H 1.079811 14.113406 -1.584908
C 1.338330 15.588270 6.122348
F 1.460082 13.436064 7.062533
F 1.326512 17.677213 5.000184
C 0.628676 16.069119 7.363569
Si 3.924122 8.819227 -0.314341
F -5.937833 3.406552 1.238692
F -5.211941 3.765910 3.234402
F -5.225322 1.775866 2.435254
F 12.473358 2.465283 -3.634718
F 12.233963 0.517960 -2.769085
F 13.182079 2.074125 -1.638682
F 7.330476 15.583648 -9.451020
F 6.275353 13.875561 -10.204534
F 8.198203 13.631299 -9.265450
F 1.392809 15.887944 8.441002
F 0.313719 17.356735 7.311113
F -0.503027 15.388239 7.549069

2-oo-F

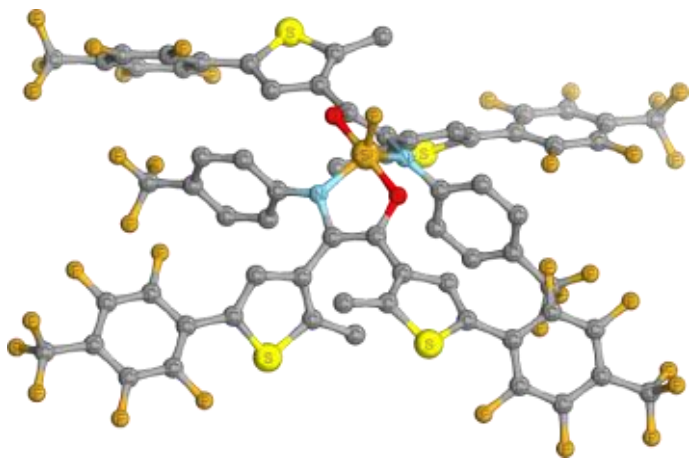

134
xyz, charge: -1, multiplicity: 1
C -5.114262 -3.351218 -0.261672
C -5.436212 -4.498491 0.391444
C -4.303057 -3.559187 -1.410702
C -4.070281 -4.889476 -1.647633
S -4.772893 -5.869312 -0.420087
H -5.404859 -2.368638 0.076456
C -6.224379 -4.618768 1.610904
C -7.457505 -3.988853 1.739904
C -8.189525 -4.069769 2.904750
C -7.737452 -4.793465 4.002356
C -6.510831 -5.431833 3.878880
C -5.779695 -5.341112 2.708236
F -7.961587 -3.299574 0.724995
F -9.365524 -3.456645 2.957393
C -8.599257 -4.842030 5.234799
F -5.989155 -6.142532 4.870430
F -4.605157 -5.960348 2.653263
C -3.801370 -2.427299 -2.180185
C -3.348733 -5.531943 -2.780123
H -3.317623 -4.850242 -3.628519
H -3.844894 -6.449518 -3.098659
H -2.317811 -5.783487 -2.522537
H -2.226665 -4.076133 0.478526
S 0.395200 -4.584614 -1.345551
C 3.076814 -4.816091 -5.720327
C 3.032317 -6.774767 -4.389995
C 3.578863 -6.087971 -5.465440
C 2.083322 -4.255554 -4.946003
C -0.547827 -3.538128 -3.466273
C -1.393094 -3.098147 -2.411618
C 1.519193 -4.940756 -3.869885
C -0.972891 -3.560876 -1.189441
C 2.030053 -6.210169 -3.622497
C -2.540969 -2.225914 -2.620486
C -1.544218 -3.286454 0.157099
C 0.467513 -4.350872 -3.056820
H -0.721110 -3.267207 -4.496800
H -2.117748 -2.359872 0.135180
F 3.567483 -4.104755 -6.727169
F 1.679725 -3.029047 -5.243337
F 3.434750 -7.995394 -4.062246
C 4.665281 -6.626020 -6.356087
F 1.542400 -6.929370 -2.615753
H -0.763756 -3.194064 0.913246
N -2.439800 -1.033132 -3.360022
O -4.663049 -1.452411 -2.459781
C -1.177723 -0.547268 -3.707330
C -0.869707 -0.281144 -5.039309
C 0.390717 0.156830 -5.408601
C 1.375269 0.343285 -4.446879
C 1.075821 0.093728 -3.111293
C -0.182504 -0.343552 -2.747872
H -0.405022 -0.531535 -1.705598
H -1.633957 -0.427468 -5.790158
H 0.595177 0.344745 -6.453276
H 1.823833 0.243520 -2.343064
C 2.733786 0.845733 -4.802066
F 3.706434 0.095407 -4.267618
F 2.936743 2.096629 -4.346164
F 2.943247 0.881273 -6.121871
H -0.795358 2.402578 -1.176926
C -6.480112 -0.196204 0.901232
C -5.603988 -0.338350 1.969490
C -3.790978 0.354285 0.548738
C -4.253106 -0.063938 1.780889
C -6.012028 0.222876 -0.332516
C -4.664392 0.513942 -0.530305
C -3.500136 2.188433 -1.833652
C -6.065097 -0.817813 3.304299
N -4.219360 0.980809 -1.768603
F -5.596499 -2.049982 3.579762
F -7.396917 -0.885576 3.397627
F -5.638846 -0.029349 4.299949
H -2.737598 0.562019 0.413022
H -6.696469 0.334748 -1.162142
H -3.549867 -0.179322 2.595932
H -7.534305 -0.404331 1.018821
C -0.845485 3.341947 -0.626485
H -0.484780 4.124235 -1.297716
C -3.412390 3.090774 -0.693462
C -2.242798 3.594241 -0.180683
H -3.499983 5.752009 -1.686780
C -4.546862 3.522943 0.045868
S -2.541780 4.644931 1.142537
S -1.198787 5.835583 -3.957363
C 3.239960 5.466808 -5.320058
C -2.172918 3.512659 -3.516693
C -2.458868 4.841571 -3.337031
C -0.302848 4.470859 -4.516364
C 2.014218 5.364403 -4.687353
C 2.443215 4.038692 -7.036908
C 1.222911 3.946441 -6.403229
C 0.975553 4.603198 -5.202567
C -2.956903 2.373311 -3.055850
C -0.954747 3.315274 -4.222803
C -3.662892 5.471875 -2.729570
C 3.481565 4.801206 -6.514170
O -3.139452 1.373889 -3.915432
C -4.246854 4.369237 1.071942
H -5.551716 3.221964 -0.209262
F 0.270559 3.219347 -6.972137
H -4.495469 4.770694 -2.754442
C 4.780815 4.859833 -7.270808
F 2.609175 3.398020 -8.187416
F 4.166959 6.215647 -4.736669
F 1.845777 6.011287 -3.538784
H -0.571041 2.335172 -4.460190
H -0.158149 3.286553 0.218453
C -5.162568 4.962975 2.033208
C -6.267533 4.264037 2.519337
C -4.991042 6.250360 2.529979
F -6.499262 3.020867 2.123805
F -3.962735 6.983385 2.113020
C -7.139064 4.828253 3.426478
C -5.856691 6.819062 3.446265
H -3.958293 6.370441 -3.272610
C -6.959466 6.118165 3.914920
F -8.167861 4.103125 3.846323
F -5.594459 8.057150 3.843340
C -7.954589 6.659259 4.904857
Si -3.940019 -0.039882 -3.229449
F -9.784640 -5.398444 4.963784
F -8.832417 -3.616639 5.706361
F -8.055714 -5.546354 6.222657
F 4.256364 -6.681406 -7.626418
F 5.056003 -7.850429 -6.016987
F 5.746225 -5.843313 -6.318194
F 5.703111 5.601240 -6.664941
F 4.597961 5.380887 -8.488700
F 5.298150 3.641341 -7.431940
F -7.992280 5.905136 6.005957
F -7.680962 7.901695 5.289967
F -9.185246 6.670438 4.385885
F -5.041694 -0.064004 -4.428938

2-oo-Et_3PO

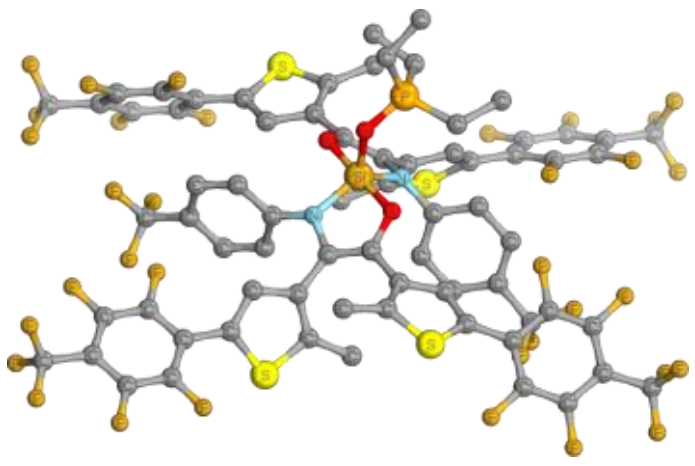

156
xyz, charge: 0, multiplicity: 1
C -5.238177 -3.239056 -0.227797
C -5.585484 -4.400318 0.384058
C -4.522251 -3.432354 -1.441844
C -4.377143 -4.758988 -1.757536
S -5.061343 -5.758676 -0.538471
H -5.447599 -2.262867 0.182525
C -6.306567 -4.542252 1.644558
C -7.504287 -3.874085 1.867279
C -8.169820 -3.972993 3.070035
C -7.679476 -4.753450 4.110702
C -6.487308 -5.430097 3.893010
C -5.823216 -5.321357 2.683338
F -8.035429 -3.125118 0.907687
F -9.313102 -3.317153 3.214350
C -8.468106 -4.814658 5.394277
F -5.935421 -6.194145 4.823084
F -4.679117 -5.977868 2.529663
C -4.022068 -2.287220 -2.187923
C -3.747912 -5.381690 -2.954912
H -3.733045 -4.670787 -3.780203
H -4.293569 -6.268810 -3.277926
H -2.714028 -5.678931 -2.768334
H -2.255626 -4.116819 0.189040
S 0.169388 -4.587403 -1.901603
C 2.311485 -4.755707 -6.579304
C 2.409179 -6.729119 -5.273904
C 2.823028 -6.036560 -6.402936
C 1.433024 -4.193313 -5.678518
C -0.963033 -3.434847 -3.864934
C -1.673801 -3.017063 -2.709361
C 1.000895 -4.882048 -4.546289
C -1.145592 -3.544518 -1.557803
C 1.520639 -6.159975 -4.378241
C -2.812613 -2.108984 -2.754024
C -1.577148 -3.330939 -0.149232
C 0.065480 -4.290560 -3.599884
H -1.238240 -3.123759 -4.860834
H -2.113009 -2.386539 -0.058573
F 2.674153 -4.037286 -7.633289
F 1.007832 -2.956522 -5.913198
F 2.830978 -7.955729 -5.011579
C 3.778961 -6.579682 -7.433970
F 1.155246 -6.881679 -3.323839
H -0.727090 -3.310651 0.532679
N -2.720584 -0.853072 -3.402416
O -4.852200 -1.236285 -2.292382
C -1.426340 -0.333648 -3.631155
C -0.926440 -0.215107 -4.919318
C 0.342851 0.291097 -5.153782
C 1.136398 0.682820 -4.087463
C 0.651558 0.555288 -2.789041
C -0.614196 0.050102 -2.566141
H -0.987367 -0.049454 -1.554308
H -1.537397 -0.533059 -5.754299
H 0.701989 0.368501 -6.170050
H 1.259709 0.852997 -1.944505
C 2.493478 1.275948 -4.299312
F 3.420186 0.677402 -3.545592
F 2.511517 2.576986 -3.967371
F 2.894046 1.192377 -5.569841
H -0.946537 2.711169 -1.072962
C -6.457402 -0.083711 1.245813
C -5.518589 -0.291630 2.245826
C -3.785861 0.456109 0.757108
C -4.178549 -0.017428 1.993841
C -6.058248 0.394267 0.008108
C -4.721861 0.668467 -0.253609
C -3.674556 2.411668 -1.600116
C -5.906170 -0.846496 3.579021
N -4.331898 1.164876 -1.509409
F -5.468162 -2.109498 3.725858
F -7.228638 -0.871814 3.757705
F -5.378196 -0.143812 4.585376
H -2.741393 0.669994 0.570044
H -6.791455 0.559239 -0.769556
H -3.431405 -0.174411 2.760952
H -7.505082 -0.284249 1.419634
C -1.005487 3.629778 -0.490328
H -0.702885 4.445233 -1.150538
C -3.565423 3.296005 -0.446651
C -2.388852 3.820494 0.025679
H -3.592358 5.996284 -1.434517
C -4.682117 3.685095 0.342085
S -2.662105 4.826504 1.386985
S -1.490650 6.062560 -3.901512
C 2.796100 5.721268 -5.648358
C -2.439995 3.756359 -3.356851
C -2.703336 5.089268 -3.170814
C -0.644470 4.686718 -4.508229
C 1.610188 5.655129 -4.937582
C 1.978020 4.050501 -7.116290
C 0.796939 3.999343 -6.409779
C 0.581687 4.792695 -5.287178
C -3.189893 2.622885 -2.838477
C -1.278193 3.539176 -4.145122
C -3.844622 5.732424 -2.463546
C 3.007245 4.913044 -6.756624
O -3.374113 1.597889 -3.689457
C -4.359935 4.514286 1.373406
H -5.690750 3.368765 0.122937
F -0.150730 3.166183 -6.831661
H -4.690565 5.048009 -2.425493
C 4.264906 4.918404 -7.586847
F 2.110921 3.264147 -8.177030
F 3.713037 6.574855 -5.220425
F 1.472263 6.440750 -3.875436
H -0.915710 2.552507 -4.388814
H -0.278476 3.572816 0.320108
C -5.251285 5.064269 2.385964
C -6.286634 4.308526 2.931539
C -5.117047 6.360052 2.867200
F -6.471531 3.052921 2.545678
F -4.151839 7.138644 2.389406
C -7.135068 4.829092 3.885699
C -5.959826 6.886992 3.830107
H -4.166227 6.643269 -2.968984
C -6.994992 6.129029 4.359640
F -8.098653 4.052425 4.360680
F -5.741709 8.136698 4.209099
C -7.963093 6.622401 5.404316
Si -4.112321 0.192818 -2.969813
F -9.689635 -5.305309 5.171244
F -8.610962 -3.598150 5.919458
F -7.897642 -5.580672 6.315996
F 3.200479 -6.615785 -8.636432
F 4.193249 -7.809259 -7.156724
F 4.860328 -5.805545 -7.529123
F 5.177604 5.770756 -7.137935
F 3.992524 5.256461 -8.849689
F 4.824331 3.708335 -7.605701
F -7.898859 5.867305 6.501628
F -7.731592 7.876564 5.771845
F -9.215829 6.566546 4.947728
O -5.403677 0.258009 -4.186650
P -5.421485 -0.293607 -5.636757
C -4.215573 0.570883 -6.687339
H -4.697567 1.493280 -7.021736
H -3.403892 0.891804 -6.032465
C -3.674815 -0.224053 -7.871476
H -4.454810 -0.557413 -8.555010
H -3.117339 -1.102826 -7.548874
H -2.988993 0.399721 -8.442477
C -5.122365 -2.078485 -5.672136
H -5.028292 -2.390927 -6.715092
H -4.148072 -2.240740 -5.203267
C -6.209471 -2.879092 -4.959142
H -7.165404 -2.819830 -5.479861
H -6.350028 -2.548198 -3.932707
H -5.925826 -3.929648 -4.925626
C -7.086371 0.032283 -6.261263
H -7.246793 1.109310 -6.170584
H -7.784433 -0.439824 -5.566601
C -7.330551 -0.437842 -7.690747
H -7.168128 -1.509523 -7.806668
H -6.694906 0.080468 -8.407677
H -8.362068 -0.236976 -7.974478

2-oc

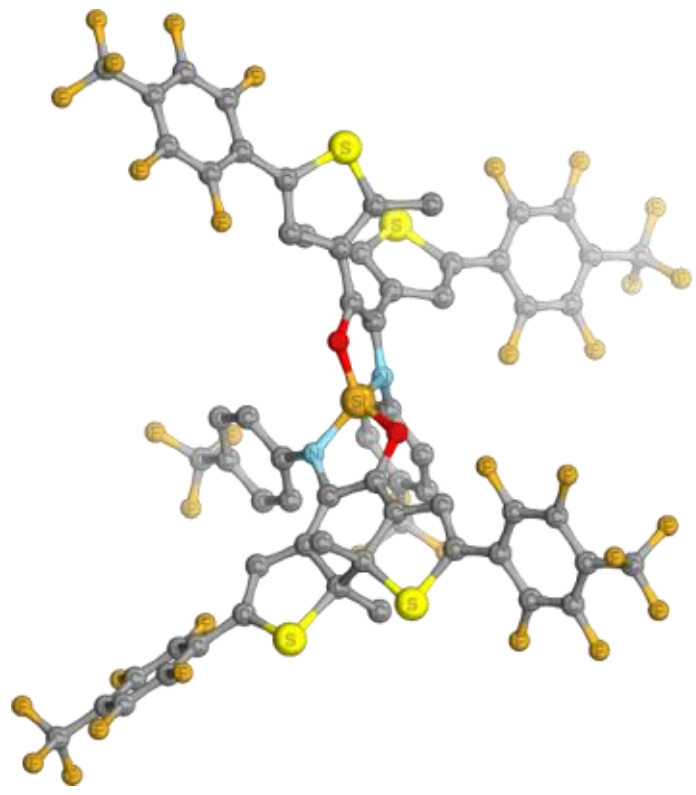

133
xyz, charge: 0, multiplicity: 1
C 1.130769 5.216708 -0.226981
C 0.426073 4.105088 -0.580815
C 2.459778 5.225640 -0.730312
C 2.734647 4.131050 -1.511534
S 1.393902 3.068458 -1.564635
H 0.735628 6.004111 0.396875
C -0.954940 3.775430 -0.249591
C -1.947066 4.751481 -0.198806
C -3.251758 4.440416 0.120846
C -3.646052 3.136499 0.400061
C -2.667329 2.154331 0.344253
C -1.358442 2.476079 0.029156
F -1.652258 6.016220 -0.471612
F -4.141860 5.421641 0.148216
C -5.093029 2.878680 0.738883
F -2.934476 0.883393 0.596561
F -0.464225 1.493342 0.002419
C 3.387491 6.299655 -0.420668
C 3.972059 3.795700 -2.268696
H 4.535136 4.700266 -2.491348
H 3.737860 3.305939 -3.213568
H 4.627804 3.130271 -1.704017
H 3.059845 3.273486 1.271882
S 6.260120 2.764071 1.058446
C 10.996421 3.517260 -0.721148
C 10.107670 1.336161 -1.002773
C 11.207747 2.181310 -1.044788
C 9.748950 3.990078 -0.371707
C 6.753703 4.843289 -0.323117
C 5.459979 5.035329 0.231423
C 8.637914 3.151857 -0.335850
C 5.069207 3.994175 1.034060
C 8.857411 1.819714 -0.657576
C 4.674421 6.234717 -0.025011
C 3.816083 3.836397 1.822506
C 7.318592 3.654982 0.027578
H 7.225802 5.561977 -0.976548
H 3.390881 4.812678 2.051805
F 12.010205 4.370004 -0.733825
F 9.625929 5.272200 -0.055735
F 10.200038 0.049549 -1.296499
C 12.603332 1.743267 -1.414437
F 7.837827 0.967922 -0.644080

H	3.997375	3.316494	2.762858
N	5.273579	7.510816	0.062162
O	2.889631	7.577146	-0.563509
C	6.425509	7.809047	0.807738
C	7.274325	8.808147	0.342870
C	8.359277	9.215599	1.099986
C	8.623808	8.610268	2.318915
C	7.790084	7.595021	2.773249
C	6.696408	7.199037	2.029939
H	6.037252	6.437174	2.420095
H	7.093024	9.263501	-0.623182
H	9.000955	9.997841	0.718495
H	7.971973	7.117961	3.726369
C	9.751491	9.085977	3.179378
F	10.326167	8.085391	3.849896
F	9.320592	9.969283	4.094735
F	10.704156	9.696646	2.471274
H	6.278727	12.833783	-0.573891
C	3.874310	8.276533	4.171135
C	4.959707	8.868043	4.798941
C	5.320928	10.248262	2.863801
C	5.675877	9.861589	4.140537
C	3.505809	8.674162	2.896565
C	4.232588	9.653362	2.230964
C	3.989162	11.193499	0.289767
C	5.404515	8.391703	6.146453
N	3.914845	9.935204	0.887825
F	6.320622	7.416581	6.034009
F	4.393404	7.889778	6.860292
F	5.960932	9.366943	6.865209
H	5.906433	10.998130	2.349798
H	2.642054	8.228248	2.419301
H	6.534453	10.325118	4.605963
H	3.295435	7.511826	4.670742
C	5.849542	13.705036	-0.077430
H	6.189581	14.595186	-0.602412
C	3.933115	12.428430	0.842800
C	4.319023	13.614115	-0.036609
H	1.787396	14.515407	-1.092414
C	3.642770	12.827701	2.180860
S	3.695509	15.091107	0.863821
S	4.273848	14.656938	-2.657819
C	5.512554	15.145613	-7.019132
C	4.057714	12.100621	-1.984131
C	3.667069	13.448044	-1.409628
C	4.350726	13.396188	-3.886396
C	5.357353	14.785429	-5.691313
C	3.964902	13.454888	-7.628136
C	3.813849	13.103683	-6.303364
C	4.513151	13.757880	-5.291945
C	4.126551	11.050231	-1.151983
C	4.232746	12.142163	-3.399998
C	2.135095	13.526147	-1.380681
C	4.817704	14.481125	-8.019770
O	4.280092	9.763050	-1.570478
C	3.507866	14.162227	2.342413
H	3.483177	12.128351	2.986706
F	2.963682	12.129290	-6.007048
H	1.723714	12.801074	-0.677357
C	4.928797	14.801891	-9.489808
F	3.264149	12.792694	-8.537431
F	6.350574	16.133835	-7.285185
F	6.057783	15.447624	-4.779709
H	4.309576	11.251427	-4.005478
H	6.235363	13.744186	0.940767
C	3.244955	14.838849	3.608787
C	3.860507	14.439438	4.793400
C	2.388812	15.928672	3.696076
F	4.719867	13.427805	4.794951
F	1.766878	16.360153	2.606653
C	3.617484	15.086573	5.986216
C	2.142002	16.585335	4.889732
H	1.741071	13.300963	-2.370737
C	2.752126	16.172417	6.065717
F	4.241780	14.656311	7.072974
F	1.300838	17.605620	4.857443
C	2.538269	16.824070	7.409553
Si	4.082052	8.696907	-0.312624
F	-5.884327	3.251877	-0.267223
F	-5.452299	3.575418	1.817939
F	-5.343768	1.600382	0.989371
F	13.039441	2.419842	-2.477746
F	12.677441	0.451597	-1.707223
F	13.449032	1.975085	-0.410331
F	5.777335	15.790263	-9.739329
F	3.742483	15.158809	-9.982565
F	5.347258	13.734272	-10.170841
F	3.689696	17.292816	7.891390
F	1.684720	17.838177	7.360968
F	2.060027	15.940793	8.286740

2-oc-F

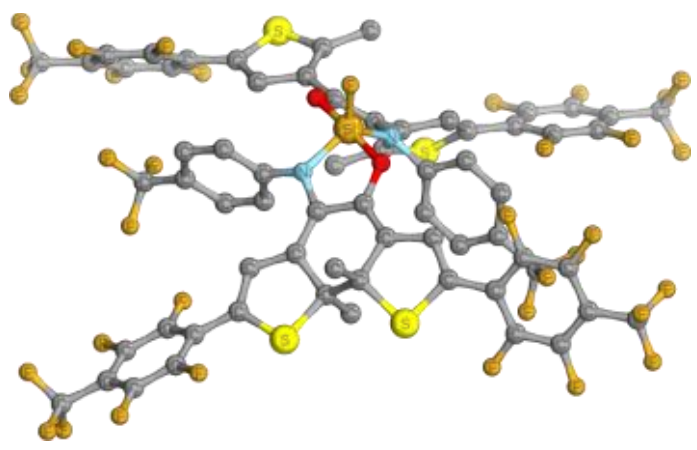

134
xyz, charge: -1, multiplicity: 1

C	-5.358111	-3.531125	-0.550288
C	-5.744484	-4.663144	0.097867
C	-4.353661	-3.750783	-1.531328
C	-4.034518	-5.078733	-1.659571
S	-4.896616	-6.034119	-0.520137
H	-5.744830	-2.549356	-0.326160
C	-6.730051	-4.770794	1.163913
C	-7.943452	-4.091753	1.102377
C	-8.862307	-4.160042	2.126783
C	-8.631919	-4.921298	3.267123
C	-7.429109	-5.611088	3.332830
C	-6.508650	-5.531277	2.303603
F	-8.246252	-3.361754	0.036460
F	-10.003205	-3.494400	1.997036
C	-9.689615	-4.952985	4.336934
F	-7.109878	-6.362392	4.378162
F	-5.368864	-6.203253	2.432574
C	-3.789270	-2.631322	-2.272044
C	-3.113680	-5.738320	-2.625603
H	-2.983993	-5.104784	-3.501501
H	-3.508285	-6.699322	-2.957159
H	-2.124270	-5.915087	-2.198833
H	-2.450259	-4.172681	0.593445
S	0.392314	-4.538354	-0.889207
C	3.576568	-4.719417	-4.917110
C	3.481348	-6.645039	-3.541248
C	4.111313	-5.954957	-4.567717
C	2.472197	-4.196126	-4.278430
C	-0.346827	-3.592304	-3.134038
C	-1.335526	-3.182859	-2.198721
C	1.824783	-4.885910	-3.253656
C	-1.041626	-3.597077	-0.923762
C	2.368846	-6.118423	-2.910661
C	-2.495875	-2.386574	-2.573389
C	-1.790039	-3.341472	0.336831
C	0.654556	-4.337108	-2.585559
H	-0.410034	-3.352264	-4.184557
H	-2.420639	-2.460614	0.221218
F	4.141747	-4.007100	-5.882829
F	2.040858	-3.002113	-4.656508
F	3.906494	-7.832683	-3.132378
C	5.320257	-6.453654	-5.312249
F	1.806146	-6.838436	-1.944468
H	-1.116254	-3.178991	1.178729
N	-2.362840	-1.229114	-3.361995
O	-4.653573	-1.704566	-2.695395
C	-1.107119	-0.632760	-3.544826
C	-0.651522	-0.357490	-4.833657
C	0.557571	0.274540	-5.036789
C	1.348716	0.643060	-3.953187
C	0.916568	0.352754	-2.668507
C	-0.300182	-0.278527	-2.467988
H	-0.636510	-0.480763	-1.459170
H	-1.268405	-0.636395	-5.677134
H	0.880583	0.483322	-6.048950
H	1.510194	0.632695	-1.809397
C	2.649721	1.328758	-4.208321
F	3.537666	0.512678	-4.796198
F	3.221089	1.784048	-3.089819
F	2.505296	2.378407	-5.032915
H	-0.947611	2.584452	-1.450871
C	-6.870791	-0.220695	0.399082
C	-6.042591	-0.516054	1.472163
C	-4.125474	0.027855	0.129576
C	-4.663075	-0.392717	1.329345
C	-6.325821	0.206292	-0.803275
C	-4.953458	0.341279	-0.947001
C	-3.752481	2.005603	-2.189683
C	-6.589336	-0.995037	2.778306
N	-4.399509	0.798026	-2.158052
F	-6.160000	-2.236570	3.064087
F	-7.925099	-1.034292	2.792771
F	-6.203974	-0.220192	3.797028
H	-3.053739	0.135781	0.020478
H	-6.967668	0.435447	-1.642672
H	-4.001552	-0.627213	2.153369
H	-7.943711	-0.318450	0.484796
C	-1.274915	3.433337	-0.847929
H	-0.461921	4.155670	-0.811430
C	-3.677535	3.012751	-1.268230
C	-2.558327	4.044474	-1.422929
H	-3.722953	6.231781	-2.922766
C	-4.404498	3.218367	-0.063454
S	-3.053365	5.443749	-0.336662
S	-0.907434	5.434903	-3.237792
C	2.650105	5.879004	-6.062618
C	-2.277573	3.219298	-3.730408
C	-2.435491	4.464002	-2.888172
C	-0.572807	4.478271	-4.691674
C	1.637405	5.514793	-5.194780
C	1.409227	4.959727	-7.853278
C	0.401028	4.607641	-6.985052
C	0.479623	4.864285	-5.613817
C	-3.002654	2.113334	-3.439082
C	-1.360976	3.381815	-4.800086
C	-3.636885	5.260677	-3.406267
C	2.563993	5.603501	-7.420228
O	-3.098215	1.058500	-4.205357
C	-4.179661	4.409632	0.544266
H	-5.133937	2.518790	0.311472
F	-0.678578	4.027480	-7.499944
H	-4.562834	4.709452	-3.235501
C	3.617134	5.956572	-8.431662
F	1.252767	4.686750	-9.145112
F	3.706881	6.488016	-5.541281
F	1.806484	5.794954	-3.907399
H	-1.259739	2.640180	-5.577270
H	-1.468573	3.083449	0.166076
C	-4.775632	4.849390	1.792956
C	-5.042044	3.967923	2.844412
C	-5.092703	6.183978	2.035657
F	-4.731972	2.681130	2.736331
F	-4.880217	7.091321	1.088333
C	-5.598423	4.394235	4.029878
C	-5.644462	6.619778	3.226061
H	-3.526170	5.418440	-4.478404
C	-5.918102	5.729669	4.255963
F	-5.811769	3.496811	4.985380
F	-5.912713	7.914620	3.333746
C	-6.522333	6.113602	5.577320
Si	-3.910680	-0.340796	-3.495176
F	-10.834735	-5.449200	3.857746
F	-9.946199	-3.726369	4.793402
F	-9.348779	-5.698752	5.382935
F	5.063232	-6.562118	-6.618199
F	5.732124	-7.645794	-4.893487

F	6.346534	-5.611272	-5.174820
F	4.680207	6.545807	-7.893776
F	3.132322	6.792145	-9.356826
F	4.053090	4.868292	-9.072234
F	-5.696809	5.818970	6.586206
F	-6.799189	7.410880	5.665734
F	-7.663512	5.453074	5.792697
F	-4.884304	-0.468443	-4.789262

2-oc-Et$_3$PO

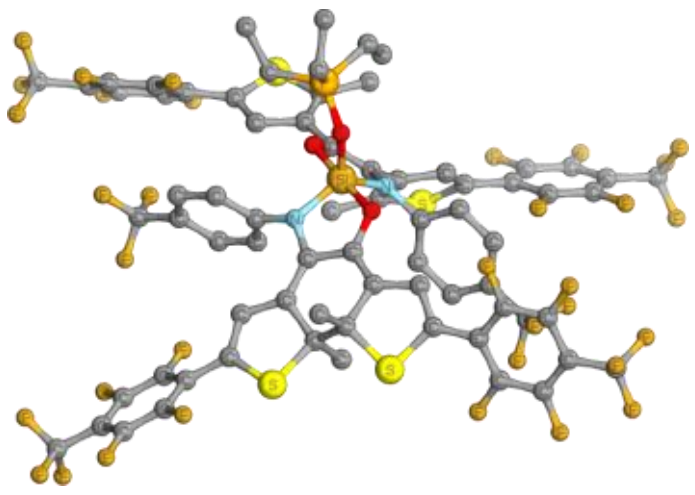

156
xyz, charge: 0, multiplicity: 1

C	-5.375335	-3.475478	-0.996553
C	-5.882760	-4.611773	-0.448515
C	-4.251278	-3.699687	-1.838252
C	-3.942822	-5.033578	-1.948754
S	-4.984537	-5.987966	-0.970742
H	-5.761966	-2.489386	-0.790944
C	-7.022666	-4.701561	0.453727
C	-8.171178	-3.945117	0.242277
C	-9.230238	-3.977309	1.121778
C	-9.213653	-4.783848	2.254066
C	-8.080368	-5.556667	2.464280
C	-7.015998	-5.509775	1.580177
F	-8.273497	-3.167560	-0.835132
F	-10.292822	-3.229530	0.853722
C	-10.417056	-4.772843	3.162606
F	-7.963984	-6.357235	3.510954
F	-5.951313	-6.259940	1.838634
C	-3.619433	-2.572123	-2.501120
C	-2.895504	-5.696700	-2.773603
H	-2.654266	-5.081893	-3.639708
H	-3.225124	-6.672774	-3.129769
H	-1.967288	-5.844343	-2.218047
H	-2.361717	-4.197430	0.332525
S	0.553481	-4.410914	-1.030274
C	3.915208	-4.379401	-4.901215
C	3.783288	-6.377254	-3.631515
C	4.451105	-5.623522	-4.586594
C	2.769983	-3.907417	-4.294783
C	-0.126901	-3.435349	-3.279445
C	-1.159859	-3.075798	-2.371411
C	2.086408	-4.660677	-3.342962
C	-0.909143	-3.518435	-1.097116
C	2.630358	-5.900183	-3.032941
C	-2.327125	-2.299727	-2.768561
C	-1.720256	-3.335317	0.137583
C	0.871699	-4.164482	-2.708824
H	-0.148601	-3.170584	-4.325674
H	-2.375247	-2.471832	0.031612
F	4.512952	-3.609981	-5.799226
F	2.330934	-2.703055	-4.633369
F	4.206485	-7.576099	-3.262892
C	5.706554	-6.066190	-5.294797
F	2.028042	-6.672554	-2.134405
H	-1.090086	-3.185833	1.014356
N	-2.198880	-1.119048	-3.525578
O	-4.481714	-1.629489	-2.944244
C	-0.941409	-0.505246	-3.701724
C	-0.469729	-0.256731	-4.986887
C	0.727770	0.400470	-5.183271
C	1.482887	0.817865	-4.092958
C	1.032341	0.552428	-2.809741
C	-0.172718	-0.104074	-2.617062
H	-0.525351	-0.289920	-1.610691
H	-1.061578	-0.572119	-5.835800
H	1.068578	0.590680	-6.192962
H	1.601912	0.869471	-1.947498
C	2.768014	1.541523	-4.342272
F	3.667649	0.754291	-4.944316
F	3.326082	1.995916	-3.219749
F	2.584540	2.595783	-5.151753
H	-0.821473	2.673331	-1.558493
C	-6.869536	-0.142465	0.166868
C	-6.089005	-0.598617	1.217903
C	-4.105274	-0.076088	-0.035625
C	-4.701197	-0.556002	1.114234
C	-6.264207	0.349135	-0.980849
C	-4.883007	0.376775	-1.098114
C	-3.633815	2.086667	-2.274853
C	-6.703091	-1.163592	2.461277
N	-4.278762	0.862029	-2.274905
F	-6.372022	-2.455411	2.618188
F	-8.036780	-1.102485	2.442497
F	-6.285390	-0.524784	3.554940
H	-3.026466	-0.032629	-0.114255
H	-6.869135	0.731634	-1.792530
H	-4.078841	-0.901996	1.929331
H	-7.948337	-0.156016	0.232137
C	-1.152011	3.500592	-0.928216
H	-0.350005	4.234334	-0.885074
C	-3.551343	3.051908	-1.320276
C	-2.453667	4.107518	-1.467374
H	-3.679853	6.309388	-2.893846
C	-4.242447	3.202638	-0.080888
S	-2.951582	5.466041	-0.332765
S	-0.855784	5.576948	-3.266633
C	2.695023	6.088086	-6.099907
C	-2.191045	3.346867	-3.801393
C	-2.358867	4.566763	-2.922631
C	-0.520769	4.661706	-4.739725
C	1.700351	5.678279	-5.229301
C	1.363559	5.362315	-7.918889
C	0.375501	4.963090	-7.046418
C	0.514283	5.100792	-5.665937
C	-2.896441	2.232957	-3.522391
C	-1.284847	3.556135	-4.878277
C	-3.581460	5.354148	-3.405240
C	2.549882	5.933410	-7.471134
O	-2.983071	1.178075	-4.321598
C	-4.018433	4.378040	0.549237
H	-4.936133	2.474749	0.307006
F	-0.738038	4.451068	-7.561711
H	-4.495330	4.781967	-3.238468
C	3.581615	6.342517	-8.489151
F	1.156106	5.201965	-9.220657
F	3.785388	6.618195	-5.568360
F	1.912660	5.839433	-3.929738
H	-1.171422	2.843632	-5.681040
H	-1.322236	3.121653	0.079310
C	-4.571484	4.761400	1.841107
C	-4.720610	3.845609	2.882861
C	-4.959368	6.067056	2.119154
F	-4.339326	2.582320	2.727060
F	-4.851964	6.995412	1.176300
C	-5.238481	4.215648	4.104994
C	-5.474913	6.447590	3.345773
H	-3.486571	5.546270	-4.473247
C	-5.631533	5.523461	4.369436
F	-5.344649	3.290062	5.049417
F	-5.820050	7.717601	3.491325
C	-6.190311	5.846224	5.730740
Si	-3.724412	-0.207986	-3.599446
F	-11.504862	-5.176731	2.502030
F	-10.653820	-3.542423	3.616477
F	-10.276039	-5.566055	4.216257
F	5.508742	-6.112909	-6.613623
F	6.117392	-7.268919	-4.913085
F	6.703723	-5.211987	-5.064673
F	4.673690	6.860422	-7.941419
F	3.084126	7.257259	-9.324889
F	3.963272	5.294487	-9.222502
F	-5.294112	5.584433	6.683995
F	-6.541904	7.119855	5.857042
F	-7.273588	5.108249	5.982675
O	-4.817466	-0.272277	-4.977746
P	-5.962431	-1.186183	-5.507496
C	-6.885220	-0.117875	-6.652921
H	-7.561337	0.493542	-6.049731
H	-6.142964	0.571042	-7.062479
C	-7.635732	-0.814383	-7.784319
H	-8.386495	-1.517025	-7.428760
H	-6.960208	-1.351302	-8.448584
H	-8.151152	-0.069431	-8.388206
C	-5.293641	-2.573573	-6.457406
H	-6.144620	-3.151800	-6.827890
H	-4.805510	-2.144478	-7.336688
C	-4.322769	-3.465364	-5.691076
H	-4.780477	-3.918763	-4.813218
H	-3.441501	-2.915331	-5.366245
H	-3.988425	-4.272445	-6.341143
C	-7.088611	-1.806571	-4.226990
H	-6.571990	-2.589251	-3.674363
H	-7.232959	-0.990719	-3.515372
C	-8.429540	-2.308380	-4.752786
H	-8.320279	-3.110857	-5.482593
H	-9.028644	-1.517132	-5.201045
H	-9.000887	-2.713539	-3.919191

2-cc

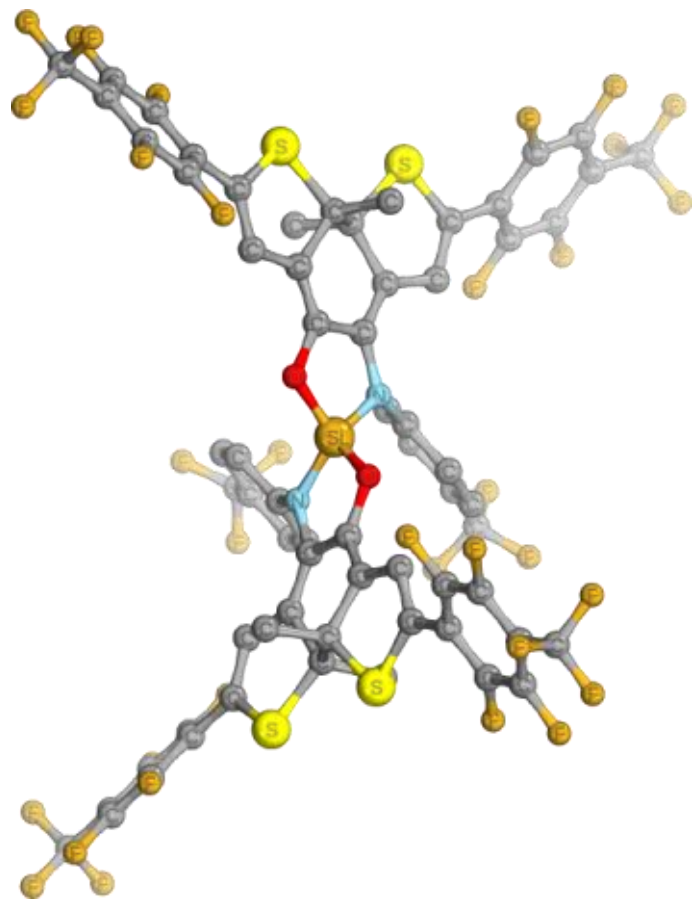

133
xyz, charge: 0, multiplicity: 1

C	1.149457	5.280873	-0.651597
C	0.689872	4.011497	-0.627335
C	2.563684	5.371061	-0.481156
C	3.257861	4.040460	-0.694181
S	1.940046	2.795259	-0.378979
H	0.514558	6.149632	-0.741698
C	-0.700907	3.600824	-0.801219
C	-1.532011	4.206022	-1.740716
C	-2.841889	3.808541	-1.906738
C	-3.395851	2.782357	-1.149136
C	-2.573634	2.166622	-0.216153
C	-1.260047	2.573007	-0.053837
F	-1.068725	5.176931	-2.517013
F	-3.574049	4.425269	-2.822919
C	-4.837977	2.409147	-1.389948
F	-3.003653	1.183014	0.556461
F	-0.524707	1.956372	0.862242
C	3.321983	6.450570	-0.237091
C	3.655680	3.945014	-2.173028
H	4.413853	4.689677	-2.418701
H	2.782351	4.129726	-2.796988
H	4.050189	2.962594	-2.421579
H	3.472982	2.957431	1.971782
S	5.507520	2.479523	-0.088935
C	10.497667	2.657690	1.042991
C	9.832853	1.114482	-0.631408
C	10.815399	1.570463	0.236009
C	9.261381	3.265301	0.985973
C	6.728972	4.787075	0.120816
C	5.348370	5.143758	0.079811
C	8.271518	2.822201	0.111309

C	4.439456	3.932442	0.269904
C	8.594457	1.731423	-0.684792
C	4.757914	6.358193	-0.021468
C	4.036732	3.860621	1.748103
C	6.958199	3.457381	0.055701
H	7.528803	5.510764	0.139304
H	3.425706	4.719710	2.027894
F	11.389779	3.128073	1.902242
F	9.025220	4.281398	1.806722
F	10.034583	0.089351	-1.442341
C	12.191609	0.963529	0.357220
F	7.698599	1.260088	-1.542091
H	4.933800	3.861521	2.366453
N	5.317419	7.638119	0.024508
O	2.838176	7.725452	-0.219951
C	6.548042	7.963182	0.629484
C	7.344799	8.938109	0.044340
C	8.501913	9.365605	0.676568
C	8.881286	8.805550	1.885810
C	8.091601	7.816097	2.463159
C	6.928794	7.402762	1.846370
H	6.306563	6.656255	2.320836
H	7.067565	9.357495	-0.914854
H	9.106321	10.127738	0.204621
H	8.363765	7.376353	3.412728
C	10.097105	9.294666	2.610286
F	10.755267	8.298904	3.205535
F	9.764449	10.171098	3.570773
F	10.953121	9.915846	1.796542
H	6.161743	12.988310	-0.743180
C	4.450820	8.473621	4.348196
C	5.591828	9.095250	4.831057
C	5.704881	10.431053	2.835213
C	6.210836	10.081772	4.070890
C	3.928641	8.836478	3.117171
C	4.558126	9.808145	2.349935
C	4.043999	11.319877	0.437377
C	6.199540	8.663296	6.129633
N	4.075967	10.064523	1.050853
F	7.141151	7.727292	5.930970
F	5.295082	8.133345	6.955898
F	6.786506	9.675832	6.768354
H	6.214190	11.175886	2.239049
H	3.020550	8.369046	2.757048
H	7.108366	10.569796	4.424227
H	3.946902	7.712604	4.928013
C	5.788884	13.857317	-0.199616
H	6.038098	14.747457	-0.773324
C	4.038720	12.558668	0.984313
C	4.280101	13.741685	0.050588
H	1.611246	14.587599	-0.654875
C	3.920532	12.968175	2.345653
S	3.759294	15.218224	1.014465
S	3.856823	14.757753	-2.547563
C	4.468038	15.222102	-7.043128
C	3.782600	12.205086	-1.831666
C	3.449973	13.551080	-1.219003
C	3.784856	13.486536	-3.765287
C	4.506665	14.872488	-5.703992
C	2.875076	13.504583	-7.415364
C	2.917460	13.163861	-6.079912
C	3.742533	13.837268	-5.182440
C	3.984657	11.165407	-1.008199
C	3.758481	12.235335	-3.258455
C	1.935019	13.601975	-0.980864
C	3.648973	14.538709	-7.930680
O	4.099231	9.875204	-1.434651
C	3.783994	14.301876	2.511563
H	3.879939	12.276212	3.172292
F	2.132447	12.180752	-5.658960
H	1.637823	12.876669	-0.222379
C	3.546947	14.847181	-9.404259
F	2.063292	12.824331	-8.211648
F	5.245371	16.219068	-7.431937
F	5.317923	15.552793	-4.904479
H	3.766843	11.340478	-3.862704
H	6.309348	13.913687	0.755898

C	3.677083	14.987444	3.796041
C	4.456253	14.616348	4.889840
C	2.813382	16.057484	3.988194
F	5.332573	13.625022	4.783475
F	2.040381	16.461039	2.988571
C	4.359917	15.271511	6.099090
C	2.712928	16.721961	5.198772
H	1.413455	13.360146	-1.905869
C	3.486130	16.337416	6.285043
F	5.135139	14.868797	7.095235
F	1.849764	17.721388	5.271569
C	3.438714	16.999756	7.640072
Si	4.076096	8.825556	-0.152131
F	-5.015380	2.015883	-2.651350
F	-5.633804	3.457285	-1.174755
F	-5.254111	1.425512	-0.603631
F	13.128676	1.873416	0.088826
F	12.379561	-0.056882	-0.468925
F	12.399453	0.518789	1.596735
F	4.336800	15.844938	-9.777942
F	2.297750	15.182828	-9.726861
F	3.881430	13.779092	-10.129236
F	4.632666	17.500393	7.958277
F	2.561876	17.993180	7.697835
F	3.104261	16.115752	8.580632

2-cc-F

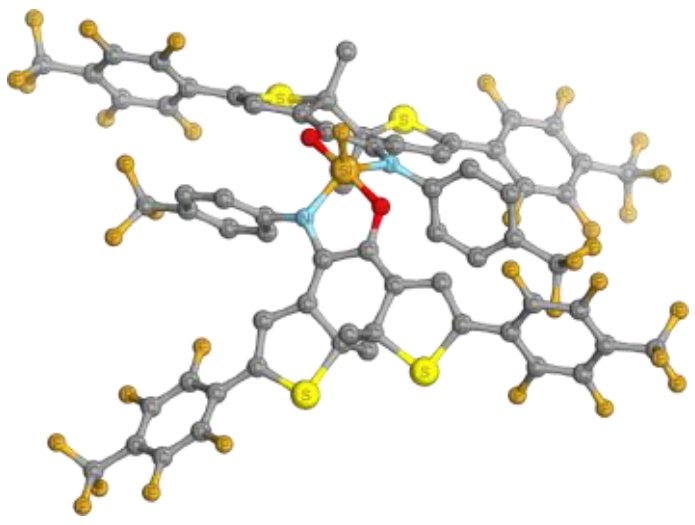

134

xyz, charge: -1, multiplicity: 1

C	-4.447969	-3.576101	-0.666635
C	-4.370430	-4.554702	0.262850
C	-3.256979	-3.406786	-1.421688
C	-2.296019	-4.566489	-1.291818
S	-2.813696	-5.394854	0.275407
H	-5.294309	-2.913933	-0.776274
C	-5.414939	-4.898839	1.214860
C	-6.765021	-4.887437	0.864278
C	-7.754151	-5.179885	1.775873
C	-7.462933	-5.514127	3.094174
C	-6.122318	-5.544448	3.451556
C	-5.133918	-5.243821	2.532054
F	-7.132227	-4.607201	-0.381109
F	-9.018044	-5.154795	1.365980
C	-8.600622	-5.822089	4.027619
F	-5.734908	-5.850714	4.682112
F	-3.873710	-5.277747	2.948419
C	-2.945367	-2.360474	-2.216965
C	-2.542930	-5.538384	-2.449353
H	-2.264558	-5.077814	-3.398456
H	-3.602381	-5.788725	-2.488309
H	-1.975789	-6.460389	-2.338074
H	-0.577294	-3.881955	0.923179
S	0.401701	-5.333659	-1.419406
C	5.052738	-3.987297	-3.214148
C	4.497122	-6.288151	-3.181209
C	5.466532	-5.308831	-3.350714
C	3.745883	-3.657961	-2.929885
C	0.713349	-3.199791	-2.903278
C	-0.619664	-3.076020	-2.421636
C	2.756605	-4.632058	-2.771529
C	-0.868760	-4.016381	-1.240328
C	3.185601	-5.950270	-2.902806
C	-1.601070	-2.190072	-2.759440
C	-0.558332	-3.239993	0.045052
C	1.378674	-4.294018	-2.460143
H	1.147601	-2.514518	-3.613159
H	-1.286676	-2.441298	0.193525

F	5.937258	-3.005026	-3.339185
F	3.452745	-2.371467	-2.779548
F	4.781925	-7.578800	-3.292137
C	6.911781	-5.584121	-3.660411
F	2.308512	-6.940433	-2.774875
H	0.433103	-2.794413	-0.033346
N	-1.570466	-1.049006	-3.518985
O	-3.781960	-1.405306	-2.554562
C	-0.351257	-0.509793	-3.982938
C	-0.060794	-0.511521	-5.340826
C	1.100499	0.076785	-5.807316
C	1.986595	0.676739	-4.919573
C	1.708935	0.664374	-3.560129
C	0.546992	0.071740	-3.097437
H	0.326333	0.069778	-2.037413
H	-0.763719	-0.959780	-6.029767
H	1.301907	0.077417	-6.870707
H	2.379895	1.132325	-2.853681
C	3.234674	1.304743	-5.452047
F	4.122595	0.384390	-5.849327
F	3.845928	2.077022	-4.550410
F	2.983194	2.074363	-6.523008
H	0.327292	2.455451	-1.369855
C	-5.784154	-0.128346	0.610621
C	-4.951067	-0.697324	1.567291
C	-3.042046	-0.075783	0.252660
C	-3.575631	-0.660778	1.387633
C	-5.246697	0.452619	-0.523462
C	-3.871661	0.474606	-0.715972
C	-2.588297	2.156801	-1.901030
C	-5.558396	-1.317163	2.784990
N	-3.331638	1.004838	-1.907519
F	-4.691455	-2.074448	3.461798
F	-6.603926	-2.099477	2.473108
F	-6.020373	-0.391476	3.635275
H	-1.969067	-0.055267	0.108818
H	-5.891812	0.876325	-1.281106
H	-2.910238	-1.104340	2.114872
H	-6.859067	-0.148010	0.735160
C	0.114910	3.264025	-0.668801
H	0.983730	3.918011	-0.633981
C	-2.337502	3.062977	-0.912057
C	-1.153831	4.016711	-1.087335
H	-2.196483	6.428181	-2.312249
C	-2.921759	3.201340	0.377640
S	-1.445596	5.350471	0.144248
S	0.485598	5.385914	-2.936587
C	3.894534	5.525490	-6.006481
C	-1.149251	3.368793	-3.473242
C	-1.105501	4.544040	-2.523309
C	0.596468	4.525216	-4.477878
C	2.909042	5.225928	-5.083622
C	2.341668	5.136068	-7.750415
C	1.364029	4.844062	-6.826268
C	1.617860	4.868769	-5.455079
C	-1.951276	2.315864	-3.204595
C	-0.312144	3.533016	-4.609100
C	-2.253173	5.496567	-2.871648
C	3.633709	5.481720	-7.368797
O	-2.215145	1.344689	-4.049397
C	-2.546120	4.312877	1.055890
H	-3.654337	2.513176	0.767182
F	0.149884	4.551156	-7.278301
H	-3.214817	5.027358	-2.658573
C	4.643207	5.790000	-8.439590
F	2.022676	5.099637	-9.040055
F	5.092116	5.844501	-5.535191
F	3.233890	5.272931	-3.797257
H	-0.351241	2.858958	-5.452319
H	-0.031058	2.834140	0.321904
C	-2.970733	4.670762	2.398100
C	-3.201573	3.712062	3.388262
C	-3.148149	5.994613	2.791879
F	-3.017160	2.422255	3.131331
F	-2.957461	6.971369	1.911130
C	-3.595358	4.060547	4.661331
C	-3.536367	6.351724	4.069946

H -2.215044 5.730729 -3.934719
C -3.776234 5.387412 5.039461
F -3.785876 3.092026 5.549248
F -3.682084 7.645852 4.321550
C -4.207179 5.683890 6.448994
Si -3.057555 -0.030895 -3.364765
F -9.305615 -6.871026 3.590892
F -9.441089 -4.788428 4.116377
F -8.199500 -6.108421 5.261628
F 7.278623 -4.990650 -4.799303
F 7.177735 -6.879915 -3.791323
F 7.705905 -5.115449 -2.693488
F 5.847076 6.074409 -7.954527
F 4.256190 6.840484 -9.171074
F 4.788559 4.757638 -9.273695
F -3.310734 5.229378 7.329244
F -4.360933 6.983287 6.683053
F -5.372585 5.093567 6.726994
F -4.161452 -0.046786 -4.549652

2-cc-Et₃PO

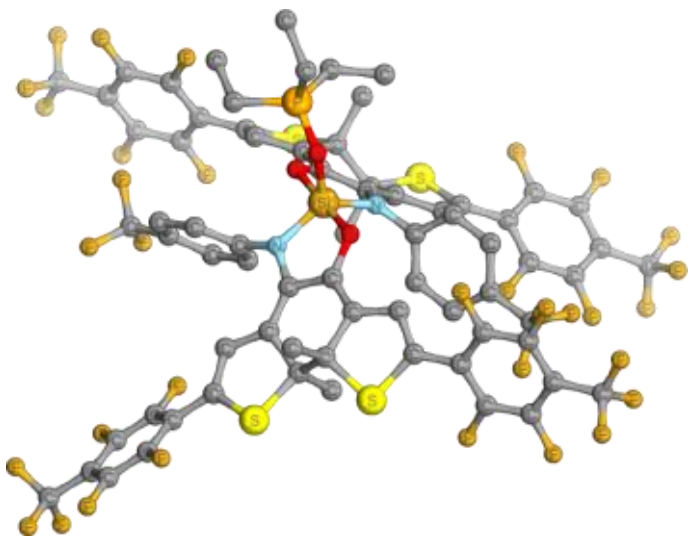

156
xyz, charge: 0, multiplicity: 1
C -5.315394 -2.463578 -0.020680
C -5.511374 -3.324991 0.998672
C -4.096440 -2.683698 -0.724024
C -3.456414 -4.018013 -0.412365
S -4.236489 -4.525753 1.183050
H -5.959464 -1.617090 -0.212846
C -6.637057 -3.276907 1.925708
C -7.939852 -3.079039 1.479024
C -8.997239 -2.985020 2.356041
C -8.816228 -3.091475 3.730688
C -7.522466 -3.301470 4.185940
C -6.464231 -3.391508 3.297305
C -3.494443 -1.859999 -1.601156
C -3.883523 -5.023437 -1.487230
H -4.970263 -5.023995 -1.570040
H -3.561300 -6.035960 -1.254265
H -3.458079 -4.748307 -2.454539
H -1.727272 -3.503286 1.837076
S -1.026793 -5.414917 -0.262375
C 2.765558 -7.529363 -1.665323
C 3.895697 -5.447117 -1.745363
C 3.961763 -6.835851 -1.784451
C 1.566507 -6.853253 -1.523287
C -0.124524 -3.609103 -1.930016
C -1.414692 -3.115117 -1.568629
C 1.488397 -5.466464 -1.490315
C -1.941149 -3.821881 -0.316478
C 2.697698 -4.780976 -1.598609
C -2.139398 -2.085024 -2.078189
C -1.508981 -2.994640 0.900625
C 0.218519 -4.769313 -1.328333
H 0.508871 -3.130951 -2.659821
H -2.020204 -2.030728 0.910734
H -0.435822 -2.813743 0.853024
N -1.816529 -1.103077 -2.996167
O -4.068619 -0.776826 -2.117543
C -0.490398 -0.933172 -3.462591
C 0.481037 -0.383006 -2.638272
C 1.756211 -0.140260 -3.120512
C 2.070939 -0.451157 -4.435243
C 1.108066 -1.026632 -5.255789
C -0.164574 -1.264766 -4.770354
H -0.920425 -1.692517 -5.415330
H 0.233259 -0.128359 -1.615399
H 2.490239 0.311192 -2.468057
H 1.338960 -1.278634 -6.282570
C 3.439221 -0.197438 -4.989301
F 4.163022 0.605914 -4.209122
F 3.376264 0.375469 -6.200521
F 4.125497 -1.334868 -5.138705
H 0.823901 2.293034 -1.514088
C -5.763309 1.706936 0.677596
C -5.220928 0.922788 1.690027
C -3.207838 0.680101 0.407024
C -3.932950 0.425153 1.558879
C -5.037810 1.950693 -0.474847
C -3.766048 1.411973 -0.631252
C -2.107680 2.522306 -2.046754
C -6.066998 0.605412 2.883716
N -3.082688 1.557626 -1.858349
F -7.142738 -0.118542 2.526329
F -6.531132 1.710632 3.471610
F -5.411557 -0.098854 3.805560
H -2.205756 0.284072 0.300261
H -5.455102 2.560173 -1.266391
H -3.490467 -0.178221 2.339042
H -6.756601 2.125500 0.778526
C 0.793355 3.242576 -0.977728
H 1.776799 3.701808 -1.047221
C -1.648841 3.499371 -1.222528
C -0.303629 4.148187 -1.553000
H -2.144557 5.243601 -3.331014
C -2.141137 3.964073 0.034040
S -0.296975 5.713692 -0.590683
S 1.547044 4.796301 -3.582456
C 3.350201 3.185553 -8.148100
C -0.467607 3.079600 -3.776665
C -0.173185 4.379433 -3.060398
C 1.484441 3.630149 -4.904659
C 2.327801 3.310272 -7.224555
C 4.927246 3.556173 -6.419560
C 3.903933 3.674000 -5.504486
C 2.568646 3.546574 -5.875921
C -1.470675 2.298009 -3.335550
C 0.390509 2.839124 -4.886141
C -1.111262 5.460423 -3.606001
C 4.678459 3.303911 -7.763563
O -1.945962 1.244029 -3.994596
C -1.531723 5.079742 0.495594
H -2.963520 3.502379 0.555698
H -1.042246 5.482397 -4.692792
H 0.220824 2.030571 -5.581602
H 0.583804 3.040434 0.072207
C -1.816656 5.738256 1.763379
C -2.142284 5.020998 2.914816
C -1.759038 7.120006 1.904947
C -2.405314 5.651019 4.111764
C -2.015979 7.759706 3.104904
H -0.859458 6.449164 -3.228175
C -2.351881 7.034932 4.239727
Si -2.987737 0.245750 -3.059976
O -4.157432 0.230599 -4.357732
P -5.527031 -0.521950 -4.483633
C -6.076713 -0.093565 -6.156384
H -6.029096 0.996096 -6.223636
H -5.321234 -0.470123 -6.849754
C -7.464764 -0.598442 -6.532856
H -7.698318 -0.301024 -7.553560
H -8.242253 -0.182116 -5.893207
H -7.538677 -1.684882 -6.492505
C -5.388240 -2.325948 -4.398341
H -5.320471 -2.635001 -3.355064
H -6.333633 -2.725141 -4.776287
C -4.213031 -2.876127 -5.200959
H -4.274593 -2.622090 -6.258996
H -4.204521 -3.962197 -5.127282
H -3.261394 -2.514881 -4.816813
C -6.768016 0.089368 -3.304896
H -6.233329 0.409390 -2.410877
H -7.212777 0.984247 -3.747788
C -7.842436 -0.927663 -2.926615
H -7.419036 -1.787884 -2.411408
H -8.407209 -1.289319 -3.784893
H -8.553377 -0.465291 -2.244089
F 3.004501 2.962497 -9.408138
F 6.174798 3.678262 -5.988257
F 4.216712 3.899470 -4.235506
F 1.080704 3.206106 -7.672333
F 0.453678 -7.572015 -1.429598
F 2.716495 -8.851847 -1.693721
F 5.006275 -4.729590 -1.837291
F 2.725203 -3.455091 -1.540056
F -8.194459 -2.985625 0.175801
F -10.213265 -2.797639 1.858832
F -7.243820 -3.414465 5.473816
F -5.247445 -3.575272 3.790441
F -1.460834 7.872830 0.852672
F -1.940297 9.081361 3.115843
F -2.703396 4.900945 5.164366
F -2.186758 3.693432 2.889974
C -2.655265 7.646631 5.582938
F -1.798844 7.206904 6.506456
F -2.584101 8.971861 5.575382
F -3.884913 7.316710 5.983355
C -10.024809 -2.976631 4.625161
F -9.726763 -3.079583 5.913153
F -10.630362 -1.801535 4.447407
F -10.909914 -3.934377 4.341995
C 5.311108 -7.487782 -1.950000
F 5.887904 -7.091122 -3.085445
F 6.121901 -7.153506 -0.945304
F 5.243548 -8.812796 -1.981359
C 5.850997 3.179189 -8.701648
F 6.661405 2.192380 -8.316663
F 5.487388 2.926027 -9.953475
F 6.565319 4.306280 -8.715654

Et₃PO

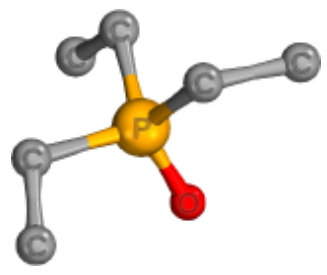

23
xyz, charge: 0, multiplicity: 1
O -3.050560 3.954196 1.769030
P -2.998409 4.237848 0.302955
C -4.529004 3.783693 -0.589249
C -5.774328 4.329952 0.100464
H -4.574321 2.692327 -0.626136
H -4.463892 4.129299 -1.624624
H -5.811722 4.010144 1.140368
H -6.677771 3.977138 -0.395944
H -5.801871 5.419850 0.087994
C -1.661899 3.346968 -0.571724
C -1.649033 1.860873 -0.230697
H -0.714944 3.803898 -0.273410
H -1.760923 3.506961 -1.648880
H -1.586662 1.712031 0.845888
H -0.795241 1.365450 -0.691900
H -2.547664 1.354183 -0.583562
C -2.727246 6.002058 -0.095942
C -1.559173 6.590772 0.687609
H -3.647048 6.538143 0.151193
H -2.579808 6.113957 -1.173635
H -1.697642 6.442748 1.757183
H -1.468687 7.660546 0.501839
H -0.609902 6.131896 0.409919

Pyridine

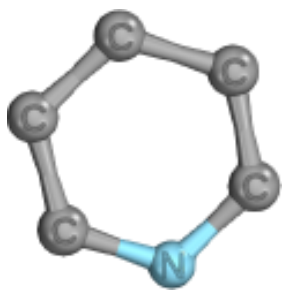

11
xyz, charge: 0, multiplicity: 1
C -9.216104 -6.451536 -7.132276
C -9.216104 -5.260634 -6.424159
C -9.216104 -5.318171 -5.037829
N -9.216104 -6.451536 -4.351727
C -9.216104 -7.584901 -5.037829
C -9.216104 -7.642438 -6.424159
H -9.216104 -6.451536 -8.214450
H -9.216104 -4.305182 -6.930685
H -9.216104 -4.404526 -4.453325
H -9.216104 -8.498546 -4.453325
H -9.216104 -8.597890 -6.930685

BCF

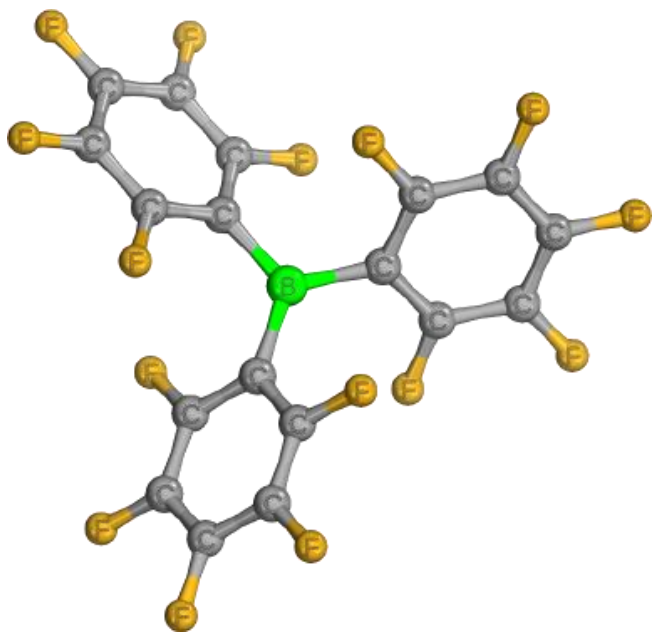

34
xyz, charge: 0, multiplicity: 1
C -1.737805 0.884161 1.560860
C -1.856125 -0.448565 1.218496
C -0.717068 1.633974 1.000570
C 0.177988 1.049035 0.119783
C 0.042714 -0.291740 -0.181388
C -0.973302 -1.074903 0.349335
F -2.582111 1.447289 2.411974
F -2.834018 -1.150506 1.782651
F -0.596201 2.912185 1.307572
F 1.145806 1.775615 -0.418555
F 0.904653 -0.826956 -1.040452
C 1.157293 -3.366398 0.907096
C 0.319284 -4.477721 -1.004978
C 2.420845 -5.126388 -0.085772
C 1.439749 -5.283320 -1.050692
C 0.144506 -3.496912 -0.035764
C 2.281592 -4.167925 0.903762
F 3.493393 -5.894326 -0.109593
F 3.219875 -4.031858 1.828254
F 1.587291 -6.197310 -1.997553
F 1.043265 -2.467742 1.878714
F -0.596680 -4.637709 -1.953609
C -3.434704 -2.470730 -1.112555
C -2.933181 -4.404780 0.152465
C -5.061647 -4.199826 -0.901642
C -4.185549 -4.923884 -0.109816
C -2.522703 -3.168970 -0.331402
C -4.687179 -2.968785 -1.413526
F -6.259193 -4.686077 -1.168899
F -5.526307 -2.287794 -2.179175
F -4.557056 -6.095888 0.382792
F -3.095167 -1.294569 -1.629908
F -2.118743 -5.111584 0.929091
B -1.115996 -2.584733 -0.008127

BCF-F

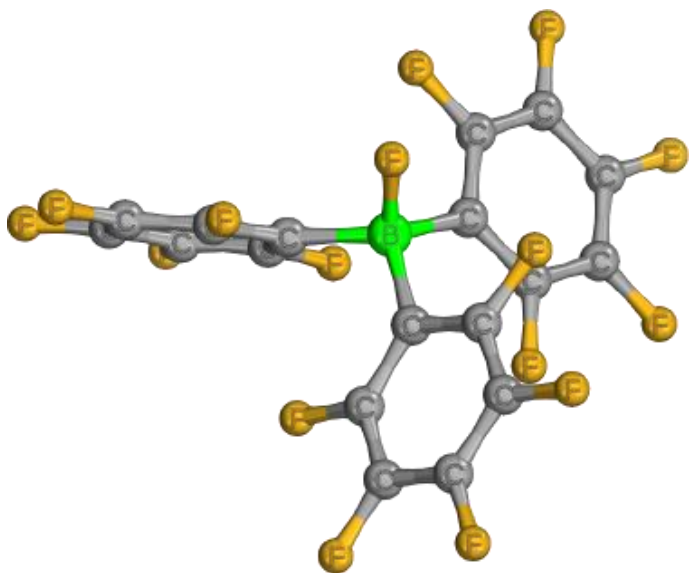

35
xyz, charge: -1, multiplicity: 1
C -1.708764 1.101208 1.531455
C -1.837102 -0.275751 1.496900
C -0.703415 1.706894 0.802749
C 0.143851 0.926503 0.044325
C -0.019903 -0.449929 0.038517
C -0.992699 -1.104276 0.771933
F -2.541702 1.854272 2.253463
F -2.855723 -0.789542 2.187998
F -0.561210 3.032521 0.825146
F 1.110574 1.506148 -0.671259
F 0.823527 -1.129443 -0.746518
C 1.319521 -3.506727 1.154677
C 0.261642 -4.307280 -0.768577
C 2.503851 -4.981700 -0.300627
C 1.393428 -5.027136 -1.116840
C 0.169858 -3.541065 0.378881
C 2.470797 -4.207264 0.842964
F 3.600658 -5.670173 -0.617460
F 3.550902 -4.142374 1.624849
F 1.423271 -5.763478 -2.229924
F 1.371487 -2.745661 2.248745
F -0.757271 -4.380413 -1.632298
C -3.251313 -2.573499 -0.816600
C -3.027776 -4.528760 0.443141
C -4.884735 -4.301492 -1.039008
C -4.182089 -5.048981 -0.113806
C -2.526347 -3.273770 0.129745
C -4.412368 -3.056635 -1.399441
F -6.001722 -4.783854 -1.584605
F -5.077473 -2.335769 -2.305380
F -4.622405 -6.263803 0.221914
F -2.843857 -1.373426 -1.244172
F -2.375694 -5.323610 1.293019
B -1.172432 -2.738138 0.901375
F -1.326326 -3.029170 2.273508

Si(^{F}am^{F}ph)$_2$

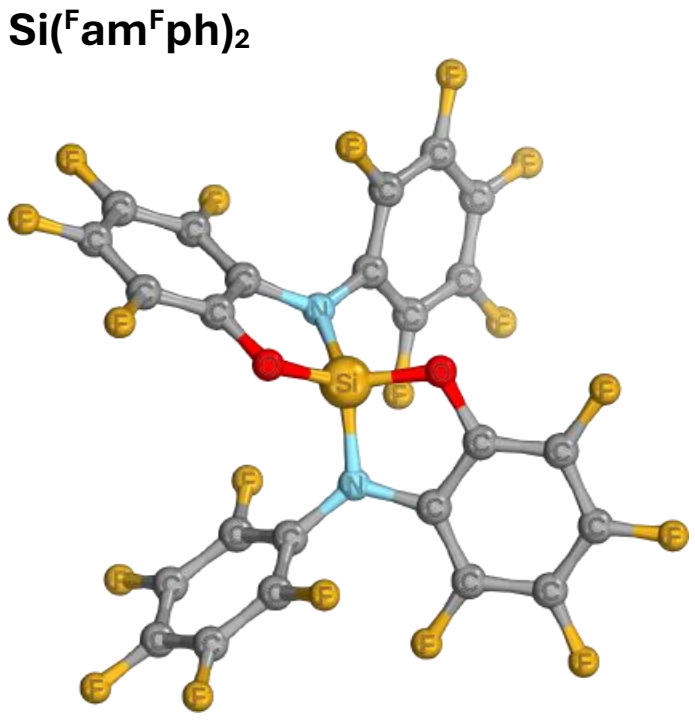

47
xyz, charge: 0, multiplicity: 1
C 0.491700 -2.025560 0.984259
C -0.071705 -3.627374 -0.685259
C 2.051781 -3.742440 0.406984
C 1.148445 -4.250059 -0.512756
C -0.428652 -2.518798 0.071661
C 1.728427 -2.621992 1.150845
F 3.227332 -4.324091 0.566754
F 2.595826 -2.130193 2.020992
F 1.468401 -5.311172 -1.235073
F 0.193112 -0.941344 1.687185
F -0.920490 -4.089129 -1.591250
N -1.651326 -1.865882 -0.124212
C -2.895445 -2.501945 -0.096899
C -3.879444 -1.715758 -0.701203
C -5.185543 -2.143385 -0.777860
C -5.518909 -3.381404 -0.243795
C -4.552264 -4.162474 0.363357
C -3.239767 -3.720501 0.449919
F -6.114973 -1.392857 -1.348737
F -6.770759 -3.812166 -0.309795
F -4.882346 -5.338812 0.876829
F -2.332161 -4.477746 1.059531
O -3.449367 -0.513469 -1.167758
F -2.516126 0.227327 2.247695
F -4.333269 1.552439 3.747812
C -2.948072 1.420283 1.856470
C -3.889572 2.085188 2.620577
F -0.105835 3.676573 0.710275
C 0.277081 2.944904 -0.331683
F 2.064038 4.416121 -0.711967
C 1.397012 3.324518 -1.057712
N -1.556138 1.241623 -0.113310
C -0.421033 1.813425 -0.701077
C 1.832364 2.565141 -2.128984
C 0.038901 1.041304 -1.769955
C 1.154982 1.407654 -2.487490
F 2.907274 2.937262 -2.808885
F 1.583480 0.668950 -3.499347
O -0.665165 -0.095145 -2.003024
C -2.472310 1.954488 0.667946
C -4.360903 3.317407 2.203908
F -5.252929 3.965948 2.932076
C -2.988628 3.174917 0.250470
C -3.906805 3.865768 1.016139
F -2.595736 3.682745 -0.909091
F -4.373340 5.033853 0.606382
Si -1.836734 -0.303496 -0.844287

Si(^{F}am^{F}ph)$_2$-F

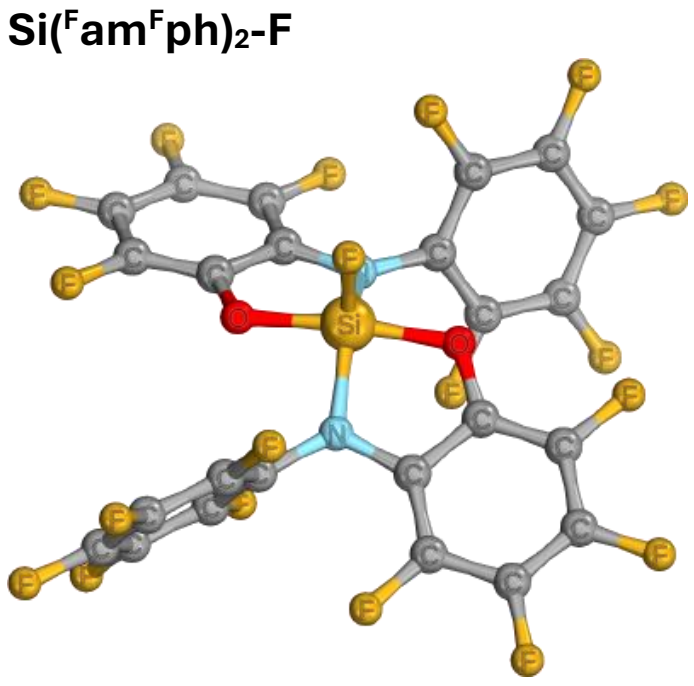

48
xyz, charge: -1, multiplicity: 1
C 0.470171 -2.658182 0.109095
C -0.593890 -3.577590 -1.807459
C 1.612248 -4.281644 -1.222580
C 0.516349 -4.359993 -2.061737
C -0.639420 -2.716555 -0.721208
C 1.592229 -3.427364 -0.135120
F 2.685648 -5.028947 -1.460544
F 2.650406 -3.359473 0.667958
F 0.543075 -5.180235 -3.109141
F 0.465018 -1.843167 1.153325
F -1.636702 -3.653541 -2.621644
N -1.747118 -1.898963 -0.497891
C -2.910151 -2.398805 0.056210
C -3.879516 -1.385368 0.138338
C -5.117573 -1.653253 0.684536
C -5.406969 -2.929520 1.152326
C -4.457315 -3.925595 1.074351
C -3.205949 -3.660742 0.529286
F -6.043661 -0.696909 0.773777
F -6.607161 -3.189851 1.677858
F -4.736440 -5.152917 1.523791
F -2.309134 -4.655250 0.484398
O -3.496029 -0.211893 -0.324239
F -2.261842 0.861324 2.345589
F -4.183496 2.258152 3.634530

C -2.870227 1.857649 1.719280
C -3.855404 2.563835 2.382463
F -0.372899 4.005566 0.560818
C 0.302923 3.066690 -0.115108
F 2.193012 4.474788 -0.077958
C 1.632317 3.315809 -0.436477
N -1.566019 1.401530 -0.265091
C -0.287266 1.875919 -0.485933
C 2.370569 2.374908 -1.121565
C 0.468452 0.917708 -1.182085
C 1.788097 1.169556 -1.495047
F 3.647072 2.622033 -1.427228
F 2.516284 0.263988 -2.150712
O -0.178913 -0.192391 -1.477227
C -2.519187 2.160939 0.412158
C -4.501701 3.603343 1.738935
F -5.447657 4.290092 2.371842
C -3.183627 3.205075 -0.213253
C -4.163761 3.928949 0.438463
F -2.874806 3.516951 -1.463620
F -4.790650 4.928693 -0.176490
Si -1.887182 -0.189628 -1.044211
F -2.419119 -0.051257 -2.574416

SbF_5

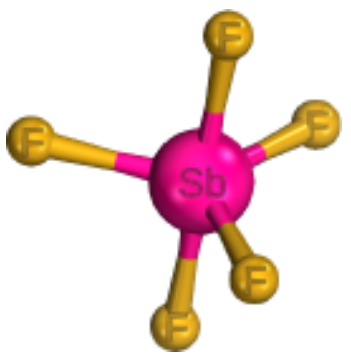

6
xyz, charge: 0, multiplicity: 1
Sb -1.460171 6.151224 -0.508443
F -1.364743 7.011970 -2.149263
F -3.080380 5.418708 0.020404
F -0.997691 4.538325 -1.321815
F 0.036502 6.021821 0.582516
F -1.907347 7.762823 0.316311

SbF_6

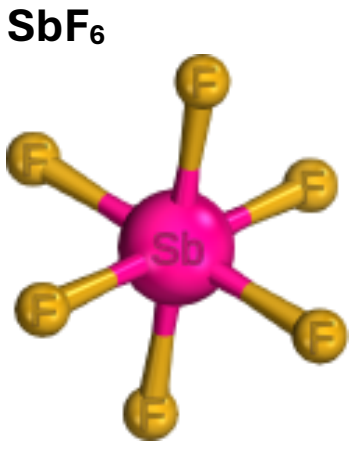

7
xyz, charge: -1, multiplicity: 1
Sb -1.730023 6.175075 -0.706291
F -0.703488 7.123273 -1.992061
F -2.756557 5.226931 0.579521
F -1.268472 4.532481 -1.541009
F -0.201833 6.036539 0.415603
F -2.191627 7.817634 0.128466
F -3.258160 6.313598 -1.828260

TMS

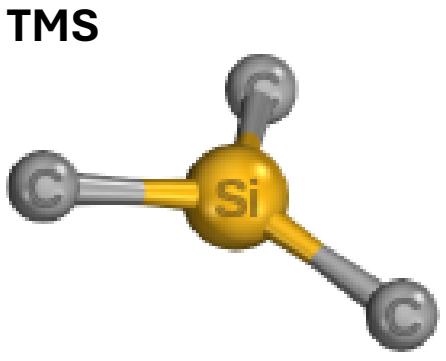

13
xyz, charge: 1, multiplicity: 1
C -2.991743 0.724986 -0.626092
Si -1.284744 0.144071 -0.301590
H -3.116623 1.797099 -0.484882
H -3.701781 0.204147 0.024400
H -3.293288 0.471642 -1.647538
C 0.007757 1.338157 0.208534
H 0.118888 2.124708 -0.544809
H 0.980224 0.877315 0.372523
H -0.291036 1.854712 1.126356
C -0.870203 -1.630753 -0.488037
H -1.717608 -2.241726 -0.793929
H -0.474508 -2.030828 0.451030
H -0.066825 -1.759720 -1.220507

TMSF

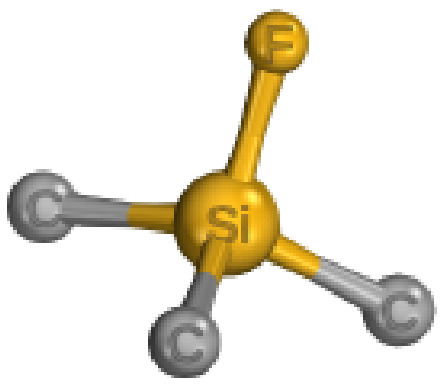

14
xyz, charge: 0, multiplicity: 1
C -2.783810 0.809545 -1.162693
Si -1.494014 0.399954 0.131390
H -3.048038 1.867701 -1.153411
H -3.702554 0.240352 -1.016306
H -2.417157 0.577800 -2.164615
C 0.048334 1.434245 -0.105577
H -0.171131 2.502409 -0.083471
H 0.516535 1.221071 -1.068485
H 0.793084 1.236128 0.666152
C -1.089830 -1.427435 0.125456
H -1.977454 -2.039575 0.289859
H -0.365698 -1.685223 0.899206
H -0.661502 -1.730121 -0.831932
F -2.116715 0.761907 1.592945

1_cc_to_oc_TS

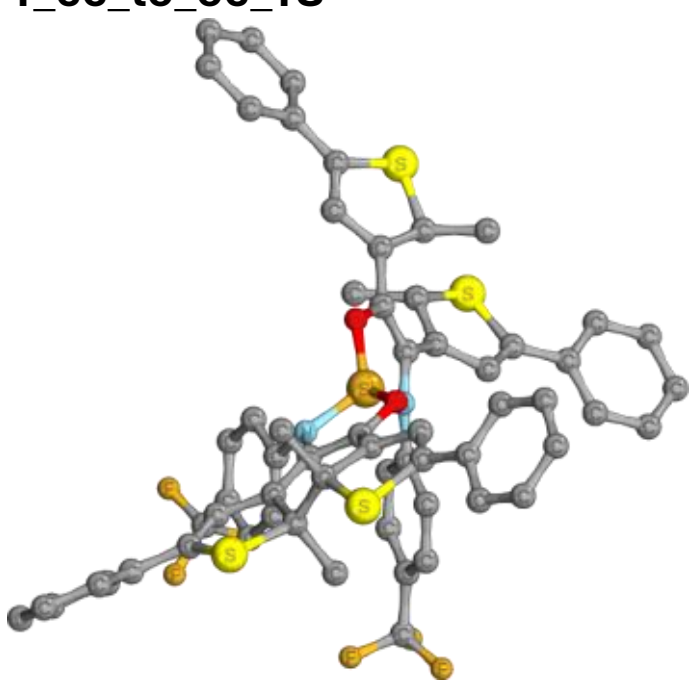

121
xyz, charge: 0, multiplicity: 1
C 0.404324 6.114563 0.370993
C -0.389616 5.005444 0.583663
C 1.676334 5.826487 -0.116821
C 1.929380 4.413290 -0.319290
S 0.449444 3.556780 0.117648
H 0.099945 7.118628 0.635151
C -1.726067 4.962416 1.163448
C -2.254139 3.778624 1.686851
C -3.523678 3.749610 2.236921
C -4.296571 4.900019 2.277318
C -3.786569 6.082087 1.757598
C -2.519933 6.114207 1.205115
H -1.658652 2.872904 1.675058
H -3.910690 2.820908 2.638838
H -5.290150 4.876257 2.707669
H -4.384375 6.985578 1.775514
H -2.145770 7.039259 0.783847
C 2.787473 6.739998 -0.132277
C 2.553241 3.916454 -1.603169
H 2.822589 2.861622 -1.529595
H 3.449114 4.484306 -1.847405
H 1.844272 4.031383 -2.424298
H 3.487760 4.435951 3.173913
S 4.095667 2.512575 1.013959
C 8.719185 1.466906 -1.114419
C 7.092189 -0.277472 -0.888339
C 8.372529 0.127765 -1.232958
C 7.796946 2.390310 -0.658912
C 5.548955 4.344179 -0.047619
C 4.433676 5.015703 0.445563
C 6.498512 1.996839 -0.316737
C 3.443093 4.149541 1.048341
C 6.165055 0.644739 -0.435330
C 4.070205 6.388709 0.157408
C 2.772342 4.512262 2.353090
C 5.528044 2.978441 0.150663
H 6.333419 4.841891 -0.602744
H 1.937848 3.842527 2.566376
H 9.720063 1.792491 -1.371416
H 8.090819 3.427063 -0.547787
H 6.810324 -1.319923 -0.975420
H 9.097673 -0.594589 -1.587017
H 5.163375 0.315556 -0.183591
H 2.392056 5.532125 2.323707
N 4.966916 7.473003 0.027541
O 2.591969 8.081432 -0.390111
C 6.229215 7.546850 0.648470
C 6.493495 6.920124 1.864274
C 7.709662 7.109876 2.489978
C 8.671800 7.931111 1.918551
C 8.418145 8.549523 0.704267
C 7.208062 8.348085 0.065444
H 7.014317 8.815500 -0.893442
H 5.734546 6.310946 2.335666
H 7.898785 6.635163 3.444053
H 9.168501 9.183196 0.249186
C 9.953179 8.203545 2.652525
F 10.959713 8.509673 1.821415
F 10.346975 7.157303 3.389942
F 9.822919 9.245489 3.499091
H 7.456184 14.014362 -1.372783
C 4.163776 8.788822 4.123629
C 5.372276 9.227783 4.636719
C 5.822599 10.401352 2.590536
C 6.198082 10.038582 3.867173
C 3.777400 9.163122 2.847142
C 4.606988 9.961588 2.067965
C 4.582896 11.356258 -0.011570
C 5.832965 8.781507 5.994985
N 4.244655 10.201297 0.722122
F 4.815702 8.390180 6.774427
F 6.486705 9.752189 6.648531
F 6.679837 7.736715 5.909199
H 6.482650 11.005107 1.982296
H 2.827095 8.830201 2.445584
H 7.151719 10.369899 4.258393
H 3.515261 8.161346 4.720983
C 6.953652 13.282673 -0.742409
H 7.409264 13.307431 0.247931
C 4.859926 12.605733 0.408972
C 5.452229 13.584803 -0.610840
H 2.704443 13.693237 -2.767892
C 4.736580 13.201025 1.711429
S 5.285191 15.235196 0.176362
S 5.451395 14.346295 -3.351114
C 4.919393 12.495564 -8.155184
C 4.683869 12.000900 -2.380148
C 4.681369 13.457655 -1.937523
C 5.166750 12.988686 -4.437455
C 4.698667 12.359438 -6.796370
C 6.507279 14.239076 -7.724996
C 6.287136 14.104033 -6.364277
C 5.386429 13.156440 -5.880496
C 4.571881 11.054536 -1.444999
C 4.780708 11.864798 -3.808083
C 3.218099 13.917141 -1.833030
C 5.826383 13.434669 -8.625191
O 4.381530 9.720425 -1.725592
C 4.933915 14.531414 1.746188
H 4.435164 12.643915 2.585880
H 6.833068 14.731236 -5.668653
H 3.150653 14.987033 -1.645919
H 5.995126 13.544081 -9.689560
H 7.215692 14.976656 -8.082232

H	4.371320	11.872397	-8.851610
H	3.969455	11.642163	-6.439662
H	4.626894	10.920687	-4.311853
H	7.103763	12.290956	-1.173546
C	4.853833	15.392265	2.933767
C	4.593839	16.756801	2.817255
C	5.035917	14.853610	4.208944
H	4.450048	17.195402	1.836346
H	5.272799	13.802113	4.319500
C	4.503071	17.557856	3.943593
C	4.941967	15.653995	5.332710
H	2.712983	13.389008	-1.021902
C	4.673950	17.009776	5.204840
H	4.297986	18.615718	3.832810
H	5.088272	15.218676	6.313905
H	4.607292	17.636527	6.085826
Si	4.037018	8.872953	-0.360629

1_oc_to_oo_TS

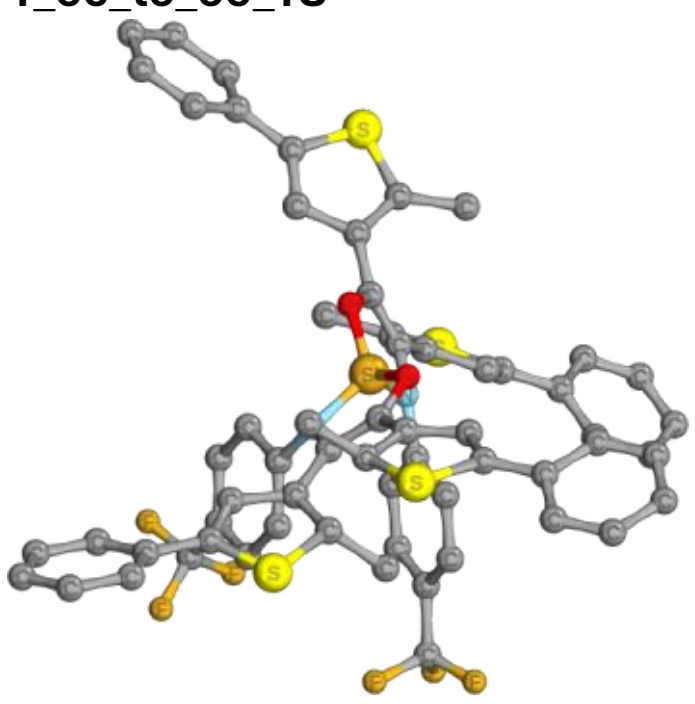

121
xyz, charge: 0, multiplicity: 1

C	0.219430	6.137338	1.003975
C	-0.783523	5.220628	1.024147
C	1.312874	5.770671	0.163463
C	1.109434	4.577361	-0.475398
S	-0.398362	3.903802	-0.015046
H	0.206877	7.045643	1.591744
C	-2.040673	5.263502	1.785652
C	-2.611365	4.103200	2.306295
C	-3.789693	4.159770	3.031978
C	-4.414562	5.377093	3.255031
C	-3.855371	6.537491	2.740693
C	-2.682450	6.481563	2.007052
H	-2.118163	3.149401	2.156126
H	-4.216795	3.247953	3.431867
H	-5.335076	5.420785	3.824300
H	-4.341978	7.492347	2.900011
H	-2.266394	7.387579	1.582620
C	2.505827	6.605401	0.057895
C	1.981122	3.869040	-1.459708
H	2.618518	3.127582	-0.971950
H	2.634178	4.582309	-1.962288
H	1.385003	3.357019	-2.215900
H	3.396178	3.934376	3.473017
S	4.911506	2.663297	1.361737
C	8.867698	2.025729	-1.858597
C	7.572152	0.080013	-1.327203
C	8.699057	0.649588	-1.899924
C	7.917878	2.826830	-1.247995
C	5.371463	4.372391	-0.449510
C	4.369254	4.944396	0.388519
C	6.775503	2.264345	-0.679623
C	4.024040	4.128163	1.430844
C	6.616054	0.880541	-0.724324
C	3.807619	6.275423	0.145399
C	3.053063	4.378265	2.537837
C	5.766469	3.132951	-0.055838
H	5.762594	4.865972	-1.329575
H	2.066147	3.969653	2.307480
H	9.750989	2.477864	-2.293495
H	8.068534	3.898473	-1.189080
H	7.431774	-0.993828	-1.356289
H	9.445265	0.023278	-2.373480
H	5.727095	0.429875	-0.297468
H	2.932117	5.451996	2.687705
N	4.659255	7.383590	-0.111259
O	2.263973	7.957392	-0.166968
C	6.004774	7.497591	0.290063
C	6.506995	6.899312	1.444697
C	7.799634	7.163026	1.853727
C	8.605115	8.029518	1.127330
C	8.117174	8.616340	-0.029256
C	6.830584	8.339470	-0.452628
H	6.451780	8.783644	-1.366358
H	5.876156	6.258289	2.044224
H	8.173403	6.711329	2.763813
H	8.743223	9.282462	-0.608903
C	9.970565	8.386619	1.638297
F	10.793939	8.781559	0.656172
F	10.562255	7.362565	2.267600
F	9.913115	9.402314	2.523963
H	7.328209	13.546006	-1.655175
C	4.329465	8.502270	4.091013
C	5.560550	8.962832	4.529559
C	5.800848	10.253186	2.515351
C	6.287561	9.845215	3.741556
C	3.830166	8.921985	2.871938
C	4.567546	9.785229	2.064256
C	4.402726	11.196139	0.004045
C	6.147542	8.463546	5.817935
N	4.072916	10.062245	0.775865
F	5.214430	7.980775	6.650843
F	6.811365	9.423993	6.476460
F	7.028011	7.465247	5.603962
H	6.396622	10.904125	1.890701
H	2.861077	8.574020	2.531994
H	7.255272	10.198227	4.075084
H	3.751219	7.824039	4.705020
C	6.937854	12.843465	-0.917267
H	7.682269	12.730723	-0.126846
C	4.825876	12.484223	0.515826
C	5.632506	13.333641	-0.334344
H	2.384564	14.080081	-2.210151
C	4.324249	13.124346	1.645228
S	5.754031	14.900659	0.463558
S	5.094186	14.284829	-3.330224
C	6.845296	11.882917	-7.594096
C	4.435665	11.981414	-2.342186
C	4.398641	13.385008	-1.979658
C	5.453018	12.854413	-4.249063
C	6.141998	11.840716	-6.404840
C	7.645937	14.074081	-7.049000
C	6.945116	14.034294	-5.856121
C	6.184213	12.914399	-5.508550
C	4.196736	10.991785	-1.326828
C	5.011014	11.728419	-3.585268
C	3.136822	14.004135	-1.423822
C	7.602872	12.998801	-7.923258
O	3.814319	9.711294	-1.671771
C	4.707411	14.444461	1.771818
H	3.634540	12.648292	2.329976
H	7.000253	14.877347	-5.176890
H	3.325744	15.005937	-1.034482
H	8.152017	13.030147	-8.856254
H	8.233299	14.950666	-7.294878
H	6.796136	11.040478	-8.273714
H	5.536552	10.972632	-6.174297
H	5.171316	10.724461	-3.955494
H	6.807302	11.877295	-1.401883
C	4.290870	15.394882	2.795032
C	4.376627	16.774287	2.587286
C	3.793551	14.940517	4.021568
H	4.752383	17.152761	1.643496
H	3.745156	13.876807	4.220675
C	3.970184	17.665419	3.565250
C	3.385544	15.833552	4.994531
H	2.727793	13.394052	-0.620710
C	3.470321	17.201309	4.772379
H	4.042931	18.730565	3.381068
H	3.006375	15.458990	5.937858
H	3.153165	17.899909	5.536831
Si	3.675406	8.780997	-0.317299

2_cc_to_oc_TS

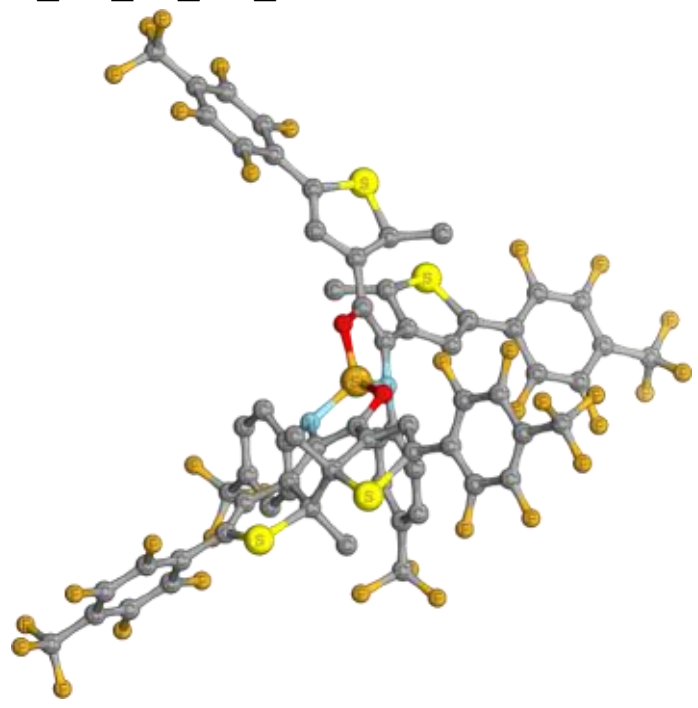

133
xyz, charge: 0, multiplicity: 1

C	1.269714	5.227359	-0.345008
C	0.772096	3.932478	-0.308431
C	2.610394	5.308486	-0.686390
C	3.234359	4.029812	-0.969772
S	2.001547	2.789687	-0.759601
H	0.687402	6.094053	-0.070659
C	-0.576080	3.533676	0.063628
C	-1.670353	4.391882	-0.102029
C	-2.952587	4.024111	0.243324
C	-3.239822	2.772976	0.779125
C	-2.170443	1.904745	0.944075
C	-0.884143	2.280283	0.594523
F	-1.503014	5.602450	-0.632076
F	-3.929971	4.899030	0.042637
C	-4.674535	2.446530	1.132229
F	-2.326941	0.689447	1.447804
F	0.083730	1.388371	0.800915
C	3.442915	6.468221	-0.502675
C	4.070409	3.838775	-2.215514
H	4.813168	4.628282	-2.313231
H	3.428104	3.867032	-3.096425
H	4.584545	2.876571	-2.200376
H	3.136364	3.170014	1.845584
S	5.662118	2.587537	0.425424
C	10.680721	2.989509	-0.884562
C	9.578622	0.948283	-1.350554
C	10.764589	1.666969	-1.307594
C	9.480376	3.565642	-0.529664
C	6.702373	4.832597	-0.288308
C	5.412658	5.146498	0.106376
C	8.271501	2.860294	-0.575085
C	4.625549	4.004569	0.522914
C	8.375168	1.533466	-0.993675
C	4.740228	6.415371	-0.100013
C	3.776617	4.050280	1.773764
C	7.011771	3.482621	-0.199815
H	7.391164	5.566595	-0.679446
H	3.145686	4.937676	1.782489
F	11.778555	3.730427	-0.806093
F	9.507269	4.830261	-0.111496
F	9.540707	-0.317263	-1.740420
C	12.120886	1.109833	-1.681883
F	7.282962	0.775551	-1.080089
H	4.416841	4.076465	2.657035
N	5.331048	7.692590	-0.017248
O	2.929635	7.736171	-0.656509
C	6.477894	7.996620	0.748715
C	7.279795	9.060850	0.348325
C	8.342327	9.467354	1.136059
C	8.625958	8.797531	2.315223
C	7.844686	7.718298	2.703846
C	6.772194	7.321524	1.930243
H	6.142392	6.507038	2.261361
H	7.068203	9.569281	-0.585608
H	8.957134	10.301857	0.824505
H	8.057007	7.200166	3.630059

C 9.730299 9.278775 3.214181
F 10.325568 8.273185 3.866081
F 9.258550 10.125062 4.151963
F 10.679986 9.940544 2.540608
H 6.237748 13.012046 -0.498120
C 3.845665 8.297940 4.075153
C 4.871262 8.940697 4.747622
C 5.193809 10.403248 2.872799
C 5.538868 10.000055 4.146449
C 3.485487 8.712058 2.803254
C 4.165309 9.757627 2.190698
C 3.947343 11.333959 0.257883
C 5.316184 8.449095 6.097050
N 3.873011 10.055704 0.835556
F 6.329646 7.567318 5.982105
F 4.332742 7.821009 6.755281
F 5.753638 9.442470 6.877791
H 5.738081 11.206506 2.393858
H 2.673550 8.222097 2.277925
H 6.348041 10.498084 4.664864
H 3.318445 7.477989 4.545380
C 5.786875 13.875248 -0.004496
H 6.135739 14.779478 -0.499824
C 3.853496 12.556022 0.818038
C 4.253273 13.768106 -0.031049
H 1.767622 14.666399 -1.209656
C 3.501501 12.942265 2.158092
S 3.574315 15.217014 0.870542
S 4.320394 14.824256 -2.663267
C 5.887595 15.242604 -6.987095
C 4.088438 12.263180 -2.011984
C 3.665307 13.610781 -1.445786
C 4.480734 13.572495 -3.890904
C 5.658466 14.897127 -5.663987
C 4.241644 13.671489 -7.653608
C 4.021137 13.331001 -6.333904
C 4.726256 13.936036 -5.295840
C 4.140428 11.211824 -1.190813
C 4.335991 12.320390 -3.427778
C 2.129619 13.677341 -1.482636
C 5.180612 14.631741 -8.012568
O 4.329637 9.919896 -1.613247
C 3.337161 14.264832 2.325546
H 3.344152 12.237242 2.960152
F 3.094150 12.413727 -6.076321
H 1.702819 12.947033 -0.792493
C 5.363109 14.948263 -9.483299
F 3.525887 13.060809 -8.588933
F 6.807353 16.166339 -7.220704
F 6.384139 15.509290 -4.733733
H 4.428351 11.437116 -4.043143
H 6.122226 13.897170 1.032888
C 3.027776 14.919475 3.606096
C 3.679784 14.548804 4.780598
C 2.096858 15.943947 3.716615
F 4.603534 13.591581 4.768566
F 1.418644 16.342305 2.645407
C 3.412656 15.152452 5.984290
C 1.822412 16.571890 4.922081
H 1.786344 13.443686 -2.490356
C 2.479832 16.195487 6.084353
F 4.078332 14.769987 7.059704
F 0.908989 17.530695 4.911527
C 2.245961 16.826911 7.442064
Si 4.109626 8.854119 -0.379566
F -5.453221 2.500219 0.044488
F -5.156655 3.329893 2.015525
F -4.815067 1.235940 1.663805
F 12.668661 1.824472 -2.672838
F 12.067004 -0.155557 -2.086603
F 12.957889 1.162226 -0.638407
F 6.304399 15.860393 -9.703393
F 4.222800 15.411729 -10.008408
F 5.703564 13.847655 -10.164069
F 3.384623 17.333540 7.928231
F 1.353907 17.811738 7.407793
F 1.804046 15.913650 8.314968

2_oc_to_oo_TS

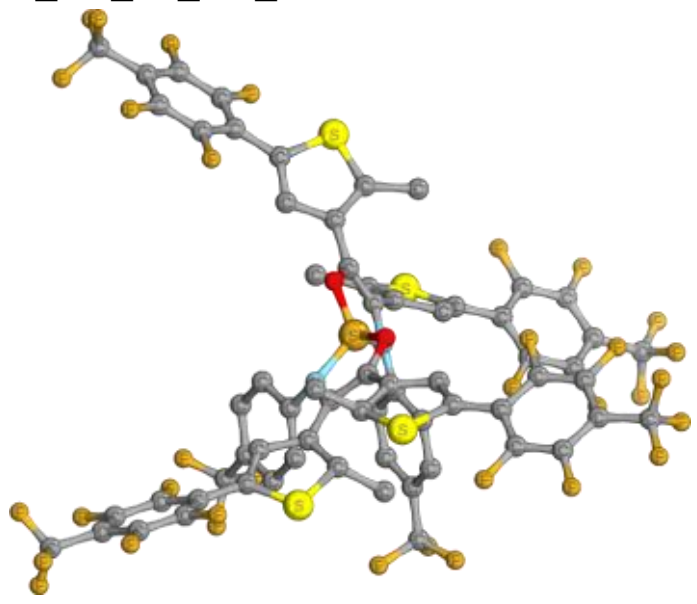

133
xyz, charge: 0, multiplicity: 1
C 0.992522 5.112637 0.380350
C 0.247124 3.993135 0.166844
C 2.191883 5.145775 -0.382930
C 2.322120 4.056350 -1.204591
S 1.011855 2.980375 -0.996813
H 0.708371 5.898962 1.064467
C -1.044095 3.661082 0.780915
C -1.995989 4.645703 1.042397
C -3.209182 4.348005 1.628794
C -3.549128 3.048469 1.986873
C -2.612894 2.057886 1.729507
C -1.395566 2.366098 1.142481
F -1.759433 5.913986 0.718370
F -4.063593 5.340209 1.842772
C -4.899099 2.806942 2.629193
F -2.832285 0.788342 2.037662
F -0.540983 1.364622 0.941948
C 3.148305 6.242513 -0.261949
C 3.399623 3.742435 -2.190139
H 3.895658 4.660873 -2.502844
H 2.991588 3.253283 -3.074916
H 4.158068 3.084365 -1.759503
H 3.203138 3.294428 1.566962
S 6.343039 2.811012 0.775113
C 10.644198 3.673430 -1.885669
C 9.815745 1.455012 -1.991886
C 10.852284 2.344136 -2.236417
C 9.459715 4.096583 -1.316974
C 6.499676 4.840215 -0.740424
C 5.331589 5.026548 0.048861
C 8.410670 3.213566 -1.068318
C 5.131588 4.004898 0.940052
C 8.628575 1.888665 -1.422387
C 4.481794 6.212269 -0.084452
C 4.055133 3.851094 1.964200
C 7.155507 3.680026 -0.467109
H 6.832649 5.552248 -1.482520
H 3.691449 4.833705 2.267922
F 11.599432 4.570941 -2.088583
F 9.347935 5.381815 -0.994649
F 9.906632 0.169042 -2.296014
C 12.179890 1.959902 -2.857025
F 7.670897 0.983999 -1.230362
H 4.420644 3.327974 2.847978
N 5.056553 7.508569 -0.132401
O 2.605523 7.518140 -0.360480
C 6.309484 7.874109 0.403194
C 6.976727 8.943687 -0.186657
C 8.136748 9.445093 0.376086
C 8.656281 8.861736 1.519281
C 8.012105 7.774276 2.094477
C 6.843050 7.284108 1.547468
H 6.326760 6.465872 2.030280
H 6.577456 9.384063 -1.093560
H 8.640747 10.284198 -0.085281
H 8.411077 7.322666 2.994148
C 9.875577 9.433502 2.185195
F 10.691036 8.478046 2.650081
F 9.539001 10.200882 3.241621
F 10.592898 10.206418 1.359467
H 6.125251 12.518611 -0.836692
C 4.161598 8.112802 4.117130
C 5.230854 8.782600 4.692485
C 5.313439 10.275842 2.812077
C 5.795656 9.869603 4.042441
C 3.665740 8.526029 2.895763
C 4.249529 9.598019 2.224119
C 3.881744 11.127780 0.277200
C 5.817716 8.280816 5.981209
N 3.795452 9.878688 0.918552
F 6.720601 7.304082 5.757026
F 4.885479 7.760562 6.791952
F 6.450185 9.243423 6.660977
H 5.790848 11.095891 2.293026
H 2.819325 8.016857 2.448539
H 6.636365 10.387949 4.484722
H 3.706998 7.273699 4.628399
C 5.966824 13.441967 -0.282003
H 6.208773 14.282944 -0.933331
C 3.928011 12.424857 0.924645
C 4.542124 13.530009 0.217516
H 1.926057 14.651148 -0.557334
C 3.224849 12.797486 2.058999
S 4.199294 14.993469 1.130551
S 3.976437 14.628206 -2.692184
C 6.660434 15.354264 -6.339101
C 3.868329 12.139267 -1.977511
C 3.447329 13.443172 -1.502883
C 4.715460 13.433636 -3.717858
C 5.986360 15.032024 -5.173348
C 6.257170 13.168174 -7.145103
C 5.587169 12.853766 -5.983178
C 5.426101 13.774451 -4.939173
C 3.819900 11.017250 -1.077438
C 4.541779 12.161667 -3.187928
C 2.032819 13.667322 -1.016665
C 6.815735 14.424434 -7.356795
O 3.787760 9.729193 -1.560427
C 3.245048 14.160110 2.316171
H 2.661829 12.097360 2.658920
F 5.067172 11.630484 -5.890801
H 1.747010 12.910224 -0.288992
C 7.533588 14.688195 -8.661936
F 6.354983 12.234164 -8.083159
F 7.151940 16.581166 -6.431678
F 5.897057 15.984019 -4.245390
H 4.949620 11.270075 -3.639163
H 6.657031 13.456035 0.562962
C 2.567864 14.842545 3.408146
C 2.351409 14.214679 4.640135
C 2.098093 16.152582 3.317419
F 2.787194 12.973159 4.847369
F 2.238986 16.833701 2.181293
C 1.713174 14.848551 5.684257
C 1.456796 16.795315 4.363287
H 1.342657 13.606861 -1.858870
C 1.247050 16.154860 5.575992
F 1.556463 14.181744 6.820754
F 1.047010 18.036504 4.146989
C 0.554452 16.779392 6.767890
Si 3.788440 8.656034 -0.304881
F -5.886397 3.205788 1.818655
F -5.008244 3.500254 3.769242
F -5.113406 1.527181 2.918453
F 12.377572 2.631531 -3.998015
F 12.263877 0.663214 -3.137776
F 13.190493 2.258654 -2.032783
F 8.041443 15.914822 -8.735888
F 6.700285 14.535864 -9.699082
F 8.544007 13.825429 -8.829013
F 1.368524 16.802928 7.830207
F 0.158668 18.028396 6.541726
F -0.529458 16.070276 7.108019

7 References

[1] L. Stoess, J. A. Davis, L. Greb, *J. Am. Chem. Soc.* **2026**.
[2] F. Neese, F. Wennmohs, U. Becker, C. Riplinger, *J. Chem. Phys.* **2020**, *152*.
[3] F. Weigend, *Phys. Chem. Chem. Phys.* **2006**, *8*, 1057-1065.
[4] F. Weigend, R. Ahlrichs, *Phys. Chem. Chem. Phys.* **2005**, *7*, 3297-3305.
[5] A. V. Marenich, C. J. Cramer, D. G. Truhlar, *J. Phys. Chem. B* **2009**, *113*, 6378-6396.
[6] S. Grimme, J. G. Brandenburg, C. Bannwarth, A. Hansen, *J. Chem. Phys.* **2015**, *143*, 054107.
[7] S. Grimme, *Chem. Eur. J.* **2012**, *18*, 9955-9964.
[8] S. Hirata, M. Head-Gordon, *Chem. Phys. Lett.* **1999**, *314*, 291-299.
[9] Y. S. Lin, G. D. Li, S. P. Mao, J. D. Chai, *J. Chem. Theory Comput.* **2013**, *9*, 263-272.
[10] H. Bohrer, N. Trapp, D. Himmel, M. Schleep, I. Krossing, *Dalton Trans.* **2015**, *44*, 7489-7499.
[11] a) P. Erdmann, L. Greb, *ChemPhysChem* **2021**, *22*, 935-943; b) P. Erdmann, J. Leitner, J. Schwarz, L. Greb, *ChemPhysChem* **2020**, *21*, 987-994.
[12] a) *S.* Bruker, *V8.40B*, Bruker AXS Inc., Madison, Wisconsin, USA; b) L. Krause, R. Herbst-Irmer, G. M. Sheldrick, D. Stalke, *J. Appl. Crystallogr.* **2015**, *48*, 3-10.
[13] a) G. M. Sheldrick, *Acta Crystallogr. C.* **2015**, *71*, 3-8; b) G. M. Sheldrick, *Acta Crystallogr. A.* **2015**, *71*, 3-8.
[14] C. R. Groom, I. J. Bruno, M. P. Lightfoot, S. C. Ward, *Acta Crystallogr. B.* **2016**, *72*, 171-179.
[15] F. D. Kratzert, V146, https://dkratzert.de/finalcif.html.
